# Health and Social Care Interventions in the 80 years Old and Over Population: An Evidence and Gap Map

**DOI:** 10.1177/18911803261462104

**Published:** 2026-06-24

**Authors:** Rebecca Abbott, Alison Bethel, Jo Thompson Coon, Morwenna Rogers, Rebecca Whear, Noreen Orr, Ruth Garside, Victoria A. Goodwin, Aseel Mahmoud, Ilianna Lourida, Debbie Cheeseman

**Affiliations:** 1 NIHR ARC South West Peninsula (PenARC), 3286 University of Exeter Medical School, Exeter, UK; 2 Department of Public Health and Sports Science (Medical School), European Centre for Environment and Human Health, Penryn, Cornwall, UK; 3 Royal Devon and Exeter NHS Trust, Royal Devon & Exeter Hospital, Exeter, UK

**Keywords:** ageing, oldest old, over 80s, evidence and gap map, evidence synthesis, healthcare interventions, intrinsic capacity

## Abstract

**Background:**

Worldwide, the population is ageing. As the population ages, so does the prevalence of age-related diseases such as arthritis, osteoporosis, diabetes, hypertension, cancer and dementia, increasing the demand on health and social care services. The evidence underpinning treatments and interventions for most health and social care issues is derived from populations younger than 80 years of age because this age group is often excluded from taking part in clinical trials. This raises concerns that many established treatments may not be the most suitable or effective approach for those aged 80 years or more.

**Objectives:**

Our aim was to produce an interactive evidence and gap map to provide an overview of the volume, diversity and nature of the evidence on health and social care interventions that target adults over 80 years of age.

**Search Methods:**

We searched 18 databases: Medline, PsycINFO, HMIC, Social Policy and Practice, Ageline, CINAHL Complete, ASSIA, PQDT; Epistemonikos; Cochrane, CENTRAL, Campbell systematic reviews, Web of Science, SCI, SSCI, AHCI, CPCI-S, CPCI-SSH, and ESCI (in October 2022). Searches were updated in July 2024. Forward and backward citation searching was also undertaken in 2024 using CiteSearch, Scopus and Google Scholar.

**Selection Criteria:**

We included systematic reviews, randomised controlled trials (RCTs) and primary qualitative studies in the map that focused on the effectiveness and/or experience of any health or social care interventions for adults aged 80 years or more. All studies were independently screened for eligibility by two people at both title/abstract and full text stages.

**Data Collection and Analysis:**

Interventions were categorised in line with the WHO definition of five domains that facilitate healthy ageing: building and maintaining intrinsic capacity, health services models and approaches, enabling environments and technologies, building and maintaining relationships and learning, growing and making decisions. Interventions could cut across multiple domains. The data extraction tool was developed on EPPI reviewer and was modified and tested through piloting and revising by the core team. The tool was informed by the research question and the structure of the map. As well as extracting data on population characteristics, intervention domain and sub-categories, we extracted additional data to enable filters, such as specific health conditions, and equity characteristics. Standardised tools were used to assess study quality for all studies: AMSTAR-2 for systematic reviews; Cochrane Risk of Bias tool (version 1) for RCTs; and the Wallace criteria for primary qualitative studies. Data extraction and quality appraisal were extracted by one person and checked by a second.

**Main Results:**

We included 172 studies: 36 systematic reviews, 120 RCTs and 16 primary qualitative studies. Most of the systematic reviews were assessed as low or very low quality with only five assessed as moderate to high quality. Similarly, most of the RCTs were assessed to be at medium to high risk of bias, with only 27 RCTs assessed with an overall low risk of bias. Ten of the qualitative studies were assessed as high quality. Over a third of the studies (n = 67) in the map have been published since 2020. The majority of the evidence, over 90% (n = 157/172), was focused within the domain of *building and maintaining intrinsic capacity*, and within this domain, on either surgical and medical procedures (n = 75) or medicines and medical technologies (n = 54) in predominantly the cardiovascular, neuromuscular and digestive areas. Rehabilitation and behavioral interventions were also represented. Only a small number of studies focused on health conditions linked to ageing: frailty a focus of research in 14 studies, and falls in six studies. Only two studies addressed mental health therapies, and there were no studies focused on skin conditions, genitourinary health and or voice/speech conditions. The second most frequently represented domain was *health service models and/or approaches* (n = 38), predominantly relating to home visits (n = 15, all RCTs) and comprehensive geriatric assessment and integrated care (n = 5, all RCTs). The three intervention domains of *enabling environments and technologies*, *building and maintaining* relationships and *learning, growing and making decisions* were poorly represented in the evidence. The majority of studies measured physiological outcomes of health, such as measures of functional health, chronic health markers or symptoms, and adverse events; few studies assessed measures of well-being or psychosocial health. Sixteen studies reported on experiences of interventions, mostly from the experience of the older adult (n = 9) and were in relation to heart surgery and procedures, colon surgery, resuscitation, medicines review and preventative screening. Only 7% of outcomes in the map were in the psychological health and wellbeing category. Social health (including connectedness and participation) was not featured as an outcome in any studies. Studies were primarily conducted in Europe and Asia: these two regions representing over 75% of the evidence. There were several gaps evident in the map, including but not limited to, end-of-life care (including advance care planning) and healthcare delivery such as hospital-at-home and telehealth. Several potential research areas where synthesis might be valuable were also identified such as medicines optimisation, home visits, and specific health conditions such as osteoporosis treatments.

**Authors’ Conclusions:**

As the worldwide population continues to age, it is increasingly important that we have evidence of appropriate effective interventions for those who have reached their 80s, 90s and beyond, a group often left out of trials. This evidence and gap map shows that currently there is a clear bias towards interventions orientated around a biomedical view of health focused on intrinsic capacity, and relatively little on the wider functional and psychosocial aspect of health, or on enabling environments, such as adaptations to health and care services, or models of care. There is also a clear need for more research to understand the experiences and preferences of interventions from adults aged 80 years or more.

## Plain Language Summary

**Research exclusively focussed on people aged over 80 targets medical treatments and surgical procedures, neglecting healthcare services or wellbeing, and much of it is poor quality**.

**The Evidence and Gap Map (EGM) in brief:** Most of the studies in people aged over 80 investigate medical procedures and treatments with a focus on basic health needs and the ability to function, with fewer studies about health and care services or wellbeing.

**What is this EGM about?:** Our population is ageing, which means that people are living longer, and age-related illnesses such as arthritis, dementia and cancer are becoming more common. Studies investigating interventions (treatments, procedures, therapies and services) are often focused on people aged under 80, because those who are older are often excluded from taking part in trials. Therefore, the treatments that are considered best practice in a general population might not be the best option for people aged over 80. This EGM displays evidence of interventions from systematic reviews and randomised controlled trials (RCTs) and studies looking at experiences (qualitative studies) where the people being investigated were aged over 80.

**What is the aim of this EGM?:** The aim of this EGM is to provide easy access to studies that investigate interventions for people aged over 80. The studies are grouped by types of interventions and outcomes. Overall, the EGM provides an overview of the volume and nature of evidence on interventions for this age group.

**What studies are included?:** The EGM contains 172 studies investigating interventions for people aged over 80. The interventions are organized into five groups: *building and maintaining intrinsic capacity* (ability to function both physically and mentally), *health services an/or approaches*, *enabling environments and technologies*, *building and maintaining relationships* and *learning, growing and making decisions*. The EGM contains 36 systematic reviews, 120 RCTs and 16 qualitative studies.

**What are the main findings of this gap map?:** Research in older people is becoming more common as the population ages. Over a third of studies (n = 67) in the map have been published since 2020. However, most of the research was moderate to poor quality. Most of the systematic reviews (n = 31) and RCTs (n = 93) were rated as low or very low quality.

Only a small number of studies focused on health conditions linked to ageing, with frailty covered in 14 studies, and falls in 6 studies.

Most of the studies (n = 157) were about *building and maintaining intrinsic capacity,* focusing on the treatment and/or prevention of diseases and health disorders. The categories with most studies were those that involved surgery (n = 75 studies), and those that involved medicines or medical devices (n = 54). Only two studies addressed mental health therapies, and no studies examined ‘end of life’, skin conditions, genitourinary health and or voice/speech conditions.

Several studies (n = 38) examined *health services and/or health approaches*. Some of these (n = 15) were RCTs assessing the effectiveness of home visits and a smaller number (n = 5) assessed geriatric assessment. Surprisingly, there were no systematic reviews in the map covering those topics. The only systematic review in the health services category investigated admitting older adults with sepsis into intensive care units. Similarly, there were no systematic reviews in the categories of *enabling environments and technologies* and *learning, growing and making decisions*.

The most common outcomes reported in the studies were physiological measures of health (such as blood cholesterol levels, or blood pressure), measures of physical function (such as strength, balance), chronic health symptoms and side effects and complications. Only 7% of outcomes in the map were in the psychological health and wellbeing category. Social health (including connectedness and participation) was not featured as an outcome in any studies.

Sixteen studies reported on experiences of interventions, mostly from the experience of the older adult (n = 9) and were in relation to heart surgery and procedures, colon surgery, resuscitation, medicines review and preventative screening.

**What do the findings of the map mean?:** This EGM provides information on systematic reviews, RCTs and qualitative studies that have examined the effectiveness of interventions for people aged over 80. This is important because people in older age groups are often excluded from research, meaning the interventions that are best for the general population might not be most suitable for them. While studies in people aged over 80 focus on basic health needs, older people are under-represented in studies about psychosocial aspects of health or enabling environments such as adaptations to health and care services. There is also a need for more studies examining how people aged over 80 experience treatments, therapies and services.

**How up-to-date is this EGM?:** The EGM contains studies published up until July 2024.

## Background

### The Problem, Condition or Issue

World-wide, the population is ageing. The proportion of older adults aged 65 years or older increased in most countries over the past decade and this rise is expected to continue (Vanhuysee, 2023). Moreover, the World Health Organization (WHO) (World Health Organisation, 2022) predict that the number of older adults aged 80 years and above globally will triple between 2020 and 2050 and expected to reach 426 million. In the UK alone, as of 2021, there are 3.3 million adults aged 80 years or more (Office for National Statistics (ONS), 2023), and this group is expected to double by 2047 (Office for National Statistics (ONS), 2025).

Population ageing is accompanied by increased prevalence of age-related diseases such as arthritis, osteoporosis, diabetes, hypertension, cancer and dementia. For example, for cancer incidence rates, in the UK in 2016-2018, across each year more than a third of new cases were in people aged 75 and over (Cancer Research UK, 2021). Furthermore, the likelihood of developing multiple long-term conditions (having two or more long-term physical or mental health conditions) increases with age, and this is reported to be globally increasing across lower, middle- and high-income countries (The Academy of Medical Science (AMS), 2018). The prevalence of disability also increases with age: a longitudinal study of those over 90 years in the US found difficulties in activities of daily living present in over 75% of adults aged 90 or more, increasing to 97% in those over 100 years. Needing help with activities of daily living was present in 44% and 92% respectively (Berlau et al., 2009). An ageing population is likely to place greater demands on health services, as evidenced by reports of a 50% increase in people over the age of 75 years being treated in an English hospital in 2014-2015, compared to 2006-2007 (Lin et al., 2016). In Australia, women aged 85 years or more represent the largest proportion of emergency surgical admissions (Australian Institute of Health and Welfare (AIHW), 2014).

There are also issues relating to age related health conditions for those living independently. Recent WHO estimates suggest that the proportion of older adults with significant and moderate loss of functional ability is two times greater among adults aged 80 years and over than those aged 60 to 70 years (World Health Organisation, 2020). Furthermore, in the UK, over half of all people aged 75 and over live alone and it is predicted that between 2008 and 2031 the increase in those aged 75 years and over living alone will be 38% (Office for National Statistics (ONS), 2010). As such, many older people will have a reduction in functional ability and require support, which if not provided by someone living with them (such as an unpaid carer i.e., spouse or child), will need to be provided by formal services, unless they can optimise their function such that support is not required. All older adults, irrespective of the level of intrinsic capacity, should have opportunities to optimise functional ability to enjoy what they value most.

The evidence underpinning the treatments and interventions for most health and social care issues is likely to have been derived from populations younger than 80 years of age. This is likely due to older adults, particularly the oldest of age categories, being excluded from trials due to their comorbidities (Lau et al., 2022), or the nature of a changing demographic itself, such that they represented a much smaller group when the primary research was undertaken. There have been concerns raised that both the effectiveness and the suitability of many established treatments may not be either suitable, or the most effective approach, for those in their 80s or 90s. For example, the way bodies respond to medications can change, with drugs having a stronger or weaker effect than intended, meaning older adults might experience more intense side effects or reduced benefits from treatment (Ngcobo, 2025). Age-related physiological changes and co-morbidities that increase risk of complications, may also impact decisions around the suitability of healthcare interventions (Eriksson et al., 2025). Indeed, 80 years has been shown to be the inflection point of rapid decline across various indicators of cardiorespiratory and physical health (Shen et al., 2024). Furthermore, whilst meta-analyses of effectiveness trials widely used to inform evidence-based practice can provide useful insights to average effects of interventions, they often are not able to offer robust conclusions for sub-groups, such as those in the oldest age categories (Clegg et al., 2022).

In terms of social care, Morgan et al. (2020), reported that there is a growing recognition of the number of family caregivers who are older adults themselves living with complex health conditions, and that the research on this at-risk group has predominantly examined the experience of caregivers aged between 60–75 with little known about the increasing number of caregivers who are over-75. Morgan et al. (2020) also highlighted the increased risk of ageing in terms of health issues experienced and declining social networks, and the impact this could have on both care-giving and the care-giver.

### The Intervention

Healthy ageing is relevant to everyone, not just those who are currently free of disease. The WHO defines healthy ageing as the process of developing and maintaining the functional ability that enables well-being in older age (Rudnicka et al., 2020). The WHO suggest that functional ability is “determined by the intrinsic capacity of the individual (that is, the combination of all the individual’s physical and mental capacities), the environments he or she inhabits (understood in the broadest sense and including physical, social and policy environments), and the interaction between these” (World Health Organisation, 2020, p. 1).

We took this health systems perspective in our consideration of interventions, broadening health care to include social care interventions that work together to impact functional ability. As such, for the purposes of this EGM, we were interested in interventions that impact functional ability: either directly on the intrinsic capacity of an individual, or on functional ability more broadly through health and social care interventions. We also were interested in considering determinants of health inequity.

### Why it is Important to Develop the EGM

The population aged 80 years and over is growing alongside increasing research on this population (Gonzalez-Alcaide et al., 2021). The number of publications per year focused on the topic of what has sometimes been termed the ‘oldest old’, has steadily increased for over three decades (Lund & Wang, 2020). In addition to focusing on the pathologies causing the greatest mortality and morbidity in this population, such as cardiovascular disease, there is increasing interest in health research related to ageing related conditions (for example frailty, falls, incontinence, skin breakdown), social aspects, and factors related to preserving quality of life and promoting healthy ageing. However, it is unclear the extent to which this research has an intervention-focus and whether it has specifically targeted those over 80 years, evidence that is important for decision makers and older people themselves (and their families), and whether there are gaps in the evidence.

Due to the rapid increase of research in the oldest age groups over the past three decades, an EGM of available intervention evidence will help stakeholders and decision makers identify where there is sufficient evidence for this age group. The map will also help researchers identify where there might be a need for evidence synthesis, but more likely in this area, where more evidence is needed.

## Objectives

The objectives of this EGM were to:

• Identify available systematic reviews and randomised controlled trials (RCTs) on interventions targeting health or social needs of people aged over 80
• Identify qualitative studies relating to the experiences of people aged over 80 of interventions that target their health or social needs
• Identify areas where systematic reviews are needed
• Identify gaps in evidence where further primary research is needed
• Assess equity considerations (using the PROGRESS plus criteria) in available systematic reviews, RCTs and qualitative studies of identified interventions
• Assess gaps and evidence related to health equity.

Our specific research questions were:

• What is the scale and focus of interventions in health and social care designed for or evaluated on those aged 80 years and over?
• What is the nature and breadth of qualitative evidence relating to how those aged 80 years and over perceive interventions aimed at their health and social wellbeing?
• What is the evidence and/or gaps in the research in this population related to health equity?

## Methods

The protocol for this EGM has been published (Abbott et al., 2023).

### Evidence and Gap Map: Definition and Purpose

Evidence and gap maps (EGMs) are used to highlight what research on a topic is available, alongside highlighting gaps in research to inform strategic health and social policy, program and research priorities (Welch et al., 2021). EGMs can identify areas for which there are no or few primary studies, or many studies, but no systematic reviews and can also highlight areas in which there are many reviews to indicate where a review of reviews may be appropriate (White et al., 2020). The purpose of EGMs is to allow users to identify and access the research evidence (or evidence gaps) most relevant to their population and intervention focus.

### Framework Development and Scope

We found no existing widely accepted framework that was relevant to the breadth of health and social care interventions for this particular age group. Therefore, we used the definition of healthy ageing and its concepts relating to intrinsic capacity, the environment and the interaction between the two to inform the basis of our framework for this EGM (World Health Organisation, 2020). The WHO definitions for functional ability, intrinsic capacity and the environment are provided below:

• Functional ability is defined as “all the health-related attributes that enable people to be and to do what they have reason to value.” (p. 2) Five sub-domains are proposed: meeting basic needs, learning and making decisions; mobility; building and maintaining relationships; and contributing to families, communities or society.
• Intrinsic capacity at any point in time is “determined by many factors, including underlying physiological and psychological changes, health-related behaviours and the presence or absence of disease.” (p. 2) Five sub-domains are proposed: neuromusculoskeletal, sensory, metabolic, cognitive and psychological.
• Environments “that people inhabit and their interaction with them are also major determinants of what older people with a given level of intrinsic capacity can do. These environments provide a range of resources or barriers that will ultimately decide whether older people can engage or participate in activities that matter to them.” (p. 2) Five sub-domains are proposed: products and technology, natural and built environment; support and relationships; attitudes; and services, systems and policies.

### Stakeholder Engagement

As methodologists we recognise the importance and value of people with lived experience of caring for people over 80 and so invited people with experience of acute geriatric care, rehabilitation for older adults, community pharmacy and dementia to join the authorship team.

We consulted with additional wider topic experts (older adult academic, community rehabilitation academic, practicing public health practitioner (older adult focus)) at the start of the project when we were first developing the framework (for purposes of sense checking and ensuring we had captured the broad spectrum of interventions and likely outcomes) and when we first drafted the map (to understand its usability and to explore their perceptions about what it found, what was surprising and what was missing from their perspectives).

We also had meetings with older adults who are part of the PenARC patient engagement group (PenPEG) to define the scope of the EGM and develop the framework with respect to identifying relevant interventions and outcomes. Our early meeting with PenPEG highlighted the dislike of the term ‘oldest old’ which is how those aged 80 years are often referred to within the academic literature, and therefore this project, originally called the Oldest Old EGM was renamed the ‘Over80s EGM’. We returned for further discussions with PenPEG when we first drafted the map to establish how the early findings of the map resonated with their understandings and whether they thought there were areas of evidence not covered within the map and what surprised them. These comments are detailed in Section ‘*Stakeholder engagement through the EGM Process’*.

### Conceptual Framework

**Figure 1.**
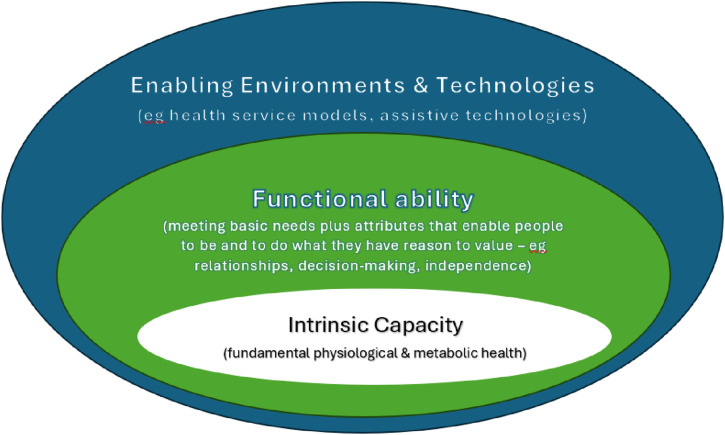
Conceptual model to inform development of the framework for the Evidence and Gap Map

We used the definition of healthy ageing and its concepts relating to intrinsic capacity, the environment and the interaction between the two to inform the basis of our framework (World Health Organisation, 2020). Alongside this, we considered the multidimensional model of healthy ageing, proposed by Rivadeneira et al. (2021), which is also based on the central role of functional ability in healthy ageing. These authors suggest that intrinsic capacity covers the concepts of (1) physiological and metabolic health, (2) geriatric syndromes, (3) risk factors, (4) physical capacity, (5) cognitive capacity, and (6) psychological well-being. The conceptual framework reflects a combination of these two models and is depicted in Figure 1.

### Dimensions

#### Interventions (Rows in the Map)

Interventions were broadly categorised into five domains:

• ‘building and maintaining intrinsic capacity’
• ‘health service models and/or approaches’
• ‘enabling environments and technologies’
• ‘building and maintaining relationships’ and
• ‘learning, growing & making decisions’.

Within these domains, interventions were further categorised and for some categories, additional sub-categories were needed. Please see Table 1 with examples of interventions within these categories and where relevant, sub-categories.

**Table 1.** Intervention Coding Framework (Interventions Could Cut Across More Than One Domain and Across Several Sub-categories Eg as Part of a Multicomponent Intervention)

Healthy Aging Domain	Category	Sub-category	*For example:*
Building and maintaining intrinsic capacity	Surgical & medical procedures	Cardiovascular, haematological, immunological, respiratory	*Heart-valve surgery, angioplasty, coronary perfusion, lung surgery, cancer surgery, stem cell transplants*
		Digestive, metabolic, endocrine	*Bowel resection, colorectal surgery*
		Genitourinary, reproductive	*Bladder surgery, prostate surgery*
		Neuro-musculoskeletal	*Knee &/or hip replacement, spinal surgery*
		Skin and related structure	*Skin surgery*
		Voice and speech	*Larynx, tongue surgery*
		Sensory	*Eye, ear, nose and throat surgery*
		Mental function	​
	Medicines and medical technologies	Medicines optimisation, review	*Community pharmacist medicines review, deprescribing intervention*
		Medical technologies	*Coronary stents, pulmonary function technology, colonoscopy preparations, hearing aids e.g. BNF2 (CVD) statins, BNF8 (malignant disease) chemotherapy, BNF10 (neuromusculoskeletal) strontium*
		Medicines – classed via BNF coding 1-15	
		Chinese medicine	*Chinese herbal medicine*
	Physical/rehabilitation	Physiotherapy	*Multi-component falls prevention*
		Occupational therapy	*Home modification intervention, activities of daily living improvement*
		Speech therapy	*Swallowing interventions*
		Resistance training	*Muscle strength intervention, circuit training*
		Stretching/Mobility	*Stretching & flexibility classes*
		Balance	*Balance training*
		Aerobic training	*Aerobic training*
		Other	*Whole body vibration*
	Behavioural/Lifestyle	Physical activity	*Walking interventions, community exercise classes*
		Yoga/Tai chi	*Yoga, tai chi, Qi gong classes*
		Diet	*Calorie counting,*
		Relaxation/Breathing/Meditation	*Auricular acupressure, community relaxation classes*
		Self-management	*Self-care management interventions*
	Oral/Nutritional	Supplementation/fortification	*L-Carnitine, branched chain amino acids, protein supplements, minerals & vitamins probiotics*
		Therapeutic diet	*Individualised nutrition plans*
		Hydration/fluid restriction	*Hydration interventions, fluid intake monitoring, fluid restriction diets*
		Dental	*Oral health care interventions, access to dental services*
	Psychological	Mindfulness	*Mindfulness training, Art-base therapy, Music-assisted reminiscence therapy*
		Cognitive behavioural therapy	
		Counselling	
		Reminiscence therapy	
		Sensory therapy	
		Art therapy	
	Cognitive health	Cognitive training	*Video games, Computerised training (‘Brain games’)*
		Other	
	Diagnostic	Screening	*Mammography*
		New technologies	​
Health service models & approaches	Comprehensive geriatric assessment	​	*Comprehensive preventative home visit intervention; Multifactorial assessment and targeted falls intervention*
	Integrated care		*Integrated home-based health interventions*
	Hospital at home		*Hospital-at-home intervention; virtual wards*
	Case management		*Individualised needs assessment & care planning*
	Mobile screening		*Mobile health screening, eg mammography, eye screening*
	Telehealth		*Telephone and/or video consultations*
	Discharge planning		*Pharmacist assisted discharge planning*
	Home visits		*Home based medication reviews, Nurse in-home health consultations*
	Specialist unit		*Intensive Care Unit, Geriatric wards for people living with dementia*
Enabling environments and technologies	Natural & built environment: Home modification	​	*Lighting interventions, home safety interventions, home modification*
	Natural & built environment: Specific building/construction		*Geriatric units for people living with dementia*
	Products & technology: Wearables		*Step counter interventions*
	Products & technology: VR/egames		*Video games for physical activity, Virtual reality for balance training*
	Products & technology: Smart technology		*Safety/Alarm centred smart technology*
	Products & technology: Personal mobility		*Mobility scooters, walking frames*
Building & maintaining relationships	Volunteer interventions	​	*Befriending schemes, ready meals/community food services*
	Animal assisted interventions		*Pet therapy*
	Intergenerational interventions		
	Peer support		
	Carer support		
	Skills/Craft		*Community dance initiatives*
Learning, growing & making decisions	Advance care planning	​	*ACP interventions*
	Information provision/Education		*Leaflets, websites*
	Decision-making		*Decision tools*

#### Outcomes/Experiences (Columns in the Map)

Outcomes were broadly categorised, firstly by whether they were reporting on experience of the intervention (via a primary qualitative studies), and then by whether they were related to physiological and/or psychosocial health outcomes, medicines-related outcomes, health services and resources outcomes, or process outcomes. The specific outcome categories were:

• Experiences of interventions (e.g. experience of procedure, activity, medicine, technology) from participants, family members, health care professionals and relevant stakeholders
• Physiological health (e.g. blood pressure, fitness, bone density, BMI, strength)
• Physiological events (e.g. myocardial infarction, stroke, falls)
• Functional/physical health (e.g. activities of daily living, mobility)
• Chronic health symptoms (e.g. pain)
• Medicines related (e.g. medicine optimisation/deprescribing rates/adherence)
• Psychological health and wellbeing (e.g. life satisfaction, wellbeing, loneliness, anxiety, depression)
• Cognitive health (e.g. cognition, memory)
• Social health (e.g. connectedness, participation)
• Service/Resource use (e.g, hospital admission, care home admissions, primary care visits, cost)
• Process-related (e.g. acceptability)
• Adverse events (e.g. death, bleeding, nausea, pain)

### Types of Study Design

We were interested in effectiveness and experience of interventions to inform decision making and to inform future research. We, therefore, included systematic reviews, RCTs and qualitative studies related to relevant (health and social care) interventions.

Systematic reviews: systematic reviews could have evaluated RCTs, non-RCTs, controlled and uncontrolled before-and-after trials, interrupted time series designs or have been a qualitative synthesis relating to an intervention. All systematic reviews, irrespective of AMSTAR quality (Shea et al., 2017), were included. To be included as a systematic review, the review needed to report (i) a research question, (ii) search sources and a reproducible search strategy, (iii) inclusion and exclusion criteria, and (iv) selection methods (adapted from (Krnic Martinic et al., 2019)). We *excluded* scoping reviews, narrative reviews or any type of evidence synthesis described as a review (systematic or not) that did not fulfil the four criteria described above.

RCTs: We included RCTs on interventions targeting health or social needs of the people aged over 80. We excluded quantitative studies that focussed on predictive factors, prognostic and diagnostic studies. Quantitative studies that were not RCTs were excluded.

Qualitative studies: We included qualitative studies that evaluated participant experience of interventions, but also clinician, family member or other relevant stakeholders perception/experience of interventions. The qualitative study did not have to have been part of a RCT.

We included on-going systematic reviews and RCTs. We also included studies published in grey literature such as reports, dissertations, and conference abstracts, if they met our study design eligibility criteria.

### Types of Intervention/Problem

We were interested in any health or social care intervention targeted at individuals aged 80 years or more. As noted above in the dimension section, we expected the interventions to be heterogenous, but that all would target one or more of the three dimensions relating to healthy ageing: intrinsic capacity, functional ability or the environment.

We did not include studies that did not specify an intervention. For example, studies that explored what people over 80 years considered important for quality of life, or barriers to accessing services in general, were not eligible.

### Types of Population

We included studies if they used a minimum ‘age cut-off’ of adults aged 80 years or more. Qualitative studies of carers and health or social care professionals needed to have been delivering interventions to those aged 80 years or more.

### Types of Outcome Measures

All outcomes or experiences relating to health and social care of any eligible intervention were of interest. We were also interested in the process measures of interventions and the impact of interventions on health service resource.

### Other Eligibility Criteria

We included interventions in any setting. Settings could be the individual’s place of residence (such as residential homes, apartments, long-term care facilities, hospices, nursing homes), but could also be in acute/sub-acute hospital and convalescent care settings. We coded the settings so that the evidence can be filtered according to setting. The settings were coded as: community (own home, community centre, day care centre), residential care, assisted living/retirement community, primary care, hospital inpatient, hospital outpatient, hospice, specialist unit (such as intensive care).

Due to the increased ageing demographic over recent decades, we restricted eligibility to publications since 1990. All articles before 1990 were excluded.

The review was not restricted by geographical area.

### Search Methods and Sources

The search strategies were developed and peer reviewed by two information specialists (AB and MR). The searches were precise and utilised both free text and relevant controlled vocabulary along with study search filters for systematic reviews, qualitative studies and RCTs.

The first searches were undertaken 24th-31st October 2022 in 18 databases: Medline (1946-), PsycINFO (1806-), Health Management Information Consortium (HMIC) (1979-), Social Policy and Practice (1890) all via Ovid; Ageline (no dates available), CINAHL Complete (1937-) via EBSCOhost; Applied Social Sciences Index & Abstracts (ASSIA) (1987-), Proquest Dissertation and Theses Global (PQDT) (1637-) via ProQuest; Epistemonikos (no dates available) via website; Cochrane Database of Systematic Reviews (CDSR) (1996-), Cochrane Central Register of Controlled Trials (CENTRAL) (1908-), Campbell systematic reviews (2000-) via Wiley; and the Web of Science databases: Science Citation Index (1990-), Social Science Citation Index (1990-), Arts & Humanities Index (1975-), Conference Proceedings Citation Index - Science (1990-), Conference Proceedings Citation Index – Social Science & Humanities (1990-), and Emerging Sources Citation Index (2015-). Once downloaded, a date restriction of 1990 was applied and any books, book chapters and conference proceedings were removed. From this search, 148 articles were included. The original Search Summary Table (Bethel et al., 2021) (see Appendix – *original search summary table*) showed that 140/148 were in Medline and that the search developed for the database retrieved 118/140. This led to the Medline and some of the other database searches being amended (more terms added, none were removed) for the update search in July 2024.

Forward and backward citation searching was undertaken in July 2024 using CiteSearch, Scopus or Google Scholar using the 152 included articles from the database searches. Once downloaded and deduplicated, 12311 records remained. A targeted ‘simple’ search was carried out to identify RCTs, reviews and qualitative studies within Endnote using the terms ‘review’, ‘random’, ‘trial’ or ‘qualitative’. Testing had shown that combining these terms in Endnote retrieved all of the existing included studies.

A final search summary table with the total number of included studies is available in the Appendix.

### Analysis and Presentation

#### Report Structure

In addition to presenting a description of the included studies primarily according to intervention and outcome/experience categories, the report provides tabulations or graphs of the number of studies, with accompanying narrative description by: year (in decades), study quality, setting and country (designated by country of first author for systematic reviews or where trial undertaken).

#### Filters for Presentation

EGMs traditionally present interventions in rows and outcomes in columns, allowing the user to choose a particular area of interest if desired. In order for the user to view certain study types, or focus on specific settings or ages, the following filter categories have been made available:

(1) Type of study: systematic review, RCT or primary qualitative study
(2) Quality of Study: the EGM can be filtered by summary assessment of study quality. See section 3.13.3 for details of the tools used for summary assessments
(3) Study region: by world region, or by individual country
(4) Year study published: in decades
(5) Setting: community, residential care, assisted living, primary care, hospital inpatient, hospital outpatient, hospice, specialist unit
(6) Population characteristics: participant sex, age (all, >90 yrs, >100 yrs), PROGRESS plus characteristics, broad health condition, ageing related conditions

#### Dependency

Each entry in the map is a systematic review, a RCT or a qualitative study. The final EGM identifies the number of entries covered by the map in each sector or subsector. We have included all relevant systematic reviews and RCTs irrespective of whether there is overlap between reviews and trials. Similarly, studies with multiple interventions or multiple outcomes may appear several times within the map.

### Data Collection and Analysis

#### Screening and Study Selection

The titles and abstracts of records identified by bibliographic and supplementary search methods were screened against inclusion criteria by two independent reviewers (BA, AB, JTC, RW, MR, NO) looking for reasons for exclusion. The full text of records retained at this stage were retrieved and screened for inclusion against the inclusion criteria using the same process. All included studies were saved in a master library using Endnote v21.2. These studies were then imported into EPPI reviewer where the remaining data extraction, coding and management were conducted.

#### Data Extraction and Management

Data extraction was undertaken by one reviewer (BA) and checked by a second (AB, JTC, RW, MR, NO) with any inconsistencies identified and resolved through discussion. Initially, the data extraction template was modified and tested by both topic and methods expert within the review team, and again subsequently through piloting. The extraction and coding were informed by the research question and the structure of the map. Data extraction was conducted on the EPPI reviewer platform (Thomas et al., 2020). We extracted data on study design, year, geographical location, setting, population (age, gender, health condition/status, equity characteristics), intervention and outcomes/experiences. When more than one article had been published for a single study, all available information from sibling papers was used for coding. It was possible for studies to involve multiple intervention types.

We used the PROGRESS-Plus framework (O’Neill et al., 2014) to identify studies that reported on equity characteristics and those that measured effects of interventions by gender or other factors that may lead to health inequalities. PROGRESS-Plus stands for Place of residence, Race/ethnicity/culture/language, Occupation, Gender or sex, Religion, Education, Socioeconomic status, Social capital. ‘Plus’ represents other factors associated with discrimination, exclusion, marginalisation or vulnerability such as personal characteristics, relationships that limit opportunities for health, or environmental situations which provide limited control of opportunities for health.

#### Tools for Assessing Risk of Bias/Study Quality of Included Reviews

Quality assessment of systematic reviews, RCTs and qualitative primary studies was conducted to indicate confidence in study findings. All assessments were performed by one reviewer and checked by a second, with disagreements settled through discussion. We did not exclude any study based on study quality.

##### AMSTAR-2

All systematic reviews included following full-text screening were appraised using the AMSTAR-2 quality appraisal tool (Shea et al., 2017) for systematic reviews of primary studies of randomised and non-randomised study designs. AMSTAR-2 is a 16-item checklist, covering all aspects of the conduct of a systematic review, from pre-specifying a protocol to appropriately analysing and discussing risk of bias.

Items 2, 4, 9, 11, and 13 of the checklist were considered “critical” in assessing overall study quality, with studies rated from high to critically low depending on the number of weaknesses (Shea et al., 2017). A rating of “high” means the study has no more than one noncritical weakness, “moderate” that there is no critical weakness but more than one noncritical weakness, “low” that a study has one critical weakness, and “critically low” that a study has more than one critical weakness. Low and critically low studies may also have multiple noncritical weaknesses.

##### Cochrane Risk of Bias

We used the Cochrane ROB tool (Higgins & Green, 2011) to assess risk of bias of the included RCTs. As our EGM focussed on interventions that could have multiple outcomes, we were assessing all outcomes that met our inclusion criteria in the included studies for risk of bias. We, therefore, decided to use ROB rather than ROB2, as this tool is more suitable for considering multiple outcomes.

ROB considers trial design, conduct and reporting, focusing on six domains related to the internal validity of the study: sequence generation, allocation concealment, blinding of participants and personnel, blinding of outcome assessment, incomplete outcome data, and selective outcome reporting. An additional domain called ‘other bias’ was available for other aspects of risk of bias in the study not identified by the six domains. Review authors discussed the application of the domains to the studies throughout the process of critical appraisal. For the selective reporting domain, as we were not performing a meta-analysis, we considered risk of bias to be low if all measured outcomes were reported in the results; these could be in any format.

If one area amongst the primary six domains was rated as a ‘high risk’ of bias or ‘unclear’, the study was categorised as medium quality, and if 2 or more areas were rated as ‘high risk of bias’ and or ‘unclear’, the study was categorised as low quality (Higgins & Green, 2011).

##### Wallace

Methodological robustness of the primary qualitative studies was assessed using the Wallace criteria (Wallace et al., 2004). A reflection of methodological quality was assigned according to the number of “yes” responses during quality appraisal, with a higher quality paper scoring ≥10 (out of the possible 14) “yes” responses.

#### Methods for Mapping

We used EPPI-Reviewer software (Thomas et al., 2020) for data extraction and coding, and to generate the online EGM (EPPI-Mapper, 2022). The map is interactive so that users can click on (i) cells within the matrix to show a list of the relevant studies and on (ii) study names to access the study or a reference and database link for the study.

## Results

### Description of Studies

#### Results of the Search

**Figure 2.**
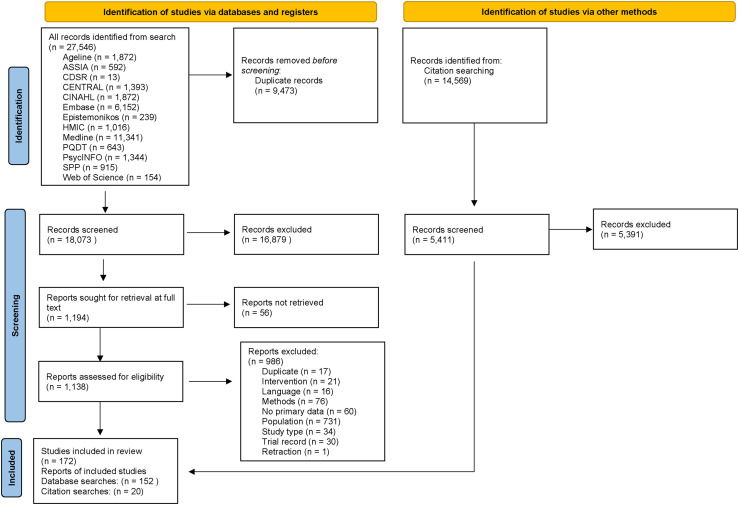
PRISMA flow diagram providing details on the screening process and decisions at each stage

Our databases searches identified 27,546 records (reduced to 18,703 after removal of duplicates), and our citation searches identified a further 5,411 records. After both stages of screening had been completed, a total of 172 research articles were included (see Appendix – *list of included studies*). Figure 2 shows the PRISMA flow diagram which provides further details on the screening process and decisions at each stage (Page et al., 2021).

#### Excluded Studies

The main reasons for exclusion at the full-text screening stage were due to age of population (n = 731 - primarily studies using an age cut off <80 years), and study design (n = 110 – studies not randomised). See supporting information material for table of excluded studies with their associated references. (see Appendix – *table of excluded studies*).

### Synthesis of Included Studies

We included 172 articles that focused on health or social care interventions for people aged 80 years or more. Of these articles, 36 (20.9%) were systematic reviews, 120 (69.8%) were RCTs and 16 (9.3%) were primary qualitative studies. No ongoing reviews or studies were identified through trial and review protocol registries. The link to the map is available here: https://eppi.ioe.ac.uk/cms/Portals/35/Maps/80plus.html

Since many of the studies included in this EGM have been coded under more than one intervention category, a single study may appear in multiple cells. See supporting information on the interactive EGM map.

**Figure 3.**
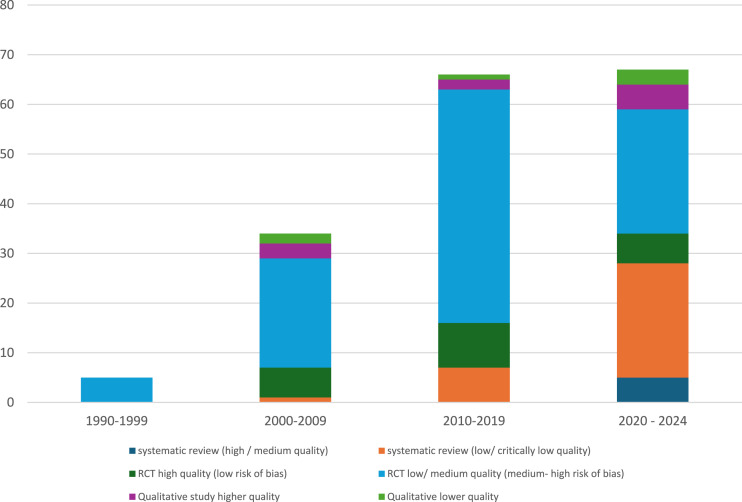
Increasing number of articles on health and social care interventions for those aged over 80 years

Of the included publications, over a third of them (38.9%) have been published in the past four years (systematic reviews: *n* = 28; RCTs: *n* = 31, and qualitative studies: n = 8), see Figure 3.

#### Study Populations

Included studies were predominantly focused on interventions for adults with a minimum age of 80 years, but a small number targeted, or included separate analyses for, nonagenarians: six systematic reviews (Bai et al., 2021; Biancari et al., 2017; Hajibandeh et al., 2021; Iqbal et al., 2022; Noguchi et al., 2022; Yan et al., 2019), five RCTs (Cadore et al., 2014; Campo-Prieto et al., 2022; Carral et al., 2019; Ruiz et al., 2015) and two qualitative studies (Instenes et al., 2024; Kylberg et al., 2013); and one RCT focused on centenarians (Malaguarnera et al., 2007). Most studies included mixed gender populations, with 11 RCTs and two qualitative studies targeting women only, and one RCT and one qualitative study focused only on men.

Ageing related conditions specified as a focus of the intervention were only present in a small proportion (22/172) of the included studies. Of these, the most common condition addressed was ‘frailty’ which was the subject of interest in 14 articles (12 RCTs, one systematic review and one qualitative study), followed by ‘falls’ in six RCTs. While 59/172 articles referred to interventions for no specific health condition, 72/172 interventions related to ‘cardiovascular, haematological, immunological and/or respiratory health’, 25/172 to neuromusculoskeletal health and 12/172 to ‘digestive, metabolic and endocrine health’. There were only two articles addressing mental health interventions, one addressing interventions for the senses and no articles in the areas of ‘end of life’, skin and related structures, genitourinary health, and or voice/speech.

The equality, diversity and inclusion characteristics, as assessed using PROGRESS-plus, of included populations were poorly reported, with only 10 of the 172 studies reporting on some aspect of the framework in the description of the sample characteristics.

#### Distribution by Setting and Location

**Figure 4.**
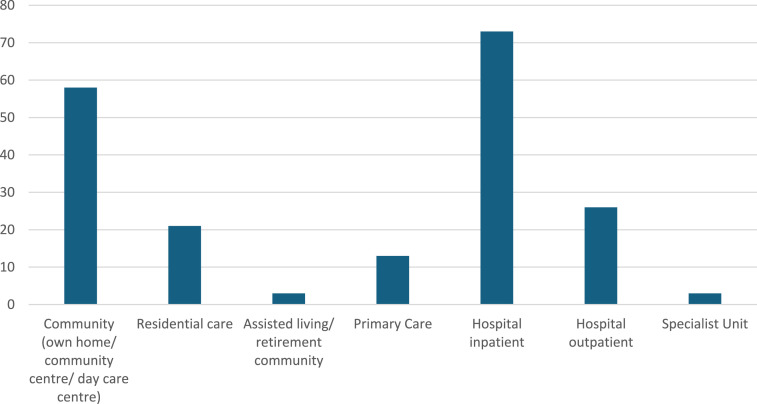
Distribution of intervention settings across studies within the map

The spread of settings across the interventions is displayed in Figure 4, with some interventions taking place across more than one setting. The two most common sites for interventions were the community, either in a person’s home or in day care centres, or within hospitals (as inpatients). Despite the focus of the EGM being the over 80s, only 11% of interventions were in residential care settings. The only pre-specified setting not reported amongst interventions was the hospice setting.

**Figure 5.**
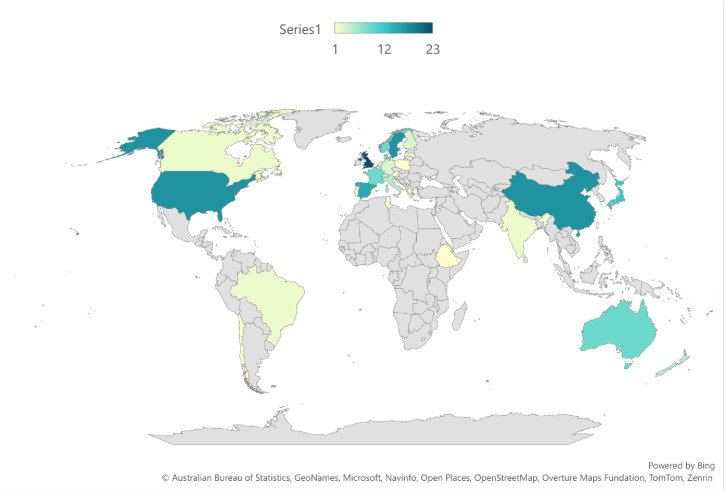
Distribution of countries featured in the map

Overall, 32 countries were represented in the map (see Figure 5). The evidence was largely focused amongst European populations, with the majority of systematic reviews (designated by lead author country), and RCTs coming from this region. After Europe (n = 81), the most common location for RCTs was Asia (n = 24), then Oceania (n = 12) and then the USA (n = 11). Of the sixteen primary qualitative studies, five were from Norway, three each from Sweden and the USA, two from the UK, and one each from China, Latvia and Finland.

#### Distribution by Interventions

**Figure 6.**
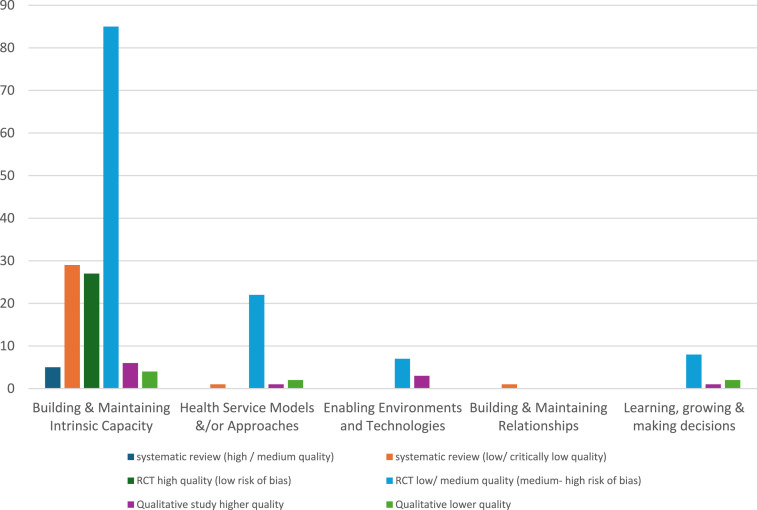
Distribution of studies across the five intervention domains, categorised by study design

The conceptual map underpinning our EGM related to intrinsic capacity, the environment and the interaction between the two. Interventions were therefore mapped across the five domains that encourage healthy ageing: ‘*building and maintaining intrinsic capacity’, ‘health services models and/or approaches’, ‘enabling environments and technologies’, ‘building and maintaining relationships’* and *‘learning, growing and making decisions’*. Figure 6 shows the distribution of studies across these broad intervention categories, organised by study design. All of the higher quality RCTs fell in the domain of *building and maintaining intrinsic capacity*.

It is clear to see that the evidence is not equally distributed across the intervention domains and in particular, that a large proportion of the evidence (157 articles, 91.2%), across all study designs had a focus, wholly or in part, on *building and maintaining intrinsic capacity*. The breakdown by sub-category and study design within this domain is shown in Table 2.

**Table 2.** Frequency of the Subcategories Within ‘Building and Maintaining Intrinsic Capacity’ Interventions According to Study Design

​	systematic review (high/medium quality)	systematic review (low/critically low quality)	RCT high quality (low risk of bias)	RCT low/medium quality (medium- high risk of bias)	Qualitative study higher quality	Qualitative lower quality
Surgical/Medical procedures	3	22	4	19	5	2
Medicines and medical technologies	1	6	22	25	1	1
Physical/Rehabilitation	1	1	1	37	0	0
Behavioural/Lifestyle	0	1	0	8	0	0
Oral/Nutrition	0	0	2	7	0	0
Psychological	0	0	0	0	0	0
Cognitive	0	0	0	4	0	0
Diagnostic	0	0	0	1	0	1

Within *building and maintaining intrinsic capacity*, systematic reviews were largely concerned with effectiveness of surgical interventions (n = 25/34, 73.5%), in cardiovascular (such as heart valve replacement or repair, coronary bypass and thrombectomy/percutaneous coronary intervention), neuromuscular (such as joint replacement or repair, and spinal/cranial surgery) and digestive areas (such as gastrectomy, and short or large bowel resection and/or removal). There were seven systematic reviews related to medicine interventions (Barssoum et al., 2021; Bhatnagar et al., 2011; Briand et al., 2022; Engelter et al., 2006; Lee et al., 2022; Marcellaud et al., 2023; Shantsila et al., 2023), predominantly on the effectiveness of anticoagulants and antihypertensives, and two systematic reviews with the focus related to physical/rehabilitation and/or behavioural interventions (Mende et al., 2022; Nicolson Philippa et al., 2021).

The RCT evidence within this category also was mainly spread across the same three sub-categories as the systematic reviews, but the relative weight of evidence differs: 47/113 (41.7%) trials were on assessing the effectiveness of medicines and as with the systematic reviews they were dominated by antihypertensives and anticoagulants, 38/113 (33.6%) trials were on physical/rehabilitation interventions (largely focused on resistance and strength/balance training), and 24/113 (21.2%) trials on surgical interventions, with a similar focus as per the systematic reviews. In addition, there were eight RCTs on behavioural/lifestyle interventions (largely physical activity interventions with or without a dietary component) (Barker et al., 2006; Campbell et al., 1999; Ehsani et al., 2003; Gene et al., 2018; Haanes et al., 2015; Luukinen et al., 2007; Oliveira et al., 2016; Puggaard et al., 2000), four on cognitive interventions (mainly brain training interventions) (Hilt Pauline et al., 2023; McCord et al., 2020; West, 2016; West et al., 2020) and one RCT on a diagnostic intervention (Burtin et al., 1995). This category also saw the largest amount of qualitative studies: 10/16 studies focused on experiences of cardiovascular, medicines and diagnostic interventions.

The second most prevalent intervention domain, represented in just over 20% of the studies, with one systematic review, 33 RCTs and four primary qualitative studies was *health service models and/or approaches.* This was predominantly RCTs assessing the effectiveness of home visits (either as the principal focus or as part of a multicomponent intervention) (Behm et al., 2014, 2016; Brettschneider et al., 2015; Campbell et al., 1997; Gene et al., 2018; Godwin et al., 2016; Gustafsson et al., 2012, 2013; Holland et al., 2005; Imhof et al., 2012; Lenaghan et al., 2007; Luck et al., 2013; Wilhelmson & Eklund, 2013; Zak & Gryglewska, 2006; Zidén et al., 2014) and/or comprehensive geriatric assessment (Brettschneider et al., 2015; Ferrer et al., 2014; Imhof et al., 2012; Luck et al., 2013; Vorilhon et al., 2016). Surprisingly, despite the number of RCTs in these sub-categories, there were no systematic reviews on these topics. The only systematic review within this intervention category was a review on the outcome of admitting very old adults with sepsis to intensive care units (Haas et al., 2017).

The two intervention domains of *enabling environments and technologies* and *learning, growing and making decisions*, contained no systematic reviews. This is despite the primary RCT evidence in several sub-categories. For example, there were eight RCTs that involved the use of information provision as part of a multi-component intervention (Behm et al., 2014, 2016; Gustafsson et al., 2012, 2013; Haanes et al., 2015; Luck et al., 2013; Wilhelmson & Eklund, 2013; Zidén et al., 2014), four RCTs that included home modification as part of an intervention (Gustafsson et al., 2012, 2013; Haanes et al., 2015; Zidén et al., 2014), four RCTs involving virtual reality/smart technology (Campo-Prieto et al., 2022; Gene et al., 2018; McCord et al., 2020; West, 2016), and three qualitative studies exploring the experience of personal mobility assistance (Kylberg et al., 2013; Lofqvist et al., 2009; Tomsone et al., 2016).

There is a dearth of published evidence for interventions relating to *building and maintaining relationships* in this age group. This intervention domain was set out to be broad, comprising the use of volunteers, animal-assisted interventions, intergenerational activities, peer and carer support interventions, and skills and craft activities. Only one systematic review represents this category in the map: a systematic review of purposeful activity (Owen et al., 2022).

#### Distribution by Outcomes

The most frequently reported outcomes (see Table 3), not surprisingly, since the majority of interventions focused on *building and or maintaining intrinsic category*, were physiological measures of health (either as events or markers of health), measures of functional health, chronic health or symptoms, and adverse events. In fact, together these categories of outcomes represent 69% of the outcome data.

**Table 3.** Distribution of Outcomes According to Intervention Domains

Outcomes	Building & maintaining intrinsic capacity	Health service models &/or approaches	Enabling environments and technologies	Building & maintaining relationships	Learning, growing & making decisions
Experiences	10	3	3	0	3
Physiological health	101	9	2	0	3
Physiological event	74	11	0	0	1
Functional health	51	11	5	0	6
Chronic health measure/symptoms	8	3	0	0	2
Medicines related	8	6	0	0	0
Psychological health & wellbeing	28	12	4	1	5
Cognitive health	15	1	2	0	0
Social health	0	0	0	0	0
Service/Resource use	25	11	0	0	0
Process	33	2	2	0	0
Adverse events	78	3	1	0	0

Psychological health and wellbeing, which included outcomes such as quality of life, loneliness, anxiety and depression, were represented in 7% of the outcomes. Other sub-categories with a low frequency (representing less than 5% of reported outcomes) in the map are medicine related measures (such as adherence, discontinuation) and measures of cognitive health (such as memory and measures of cognition).

Service resource outcomes, such as hospital admission, length of stay, primary care contact, and process related outcomes, such as acceptability, safety and adherence, accounted for 6% and 7% of the outcome data respectively. Social health (which included measures of connectedness and participation) was the only sub-category of outcomes that had no evidence: it did not feature as a reported outcome in any trial.

There were 16 studies that reported experiences of interventions. Nine focused solely on the experience of the older adult (Amofah et al., 2021; Behm et al., 2013; Bynum et al., 2014; Eriksen Kristina et al., 2021; Instenes et al., 2024; Kylberg et al., 2013; Lofqvist et al., 2009; Tomsone et al., 2016; Wising et al., 2022), and one each on the experience of carers (Ganske, 2002) and health professionals (Berkhout et al., 2022) and five from mixed perspectives (Burden et al., 2020; Chen et al., 2020; McCombie et al., 2021; Salter, 2007; Schonberg et al., 2006). The interventions fell predominantly in the *building capacity and maintaining intrinsic capacity* domain, relating to experiences of heart surgery and procedures, colon surgery, resuscitation, medicines review and preventative screening. There were three articles describing experience of preventive home visits (*health service and/or model interventions* domain) (Behm et al., 2013; Chen et al., 2020; Salter, 2007), three articles describing experiences of using and accessing mobility assistance devices (*enabling environments and technologies* domain) (Kylberg et al., 2013; Lofqvist et al., 2009; Tomsone et al., 2016) and two articles on the experience of decision-making (*leaning, growing and making decisions* domain) (Bynum et al., 2014; Schonberg et al., 2006).

The impact of interventions on equality, diversion and inclusion characteristics were poorly reported, with only nine articles reflecting this in their analyses: seven in relation to personal characteristics (two systematic reviews (DeBernardis Dennis et al., 2023; Merga et al., 2023) and five RCTs ((Bechshoft et al., 2017; Ehsani et al., 2003; Lopiz et al., 2019; Tegn et al., 2016; West, 2016), two with respect to education (West, 2016; Xu et al., 2022) and one systematic review each with respect to place of residence (Mende et al., 2022) and race/ethnicity (Merga et al., 2023).

#### Risk of Bias in Included Reviews

Table 4 shows the summary results of quality assessment of all the included studies in the EGM. The assessments for individual studies are provided in the Appendix (AMSTAR-2 overall assessment; Risk of bias for RCTs; and Wallace Criteria for qualitative studies).

**Table 4.** Quality Assessment of the Included Studies Within the Map

​	Risk of bias/Quality assessment	Number
AMSTAR 2	High/moderate confidence in results	5
	Low/critically low confidence in results	31
Cochrane ROB	Low risk of bias	27
	Medium/high risk of bias	93
Wallace criteria	High quality	10
	Lower quality	6

None of the reviews were assessed as having high overall level of confidence, and only five reviews were assessed as moderate, with the majority of reviews assessed as being low or critically low (31/36 (86%) reviews). There was no report of a protocol in over half of the reviews (21/36 (58%)), and just under half of the reviews did not incorporate a comprehensive search strategy. Whilst two thirds used satisfactory techniques to assess risk of bias in their included studies, just under a third of them (11/36 (31%)) adequately discussed the risk of bias in their findings. Only one review provided details of the excluded studies. Low and critically-low quality reviews may not provide an accurate and comprehensive summary of the available studies of interest.

The majority of RCTs had an overall summary assessment of being at moderate to high risk of bias (93/120). For these RCTs, in 80/93 (86%) risk of bias was assessed as high risk or unclear for ‘blinding of participants and personnel’, in 62/93 (67%) risk of bias was assessed as high or unclear for ‘blinding of outcome assessment’, and in 35/93 (38%) risk of bias was unclear for allocation concealment.

Ten of the 16 qualitative studies were assessed as being of higher quality, using the Wallace criteria and six of low-to-moderate quality. The main areas of concern were the lack of “explicit consideration of a theory or framework informing the research” and “consideration of how this influenced study design” (n = 11/16, 69%), insufficient description of data collection to ensure confidence in the findings (n = 7/16, 44%), insufficient details to ensure confidence that data analysis had been rigorous and the findings could be trusted (n = 7/16, 44%), and a lack of reflexivity from the authors reported in the paper (n = 12/16, 75%).

## Discussion

### Summary of Main Results

This EGM presents the volume of evidence of health and social care interventions for adults aged 80 years or more, including evidence of experiences of interventions. Overall, we found a bias towards interventions orientated around a biomedical view of health, centred on interventions for building and maintaining intrinsic capacity, and relatively little evidence on psychosocial and environmental aspects of health, or on adaptations to services/models of health and social care. There was also very little evidence on older people’s experiences of interventions.

The quality of the evidence was in general poor with the majority of systematic reviews assessed as low or critically low and the majority of RCTs at moderate or high risk of bias. Equity characteristics were poorly reported, both in terms of describing the study populations and when considering potential impact of interventions.

### Areas of Major Gaps in the Evidence

It was surprising to see several parts of the map where there was no evidence at all for this age group. In the domain of *health service models and/or approaches*, there were no studies assessing effectiveness of ‘hospital at home’, ‘telehealth’ or ‘mobile screening’. In the domain of *learning, growing and making decisions*, there were no qualitative or quantitative studies relating to end-of-life care or advance care planning. Similarly, in the domain of *building and maintaining relationships*, there were no RCTs or qualitative studies of interventions focused on person-centred or relational models of care: animal-assisted interventions, craft and/or skill interventions, intergenerational interventions, volunteer interventions, and peer/carer support interventions. This is matched in the map by the lack of social health outcomes measured across any intervention, such as connectedness and participation, and only one study reported on the outcome of loneliness.

Despite the majority of evidence falling within the domain of *building and maintaining intrinsic capacity*, there were areas within this that surprisingly lacked evidence. There were no studies, within the surgical and medical procedures, or medicines and technologies relating to osteoarthritis, chronic kidney disease, and urinary or faecal incontinence. These health conditions have been shown to rise with increasing age and are prevalent in approximately 30% or more of those aged 80 years or older (Melzer et al., 2015). There were also no studies relating to interventions for sensory impairments, despite the high prevalence in this age group: more than two thirds of adults over 80 years have been found to have one or more sensory impairments (Armstrong et al., 2022; Assi et al., 2020). Within the oral and nutrition category there were no studies relating to hydration, or dental health. Oral health problems, particularly tooth loss and dry mouth, in older adults have been associated with progression to frailty, and research is needed to ascertain if improving (or maintaining) oral health can help reduce the risk of frailty in older adults (Kimble et al., 2023).

There were also no interventions, or experiences of interventions, assessing psychological or cognitive therapies or on interventions to reduce loneliness and/or isolation. Research in ‘younger’ older adults, 65-80 years, has demonstrated beneficial effects of psychological and social-facilitation based interventions (Gardiner et al., 2018; Yu et al., 2023), yet studies are missing in those who might benefit the most. Social isolation and loneliness are risk factors for poor mental and physical health, and are particularly problematic for those aged 80 years of more as a result of reduced economic resources, fewer social networks, and changes in family structures (Courtin & Knapp, 2017). There were also few studies focused on this age group in the residential care setting.

Another purpose of EGMs is to identify future research opportunities, and where there are primary studies, in this case RCTs of the effectiveness of interventions, but no systematic review. There appears to be several opportunities for synthesis which could provide valuable summative evidence for informing health services, notably in the areas of polypharmacy, osteoporosis, hip surgery, and frailty prevention. There are also potential areas for syntheses in areas relating to the role/value of home-visits in healthcare for this age group, as well as information provision as part of broader health promotion initiatives.

### Potential Biases in the Mapping Process

We developed a framework based on the WHO model of healthy ageing (World Health Organisation, 2020) along with concepts the multidimensional model of healthy ageing proposed by Rivadeneira et al. (2021), which is also based on the central role of functional ability in healthy ageing. Other authors may have chosen different models to base the framework on, and within that, might have used different categories for interventions and outcomes.

Qualitative data was not categorized into themes. This may mean that there are data within these qualitative studies that could inform other outcome categories, such as quality of life, sleeping and adverse events.

### Limitations of the EGM

We used rigorous transparent review methods, including a comprehensive search strategy, duplicate screening of all the identified records and all data coding and assessments of the quality of included studies were checked by a second reviewer. Since our research question was about the effectiveness and/or experience of health and social care interventions, we included only systematic reviews, RCTs and primary qualitative studies. However, we did include all RCTs as primary studies irrespective of whether they were included within a systematic review so it may appear that there is more evidence within some domains than there really is. Conversely, we acknowledge that some of the systematic reviews contained non-randomised studies, which were not eligible on their own for inclusion in the map.

Although there were no language restrictions applied, we may have missed studies and reviews published in non-English language since mainly English databases were searched. We used only systematic review and qualitative search filters in Embase which could have led to missing relevant RCTs, but thorough searching of multiple databases hopefully mitigated this. We did not have the resource at the time to chase down hard to find full texts so we may have missed relevant studies. We have maintained a list of these articles, some of which may now have become available, for any future update. There was no standardized framework to guide the classification of interventions, but our framework was informed by both the literature on models of health ageing, and by topic experts and a public and patient engagement group (for details see ‘Stakeholder Engagement throughout the EGM process’).

The focus of the EGM was interventions specifically targeted at adults aged 80 years or more. We therefore searched for age and age-related terms but did not include search terms related to settings such as care homes, assisted living or residential care. There is a possibility that there are studies that have been undertaken within these settings that include populations with a minimum age of 80 years by chance, but if age itself was not the focus of the study they would not have been eligible for this review. If ‘age’ had been the focus of the study, we believe they would have been picked up by the database searches and supplementary searches. We acknowledge that studies focused on the ‘higher range’ of older adults using lower age cut-offs, such as 70 or 75 years, which may have included substantial proportion of adults aged over 80 are not included in this map. This was not the purpose of our map, we were only interested in adults aged over 80 years. As has been shown, the age of 80 years marks the inflection point of rapid decline across various indicators of cardiorespiratory and physical health (Ngcobo, 2025).

We are cognisant that this review is bounded by the study designs that we considered eligible for inclusion in the map. By restricting quantitative studies to systematic reviews and RCTs, we were hoping to map the best available evidence in relation to effectiveness of interventions. Clearly broadening the study designs to broader observational or non-randomised studies might have altered the balance of intervention domains covered by the EGM.

### Stakeholder Engagement Throughout the EGM Process

Feedback, and actions where appropriate, relating to stakeholder and PenPEG goup engagement throughout the review process are detailed below in Table 5.

**Table 5.** Stakeholder engagement Through the Process of the EGM

Stage of review	Group	Comment	Action
Protocol	PenPEG	Dislike of the term ‘oldest old’	Review name changed to ‘Over 80 EGM’, and oldest old term not used as a descriptive term for the main manuscript
Protocol	External topic experts	Clarification needed around intervention categories	Revised intervention categories & subcategories
First draft of EGM - general	PenPEG	Overall impressed with the EGM.	NR
First draft of EGM - gaps	PenPEG	Surprised at how little research there was on assistive technologies – strong suggestion that future trials could look at this including AI. Also surprised at how little there was on mental health outcomes, and nothing on social isolation.	Added to discussion.
First draft of EGM – other thoughts	PenPEG	Older people experience social isolation, it’s not just about gadgets and gizmos – links back to mental health. On what older people actually want for themselves. Need to be aware as population of >80 yrs grows, ideas of what an 80 yr old will be capable of and want will change. Stays in hospitals too long now there are no cottage hospitals – imp for those >80 yrs and needs addressing.	NR
First draft of EGM - general	External topic expert (rehab)	“Almost all evidence is biomedical, even within rehabilitation – I thought we were progressing away from this, but it appears not!”	Already noted in discussion and conclusion
First draft of EGM - gaps	External topic expert (rehab)	Absence of interventions targeting functional decline due to sensory impairment. No intervention studies on hydration or oral health – “so important in holistic care”. No RCTs on interventions to support carers of over-80s – “carer burden is so high and clinically we spend a lot of time supporting/managing carers, I’m surprised there’s not a focus on this”	Dearth of sensory impairment and oral health/hydration interventions already noted. Lack of carer support intervention added to results
Plain language summary	PenPEG and convenience sample of public (n = 4, aged 60-90 yrs)	Clarification of terms such as adverse events, psychosocial	Amended where needed

## Authors’ Conclusions

As the worldwide population continues to age, it is increasingly important that we have evidence of appropriate effective interventions for those who have reached their 80s, 90s and beyond, a group often left out of trials. This evidence and gap map shows that there is an under-representation of studies that focus specifically on those at the higher end of older adults, those aged 80 years or more, across the broad spectrum of health and social care interventions. There is a clear bias towards interventions orientated around a biomedical view of health, focusing on intrinsic capacity and basic needs, and relatively little on the wider functional and psychosocial aspect of health, or on enabling environments, such as adaptations to health and care services, or adapted models of care. There is also a clear need for more research to understand the experiences and preferences of interventions from adults aged 80 years or more, and for more research in general for this age group beyond the hospital, within the home and community setting.

### Implications for Research, Practice And/or Policy

#### Implications for Research

Challenges in delivering health and social care services alongside the ageing population globally means there should be greater focus on keeping populations healthy, with individuals growing older independently in their own home. Evidence of interventions to inform this, from those aged 80 years or more, is clearly lacking. Research findings drawn from younger age groups might not be applicable for this age group. Treatments may be less effective and the care being offered might not be appropriately tailored. The absence of evidence may also mean that those aged over 80 may not be offered certain treatments.

This map of existing interventions targeting those aged 80 years or more, shows that in terms of research, there is still a clear need for:

• A more inclusive approach both (i) broadly and (ii) in specific target areas (such as osteoporosis, end-of-life care, sensory impairments);
• A greater understanding of how interventions may impact different members of this age group differently (equity);
• A greater focus on evaluating the experience of those aged 80 years or more, and their carers, on interventions they have received;
• An understanding from those aged 80 years or more, their carers and relevant health professionals, of which interventions researchers should be focusing on – this might be quite different from the research they are invited to be part of.

Whilst best practice recommendations to promote the inclusion of older people in research have been developed (Goodwin et al., 2023), and include principles such as focusing on engagement, inclusive study design, adequate resources and communication, research on how best to implement them is lacking. This is particularly relevant for those who are not specialists in conducting research with older people. In addition, adopting better practices with regards to using PROGRESS plus criteria when reporting results of studies needs to become standard practice amongst researchers.

#### Implications for Practice and/or Policy

Transformations in healthcare systems are happening worldwide (Hunter & Bengoa, 2023). Focusing on what matters most to patients and the community, being person-centred, investing in wellness and utilising digital innovation is seen across health strategies (Ministerio de Sanidad, 2025; NSW Health, 2023). In the UK, there has been a call for a shift in healthcare: from hospital to community, from analogue to digital, and from sickness to prevention (UK Government, 2025). Our EGM shows that there is little evidence that has implicitly included, or drawn on, the experiences of those in their 80s or 90s. How best to implement healthcare in the community for this growing subset of the population, and whether, or indeed if, digital healthcare would work for this growing subset is unknown.

The recent joint statement of intent relating to integrating older age into health and care research from NIHR, 2025 and other funding bodies (NIHR, 2025) recognises that older adults are disproportionally underrepresented in research and calls for a commitment to actively support the inclusion of older adults in research, particularly where health conditions have a particular impact on this group. Whilst details with regards to inclusion are now a requirement of many funding applications, it will be paramount that funding bodies bear this statement in mind when deciding on whether inclusion in relation to age, has been fully taken into consideration.

## Supplemental Material

Supplemental Material - Health and Social Care Interventions in the 80 years Old and Over Population: An Evidence and Gap Map Supplemental Material for Health and Social Care Interventions in the 80 years Old and Over Population: An Evidence and Gap Map by Rebecca Abbott, Alison Bethel, Jo Thompson Coon, Morwenna Rogers, Rebecca Whear, Noreen Orr, Ruth Garside, Victoria A. Goodwin, Aseel Mahmoud, Ilianna Lourida, Debbie Cheeseman in Campbell Systematic Reviews.
