# Supplementary material for: Health and Social Care Interventions in the 80 years Old and Over Population: An Evidence and Gap Map
Source: Campbell Syst Rev. 2026 Jun 24;22(2):18911803261462104. doi: 10.1177/18911803261462104 (PMC13305380; doi:10.1177/18911803261462104)
Supplement: Supplemental Material - Health and Social Care Interventions in the 80 years Old and Over Population: An Evidence and Gap Map [file sj-pdf-1-cam-10.1177_18911803261462104.pdf]

APPENDIX

1. Search strategies (page 2)
2. Original search summary table (page 24)
3. Final search summary table (page 32)
4. List of included studies (page 40)
5. Table of excluded studies (page 54)
6. Quality Appraisal of Systematic Reviews: Amstar-2 (page 112)
7. Risk of Bias table for RCTs: ROB1 (page 117)
8. Quality Appraisal for qualitative studies: Wallace Criteria (page 125)

Appendix (1) Search Strategies

Ageline searches

Ageline update July 2024. Edits in red

#	Query	Limiters/Expanders	Last Run Via	Results
S16	S8 AND S15	Expanders - Apply equivalent subjects<br>Search modes - Boolean/Phrase	Interface - EBSCOhost Research Databases<br>Search Screen - Advanced Search<br>Database - AgeLine	1,872
S15	S9 OR S10 OR S11 OR S12 OR S13 OR S14	Expanders - Apply equivalent subjects<br>Search modes - Boolean/Phrase	Interface - EBSCOhost Research Databases<br>Search Screen - Advanced Search<br>Database - AgeLine	4,426
S14	(ZU geriatric assessment)	Expanders - Apply equivalent subjects<br>Search modes - Boolean/Phrase	Interface - EBSCOhost Research Databases<br>Search Screen - Advanced Search<br>Database - AgeLine	603
S13	(ZU "oldest old") or (ZU "oldest-old")	Expanders - Apply equivalent subjects<br>Search modes - Boolean/Phrase	Interface - EBSCOhost Research Databases<br>Search Screen - Advanced Search<br>Database - AgeLine	183
S12	AB (oldest and (people* or person* or adult* or old or olds or elderly)).	Expanders - Apply equivalent subjects<br>Search modes - Boolean/Phrase	Interface - EBSCOhost Research Databases<br>Search Screen - Advanced Search<br>Database - AgeLine	1,729
S11	TI ("over 85" or "over 85s") or AB ("over 85" or "over 85s") or TI ("over 90" or "over 90s") or AB ("over 90" or "over 90s") or TI ("over 80" or "over 80s") or AB ("over 80" or "over 80s")	Expanders - Apply equivalent subjects<br>Search modes - Boolean/Phrase	Interface - EBSCOhost Research Databases<br>Search Screen - Advanced Search<br>Database - AgeLine	497
S10	TI "very old*" OR AB "very old*" OR AB centenarian* OR TI	Expanders - Apply equivalent subjects	Interface - EBSCOhost Research Databases<br>Search Screen - Advanced Search	1,682

	centenarian* OR AB nonagenarian* OR TI nonagenarian* OR TI octogenarian* OR AB octogenarian*	Search modes - Boolean/Phrase	Database - AgeLine	
S9	TI oldest	Expanders - Apply equivalent subjects<br>Search modes - Boolean/Phrase	Interface - EBSCOhost Research Databases<br>Search Screen - Advanced Search<br>Database - AgeLine	523
S8	S1 OR S2 OR S3 OR S4 OR S5 OR S6 OR S7	Expanders - Apply equivalent subjects<br>Search modes - Boolean/Phrase	Interface - EBSCOhost Research Databases<br>Search Screen - Advanced Search<br>Database - AgeLine	86,863
S7	(ZU "randomised controlled trial") or (ZU "randomised controlled trials") or (ZU "randomized clinical trial") or (ZU "randomized controlled trial")	Expanders - Apply equivalent subjects<br>Search modes - Boolean/Phrase	Interface - EBSCOhost Research Databases<br>Search Screen - Advanced Search<br>Database - AgeLine	203
S6	(ZU "meta-analysis")	Expanders - Apply equivalent subjects<br>Search modes - Boolean/Phrase	Interface - EBSCOhost Research Databases<br>Search Screen - Advanced Search<br>Database - AgeLine	418
S5	(ZU "systematic review") or (ZU "systematic reviews")	Expanders - Apply equivalent subjects<br>Search modes - Boolean/Phrase	Interface - EBSCOhost Research Databases<br>Search Screen - Advanced Search<br>Database - AgeLine	1,745
S4	(ZU "qualitative")	Expanders - Apply equivalent subjects<br>Search modes - Boolean/Phrase	Interface - EBSCOhost Research Databases<br>Search Screen - Advanced Search<br>Database - AgeLine	373
S3	AB (trial* or random*) OR TI (trial* or random*)	Expanders - Apply equivalent subjects<br>Search modes - Boolean/Phrase	Interface - EBSCOhost Research Databases<br>Search Screen - Advanced Search<br>Database - AgeLine	14,269
S2	AB (qualitativ* or interview* or experienc*) OR TI (qualitativ* or interview* or experienc*)	Expanders - Apply equivalent subjects<br>Search modes - Boolean/Phrase	Interface - EBSCOhost Research Databases<br>Search Screen - Advanced Search<br>Database - AgeLine	48,602

S1	AB (synthes* or review* or overview* or evidence* or "meta analysis" or metaanalysis) OR TI (synthes* or review* or overview* or evidence* "meta analysis" or metaanalysis)	Expanders - Apply equivalent subjects<br>Search modes - Boolean/Phrase	Interface - EBSCOhost Research Databases<br>Search Screen - Advanced Search<br>Database - AgeLine	37,854

#	Query	Limiters/Expanders	Last Run Via	Results
S15	S8 AND S14	Expanders - Apply equivalent subjects<br>Search modes - Boolean/Phrase	Interface - EBSCOhost Research Databases<br>Search Screen - Advanced Search<br>Database - AgeLine	1,508
S14	S9 OR S10 OR S11 OR S12 OR S13	Expanders - Apply equivalent subjects<br>Search modes - Boolean/Phrase	Interface - EBSCOhost Research Databases<br>Search Screen - Advanced Search<br>Database - AgeLine	3,705
S13	(ZU "oldest old") or (ZU "oldest-old")	Expanders - Apply equivalent subjects<br>Search modes - Boolean/Phrase	Interface - EBSCOhost Research Databases<br>Search Screen - Advanced Search<br>Database - AgeLine	171
S12	AB (oldest and (people* or person* or adult* or old or olds or elderly)).	Expanders - Apply equivalent subjects<br>Search modes - Boolean/Phrase	Interface - EBSCOhost Research Databases<br>Search Screen - Advanced Search<br>Database - AgeLine	1,674
S11	TI ("over 85" or "over 85s") or AB ("over 85" or "over 85s") or TI ("over 90" or "over 90s") or AB ("over 90" or "over 90s") or TI ("over 80" or "over 80s") or AB ("over 80" or "over 80s")	Expanders - Apply equivalent subjects<br>Search modes - Boolean/Phrase	Interface - EBSCOhost Research Databases<br>Search Screen - Advanced Search<br>Database - AgeLine	479
S10	TI "very old*" OR AB "very old*" OR AB centenarian* OR TI centenarian* OR AB	Expanders - Apply equivalent subjects<br>Search modes - Boolean/Phrase	Interface - EBSCOhost Research Databases<br>Search Screen - Advanced Search<br>Database - AgeLine	1,626

	nonagenarian* OR TI nonagenarian* OR TI octogenarian* OR AB octogenarian*			
S9	TI oldest	Expanders - Apply equivalent subjects<br>Search modes - Boolean/Phrase	Interface - EBSCOhost Research Databases<br>Search Screen - Advanced Search<br>Database - AgeLine	497
S8	S1 OR S2 OR S3 OR S4 OR S5 OR S6 OR S7	Expanders - Apply equivalent subjects<br>Search modes - Boolean/Phrase	Interface - EBSCOhost Research Databases<br>Search Screen - Advanced Search<br>Database - AgeLine	82,992
S7	(ZU "randomised controlled trial") or (ZU "randomised controlled trials") or (ZU "randomized clinical trial") or (ZU "randomized controlled trial")	Expanders - Apply equivalent subjects<br>Search modes - Boolean/Phrase	Interface - EBSCOhost Research Databases<br>Search Screen - Advanced Search<br>Database - AgeLine	186
S6	(ZU "meta-analysis")	Expanders - Apply equivalent subjects<br>Search modes - Boolean/Phrase	Interface - EBSCOhost Research Databases<br>Search Screen - Advanced Search<br>Database - AgeLine	343
S5	(ZU "systematic review") or (ZU "systematic reviews")	Expanders - Apply equivalent subjects<br>Search modes - Boolean/Phrase	Interface - EBSCOhost Research Databases<br>Search Screen - Advanced Search<br>Database - AgeLine	1,128
S4	(ZU "qualitative")	Expanders - Apply equivalent subjects<br>Search modes - Boolean/Phrase	Interface - EBSCOhost Research Databases<br>Search Screen - Advanced Search<br>Database - AgeLine	340
S3	AB (trial* or random*) OR TI (trial* or random*)	Expanders - Apply equivalent subjects<br>Search modes - Boolean/Phrase	Interface - EBSCOhost Research Databases<br>Search Screen - Advanced Search<br>Database - AgeLine	13,401
S2	AB (qualitativ* or interview* or experienc*) OR TI (qualitativ* or interview* or experienc*)	Expanders - Apply equivalent subjects<br>Search modes - Boolean/Phrase	Interface - EBSCOhost Research Databases<br>Search Screen - Advanced Search<br>Database - AgeLine	46,597

S1	AB (synthes* or review* or overview* or evidence* or "meta analysis" or metaanalysis) OR TI (synthes* or review* or overview* or evidence* "meta analysis" or metaanalysis)	Expanders - Apply equivalent subjects<br>Search modes - Boolean/Phrase	Interface - EBSCOhost Research Databases<br>Search Screen - Advanced Search<br>Database - AgeLine	36,065

2b ASSIA search

2022 and 2024 same search strategy

(((title("oldest") OR (abstract("oldest") and abstract("people*" or "person*" or "adult*" or "old" or "elderly")) OR (title("octogenarian*" OR "centenarian*" OR "nonogenarian*") OR abstract("octogenarian*" OR "centenarian*" OR "nonogenarian*")) OR title("over 85" or "over 85s" or "over 90" or "over 90s" or "over 80" or "over 80s") OR (abstract("over 85" or "over 85s" or "over 90" or "over 90s" or "over 80" or "over 80s") AND abstract("year*" or "age*"))) AND ((TI(synthes* or review* or overview*) OR AB(synthes* or review* or overview*)) OR MAINSUBJECT.EXACT("Systematic reviews"))) OR ((title("oldest") OR (abstract("oldest") and abstract("people*" or "person*" or "adult*" or "old" or "elderly")) OR (title("octogenarian*" OR "centenarian*" OR "nonogenarian*") OR abstract("octogenarian*" OR "centenarian*" OR "nonogenarian*")) OR title("over 85" or "over 85s" or "over 90" or "over 90s" or "over 80" or "over 80s") OR (abstract("over 85" or "over 85s" or "over 90" or "over 90s" or "over 80" or "over 80s") AND abstract("year*" or "age*"))) AND (MAINSUBJECT.EXACT("Randomized controlled trials") OR AB(random* NEAR/2 control* NEAR/2 trial*) OR TI(random* NEAR/2 control* NEAR/2 trial*)))) OR ((title("oldest") OR (abstract("oldest") and abstract("people*" or "person*" or "adult*" or "old" or "elderly")) OR (title("octogenarian*" OR "centenarian*" OR "nonogenarian*") OR abstract("octogenarian*" OR "centenarian*" OR "nonogenarian*")) OR title("over 85" or "over 85s" or "over 90" or "over 90s" or "over 80" or "over 80s") OR (abstract("over 85" or "over 85s" or "over 90" or "over 90s" or "over 80" or "over 80s") AND abstract("year*" or "age*"))) AND (title("qualitativ*" or "interview*" or "experienc*") OR abstract("qualitativ*" or "interview*" or "experienc*") OR MAINSUBJECT.EXACT("Qualitative research")))

2c CDSR and Central

Update (edited) : July 2024. Edits in red

#1 (oldest and (people* or person* or adult* or old or elderly)):ti 48

#2 (oldest NEAR/2 (people* or person* or adult* or old or elderly)):ab 86

#3 "very old*":ti,ab 236

#4 centenarian*:ti,ab 16

#5 nonagenarian*:ti,ab 33

#6 octogenarian*:ti,ab 117

#7 #1 or #2 or #3 or #4 or #5 or #6 482

#8 meSH descriptor: [Aged] this term only 255357

#9 MeSH descriptor: [Aged, 80 and over] this term only 62464

#10 MeSH descriptor: [Frail elderly] this term only 1025

#11 #9 or #10

#12 #11 NOT #8 951

#13 #7 or #12 1406

Oct 2023. Original search

ID Search Hits

#1 (oldest and (people* or person* or adult* or old or elderly)):ti 48

#2 (oldest NEAR/2 (people* or person* or adult* or old or elderly)):ab 86

#3 "very old*":ti,ab 236

#4 centenarian*:ti,ab 16

#5 nonagenarian*:ti,ab 33

#6 octogenarian*:ti,ab 117

#7 #1 or #2 or #3 or #4 or #5 or #6 482

2d CINAHL

2022 and 2024 search strategy the same

Tuesday, October 25, 2022 12:56:05 PM

#	Query	Limiters/Expanders	Last Run Via	Results
S29	S24 OR S28	Expanders - Apply equivalent subjects<br>Search modes - Boolean/Phrase	Interface - EBSCOhost Research Databases<br>Search Screen - Basic Search<br>Database - CINAHL Complete	2,002
S28	S18 AND S27	Expanders - Apply equivalent subjects<br>Search modes - Boolean/Phrase	Interface - EBSCOhost Research Databases<br>Search Screen - Basic Search<br>Database - CINAHL Complete	903
S27	S25 OR S26	Expanders - Apply equivalent subjects<br>Search modes - Boolean/Phrase	Interface - EBSCOhost Research Databases<br>Search Screen - Basic Search<br>Database - CINAHL Complete	1,121,333
S26	(MH "Systematic Review")	Expanders - Apply equivalent subjects<br>Search modes - Boolean/Phrase	Interface - EBSCOhost Research Databases<br>Search Screen - Basic Search<br>Database - CINAHL Complete	114,875
S25	(TI synthes* or TI review* or TI overview* or AB evidence* or AB synthes* or AB review* or AB overview* or AB evidence*)	Expanders - Apply equivalent subjects<br>Search modes - Boolean/Phrase	Interface - EBSCOhost Research Databases<br>Search Screen - Basic Search<br>Database - CINAHL Complete	1,107,636
S24	S19 OR S23	Expanders - Apply equivalent subjects<br>Search modes - Boolean/Phrase	Interface - EBSCOhost Research Databases<br>Search Screen - Advanced Search<br>Database - CINAHL Complete	1,358
S23	S18 AND S22	Expanders - Apply equivalent subjects<br>Search modes - Boolean/Phrase	Interface - EBSCOhost Research Databases<br>Search Screen - Advanced Search<br>Database - CINAHL Complete	969
S22	S20 OR S21	Expanders - Apply equivalent subjects<br>Search modes - Boolean/Phrase	Interface - EBSCOhost Research Databases<br>Search Screen - Advanced Search<br>Database - CINAHL Complete	796,244

S21	(MH "Qualitative Studies+")	Expanders - Apply equivalent subjects<br>Search modes - Boolean/Phrase	Interface - EBSCOhost Research Databases<br>Search Screen - Advanced Search<br>Database - CINAHL Complete	174,407
S20	(TI qualitative* OR AB qualitative* OR TI interview* OR AB Interview* OR TI experienc* OR AB experienc*)	Expanders - Apply equivalent subjects<br>Search modes - Boolean/Phrase	Interface - EBSCOhost Research Databases<br>Search Screen - Advanced Search<br>Database - CINAHL Complete	754,702
S19	S16 AND S18	Expanders - Apply equivalent subjects<br>Search modes - Boolean/Phrase	Interface - EBSCOhost Research Databases<br>Search Screen - Advanced Search<br>Database - CINAHL Complete	449
S18	S1 OR S2 OR S3 OR S4 OR S5 OR S6 OR S7 OR S8 OR S9 OR S10	Expanders - Apply equivalent subjects<br>Search modes - Boolean/Phrase	Interface - EBSCOhost Research Databases<br>Search Screen - Advanced Search<br>Database - CINAHL Complete	6,393
S17	S12 AND S16	Expanders - Apply equivalent subjects<br>Search modes - Boolean/Phrase	Interface - EBSCOhost Research Databases<br>Search Screen - Advanced Search<br>Database - CINAHL Complete	43,681
S16	S13 OR S14 OR S15	Expanders - Apply equivalent subjects<br>Search modes - Boolean/Phrase	Interface - EBSCOhost Research Databases<br>Search Screen - Advanced Search<br>Database - CINAHL Complete	659,693
S15	(MH "Randomized Controlled Trials+")	Expanders - Apply equivalent subjects<br>Search modes - Boolean/Phrase	Interface - EBSCOhost Research Databases<br>Search Screen - Advanced Search<br>Database - CINAHL Complete	134,193
S14	(AB placebo* or trial* or randomly)	Expanders - Apply equivalent subjects<br>Search modes - Boolean/Phrase	Interface - EBSCOhost Research Databases<br>Search Screen - Advanced Search<br>Database - CINAHL Complete	630,378
S13	(AB randomi?ed OR TI randomi?ed)	Expanders - Apply equivalent subjects<br>Search modes - Boolean/Phrase	Interface - EBSCOhost Research Databases<br>Search Screen - Advanced Search<br>Database - CINAHL Complete	297,393
S12	S1 OR S2 OR S3 OR S4	Expanders - Apply equivalent subjects	Interface - EBSCOhost Research Databases	330,714

	OR S5 OR S6 OR S7 OR S8 OR S9 OR S10 OR S11	Search modes - Boolean/Phrase	Search Screen - Advanced Search<br>Database - CINAHL Complete	
S11	(MH "Aged, 80 and Over")	Expanders - Apply equivalent subjects<br>Search modes - Boolean/Phrase	Interface - EBSCOhost Research Databases<br>Search Screen - Advanced Search<br>Database - CINAHL Complete	328,563
S10	(MM "Centenarians")	Expanders - Apply equivalent subjects<br>Search modes - Boolean/Phrase	Interface - EBSCOhost Research Databases<br>Search Screen - Advanced Search<br>Database - CINAHL Complete	77
S9	(MM "Octogenarians")	Expanders - Apply equivalent subjects<br>Search modes - Boolean/Phrase	Interface - EBSCOhost Research Databases<br>Search Screen - Advanced Search<br>Database - CINAHL Complete	46
S8	(MM "Nonagenarians")	Expanders - Apply equivalent subjects<br>Search modes - Boolean/Phrase	Interface - EBSCOhost Research Databases<br>Search Screen - Advanced Search<br>Database - CINAHL Complete	51
S7	((TI oldest) AND ((TI people*) OR (TI person*) OR (TI adult*) OR (TI old) OR (TI elderly))	Expanders - Apply equivalent subjects<br>Search modes - Boolean/Phrase	Interface - EBSCOhost Research Databases<br>Search Screen - Advanced Search<br>Database - CINAHL Complete	975
S6	((AB oldest) N4 ((AB people*) OR (AB person*) OR (AB adult*) OR (AB old) OR (AB elderly)))	Expanders - Apply equivalent subjects<br>Search modes - Boolean/Phrase	Interface - EBSCOhost Research Databases<br>Search Screen - Advanced Search<br>Database - CINAHL Complete	1,589
S5	(TI "very old*" OR AB "very old*")	Expanders - Apply equivalent subjects<br>Search modes - Boolean/Phrase	Interface - EBSCOhost Research Databases<br>Search Screen - Advanced Search<br>Database - CINAHL Complete	1,647
S4	(TI centenarian* OR AB centenarian*)	Expanders - Apply equivalent subjects<br>Search modes - Boolean/Phrase	Interface - EBSCOhost Research Databases<br>Search Screen - Advanced Search<br>Database - CINAHL Complete	805
S3	(TI nonagenarian* OR AB nonagenarian*)	Expanders - Apply equivalent subjects<br>Search modes - Boolean/Phrase	Interface - EBSCOhost Research Databases<br>Search Screen - Advanced Search	668

			Database - CINAHL Complete	
S2	(TI octogenarian* OR AB octogenarian*)	Expanders - Apply equivalent subjects<br>Search modes - Boolean/Phrase	Interface - EBSCOhost Research Databases<br>Search Screen - Advanced Search<br>Database - CINAHL Complete	1,190
S1	(((TI year* OR AB year*) OR (TI age* OR AB age*)) N3 ((TI "over 85" OR AB "over 85") OR (TI "over 85s" OR AB "over 85s") OR (TI "over 90" OR AB "over 90") OR (TI "over 90s" OR AB "over 90s") OR (TI "over 80" OR AB "over 80") OR (TI "over 80s" OR AB "over 80s")))	Expanders - Apply equivalent subjects<br>Search modes - Boolean/Phrase	Interface - EBSCOhost Research Databases<br>Search Screen - Advanced Search<br>Database - CINAHL Complete	841

2e Embase

2022 and 2024 search strategies the same

1 very elderly/ (271161)

2 old*.tw. (2419585)

3 1 and 2 (73828)

NOTE: Emtree term has so many hits that added in a text word (might have somethin to do with automatic indexing as all Embase refs only indexed automatically unless drug, disease or device refs)

4 (oldest and (people* or person* or adult* or old or elderly)).ti. (1864)

5 (oldest adj2 (people* or person* or adult* or old or elderly)).ab. (3732)

6 "very old*".tw. (6519)

7 centenarian*.tw. (2723)

8 nonagenarian*.tw. (2331)

9 octogenarian*.tw. (6007)

10 (over 85 or over 85s).tw. (4151)

11 (over 90 or over 90s).tw. (23896)

12 (over 80 or over 80s).tw. (20113)

13 (year* or age*).tw. (9484285)

14 10 or 11 or 12 (47588)

15 ((year* or age*) adj2 (over 85 or over 85s or over 90 or over 90s or over 80 or over 80s)).tw. (6008)

16 3 or 4 or 5 or 6 or 7 or 8 or 9 or 15 (92237)

17 exp meta analysis/ or "systematic review"/ (494385)

18 ((systematic* or systematized or integrative or mapping or rapid or scoping) adj3 (review* or overview*)).tw. (369313)

19 ((evidence or interpretive or meta or quantitative or qualitative or integrative or collaborative or mixed method*) adj3 (synthes* or review* or overview*)).tw. (238408)

20 ("meta-analys?s" or metaanalys?s or metasynth*).tw. (313311)

21 (cost* adj3 review*).tw. (5375)

22 (data adj extraction).ab. (35978)

23 (narrative adj (review* or synthes?s)).tw. (28935)

24 (cochrane or evidence report).jw. (24023)

25 17 or 18 or 19 or 20 or 21 or 22 or 23 or 24 (738465)

26 (("semi-structured" or semistructured or unstructured or informal or "in-depth" or indepth or "face-to-face" or structured or guide) adj3 (interview* or discussion* or questionnaire*)).ti,ab. (208150)

27 (focus group* or qualitative or ethnograph*).tw,kw. (412280)

28 qualitative research/ (105967)

29 26 or 27 or 28 (552399)*1

30 16 and 29 (3655)

31 16 and 25 (1983)

32 30 or 31 (5498)

*1 used the CHLA search lines: https://extranet.santecom.qc.ca/wiki/!biblio3s/doku.php?id=concepts:recherche-qualitative

Search restricted to systematic reviews and qualitative studies

2f Epistemonikos

2022 and 2024 search strategies the same

(title:(oldest old) OR abstract:(oldest old))	55	64
simple search: oldest	24	29
(title:(centenarian) OR abstract:(centenarian))	7	7 (not exported)
(title:(octogenarian) OR abstract:(octogenarian))	22	24
(title:(nonagenarian) OR abstract:(nonagenarian))	11	12
Simple search: octogenarian	56	62
Simple search: nonagenarian	18	21
Simple search: centenarian	10	15
(title:("very old") OR abstract:("very old"))	71	77

2g HMIC

Update search 2024. Edits in red

1	oldest.ti.	20
2	very old*.tw.	95
3	(oldest and (people* or person* or adult* or old or **women or men** or elderly)).ab.	151
4	centenarian*.tw.	17
5	nonagenarian*.tw.	3
6	octogenarian*.tw.	8
7	(over 85 or over 85s).tw.	72
8	(over 90 or over 90s).tw.	177
9	(over 80 or over 80s).tw.	239
10	(year* or age*).tw.	78619
11	7 or 8 or 9	478
12	10 and 11	242
13	1 or 2 or 3 or 4 or 5 or 6 or 12	503
14	**(frail adj2 old*).tw.**	270
15	centenarians/ or frail elderly people/ or frail older people/ or over 80s/	406
16	13 or 14 or 15	1016

Original search 2022

1 oldest.ti. (20)
2 (oldest and (people* or person* or adult* or old or elderly)).ab. (136)
3 very old*.tw. (95)
4 centenarian*.tw. (16)
5 nonagenarian*.tw. (3)
6 octogenarian*.tw. (8)
7 (over 85 or over 85s).tw. (72)
8 (over 90 or over 90s).tw. (177)
9 (over 80 or over 80s).tw. (235)
10 (year* or age*).tw. (77748)
11 7 or 8 or 9 (474)
12 ((year* or age*) adj5 (over 85 or over 85s or (over 90 or over 90s) or (over 80 or over 80s))).tw. (77)
13 1 or 2 or 3 or 4 or 5 or 6 or 12 (325)
14 centenarians/ or frail elderly people/ or frail older people/ or over 80s/ (395)
15 13 or 14 (687)

NOTE: Kept broader to try and capture additional refs as HMIC is a much smaller database

2h Medline

Ovid MEDLINE(R) Update search 2024. Edits in red

1	*"aged, 80 and over"/ or centenarians/ or nonagenarians/ or octogenarians/	2080
2	(oldest and (people* or person* or adult* or old or elderly)).ti.	1734
3	(oldest adj2 (people* or person* or adult* or old or elderly)).ab.	3325
4	"very old*".tw.	5241
5	centenarian*.tw.	2665
6	nonagenarian*.tw.	1961
7	octogenarian*.tw.	4430
8	(over 85 or over 85s).tw.	3429
9	(over 90 or over 90s).tw.	20480
10	(over 80 or over 80s).tw.	16534
11	(year* or age*).tw.	7902398
12	((year* or age*) adj2 (over 85 or over 85s or over 90 or over 90s or over 80 or over 80s)).tw.	4344
13	1 or 2 or 3 or 4 or 5 or 6 or 7 or 12	20987
14	*Geriatric Assessment/	15671
15	*Frail Elderly/	9681
16	geriatric assessment*.tw.	6278
17	14 or 15 or 16	27291
18	((older or old) adj2 (people* or person* or adult* or men or women)).tw.	263092
19	17 and 18	11056
20	13 or 19	31713
21	randomized controlled trial.pt.	644410
22	controlled clinical trial.pt.	95728
23	randomi#ed.ab.	840235
24	placebo$.ab.	262132
25	drug therapy.fs.	2844726
26	randomly.ab.	467018
27	trial.ab.	770059
28	groups.ab.	2901569
29	exp animals/ not humans.sh.	5368625
30	21 or 22 or 23 or 24 or 25 or 26 or 27 or 28	6406664

31 30 not 29 5621101
32 exp Qualitative Research/ 108442
33 qualitative*.tw. 446898
34 interview*.tw. 527278
35 experienc*.tw. 1657277
36 32 or 33 or 34 or 35 2278518
37 31 or 36 7354323
38 meta-analysis/ or systematic review/ or systematic reviews as topic/ or meta-analysis as topic/ or "meta analysis (topic)"/ or "systematic review (topic)"/ 418967
39 ((systematic* or systematized or integrative or mapping or rapid or scoping) adj3 (review* or overview*)).tw. 465813
40 ((evidence or interpretive or meta or quantitative or qualitative or integrative or collaborative or mixed method*) adj3 (synthes* or review* or overview*)).tw. 303456
41 ("meta-analys?s" or metaanalys?s or metasynth*).tw. 351160
42 systematic review.pt. 300479
43 meta-analysis.pt. 215530
44 (cost* adj3 review*).tw. 4678
45 (data adj extraction).ab. 43411
46 (narrative adj (review* or synthes?s)).tw. 54659
47 (cochrane or evidence report).jw. 17556
48 38 or 39 or 40 or 41 or 42 or 43 or 44 or 45 or 46 or 47 763004
49 37 or 48 7774837
50 20 and 49 12901

Ovid MEDLINE(R). Original search 2022

1 *"aged, 80 and over"/ or centenarians/ or nonagenarians/ or octogenarians/ (1753)
2 (oldest and (people* or person* or adult* or old or elderly)).ti. (1450)
3 (oldest adj2 (people* or person* or adult* or old or elderly)).ab. (2827)
4 "very old*".tw. (4822)
5 centenarian*.tw. (2271)
6 nonagenarian*.tw. (1637)
7 octogenarian*.tw. (3725)
8 (over 85 or over 85s).tw. (2712)
9 (over 90 or over 90s).tw. (16423)
10 (over 80 or over 80s).tw. (13270)
11 (year* or age*).tw. (6686226)

12 8 or 9 or 10 (32023)
13 ((year* or age*) adj2 (over 85 or over 85s or over 90 or over 90s or over 80 or over 80s)).tw. (3586)
14 1 or 2 or 3 or 4 or 5 or 6 or 7 or 13 (18147)
15 randomized controlled trial.pt. (579325)
16 controlled clinical trial.pt. (95078)
17 randomi#ed.ab. (692659)
18 placebo$.ab. (233439)
19 drug therapy.fs. (2540699)
20 randomly.ab. (393888)
21 trial.ab. (620810)
22 groups.ab. (2424322)
23 exp animals/ not humans.sh. (5057996)
24 15 or 16 or 17 or 18 or 19 or 20 or 21 or 22 (5510892)
25 24 not 23 (4804559) *1
26 exp Qualitative Research/ (77541)
27 qualitative*.tw. (343848)
28 interview*.tw. (429482)
29 experienc*.tw. (1337023)
30 26 or 27 or 28 or 29 (1860128) * 2
31 25 or 30 (6224453)
32 meta-analysis/ or systematic review/ or systematic reviews as topic/ or meta-analysis as topic/ or "meta analysis (topic)"/ or "systematic review (topic)"/ (313708)
33 ((systematic* or systematized or integrative or mapping or rapid or scoping) adj3 (review* or overview*)).tw. (304675)
34 ((evidence or interpretive or meta or quantitative or qualitative or integrative or collaborative or mixed method*) adj3 (synthes* or review* or overview*)).tw. (205514)
35 ("meta-analys?s" or metaanalys?s or metasynth*).tw. (245480)
36 systematic review.pt. (210384)
37 meta-analysis.pt. (169546)
38 (cost* adj3 review*).tw. (3794)
39 (data adj extraction).ab. (29676)
40 (narrative adj (review* or synthes?s)).tw. (27823)
41 (cochrane or evidence report).jw. (16344)
42 32 or 33 or 34 or 35 or 36 or 37 or 38 or 39 or 40 or 41 (540180)
43 31 or 42 (6517886)
44 14 and 43 (6979)

*1 Cochrane HSS filter Glanville J, Kotas E, Featherstone R, Dooley G. Which are the most sensitive search filters to identify randomized controlled trials in MEDLINE? J Med Libr Assoc. 2020 Oct 1;108(4):556-563. doi: 10.5195/jmla.2020.912. PMID: 33013212; PMCID: PMC7524635.

2i PsycINFO

2022 and 2024 search strategies the same

1 (oldest and (people* or person* or adult* or old or elderly)).ti. 638
2 (oldest adj2 (people* or person* or adult* or old or elderly)).ab. 1324
3 "very old*".tw. 1616
4 centenarian*.tw.507
5 nonagenarian*.tw. 173
6 octogenarian*.tw. 221
7 (over 85 or over 85s).tw. 403
8 (over 90 or over 90s).tw. 1848
9 (over 80 or over 80s).tw. 1817
10 (year* or age*).tw. 1470110
11 7 or 8 or 9 4033
12 ((over 85 or over 85s or (over 90 or over 90s) or (over 80 or over 80s)) adj2 (year* or age*)).tw. 394
13 1 or 2 or 3 or 4 or 5 or 6 or 12 3849
14 clinical trials/ or exp randomized controlled trials/ 13755
15 randomi#ed.ti,ab. 107290
16 placebo$.ab. 43936
17 randomly.ab. 84938
18 trial.ti,ab. 127911
19 groups.ab. 568423
20 or/14-19 773794
21 *meta analysis/ 3871
22 exp "Systematic Review"/ 839
23 ((systematic* or systematized or integrative or mapping or rapid or scoping) adj3 (review* or overview*)).tw. 65220
24 ((evidence or interpretive or meta or quantitative or qualitative or integrative or collaborative or mixed method*) adj3 (synthes* or review* or overview*)).tw. 52892
25 ("meta-analys?s" or metaanalys?s or metasynth*).tw. 50650
26 (cost* adj3 review*).tw. 492
27 (data adj2 extract*).ti,ab. 9676
28 (narrative adj (review* or synthes?s)).tw. 6127

29 "systematic review".md. 45971
30 "meta analysis".md. 31823
31 or/21-30 132826
32 20 or 31 878890
33 exp Qualitative Methods/ 21547
34 interview*.tw. 401004
35 (("semi-structured" or semistructured or unstructured or informal or "in-depth" or indepth or "face-to-face" or structured or guide or guides) adj3 (discussion* or questionnaire*)).ti,ab. 12200
36 (focus group* or qualitative or ethnograph* or fieldwork or "field work" or "key informant").ti,ab,id. 282703
37 qualitative study.md. 318968
38 or/33-37 646336
39 32 or 38 1401878
40 13 and 39 1344

University of Texas:

https://libguides.sph.uth.tmc.edu/search_filters/ovid_psycinfo_filters as a guide for terms

2j. PQDT

2022 and 2024 search strategy the same

Set#	Searched for	Databases	Results
S3	(abstract("oldest old") OR title("oldest old")) OR (title("over 85") or title("over 85s") or title("over 90") or title("over 90s") or title("very old") or title("octogenarian*") or title("nonogenarian*") or title("cenenarian*") or abstract("octogenarian*") or abstract("nonogenarian*") or abstract("centenarian*"))	ProQuest Dissertations & Theses Global	391
S4	abstract("frail elderly") OR title("frail elderly") OR abstract("frail older") OR title("frail older")	ProQuest Dissertations & Theses Global	483
S5	[S3] OR [S4]	ProQuest Dissertations & Theses Global	867

S6	abstract(trial*) OR title(trial*) OR abstract(random*) OR title(random*) OR abstract(groups) OR title(groups) OR abstract(synthes* or evidence* or review* or overview*) OR title(synthes* or evidence* or review* or overview*) OR abstract(qualitativ* or interview* or experienc*) OR title(qualitativ* or interview* or experienc*)	ProQuest Dissertations & Theses Global	2120365
S7	[S5] AND [S6]	ProQuest Dissertations & Theses Global	643

2k. SPP

Update search strategy 2024. Edits in red

1 very old*.tw. 354
2 centenarian*.tw.152
3 nonagenarian*.tw. 26
4 octogenarian*.tw. 35
5 (over 85 or over 85s).tw. 98
6 (over 90 or over 90s).tw. 147
7 (over 80 or over 80s).tw. 257
8 (year* or age*).tw. 124045
9 5 or 6 or 7 494
10 ((year* or age*) adj5 (over 85 or over 85s or (over 90 or over 90s) or (over 80 or over 80s))).tw. 126
11 (oldest and (people* or person* or adult* or old or olds or **women or men** or elderly)).ti. 192
12 (oldest adj5 (people* or person* or adult* or old or olds or **women or men** or elderly)).ab. 417
13 **(frail adj2 older).tw.** 787
14 1 or 2 or 3 or 4 or 10 or 11 or 12 or 13 1816
15 trial*.tw.5248
16 random*.tw. 5721
17 groups.tw. 36304
18 15 or 16 or 17 43069
19 (synthes* or review* or overview* or evidence*).tw. 94705
20 (qualitativ* or interview* or experienc*).tw. 90677
21 18 or 19 or 20 186481
22 14 and 21 915

Original search strategy 2022

1 very old*.tw. (349)
2 centenarian*.tw. (149)
3 nonagenarian*.tw. (26)
4 octogenarian*.tw. (34)
5 (over 85 or over 85s).tw. (97)
6 (over 90 or over 90s).tw. (148)
7 (over 80 or over 80s).tw. (246)
8 (year* or age*).tw. (122630)
9 5 or 6 or 7 (483)
10 ((year* or age*) adj5 (over 85 or over 85s or (over 90 or over 90s) or (over 80 or over 80s))).tw. (121)
11 (oldest and (people* or person* or adult* or old or olds or elderly)).ti. (186)
12 (oldest adj5 (people* or person* or adult* or old or olds or elderly)).ab. (397)
13 1 or 2 or 3 or 4 or 10 or 11 or 12 (1010)
14 trial*.tw. (5244)
15 random*.tw. (5761)
16 groups.tw. (36020)
17 14 or 15 or 16 (42798)
18 (synthes* or review* or overview* or evidence*).tw. (93777)
19 (qualitativ* or interview* or experienc*).tw. (89803)
20 17 or 18 or 19 (184828)
21 13 and 20 (463)
NOTE: a bit broader than Medline search as smaller database. Doesn’t have any controlled vocab

2l. Web of Science. Science Citation Index (1900), Social Science Citation Index (1900), Arts and Humanities Citation Index (1975-), Conference Proceedings Citation Index – Science (1990-), Conference Proceedings Citation Index – Social Science and Humanities (1990-), Emerging Sources Citation Index (2015-). 2022 and 2024

8 #7 OR #5 OR #3 2,706 (restricted from 2023: 154)
7 #1 AND #6 503
6 ((TS=((systematic* or systematized or integrative or mapping or rapid or scoping) NEXT (review or overview))) OR TS=((evidence or interpretive or meta or quantitative or qualitative or integrative or collaborative or "mixed method*") NEXT (synthes* or review* or overview*))) OR TS=(("meta-analys?s" or metaanalys?s or metasynth*)) 491,855
5 #4 AND #1 1,881
4 ((TS=(qualitative)) OR TS=(interview*)) OR TS=((experience or experiences))

3,204,399
3 #2 AND #1 524
2 (((TS=("randomized controlled trial")) OR TS=("controlled clinical trial")) OR AB=(randomi$ed)) OR AB=(randomly) 1,192,934
1 ((TI=((oldest NEXT (person* or people or adult* or old or elderly))) OR TS=(octogenarian OR nonagenarian OR centenarian)) OR TS=("very old*")) 16,844

APPENDIX (2) Original Search Summary Table (2023)

Health and social care interventions in the 80 years old and over population: an evidence and gap map															
**Included references**		**Database searches (*date run: Oct 2022, date re-run: Nov 2023*)**													
	Type	***Ageline***	***Assia***	***Campbell***	***CDSR***	***Central***	***Cinahl***	***Embase***	***Epist***	***HMIC***	***Medline***	***Psycinfo***	***PQDT***	***SPP***	***WoS***
Aas, S. N., et al. 2020	rct	***x***				***x***	***x***				***y***				***x***
Abraha, I., et al. 2017	sr							***x***			***x***				
Alexopoulos, G., et al. 2004	rct		***x***			***x***	***x***				***x***	***x***			***x***
Amofah, H. A., et al. 2021	qual							***x***			***x***				***x***
Bai, X., et al. 2021	sr							***x***	***x***		***x***				***x***
Baleztena, J., et al. 2018	rct					***x***					***x***				***x***
Barbosa, F. A., et al. 2016	rct										***n***				***x***
Barssoum, K., et al. 2021	sr						***x***		***x***		***x***				***x***
Baumgartner, A., et al. 2021	rct					***x***					***x***				***x***
Bechshoft, R. L., et al. 2017	rct					***x***					***x***				
Behm, L., et al. 2016	rct					***x***	***x***				***x***				***x***
Behm, L., et al. 2013	rct						***x***	***x***			***x***				***x***

Behm, L., et al. 2014	rct					***x***	***x***				***x***				***x***
Boonen, S., et al. 2004	rct		***x***			***x***	***x***				***x***			***x***	***x***
Bruunsgaard, H., et al. 2004	rct					***x***	***x***				***x***				
Bueno, H., et al. 2011	rct					***x***					***x***				***x***
Burden, E., et al. 2020	qual							***x***			***x***				
Burdinski, S., et al. 2022	rct										***x***	***x***			***x***
Burrows, A. B., et al. 2002	rct					***x***					***x***				***x***
Burtin, P., et al. 1995	rct										***x***				
Bynum, J. P., et al. 2014	qual						***x***	***x***			***x***	***x***			***x***
Cadore, E. L., et al. 2014	rct					***x***					***x***				***x***
Calder, S. J., et al. 1996	rct					***x***					***x***				***x***
Campbell, A. J., et al. 1999	rct		***x***								***z***				
Campo-Prieto, P., et al. 2022	rct										***x***				
Carral, J. M. C., et al. 2019	rct					***x***					***x***				***x***
Cederbom, S., et al. 2019	rct	***x***				***x***					***x***				***x***
Chammout, G., et al. 2019	rct					***x***					***x***				
Chen, Y.-Y., et al. 2020	qual	***x***					***x***			***x***	***n***	***x***		***x***	
Chena, J. O., et al. 2010	qual										***x***				
Chiang, F. Y., et al. 2021	rct										***x***				***x***
Clough, A. J., et al.	rct										***x***				

2019															
Cockayne, S., et al. 2021	rct										***x***				
Cordes, T., et al. 2021	rct					***x***					***x***				***x***
Courage, O., et al. 2021	sr							***x***			***x***				
Crotty, M., et al. 2019	rct	***x***				***x***	***x***				***x***				***x***
Davidson, T., et al. 2015	rct									***x***	***z***				
de Asteasu, M. L. S., et al. 2019 assessing	rct					***x***	***x***				***x***				***x***
de Asteasu, M. L. S., et al. 2019 physical	rct					***x***	***x***				***x***				***x***
de Belder, A., et al. 2014	rct					***x***	***x***				***x***				***x***
Dozeman, E., et al. 2011	rct	***x***				***x***	***x***				***x***	***x***			***x***
Drahota, A. K. 2013	rct									***x***	***z***				
Ehsani, A. A., et al. 2003	rct					***x***					***x***				***x***
Eriksen, C. S., et al. 2018	rct					***x***					***x***				***x***
Eriksen, K. S., et al. 2021	qual						***x***				***n***				
Ersek, M., et al. 2003	rct										***x***				
Ferrer, A., et al. 2014	rct	***x***				***x***					***x***				***x***
Fiatarone, M. A., et al. 1994	rct										***z***			***x***	
Flamaing, J., et al. 2008	rct					***x***	***x***				***x***				
Foley, C., et al. 2022	sr							***x***	***x***		***x***				
Freixa, X., et al. 2012	rct					***x***					***x***				***x***
Fu, M., et al. 2020	rct					***x***					***x***				

Gallingani, A., et al. 2022	sr							***x***	***x***		***x***				***x***
Gill, T. M. 2002	rct									***x***	***z***				
Greuter, L., et al. 2020	rct										***z***				***x***
Haanes, G. G., et al. 2015	rct										***x***				
Haas, L. E. M., et al. 2017	sr							***x***	***x***		***n***				
Hajibandeh, S., et al. 2021	sr							***x***	***x***		***x***				***x***
Han, S. J., et al. 2017	rct										***x***				
Hanson, E. J. and Clarke, A. 2000	qual									***x***	***z***				
Hashimoto, M., et al. 2017	rct					***x***	***x***				***x***	***x***			
Hassan, B. H., et al. 2016	rct	***x***				***x***	***x***				***x***				***x***
Hjelm, M., et al. 2015	qual							***x***		***x***	***z***				
Hofmann, M., et al. 2016	qr					***x***	***x***				***x***				***x***
Holland, R., et al. 2005	rct		***x***							***x***	***x***				
Hutcheon, S. D., et al. 2002	rct					***x***	***x***				***x***				
Imhof, L., et al. 2012	rct	***x***									***z***				
Joling, K. J., et al. 2011	rct	***x***				***x***	***x***				***x***	***x***			***x***
Jonker, A. A. G. C., et al. 2015	rct									***x***	***z***				
Kalapotharakos, V. I., et al. 2010	rct	***x***				***x***	***x***				***x***				***x***
Karlsen, A., et al. 2019	rct					***x***					***x***				***x***
Khatri, K., et al. 2022	rct										***x***				
Kryger, A. I. and Andersen, J. L. 2007	rct					***x***	***x***				***x***				

Kylberg, M., et al. 2013	qual						***x***	***x***			***x***				***x***
Langkamp-Henken, B., et al. 2000	rct					***x***	***x***				***x***				***x***
Legrain, S etal 2011	rct	***x***				***x***	***x***				***x***	***x***			
Lenaghan, E., et al. 2007	rct		***x***								***x***				
Lewin, G., et al. 2013	rct		***x***			***x***	***x***			***x***	***x***	***x***		***x***	
Liang, H., et al. 2020	sr							***x***	***x***		***x***				***x***
Lin, F. V., et al. 2021	qual	***x***				***x***	***x***				***x***	***x***			
Lorenzano, S., et al. 2021	sr							***x***			***x***				
Louvard, Y., et al. 2004	rct					***x***					***x***				
Louzada, L. L., et al. 2022	rct										***z***	***x***			
Malaguarnera, M., et al. 2007	rct					***x***	***x***				***x***				***x***
Maniewicz, S., et al. 2019	rct					***x***					***x***				***x***
Manor, B., et al. 2014	rct	***x***				***x***	***x***				***x***	***x***			
Martinez-Velilla, N., et al. 2022	rct					***x***	***x***				***x***				
Martinez-Velilla, N., et al. 2021	rct					***x***					***x***				
Maruyama, M., et al. 2016	rct					***x***	***x***				***x***				
McCombie, A. M., et al. 2021	qual										***x***				
McCord, A., et al. 2020	rct					***x***	***x***				***n***	***x***			
McMurdo, M. E. and Rennie, L. 1993	rct										***x***				
Mende, E., et al. 2022	sr							***x***	***x***		***x***				
Meng, R., et al. 2015	rct					***x***	***x***				***x***	***x***			***x***
Minai, K., et al. 2002	rct										***x***				

Monteserin, R. 2010	rct									***x***	***z***				
Morioka, S., et al. 2011	rct					***x***	***x***				***x***	***x***			***x***
Muller, F., et al. 2013	rct					***x***	***x***				***x***				***x***
Naughton, B. J., et al. 1994	rct									***x***	***z***				
Nava, S., et al. 2011	rct	***x***				***x***	***x***				***x***				***x***
Niki, K., et al. 2020	rct					***x***					***x***	***x***			
Nygårdh, A., et al. 2016	qual							***x***		***x***	***z***				
Oliveira, J., et al. 2016	rct					***x***	***x***				***x***				***x***
Ortiz-Alonso, J., et al. 2020	rct					***x***	***x***				***x***				***x***
Owen, R., et al. 2022	sr	***x***					***x***	***x***			***x***				
Pedone, C., et al. 2015	rct	***x***				***x***	***x***				***x***				***x***
Peskind, E. R., et al. 2005	rct					***x***					***x***	***x***			***x***
Porter, E. J. and Ganong, L. H. 2005	qual										***x***				
Price, R., et al. 2005	rct					***x***					***x***				***x***
Puggaard, L., et al. 1999	rct										***x***				
Puggaard, L., et al. 2000	rct										***x***				***x***
Rash, A., et al. 2007	rct		***x***			***x***	***x***				***x***				***x***
Rimon, E., et al. 2005	rct					***x***					***x***				***x***
Rodrigues, R. N., et al. 2022	rct										***x***				
Rodriguez-Diaz, M. T., et al. 2016	rct										***z***	***x***			***x***
Rosie, J. and Taylor, D. 2007	rct	***x***	***x***				***x***				***x***				
Ruggiero, C., et al. 2019	rct					***x***	***x***				***x***				

Ruiz, J. R., et al. 2015	rct					***x***	***x***				***x***				***x***
Salter, C. 2007	qual									***x***	***z***				
Scherder E. J, A. and al., e. 2005	rct					***x***	***x***				***x***	***x***		***x***	
Schonberg, M. A., et al. 2006	qual							***x***			***x***	***x***			
Seeman, E., et al. 2010	rct										***x***				
Seeman, E., et al. 2006	rct					***x***					***x***				
Seinela, L., et al. 2003	rct					***x***					***x***				***x***
Serra-Rexach, J. A., et al. 2011	rct	***x***				***x***	***x***				***x***				***x***
Simons, R. and Andel, R. 2006	rct					***x***	***x***				***x***	***x***		***x***	***x***
Skelton, D. A., et al. 1995	rct					***x***					***x***				***x***
Stappaerts, K. H., et al. 1995	rct					***x***	***x***				***x***				***x***
Strasser, E. M., et al. 2018	rct					***x***	***x***				***x***				***x***
Thompson, W., et al. 2018	sr						***x***		***x***		***z***				
Tibaldi, V., et al. 2013	rct					***x***					***n***				
Timonen, L., et al. 2006	rct					***x***	***x***				***x***				***x***
Timonen, L., et al. 2002	rct					***x***	***x***				***x***				***x***
Tomsone, S., et al. 2016	qual						***x***	***x***			***x***				***x***
Valenzuela, P. L., et al. 2020	rct					***x***					***x***				
van der Weele, G. M., et al. 2012 Response	qual	***x***									***z***				
van der Weele, G. M., et al. 2012 effects	rct	***x***									***z***				
van Halsema, M. S., et	sr							***x***	***x***		***x***				

al. 2021															
Van Roie, E., et al. 2017	rct					***x***	***x***				***x***				
West, R. K., et al. 2020	rct	***x***				***x***	***x***				***x***	***x***			
Widell, C., et al. 2020	qual						***x***	***x***			***x***				***x***
Widimsky, J. 2008	rct					***x***					***n***				
Wieland, J., et al. 2022	qual							***x***			***x***				***x***
Wising, J., et al. 2022	qual						***x***	***x***			***x***				***x***
Wubbeke, L. F., et al. 2020	sr							***x***	***x***		***x***				***x***
Zak, M. and Gryglewska, B. 2006	rct					***x***					***n***				
Zidén, L., et al. 2014	rct	x									z				
No. included refs		22	8	0	0	78	59	25	11	13	118	22	0	6	71
No. refs in the database; not retrieved											20				
No. unique refs		3	3	0	0	3	1	0	0	8	16	1	0	1	2
x = found from the search															
y = in database; found when search strategy re-run															
n = not in the database															
z = in the database; not found using the search strategy															
**(red) =** databases where searches re-run															

APPENDIX (3) – Final search summary table

Health and social care interventions in the 80 years old and over population: an evidence and gap map																
**Included references**		**Database searches (*date run: Oct 22. Update search: Jul 24)***													**Suppl. searches**	
	Type	***Ageline***	***Assia***	***CDSR***	***Central***	***Cinahl***	***Embase***	***Epist***	***HMIC***	***Medline***	***Psycinfo***	***PQDT***	***spp***	***WoS***	***fcs***	***bcs***
Akao, 2024	rct														***x***	
Akashi, S., et al. 2022	rct														***x***	
Alassaad, A., et al. 2014	rct				***xup***											
Álvarez-Barbosa, F., et al. 2014	rct				***xup***											
Amofah, H. A., et al. 2021	qual						***x***			***x***				***x***		
Ansai, J. H., et al. 2016	rct				***x***	***x***				***x***				***x***		
Ansai, J. H. and Rebelatto, J. R. 2015	rct				***x***	***x***				***x***	***x***					
Bai, X., et al. 2021	sr						***x***	***x***		***x***				***x***		
Barbosa, F. A., et al. 2016	rct													***x***		
Barker, R., et al. 2006	rct				***xup***											
Barssoum, K., et al. 2021	sr					***x***		***x***		***x***				***x***		
Bays-Moneo, A. B., et al. 2023	rct				***xup***					***xup***						
Bechshoft, R. L., et al. 2017	rct				***x***					***x***						
Beckett, N., et al. 2011	rct														***x***	
Beckett, N. S., et al. 2008	rct				***xup***											
Behm, L., et al. 2016	rct				***x***	***x***				***x***				***x***		
Behm, L., et al. 2013	rct					***x***	***x***			***x***				***x***		
Behm, L., et al. 2014	rct				***x***	***x***				***x***				***x***		
Berg, E. S., et al. 2023	rct				***xup***					***xup***						
Berkhout, M., et al.	qual		***xup***							***xup***						

2022																
Bhatnagar, P., et al. 2011	sr					***x***				***x***	***x***					
Biancari, F., et al. 2017	sr							***x***		***x***				***x***		
Boonen, S., et al. 2004	rct		***x***		***x***	***x***				***x***			***x***	***x***		
Brettschneider, C., et al. 2015	rct				***xup***					***xup***						
Briand, M., et al. 2022	sr							***x***		***x***						
Bruunsgaard, H., et al. 2004	rct				***x***	***x***				***x***						
Bulpitt, C. J., et al. 2013	rct				***xup***											
Bulpitt, C. J., et al. 2003	rct															***x***
Bulpitt, C. J., et al. 2012	rct				***xup***											
Buondonno, I., et al. 2020	rct				***xup***											
Burden, E., et al. 2020	qual						***x***			***x***						
Burrows, A. B., et al. 2002	rct				***x***					***x***				***x***		
Burtin, P., et al. 1995	rct									***x***						
Bynum, J. P., et al. 2014	qual					***x***	***x***			***x***	***x***			***x***		
Cadore, E. L., et al. 2014	rct				***x***					***x***				***x***		
Calder, S. J., et al. 1996	rct				***x***					***x***				***x***		
Campbell, A. J., et al. 1999	rct		***x***													
Campbell, A. J., et al. 1997	rct															***x***
Campo-Prieto, P., et al. 2022	rct									***x***						
Carral, J. M. C., et al. 2019	rct				***x***					***x***				***x***		
Chammout, G., et al. 2019	rct				***x***					***x***						
Chen, B., et al. 2023	sr							***xup***		***xup***				***xup***		

Chen, Y.-Y., et al. 2020	qual	***x***				***x***			***x***		***x***		***x***			
Clough, A. J., et al. 2019	rct									***x***						
Cofre-Bolados, C., et al. 2023	rct									***xup***						
Courage, O., et al. 2021	sr						***x***			***x***						
de Belder, A., et al. 2014	rct				***x***	***x***				***x***				***x***		
de Belder, A., et al. 2021	rct														***x***	
DeBernardis, D. A., et al. 2023	sr														***x***	
Dodge, H. H., et al. 2008	rct				***xup***											
Duffis, E. J., et al. 2013	sr							***x***		***x***				***x***		
Ehsani, A. A., et al. 2003	rct				***x***					***x***				***x***		
Elahwal, M., et al. 2023	sr							***xup***		***xup***						
Engelter, S. T., et al. 2006	sr															***x***
Eriksen, C. S., et al. 2018	rct				***x***					***x***				***x***		
Eriksen, K. S., et al. 2021	qual					***x***										
Ferrer, A., et al. 2014	rct	***x***			***x***					***x***				***x***		
Freixa, X., et al. 2012	rct				***x***					***x***				***x***		
Fu, M., et al. 2020	rct				***x***					***x***						
Ganske, K. M. 2002	qual	***x***				***x***						***x***				
Gavazzi, G., et al. 2022	rct				***xup***					***xup***				***xup***		
Gene Huguet, L., et al. 2018	rct				***xup***											
Gillespie, U., et al. 2013	rct				***xup***											
Gillespie, U., et al. 2009	rct				***xup***											
Gine-Garriga, M., et al. 2010	rct				***xup***					***xup***						

Giné-Garriga, M., et al. 2013	rct				***xup***											
Godwin, M., et al. 2016	rct				***xup***											
Greenspan, S. L., et al. 2020	rct				***xup***											
Greuter, L., et al. 2020	rct													***x***		
Gustafsson, S., et al. 2013	rct				***xup***					***xup***						
Gustafsson, S., et al. 2012	rct				***xup***					***xup***						
Haanes, G. G., et al. 2015	rct									***x***						
Haas, L. E. M., et al. 2017	sr						***x***	***x***								
Hagiwara, E., et al. 2019	rct				***xup***											
Hajibandeh, S., et al. 2021	sr						***x***	***x***		***x***				***x***		
Han, S. J., et al. 2017	rct									***x***						
Hedbeck, C. J., et al. 2011	rct				***xup***											
Hilt, P. M., et al. 2023	rct									***xup***						
Hirlekar, G., et al. 2020	rct				***xup***											
Hjelm, M., et al. 2015	qual						***x***		***x***							
Holland, R., et al. 2005	rct		***x***						***x***	***x***						
Imhof, L., et al. 2012	rct	***x***			***xup***	***xup***										
Instenes, I., et al. 2024	qual														***x***	
Iqbal, U., et al. 2022	sr					***x***		***x***		***x***				***x***		
Johnman, C., et al. 2013	sr					***x***		***x***	***x***	***x***				***x***		
Kalapotharakos, V. I., et al. 2010	rct	***x***			***x***	***x***				***x***				***x***		
Karlsen, A., et al. 2019	rct				***x***					***x***				***x***		
Khatri, K., et al. 2022	rct									***x***						
Kim, S. Y., et al. 2017	sr							***x***		***x***				***x***		
Kryger, A. I. and Andersen, J. L. 2007	rct				***x***	***x***				***x***						

Kylberg, M., et al. 2013	qual					***x***	***x***			***x***				***x***		
Lee, K.-H., et al. 2022	sr	***xup***				***xup***										
Lenaghan, E., et al. 2007	rct		***x***							***x***						
Liang, H., et al. 2020	sr						***x***	***x***		***x***				***x***		
Liang, Y., et al. 2020	rct				***xup***											
Lin, N. H., et al. 2022	sr														***x***	
Lin, Y. L., et al. 2009	rct				***xup***											
Lofqvist, C., et al. 2009	qual					***x***				***x***				***x***		
Lopiz, Y., et al. 2019	rct				***xup***											
Lorenzano, S., et al. 2021	sr						***x***			***x***						
Louvard, Y., et al. 2004	rct				***x***					***x***						
Lu, Q., et al. 2017	rct				***xup***											
Luck, T., et al. 2013	rct				***xup***					***xup***						
Luukinen, H., et al. 2007	rct				***xup***											
Malaguarnera, M., et al. 2007	rct				***x***	***x***				***x***				***x***		
Marcellaud, E., et al. 2023	sr									***xup***						
McClung, M. R., et al. 2018	rct				***xup***											
McCombie, A. M., et al. 2021	qual									***x***						
McCord, A., et al. 2020	rct				***x***	***x***					***x***					
Mende, E., et al. 2022	sr						***x***	***x***		***x***						
Meng, R., et al. 2015	rct				***x***	***x***				***x***	***x***			***x***		
Merga, Z. C., et al. 2023	sr													***xup***		
Mikami, T., et al. 2022	rct														***x***	
Minai, K., et al. 2002	rct									***x***						
Namkoong, H., et al. 2015	rct				***xup***											
Nana, P., et al. 2023	sr							***xup***		***xup***				***xup***		

Nicolson, P. J. A., et al. 2021	sr														***x***	
Noguchi, M., et al. 2022	sr							***x***		***x***				***x***		
Oberic, L., et al. 2021	rct				***xup***											
Okazaki, O., et al. 2022	rct														***x***	
Okumura, K., et al. 2020	rct				***xup***											
Oliveira, J., et al. 2016	rct				***x***	***x***				***x***				***x***		
Ouwehand, A. C., et al. 2008	rct				***xup***											
Owen, R., et al. 2022	sr	***x***				***x***	***x***			***x***						
Pajewski, N. M., et al. 2019	rct														***x***	
Pershad, A., et al. 2014	rct				***x***					***x***				***x***		
Peters, R., et al. 2010	rct				***xup***											
Peters, R., et al. 2008	rct				***xup***											
Peters, R., et al. 2013	rct				***xup***											
Puggaard, L., et al. 1999	rct									***x***						
Puggaard, L., et al. 2000	rct									***x***				***x***		
Rash, A., et al. 2007	rct		***x***		***x***	***x***				***x***				***x***		
Rautalin, I., et al. 2021	sr							***x***		***x***						
Rimon, E., et al. 2005	rct				***x***					***x***				***x***		
Rodrigues, R. N., et al. 2022	rct									***x***						
Rosado, P., et al. 2008	rct				***x***											
Rosie, J. and Taylor, D. 2007	rct	***x***	***x***			***x***				***x***						
Ruiz, J. R., et al. 2015	rct				***x***	***x***				***x***				***x***		
Salter, C. 2007	qual								***x***							
Schonberg, M. A., et al. 2006	qual						***x***			***x***	***x***					
Seeman, E., et al.	rct									***x***						

2010																
Seeman, E., et al. 2006	rct				***x***					***x***						
Seinela, L., et al. 2003	rct				***x***					***x***				***x***		
Serra-Rexach, J. A., et al. 2011	rct	***x***			***x***	***x***				***x***				***x***		
Shantsila, E., et al. 2023	sr														***x***	
Sheppard, J. P., et al. 2020	rct				***xup***											
Sun, L., et al. 2022	sr							***x***		***x***				***x***		
Tegn, N., et al. 2016	rct				***xup***											
Tegn, N., et al. 2018	rct				***xup***											
Tegn, N., et al. 2020	rct				***xup***											
Thommessen, B., et al. 2021	rct				***xup***											
Tomsone, S., et al. 2016	qual					***x***	***x***			***x***				***x***		
van Halsema, M. S., et al. 2021	sr						***x***	***x***		***x***						
Vorilhon, C., et al. 2016	rct				***xup***											
Wang, G., et al. 2023	sr									***xup***				***xup***		
Wang, Q. X., et al. 2010	rct				***xup***											
Warwick, J., et al. 2015	rct				***xup***					***xup***						
Watkins, A. R., et al. 2022	sr					***x***		***x***		***x***				***x***		
Rabin, et al. 2016	rct				***x***						***x***	***x***				
West, R. K., et al. 2020	rct	***x***			***x***	***x***				***x***	***x***					
Wilhelmson, K. and Eklund, K. 2013	rct									***xup***						
Wising, J., et al. 2022	qual					***x***	***x***			***x***				***x***		
Wubbeke, L. F., et al. 2020	sr						***x***	***x***		***x***				***x***		
Xu, Y., et al. 2022	rct														***x***	
Xu, Y., et al. 2019	sr					***x***		***x***		***x***				***x***		

Yamashita, K., et al. 2009	rct				***xup***											
Yamashita, T., et al. 2024	rct														***x***	
Yan, L., et al. 2019	sr					***x***										
Yoshida, T., et al. 2022	rct				***xup***											
Zak, M. and Gryglewska, B. 2006	rct				***x***											
Zhou, J., et al. 2020	rct				***xup***											
Zidén, L., et al. 2014	rct	x			***xup***	***xup***										
No. included refs		10	6	0	37	35	19	19	5	79	9	2	2	48	14	3
update search		0	1	0	51	3	0	3	0	18	0	0	0	5	14	3
No. unique refs		3	3	0	3	1	0	0	8	16	1	0	1	2		
Yield		1872	592	13	1393	1872	6152	239	1016	11341	1344	643	915	154		
Sensitivity		6.29	3.77	0.00	23.27	22.01	11.95	11.95	3.14	49.69	5.66	1.26	1.26	30.19		
Precision		0.53	1.01	0.00	2.66	1.87	0.31	7.95	0.49	0.70	0.67	0.31	0.22	31.17		
No. database searches carried out =				18												
Sum of yields =				27,546				Overall sensitivity		92.44						
No. refs screened at Ti&Ab =				18,073				Overall precision		0.88						
No. refs screened at FT=				1,099				NNR		113.67						
No. of included refs from searching =				159				NNR FT		6.91						
Total no. of included refs =				172				NNS		16.44						
**Codes**						**Other codes**										
x = found from the search						NNR = number needed to read. 1/overall precision										
y = in database; found when search strategy re-run						NNR FT = number needed to read at FT to find one included reference										
n = not in the database						NNS = number needed to screen to find one reference to include for FT screening										
z = in the database; not found using the search strategy						FT = full text										
xup = update search																

APPENDIX (4) – List of Included Studies

1. Akao M, Yamashita T, Fukuzawa M, Hayashi T, Okumura K. Efficacy and Safety of Low-Dose Edoxaban by Body Weight in Very Elderly Patients With Atrial Fibrillation: A Subanalysis of the Randomized ELDERCARE-AF Trial. Journal of the American Heart Association. 2024;13(3).

2. Akashi S, Oguri M, Ikeno E, Manita M, Taura J, Watanabe S, et al. Outcomes and Safety of Very-Low-Dose Edoxaban in Frail Patients With Atrial Fibrillation in the ELDERCARE-AF Randomized Clinical Trial. JAMA network open. 2022;5(8).

3. Alassaad A, Bertilsson M, Gillespie U, Sundström J, Hammarlund-Udenaes M, Melhus H. The effects of pharmacist intervention on emergency department visits in patients 80 years and older: subgroup analyses by number of prescribed drugs and appropriate prescribing. PloS one. 2014;9(11):e111797.

4. Álvarez-Barbosa F, del Pozo-Cruz J, del Pozo-Cruz B, Alfonso-Rosa RM, Rogers ME, Zhang Y. Effects of supervised whole body vibration exercise on fall risk factors, functional dependence and health-related quality of life in nursing home residents aged 80+. Maturitas. 2014;79(4):456-63.

5. Amofah HA, Brostrom A, Instenes I, Fridlund B, Haaverstad R, Kuiper K, et al. Octogenarian patients' sleep and delirium experiences in hospital and four years after aortic valve replacement: a qualitative interview study. BMJ Open. 2021;11(1):e039959.

6. Ansai JH, Aurichio TR, Goncalves R, Rebelatto JR. Effects of two physical exercise protocols on physical performance related to falls in the oldest old: A randomized controlled trial. Geriatrics & gerontology international. 2016;16(4):492-9.

7. Ansai JH, Rebelatto JR. Effect of two physical exercise protocols on cognition and depressive symptoms in oldest-old people: A randomized controlled trial. Geriatrics & gerontology international. 2015;15(9):1127-34.

8. Bai X, Zhang X, Zhang Y, Yang W, Wang T, Feng Y, et al. Mechanical Thrombectomy in Nonagenarians: a Systematic Review and Meta-analysis. Translational Stroke Research. 2021;12(3):394-405.

9. Barbosa FA, Cruz JD, Cruz BD. Whole body vibration in institutionalized older people over the age of 80 to improve static balance. Retos-Nuevas Tendencias En Educacion Fisica Deporte Y Recreacion. 2016(29):38-41.

10. Barker R, Kober A, Hoerauf K, Latzke D, Adel S, Kain ZN, et al. Out-of-hospital auricular acupressure in elder patients with hip fracture: a randomized double-blinded trial. Academic emergency medicine. 2006;13(1):19-23.

11. Barssoum K, Kumar A, Thakkar S, Sheth AR, Kharsa A, Ibrahim M, et al. Meta-analysis of Safety and Efficacy of Anticoagulation versus no Anticoagulation in Octogenarians and Nonagenarians with Atrial Fibrillation. High Blood Pressure & Cardiovascular Prevention. 2021;28(3):271-82.

12. Bays-Moneo AB, Izquierdo M, Anton MM, Cadore EL. Cost-Consequences Analysis Following Different Exercise Interventions in Institutionalized Oldest Old: A Pilot Study of a Randomized Clinical Trial. The journal of nutrition, health & aging. 2023;27(11):1091-9.

13. Bechshoft RL, Malmgaard-Clausen NM, Gliese B, Beyer N, Mackey AL, Andersen JL, et al. Improved skeletal muscle mass and strength after heavy strength training in very old individuals. Experimental Gerontology. 2017;92:96-105.

14. Beckett N, Peters R, Tuomilehto J, Swift C, Sever PS, Potter JF, et al. Immediate and late benefits of treating very elderly people with hypertension: results from active treatment extension to Hypertension in the Very Elderly randomised controlled trial. BMJ (Clinical research ed). 2011;344(7839).

15. Beckett NS, Peters R, Fletcher AE, Staessen JA, Liu L, Dumitrascu D, et al. Treatment of hypertension in patients 80 years of age or older. New England journal of medicine. 2008;358(18):1887-98.

16. Behm L, Eklund K, Wilhelmson K, Ziden L, Gustafsson S, Falk K, et al. Health Promotion Can Postpone Frailty: Results from the RCT Elderly Persons in the Risk Zone. Public Health Nursing. 2016;33(4):303-15.

17. Behm L, Ivanoff SD, Ziden L. Preventive home visits and health--experiences among very old people. BMC Public Health. 2013;13:378.

18. Behm L, Wilhelmson K, Falk K, Eklund K, ZidÃ©n L, Dahlin-Ivanoff S. Positive health outcomes following health-promoting and disease-preventive interventions for independent very old persons: Long-term results of the three-armed RCT Elderly Persons in the Risk Zone. Archives of Gerontology & Geriatrics. 2014;58(3):376-83.

19. Berg ES, Tegn NK, Abdelnoor M, Roysland K, Ryalen PC, Aaberge L, et al. Long-Term Outcomes of Invasive vs Conservative Strategies for Older Patients With Non–ST-Segment Elevation Acute Coronary Syndromes. Journal of the American College of Cardiology. 2023;82(21):2021-30.

20. Berkhout M, Bengtsson Bostrom K, Ostberg A-L. Hypertension treatment in the oldest-old: focus group interviews with Swedish general practitioners. Scandinavian journal of primary health care. 2022;40(3):395-404.

21. Bhatnagar P, Sinha D, Parker RA, Guyler P, O'Brien A, Bhatnagar P, et al. Intravenous thrombolysis in acute ischaemic stroke: a systematic review and meta-analysis to aid decision making in patients over 80 years of age. Journal of Neurology, Neurosurgery & Psychiatry. 2011;82(7):712-7.

22. Biancari F, D'Errigo P, Rosato S, Pol M, Tamburino C, Ranucci M, et al. Transcatheter aortic valve replacement in nonagenarians: early and intermediate outcome from the OBSERVANT study and meta-analysis of the literature. Heart and Vessels. 2017;32(2).

23. Boonen S, McClung Michael R, Eastell R. Safety and efficacy of risedronate in reducing fracture risk in osteoporotic women aged 80 and older. Journal of the American Geriatrics Society. 2004;52(11).

24. Brettschneider C, Luck T, Fleischer S, Roling G, Beutner K, Luppa M, et al. Cost-utility analysis of a preventive home visit program for older adults in Germany. BMC health services research. 2015;15:141.

25. Briand M, Gerard S, Gauthier M, Garric M, Steinmeyer Z, Balardy L. Impact of therapeutic management and geriatric evaluation on patient of 80 years and older with diffuse large B-cell lymphoma on survival: a systematic review. European journal of haematology. 2022;108(1):3-17.

26. Bruunsgaard H, Bjerregaard E, Schroll M, Pedersen BK. Muscle strength after resistance training is inversely correlated with baseline levels of soluble tumor necrosis factor receptors in the oldest old. Journal of the American Geriatrics Society. 2004;52(2):237-41.

27. Bulpitt CJ, Beckett N, Peters R, Staessen JA, Wang JG, Comsa M, et al. Does white coat hypertension require treatment over age 80?: results of the hypertension in the very elderly trial ambulatory blood pressure side project. Hypertension (dallas, tex : 1979). 2013;61(1):89-94.

28. Bulpitt CJ, Beckett NS, Cooke J, Dumitrascu DL, Gil-Extremera B, Nachev C, et al. Results of the pilot study for the Hypertension in the Very Elderly Trial. Journal of Hypertension. 2003;21(12).

29. Bulpitt CJ, Beckett NS, Peters R, Leonetti G, Gergova V, Fagard R, et al. Blood pressure control in the Hypertension in the Very Elderly Trial (HYVET). Journal of human hypertension. 2012;26(3):157-63.

30. Buondonno I, Sassi F, Carignano G, Dutto F, Ferreri C, Pili FG, et al. From mitochondria to healthy aging: the role of branched-chain amino acids treatment: mATeR a randomized study. Clinical nutrition (Edinburgh, Scotland). 2020;39(7):2080-91.

31. Burden E, Pollock L, Paget C. Quality of life after in-hospital cardiopulmonary resuscitation for patients over the age of 80 years. Postgraduate Medical Journal. 2020;96(1134):186-9.

32. Burrows AB, Salzman C, Satlin A, Noble K, Pollock BG, Gersh T. A randomized, placebo-controlled trial of paroxetine in nursing home residents with non-major depression. Depression & Anxiety. 2002;15(3):102-10.

33. Burtin P, Bour B, Charlois T, Ruget O, Cales P, Dauver A, et al. Colonic investigations in the elderly: colonoscopy or barium enema? Aging-Clinical & Experimental Research. 1995;7(4):190-4.

34. Bynum JP, Barre L, Reed C, Passow H. Participation of very old adults in health care decisions. Medical Decision Making. 2014;34(2):216-30.

35. Cadore EL, Casas-Herrero A, Zambom-Ferraresi F, Idoate F, Millor N, Gomez M, et al. Multicomponent exercises including muscle power training enhance muscle mass, power output, and functional outcomes in institutionalized frail nonagenarians. Age. 2014;36(2):773-85.

36. Calder SJ, Anderson GH, Jagger C, Harper WM, Gregg PJ. Unipolar or bipolar prosthesis for displaced intracapsular hip fracture in octogenarians: a randomised prospective study. Journal of Bone & Joint Surgery - British Volume. 1996;78(3):391-4.

37. Campbell AJ, Robertson MC, Gardner Melinda M, Norton Robyn N, Buchner David M. Falls prevention over 2 years: A randomized controlled trial in women 80 years and older. Age and Ageing. 1999;28(6):513-8.

38. Campbell AJ, Robertson MC, Gardner MM, Norton RN, Tilyard MW, Buchner DM. Randomised controlled trial of a general practice programme of home based exercise to prevent falls in elderly women. Bmj-British Medical Journal. 1997;315(7115).

39. Campo-Prieto P, Cancela-Carral JM, Alsina-Rey B, Rodriguez-Fuentes G. Immersive Virtual Reality as a Novel Physical Therapy Approach for Nonagenarians: Usability and

Effects on Balance Outcomes of a Game-Based Exercise Program. Journal of Clinical Medicine. 2022;11(13):05.

40. Carral JMC, Rodriguez AL, Cardalda IM, Bezerra J. Muscle strength training program in nonagenarians - a randomized controlled trial. Revista Da Associacao Medica Brasileira. 2019;65(6):851-6.

41. Chammout G, Kelly-Pettersson P, Hedbeck CJ, Stark A, Mukka S, Skoldenberg O. HOPE-Trial: Hemiarthroplasty Compared with Total Hip Arthroplasty for Displaced Femoral Neck Fractures in Octogenarians: A Randomized Controlled Trial. JB & JS Open Access. 2019;4(2):e0059.

42. Chen B, Yu W, Ma Y, Xu P, Yao Q, Sun Q, et al. Evaluation of the safety and efficacy of perform enterectomy in colorectal cancer patients aged 80 or older. A meta-analysis and a systematic review. International journal of colorectal disease. 2023;38(1):185.

43. Chen Y-Y, Chen H, Song P. Promises and pitfalls of integrating home-based health services into Shanghai's elder-care system. Ageing & Society. 2020;40(3):480-500.

44. Clough AJ, Hilmer SN, Naismith SL, Gnjidic D. The Feasibility of Using N-Of-1 Trials to Investigate Deprescribing in Older Adults with Dementia: A Pilot Study. Healthcare. 2019;7(4):12.

45. Cofre-Bolados C, Vidal F, Gutierrez Espinoza H, Betancourt-Peters I, Orihuela PA, Izquierdo M. Periodized Aerobic Training between Thresholds Improves Submaximal Cardiorespiratory Parameters in Octogenarians. Sports (Basel, Switzerland). 2023;11(11).

46. Courage O, Strom L, van Rooij F, Lalevee M, Heuze D, Papin PE, et al. Higher rates of surgical and medical complications and mortality following TKA in patients aged >= 80 years: a systematic review of comparative studies. EFORT Open Reviews. 2021;6(11):1052-62.

47. de Belder A, de la Torre Hernandez JM, Lopez-Palop R, O'Kane P, Hernandez H, Strange J, et al. A prospective randomized trial of everolimus-eluting stents versus bare-metal stents in octogenarians: the XIMA Trial (Xience or Vision Stents for the Management of Angina in the Elderly). Journal of the American College of Cardiology. 2014;63(14):1371-5.

48. de Belder A, Myat A, Blaxill J, Haworth P, O'Kane P, Hatrick R, et al. Revascularisation or medical therapy in elderly patients with acute anginal syndromes: the RINCAL randomised trial. EuroIntervention : journal of EuroPCR in collaboration with the Working Group on Interventional Cardiology of the European Society of Cardiology. 2021;17(1).

49. DeBernardis DA, Zhang T, Duong A, Fleckenstein CM, Almasri M, Hasan SS. Total shoulder arthroplasty in patients aged 80 years and older: a systematic review. Journal of shoulder and elbow surgery. 2023;33(2).

50. Dodge HH, Zitzelberger T, Oken BS, Howieson D, Kaye J. A randomized placebo-controlled trial of Ginkgo biloba for the prevention of cognitive decline. Neurology. 2008;70(19 Pt 2):1809-17.

51. Duffis EJ, He W, Prestigiacomo CJ, Gandhi CD. Endovascular treatment for acute ischemic stroke in octogenarians compared with younger patients: a meta-analysis. International journal of stroke : official journal of the International Stroke Society. 2013;9(3).

52. Ehsani AA, Spina RJ, Peterson LR, Rinder MR, Glover KL, Villareal DT, et al. Attenuation of cardiovascular adaptations to exercise in frail octogenarians. Journal of Applied Physiology. 2003;95(5):1781-8.

53. Elahwal M, Richards T, Imsirovic A, Bagga R, Almond G, Yusuf SW. Systematic review of the results of fenestrated endovascular aortic repair in octogenarians. Annals of the Royal College of Surgeons of England. 2023.

54. Engelter ST, Bonati LH, Lyrer PA. Intravenous thrombolysis in stroke patients of ≥80 versus <80 years of age -: a systematic review across cohort studies. Age and Ageing. 2006;35(6).

55. Eriksen CS, Henkel C, Svensson RB, Agergaard AS, Couppe C, Kjaer M, et al. Lower tendon stiffness in very old compared with old individuals is unaffected by short-term resistance training of skeletal muscle. Journal of Applied Physiology. 2018;125(1):205-14.

56. Eriksen Kristina S, Eikeland Husebø Sissel I, Kørner H, Lode K. Experiences of recovery from colorectal cancer surgery after hospital discharge among the oldest old: A qualitative study. Nordic Journal of Nursing Research. 2021;41(3):140-8.

57. Ferrer A, Formiga F, Sanz H, de Vries Oscar J, Badia T, Pujol R. Multifactorial assessment and targeted intervention to reduce falls among the oldest-old: a randomized controlled trial. 2014;9:383-93.

58. Freixa X, Trilla M, Feldman M, Jimenez M, Betriu A, Masotti M. Right versus left transradial approach for coronary catheterization in octogenarian patients. Catheterization & Cardiovascular Interventions. 2012;80(2):267-72.

59. Fu M, Wang Z, Liu Y. Effects of Xinkeshu combined with levosimendan on perioperative heart failure in oldest-old patients with hip fractures. Journal of Traditional Chinese Medicine. 2020;40(5):870-4.

60. Gallingani A, D'Alessandro S, Singh G, Hernandez-Vaquero D, Celik M, Ceccato E, et al. The impact of coronary artery bypass grafting added to aortic valve replacement on long-term outcomes in octogenarian patients: a reconstructed time-to-event meta-analysis. Interactive Cardiovascular & Thoracic Surgery. 2022;35(2):09.

61. Ganske KM. The lived experience of and ethical issues involved in caring for octogenarian CABG patients at home 2002.

62. Gavazzi G, Drevet S, Debray M, Bosson JL, Tidadini F, Paccalin M, et al. Procalcitonin to reduce exposure to antibiotics and individualise treatment in hospitalised old patients with pneumonia: a randomised study. BMC geriatrics. 2022;22(1):965.

63. Gene Huguet L, Navarro Gonzalez M, Kostov B, Ortega Carmona M, Colungo Francia C, Carpallo Nieto M, et al. Pre Frail 80: multifactorial Intervention to Prevent Progression of Pre-Frailty to Frailty in the Elderly. Journal of nutrition, health & aging. 2018;22(10):1266-74.

64. Gillespie U, Alassaad A, Hammarlund-Udenaes M, Mörlin C, Henrohn D, Bertilsson M, et al. Effects of pharmacists' interventions on appropriateness of prescribing and evaluation of the instruments' (MAI, STOPP and STARTs') ability to predict hospitalization--analyses from a randomized controlled trial. PloS one. 2013;8(5):e62401.

65. Gillespie U, Alassaad A, Henrohn D, Garmo H, Hammarlund-Udenaes M, Toss H, et al. A comprehensive pharmacist intervention to reduce morbidity in patients 80 years or older: a randomized controlled trial. Archives of internal medicine. 2009;169(9):894-900.

66. Gine-Garriga M, Guerra M, Pages E, Manini TM, Jimenez R, Unnithan VB. The effect of functional circuit training on physical frailty in frail older adults: a randomized controlled trial. Journal of aging and physical activity. 2010;18(4):401-24.

67. Giné-Garriga M, Guerra M, Unnithan VB. The effect of functional circuit training on self-reported fear of falling and health status in a group of physically frail older individuals: a randomized controlled trial. Aging clinical and experimental research. 2013;25(3):329-36.

68. Godwin M, Gadag V, Pike A, Pitcher H, Parsons K, McCrate F, et al. A randomized controlled trial of the effect of an intensive 1-year care management program on measures of health status in independent, community-living old elderly: the Eldercare project. Family practice. 2016;33(1):37-41.

69. Greenspan SL, Fitzpatrick LA, Mitlak B, Wang Y, Harvey NC, Deal C, et al. Abaloparatide followed by alendronate in women ≥80 years with osteoporosis: post hoc analysis of ACTIVExtend. Menopause (New York, NY). 2020;27(10):1137-42.

70. Greuter L, Lutz K, Fandino J, Mariani L, Guzman R, Soleman J. Drain type after burr-hole drainage of chronic subdural hematoma in geriatric patients: a subanalysis of the cSDH-Drain randomized controlled trial. Neurosurgical Focus. 2020;49(4).

71. Gustafsson S, Eklund K, Wilhelmson K, Edberg A-K, Johansson B, Kronlof GH, et al. Long-term outcome for ADL following the health-promoting RCT--elderly persons in the risk zone. The Gerontologist. 2013;53(4):654-63.

72. Gustafsson S, Wilhelmson K, Eklund K, Gosman-Hedstrom G, Ziden L, Kronlof GH, et al. Health-promoting interventions for persons aged 80 and older are successful in the short term--results from the randomized and three-armed Elderly Persons in the Risk Zone study. Journal of the American Geriatrics Society. 2012;60(3):447-54.

73. Haanes GG, Kirkevold M, Hofoss D, Horgen G, Eilertsen G. An intervention designed to improve sensory impairments in the elderly and indoor lighting in their homes: an exploratory randomized controlled trial. Journal of multidisciplinary healthcare. 2015;8:11-20.

74. Haas LEM, van Dillen LS, de Lange DW, van Dijk D, Hamaker ME. Outcome of very old patients admitted to the ICU for sepsis: A systematic review. European Geriatric Medicine. 2017;8(5-6):446-53.

75. Hagiwara E, Suido Y, Asaoka M, Katano T, Okuda R, Sekine A, et al. Safety of pyrazinamide-including regimen in late elderly patients with pulmonary tuberculosis: a prospective randomized open-label study. Journal of infection and chemotherapy. 2019;25(12):1026-30.

76. Hajibandeh S, Hajibandeh S, Antoniou GA, Antoniou GA, Antoniou SA, Antoniou SA. Meta-analysis of mortality risk in octogenarians undergoing emergency general surgery operations. Surgery. 2021.

77. Han SJ, Lee TH, Park SH, Cho YS, Lee YN, Jung Y, et al. Efficacy of midazolam- versus propofol-based sedations by non-anesthesiologists during therapeutic endoscopic retrograde cholangiopancreatography in patients aged over 80 years. Digestive Endoscopy. 2017;29(3):369-76.

78. Hedbeck CJ, Blomfeldt R, Lapidus G, Törnkvist H, Ponzer S, Tidermark J. Unipolar hemiarthroplasty versus bipolar hemiarthroplasty in the most elderly patients with displaced femoral neck fractures: a randomised, controlled trial. International orthopaedics. 2011;35(11):1703-11.

79. Hilt PM, Bertrand MF, Feasson L, Lebon F, Mourey F, Ruffino C, et al. Motor Imagery Training Is Beneficial for Motor Memory of Upper and Lower Limb Tasks in Very Old Adults. International journal of environmental research and public health. 2023;20(4).

80. Hirlekar G, Libungan B, Karlsson T, Bäck M, Herlitz J, Albertsson P. Percutaneous coronary intervention in the very elderly with NSTE-ACS: the randomized 80+ study. Scandinavian cardiovascular journal : SCJ. 2020;54(5):315-21.

81. Holland R, Lenaghan E, Harvey I, Smith R, al e. Does home based medication review keep older people out of hospital? The HOMER randomised controlled trial: BMJ. British Medical Journal. 2005;330(7486):293-5.

82. Imhof L, Naef R, Wallhagen Margaret I, Schwarz J, Mahrer-Imhof R. Effects of an Advanced Practice Nurse In-Home Health Consultation Program for Community-Dwelling Persons Aged 80 and Older. Journal of the American Geriatrics Society. 2012;60(12):2223-31.

83. Instenes I, Fridlund B, Borregaard B, Larsen AI, Allore H, Bendz B, et al. 'When age is not a barrier': an explorative study of nonagenarian patients' experiences of undergoing percutaneous coronary intervention. European journal of cardiovascular nursing. 2024;NA(NA).

84. Iqbal U, Anwar H, Khan MA, Weissman S, Kothari ST, Kothari TH, et al. Safety and Efficacy of Endoscopic Retrograde Cholangiopancreatography in Nonagenarians: A Systematic Review and Meta-Analysis. Digestive diseases and sciences. 2022;67(4):1352-61.

85. Johnman C, Mackay DF, Oldroyd KG, Pell JP. Quality of life following percutaneous coronary interventions in octogenarians: a systematic review. Heart (British Cardiac Society). 2013;99(11):779-84.

86. Kalapotharakos VI, Diamantopoulos K, Tokmakidis SP. Effects of resistance training and detraining on muscle strength and functional performance of older adults aged 80 to 88 years. Aging-Clinical & Experimental Research. 2010;22(2):134-40.

87. Karlsen A, Bechshoft RL, Malmgaard-Clausen NM, Andersen JL, Schjerling P, Kjaer M, et al. Lack of muscle fibre hypertrophy, myonuclear addition, and satellite cell pool expansion with resistance training in 83-94-year-old men and women. Acta Physiologica. 2019;227(1):e13271.

88. Khatri K, Banga RK, Malhotra N, Bansal D. Cemented Calcar Replacement versus Long Stem Cemented Hemiarthroplasty in Unstable Intertrochanteric Fractures in Octogenarians. Revista Brasileira de Ortopedia. 2022;57(3):511-20.

89. Kim SY, Weinberg L, Christophi C, Nikfarjam M. The outcomes of the pancreaticoduodenectomy in patients aged 80 or older. A systematic review and meta-analysis. HPB : the official journal of the International Hepato Pancreato Biliary Association. 2017;19(6):475-82.

90. Kryger AI, Andersen JL. Resistance training in the oldest old: consequences for muscle strength, fiber types, fiber size, and MHC isoforms. Scandinavian Journal of Medicine & Science in Sports. 2007;17(4):422-30.

91. Kylberg M, Lofqvist C, Phillips J, Iwarsson S. Three very old men's experiences of mobility device use over time. Scandinavian Journal of Occupational Therapy. 2013;20(5):397-405.

92. Lee K-H, Chen Y-F, Yeh W-Y, Yeh J-T, Yang T-H, Chou C-Y, et al. Optimal stroke preventive strategy for patients aged 80 years or older with atrial fibrillation: a systematic review with traditional and network meta-analysis. Age and Ageing. 2022;51(12):1-10.

93. Lenaghan E, Holland R, Brooks A. Home-based medication review in a high risk elderly population in primary care--the POLYMED randomised controlled trial. Age & Ageing. 2007;36(3):292-7.

94. Liang H, Lu S, Jiang D, Fei Q. Clinical outcomes of lumbar spinal surgery in patients 80 years or older with lumbar stenosis or spondylolisthesis: a systematic review and meta-analysis. European Spine Journal. 2020;29(9):2129-42.

95. Liang Y, Wang R, Jiang J, Tan L, Yang M. A randomized controlled trial of resistance and balance exercise for sarcopenic patients aged 80-99 years. Scientific reports. 2020;10(1):18756.

96. Lin NH, Ho JSY, Djohan AH, Ho VW-T, Teo YN, Teo YH, et al. Percutaneous coronary intervention in patients aged 80 years old and above: a systematic review and meta-analysis. AsiaIntervention. 2022;8(2).

97. Lin YL, Chen LL, Luo YK, Zheng XC, Li WW. Benefit of standard versus low-dose tirofiban for percutaneous coronary intervention in very elderly patients with high-risk acute coronary syndrome. Acta pharmacologica Sinica. 2009;30(5):553-8.

98. Lofqvist C, Nygren C, Brandt A, Iwarsson S. Very old Swedish women's experiences of mobility devices in everyday occupation: a longitudinal case study. Scandinavian journal of occupational therapy. 2009;16(3):181-92.

99. Lopiz Y, Alcobía-Díaz B, Galán-Olleros M, García-Fernández C, Picado AL, Marco F. Reverse shoulder arthroplasty versus nonoperative treatment for 3- or 4-part proximal humeral fractures in elderly patients: a prospective randomized controlled trial. Journal of shoulder and elbow surgery. 2019;28(12):2259-71.

100. Lorenzano S, Vestri A, Lancia U, Bovi P, Cappellari M, Stanzione P, et al. Thrombolysis in elderly stroke patients in Italy (TESPI) trial and updated meta-analysis of randomized controlled trials. International Journal of Stroke. 2021;16(1):43-54.

101. Louvard Y, Benamer H, Garot P, Hildick-Smith D, Loubeyre C, Rigattieri S, et al. Comparison of transradial and transfemoral approaches for coronary angiography and angioplasty in octogenarians (the OCTOPLUS study). American journal of cardiology. 2004;94(9):1177-80.

102. Lu Q, Tang G, Zhao X, Guo S, Cai B, Li Q. Hemiarthroplasty versus internal fixation in super-aged patients with undisplaced femoral neck fractures: a 5-year follow-up of randomized controlled trial. Archives of orthopaedic and trauma surgery. 2017;137(1):27-35.

103. Luck T, Motzek T, Luppa M, Matschinger H, Fleischer S, Sesselmann Y, et al. Effectiveness of preventive home visits in reducing the risk of falls in old age: a randomized controlled trial. Clinical interventions in aging. 2013;8:697-702.

104. Luukinen H, Lehtola S, Jokelainen J, Väänänen-Sainio R, Lotvonen S, Koistinen P. Pragmatic exercise-oriented prevention of falls among the elderly: a population-based, randomized, controlled trial. Preventive medicine. 2007;44(3):265-71.

105. Malaguarnera M, Cammalleri L, Gargante MP, Vacante M, Colonna V, Motta M. L-Carnitine treatment reduces severity of physical and mental fatigue and increases cognitive functions in centenarians: a randomized and controlled clinical trial. American Journal of Clinical Nutrition. 2007;86(6):1738-44.

106. Marcellaud E, Jost J, Tchalla A, Magne J, Aboyans V. Statins in Primary Prevention in People Over 80 Years. The American journal of cardiology. 2023;187:62-73.

107. McClung MR, Harvey NC, Fitzpatrick LA, Miller PD, Hattersley G, Wang Y, et al. Effects of abaloparatide on bone mineral density and risk of fracture in postmenopausal women aged 80 years or older with osteoporosis. Menopause (New York, NY). 2018;25(7):767-71.

108. McCombie AM, Frampton CM, Frizelle FA. Quality of life preferences in colorectal cancer patients aged 80 and over. ANZ Journal of Surgery. 2021;91(9):1859-65.

109. McCord A, Cocks B, Barreiros Ana R, Bizo Lewis A. Short video game play improves executive function in the oldest old living in residential care. Computers in Human Behavior. 2020;108.

110. Mende E, Moeinnia N, Schaller N, Weis M, Haller B, Halle M, et al. Progressive machine-based resistance training for prevention and treatment of sarcopenia in the oldest old: A systematic review and meta-analysis. Experimental Gerontology. 2022;163:111767.

111. Meng R, Ding Y, Asmaro K, Brogan D, Meng L, Sui M, et al. Ischemic Conditioning Is Safe and Effective for Octo- and Nonagenarians in Stroke Prevention and Treatment. Neurotherapeutics. 2015;12(3):667-77.

112. Merga ZC, Lee JS, Gong CS. Outcomes of Gastrectomy for Gastric Cancer in Patients Aged >80 Years: A Systematic Literature Review and Meta-Analysis. Journal of Gastric Cancer. 2023;23(3):428-50.

113. Mikami T, Hirabayashi K, Okawa K, Betsuyaku T, Watanabe S, Imamura Y, et al. Laboratory Test Predictors for Major Bleeding in Elderly (≥80 Years) Patients With Nonvalvular Atrial Fibrillation Treated With Edoxaban 15 mg: Sub-Analysis of the ELDERCARE-AF Trial. Journal of the American Heart Association. 2022;11(17).

114. Minai K, Horie H, Takahashi M, Nozawa M, Kinoshita M. Long-term outcome of primary percutaneous transluminal coronary angioplasty for low-risk acute myocardial infarction in patients older than 80 years: a single-center, open, randomized trial. American Heart Journal. 2002;143(3):497-505.

115. Namkoong H, Funatsu Y, Oishi K, Akeda Y, Hiraoka R, Takeshita K, et al. Comparison of the immunogenicity and safety of polysaccharide and protein-conjugated pneumococcal vaccines among the elderly aged 80 years or older in Japan: an open-labeled randomized study. Vaccine. 2015;33(2):327-32.

116. Nana P, Spanos K, Brotis A, Fabre D, Mastracci T, Haulon S. Systematic Review on Early and Follow-up Mortality Rate in Octogenarians Treated With a Fenestrated and/or Branched Endovascular Aortic Repair. Journal of endovascular therapy : an official journal of the International Society of Endovascular Specialists. 2023:15266028231182798.

117. Nicolson PJA, Duong V, Williamson E, Hopewell S, Lamb SE. The Effect of Therapeutic Exercise Interventions on Physical and Psychosocial Outcomes in Adults Aged 80 Years and Older: A Systematic Review and Meta-Analysis. Journal of aging and physical activity. 2021;30(3).

118. Noguchi M, Ueyama H, Ando T, Takagi H, Toshiki K. Clinical outcomes in nonagenarians undergoing transcatheter aortic valve implantation: a systematic review and meta-analysis. Cardiovascular intervention and therapeutics. 2022;37(1):202-8.

119. Oberic L, Peyrade F, Puyade M, Bonnet C, Dartigues-Cuillères P, Fabiani B, et al. Subcutaneous Rituximab-MiniCHOP Compared With Subcutaneous Rituximab-MiniCHOP Plus Lenalidomide in Diffuse Large B-Cell Lymphoma for Patients Age 80 Years or Older. Journal of clinical oncology. 2021;39(11):1203-13.

120. Okazaki O, Higashino Y, Yokoya K, An Y, Tanizawa K, Imamura Y, et al. Prognosis of elderly non-valvular atrial fibrillation patients stratified by B-type natriuretic peptide: ELDERCARE-AF subanalysis. American heart journal. 2022;250(NA).

121. Okumura K, Akao M, Yoshida T, Kawata M, Okazaki O, Akashi S, et al. Low-Dose Edoxaban in Very Elderly Patients with Atrial Fibrillation. New England journal of medicine. 2020;383(18):1735-45.

122. Oliveira J, Mesquita-Bastos J, Argel de Melo C, Ribeiro F. Postaerobic Exercise Blood Pressure Reduction in Very Old Persons With Hypertension. Journal of Geriatric Physical Therapy. 2016;39(1):8-13.

123. Ouwehand AC, Bergsma N, Parhiala R, Lahtinen S, Gueimonde M, Finne-Soveri H, et al. Bifidobacterium microbiota and parameters of immune function in elderly subjects. FEMS immunology and medical microbiology. 2008;53(1):18-25.

124. Owen R, Berry K, Brown LJE. Enhancing Older Adults' Well-Being and Quality of Life Through Purposeful Activity: A Systematic Review of Intervention Studies. Gerontologist. 2022;62(6):e317-e27.

125. Pajewski NM, Berlowitz DR, Bress AP, Callahan KE, Cheung AK, Fine LJ, et al. Intensive vs Standard Blood Pressure Control in Adults 80 Years or Older: A Secondary Analysis of the Systolic Blood Pressure Intervention Trial. Journal of the American Geriatrics Society. 2019;68(3).

126. Pershad A, Fraij G, Massaro JM, David SW, Kleiman NS, Denktas AE, et al. Comparison of the use of hemodynamic support in patients >=80 years versus patients <80 years during high-risk percutaneous coronary interventions (from the Multicenter PROTECT II Randomized Study). The American journal of cardiology. 2014;114(5):657-64.

127. Peters R, Beckett N, Burch L, de Vernejoul MC, Liu L, Duggan J, et al. The effect of treatment based on a diuretic (indapamide) +/- ACE inhibitor (perindopril) on fractures in the Hypertension in the Very Elderly Trial (HYVET). Age and ageing. 2010;39(5):609-16.

128. Peters R, Beckett N, Forette F, Tuomilehto J, Clarke R, Ritchie C, et al. Incident dementia and blood pressure lowering in the Hypertension in the Very Elderly Trial cognitive

function assessment (HYVET-COG): a double-blind, placebo controlled trial. The lancet Neurology. 2008;7(8):683-9.

129. Peters R, Beckett N, Poulter R, Burch L, Narkiewicz K, Fagard R, et al. Kidney function in the very elderly with hypertension: data from the hypertension in the very elderly (HYVET) trial. Age and ageing. 2013;42(2):253-8.

130. Puggaard L, Larsen JB, Ebbesen E, Jeune B. Body composition in 85 year-old women: effects of increased physical activity. Aging-Clinical & Experimental Research. 1999;11(5):307-15.

131. Puggaard L, Larsen JB, Stovring H, Jeune B. Maximal oxygen uptake, muscle strength and walking speed in 85-year-old women: effects of increased physical activity. Aging-Clinical & Experimental Research. 2000;12(3):180-9.

132. Rash A, Downes T, Portner R, Yeo WW, Morgan N, Channer KS. A randomised controlled trial of warfarin versus aspirin for stroke prevention in octogenarians with atrial fibrillation (WASPO). Age & Ageing. 2007;36(2):151-6.

133. Rautalin I, Niemela M, Korja M. Is surgery justified for 80-year-old or older intracranial meningioma patients? A systematic review. Neurosurgical review. 2021;44(2):1061-9.

134. Rimon E, Kagansky N, Kagansky M, Mechnick L, Mashiah T, Namir M, et al. Are we giving too much iron? Low-dose iron therapy is effective in octogenarians. American Journal of Medicine. 2005;118(10):1142-7.

135. Rodrigues RN, Carballeira E, Silva F, Caldo-Silva A, Abreu C, Furtado GE, et al. The Effect of a Resistance Training, Detraining and Retraining Cycle on Postural Stability and Estimated Fall Risk in Institutionalized Older Persons: A 40-Week Intervention. Healthcare. 2022;10(5):22.

136. Rosado P, Lisón JF, Amer JJ, Benavent V, Camps. Effects of a program of exercise and electrotherapy on muscle strength in elderly octogenarian. Fisioterapia al día. 2008;4(1):24-35.

137. Rosie J, Taylor D. Sit-to-stand as home exercise for mobility-limited adults over 80 years of age--GrandStand System may keep you standing? Age & Ageing. 2007;36(5):555-62.

138. Ruiz JR, Gil-Bea F, Bustamante-Ara N, Rodriguez-Romo G, Fiuza-Luces C, Serra-Rexach JA, et al. Resistance training does not have an effect on cognition or related serum biomarkers in nonagenarians: a randomized controlled trial. International Journal of Sports Medicine. 2015;36(1):54-60.

139. Salter C. "I haven't even phoned my doctor yet." The advice giving role of the pharmacist during consultations for medication review with patients aged 80 or more: qualitative discourse analysis. British Medical Journal. 2007;334(7603).

140. Schonberg MA, Ramanan RA, McCarthy EP, Marcantonio ER. Decision making and counseling around mammography screening for women aged 80 or older. Journal of General Internal Medicine. 2006;21(9):979-85.

141. Seeman E, Boonen S, Borgstrom F, Vellas B, Aquino JP, Semler J, et al. Five years treatment with strontium ranelate reduces vertebral and nonvertebral fractures and increases

the number and quality of remaining life-years in women over 80 years of age. Bone. 2010;46(4):1038-42.

142. Seeman E, Vellas B, Benhamou C, Aquino JP, Semler J, Kaufman JM, et al. Strontium ranelate reduces the risk of vertebral and nonvertebral fractures in women eighty years of age and older. Journal of bone and mineral research. 2006;21(7):1113-20.

143. Seinela L, Pehkonen E, Laasanen T, Ahvenainen J. Bowel preparation for colonoscopy in very old patients: a randomized prospective trial comparing oral sodium phosphate and polyethylene glycol electrolyte lavage solution. Scandinavian Journal of Gastroenterology. 2003;38(2):216-20.

144. Serra-Rexach JA, Bustamante-Ara N, Hierro V, González G, Sanz I, M J, et al. Short-term, light- to moderate-intensity exercise training improves leg muscle strength in the oldest old: a randomized controlled trial. Journal of the American Geriatrics Society. 2011;59(4):594-602.

145. Shantsila E, Lip G, Shantsila A, Maden M, Hill R, Beevers G. Pharmacotherapy for hypertension in very old people with frailty: A systematic review. NA. 2023;NA(NA).

146. Sheppard JP, Burt J, Lown M, Temple E, Lowe R, Fraser R, et al. Effect of Antihypertensive Medication Reduction vs Usual Care on Short-term Blood Pressure Control in Patients With Hypertension Aged 80 Years and Older: the OPTIMISE Randomized Clinical Trial. JAMA. 2020;323(20):2039-51.

147. Sun L, Zhou M, Ji Y, Wang X, Wang X. Off-pump versus on-pump coronary artery bypass grafting for octogenarians: A meta-analysis involving 146 372 patients. Clinical cardiology. 2022.

148. Tegn N, Abdelnoor M, Aaberge L, Endresen K, Smith P, Aakhus S, et al. Invasive versus conservative strategy in patients aged 80 years or older with non-ST-elevation myocardial infarction or unstable angina pectoris (After Eighty study): an open-label randomised controlled trial. Lancet (london, england). 2016;387(10023):1057-65.

149. Tegn N, Abdelnoor M, Aaberge L, Hylen Ranhoff A, Endresen K, Gjertsen E, et al. Health-related quality of life in older patients with acute coronary syndrome randomised to an invasive or conservative strategy. The After Eighty randomised controlled trial. Age and ageing. 2018;47(1):42-7.

150. Tegn N, Eek C, Abdelnoor M, Aaberge L, Endresen K, Skårdal R, et al. Patients aged 80 years or older with non-ST-elevation myocardial infarction or unstable angina pectoris randomised to an invasive versus conservative strategy: angiographic and procedural results from the After Eighty study. Open heart. 2020;7(2).

151. Thommessen B, Næss H, Logallo N, Kvistad CE, Waje-Andreassen U, Ihle-Hansen H, et al. Tenecteplase versus alteplase after acute ischemic stroke at high age. International journal of stroke. 2021;16(3):295-9.

152. Tomsone S, Haak M, Lofqvist C. Experiences of mobility device use over time: A multiple case study among very old Latvian women. Scandinavian Journal of Occupational Therapy. 2016;23(1):67-78.

153. van Halsema MS, Boers RAR, Leferink VJM. An overview on the treatment and outcome factors of ankle fractures in elderly men and women aged 80 and over: a systematic review. Archives of orthopaedic and trauma surgery. 2021.

154. Vorilhon C, Jean F, Mulliez A, Clerfond G, Pereira B, Sapin V, et al. Optimized management of heart failure patients aged 80 years or more improves outcomes versus usual care: the HF80 randomized trial. Archives of cardiovascular diseases. 2016;109(12):667-78.

155. Wang G, Sun Y, Lin Z, Fei X. Elective Endovascular vs Open Repair for Elective Abdominal Aortic Aneurysm in Patients >=80 years of Age: A Systematic Review and Meta-Analysis. Vascular and endovascular surgery. 2023;57(4):386-401.

156. Wang QX, Zhang XY, Li Q. Effects of a flutter mucus-clearance device on pulmonary function test results in healthy people 85 years and older in China. Respiratory care. 2010;55(11):1449-52.

157. Warwick J, Falaschetti E, Rockwood K, Mitnitski A, Thijs L, Beckett N, et al. No evidence that frailty modifies the positive impact of antihypertensive treatment in very elderly people: an investigation of the impact of frailty upon treatment effect in the HYpertension in the Very Elderly Trial (HYVET) study, a double-blind, placebo-controlled study of antihypertensives in people with hypertension aged 80 and over. BMC medicine. 2015;13:78.

158. Watkins AR, Fialka N, El-Andari R, Kang JJH, Bozso SJ, Moon MC, et al. Mortality and morbidity of surgical and transcatheter mitral valve repair in octogenarians: A systematic review. Journal of Cardiac Surgery. 2022;37(9):2752-60.

159. West RK. Computerized Cognitive Intervention in Cognitively Normal Very Elderly Individuals 2016.

160. West RK, Rabin LA, Silverman JM, Moshier E, Sano M, Beeri MS. Short-term computerized cognitive training does not improve cognition compared to an active control in non-demented adults aged 80 years and above. International Psychogeriatrics. 2020;32(1):65-73.

161. Wilhelmson K, Eklund K. Positive effects on life satisfaction following health-promoting interventions for frail older adults: a randomized controlled study. Health psychology research. 2013;1(1).

162. Wising J, Mattsson G, Rambaree K, Willmer M, Wallhagen M, Magnusson P. 'Life with a device': the octogenarians' experiences with an implantable cardioverter-defibrillator-a qualitative study. European Journal of Cardiovascular Nursing. 2022;21(2):161-8.

163. Wubbeke LF, Naves C, Daemen J, Jacobs MJ, Mees BME. Mortality and Major Amputation after Revascularisation in Octogenarians Versus Non-Octogenarians with Chronic Limb Threatening Ischaemia: A Systematic Review and Meta-Analysis. European Journal of Vascular and Endovascular Surgery. 2020;60(2):231-41.

164. Xu Y, Bouliotis G, Beckett NS, Antikainen RL, Anderson CS, Bulpitt CJ, et al. Left ventricular hypertrophy and incident cognitive decline in older adults with hypertension. Journal of human hypertension. 2022;37(4).

165. Xu Y, Wang Y, Xi C, Ye N, Xu X, Yang F. Is it safe to perform gastrectomy in gastric cancer patients aged 80 or older?: A meta-analysis and systematic review. Medicine. 2019;98(24):e16092-e.

166. Yamashita K, Fukusaki M, Ando Y, Tanabe T, Terao Y, Sumikawa K. Postoperative analgesia with minidose intrathecal morphine for bipolar hip prosthesis in extremely elderly patients. Journal of anesthesia. 2009;23(4):504-7.

167. Yamashita T, Igawa Y, Fukuzawa M, Hayashi T, Hennig S, Okumura K. Pharmacokinetics of Edoxaban 15 mg in Very Elderly Patients with Nonvalvular Atrial Fibrillation: A Subanalysis of the ELDERCARE-AF Study. Thrombosis and haemostasis. 2024;NA(NA).

168. Yan L, Yu D, Mingjie F, YueMa, DeguangWang, Jinglin Z, et al. Clinical Outcomes of Transcatheter Aortic Valve Replacement in Nonagenarians: A Systematic Review and Meta-Analysis. Journal of Interventional Cardiology. 2019:1-10.

169. Yoshida T, Nakamura A, Funada J, Amino M, Shimizu W, Fukuzawa M, et al. Efficacy and Safety of Edoxaban 15 mg According to Renal Function in Very Elderly Patients With Atrial Fibrillation: a Subanalysis of the ELDERCARE-AF Trial. Circulation. 2022;145(9):718-20.

170. Zak M, Gryglewska B. Application of two structured rehabilitation regimens in the frail octogenarians (over 85) with functional disorders. Rehabilitacja medyczna. 2006;10(2):20-4.

171. Zhou J, Liu B, Qin MZ, Liu JP. Fall Prevention and Anti-Osteoporosis in Osteopenia Patients of 80 Years of Age and Older: a Randomized Controlled Study. Orthopaedic surgery. 2020;12(3):890-9.

172. Zidén L, Häggblom-Kronlöf G, Gustafsson S, Lundin-Olsson L, Dahlin-Ivanoff S. Physical Function and Fear of Falling 2 Years After the Health-Promoting Randomized Controlled Trial: Elderly Persons in the Risk Zone. Gerontologist. 2014;54(3):387-97.

APPENDIX (5) Table of excluded studies

REASON FOR EXCLUSION	Reference
**Wrong Study type** (n=32)	Blandfort S, Gregersen M, Borris LC, Damsgaard EM. Blood transfusion strategy and risk of postoperative delirium in nursing homes residents with hip fracture. A post hoc analysis based on the TRIFE randomized controlled trial. Aging clinical and experimental research. 2017;29(3):459-66.
	Bleijenberg N, Imhof L, Mahrer-Imhof R, Wallhagen MI, Wit NJ, Schuurmans MJ. Patient Characteristics Associated With a Successful Response to Nurse-Led Care Programs Targeting the Oldest-Old: A Comparison of Two RCTs. Worldviews on Evidence-Based Nursing. 2017;14(3):210-22.
	Bohers E, Viailly PJ, Ruminy P, Marchand V, Viennot M, Bobee V, et al. Molecular characterisation of diffuse large B cell lymphoma in patients of 80 years old or more: clinical relevance in a multicentric randomized phase III study of the LYSA (senior study). Blood. 2019;134.
	Boonen S, McClung MR, Eastell R. Safety and efficacy of risedronate in reducing fracture risk in osteoporotic women aged 80 and older. Journal of the American Geriatrics Society. 2004;52(11).
	Briand M, Gerard S, Gauthier M, Garric M, Steinmeyer Z, Balardy L. Impact of therapeutic management and geriatric evaluation on patient of eighty years and older with diffuse large B-cell lymphoma on survival: A systematic review. European Journal of Haematology. 2022;108(1):3-17.
	de la Sierra A, Sierra C, Murillo M, Aiello TF, Mateu A, Almagro P. Pulse Wave Velocity and Blood Pressure Variability as Prognostic Indicators in Very Elderly Patients. Journal of Clinical Medicine. 2023;12(4).
	Ding Y, Si QJ. Lipid-lowering efficacy and safety of policosanol on cardiovascular diseases in the very old patients. Journal of the American Geriatrics Society. 2015;63:S382.
	Douros A, Basedow F, Cui Y, Dimakos J, Walker J, Enders D, et al. Effectiveness and safety of direct oral anticoagulants among octogenarians with venous thromboembolism: an international multi-database cohort study. American Journal of Medicine. 2022;20:20.
	Fallah J, Zhang L, Amatya A, Gong Y, King-Kallimanis B, Bhatnagar V, et al. Survival outcomes in older men with non-metastatic castration-resistant prostate cancer treated with androgen receptor inhibitors: a US Food and Drug Administration pooled analysis of patient-level data from three randomised trials. The Lancet Oncology. 2021;22(9):1230-9.
	Fisher A, Davis M. Efficacy of antithrombotic therapy for atrial fibrillation in the oldest old. Journal of the American Geriatrics Society. 2003;51(6):887-9.
	Gokcal F, Morrison S, Kudsi OY. Robotic ventral hernia repair in octogenarians: perioperative and long-term outcomes. Journal of Robotic Surgery. 2020;14(2):275-81.
	Gregersen M, Borris LC, Damsgaard EM. A liberal blood transfusion strategy after hip fracture surgery does not increase the risk of infection in frail elderly. European geriatric medicine. 2012;3:S74.
	Guhne U, Luppa M, Konig HH, Hautzinger M, Riedel-Heller S. [Are psychotherapeutic interventions effective in late-life depression? a systematic review]. Psychiatrische Praxis. 2014;41(8):415-23.
	Han Y, Zhang S, Zhang J, Ji C, Eckstein HH. Outcomes of Endovascular Abdominal Aortic Aneurysm Repair in Octogenarians: Meta-analysis and Systematic Review. European Journal of Vascular & Endovascular Surgery. 2017;54(4):454-63.
	Hanna L, Lam K, Jha R, Abdullah A, Sounderajah V, Antoniou G, et al. Outcomes following fenestrated and branched endovascular repair (F/BEVAR) of complex aneurysms in octogenarians: a time to event meta-analysis. British Journal of Surgery. 2023;110:III18-III.

	Johnman C. Quality of life following percutaneous coronary interventions in octogenarians : a systematic review. Heart. 2013.
	Kitridis D, Tsikopoulos K, Givissis P, Chalidis B. Mortality and complication rates in nonagenarians and octogenarians undergoing total hip and knee arthroplasty: a systematic review and meta-analysis. European Geriatric Medicine. 2022;13(3):725-33.
	Kuo H-K, Scandrett KG, Dave J, Mitchell SL. The influence of outpatient comprehensive geriatric assessment on survival: a meta-analysis. Archives of gerontology and geriatrics. 2004;39(3):245-54.
	Le Quintrec JL, Maheu E, Verlhac B, Cadet C, Breville P, Vetel JM, et al. Management of lower limb osteoarthritis: Are physical exercises and weight reduction efficient in very old patients? Cahiers de l'Annee Gerontologique. 2015;7(2):66-73.
	Lin AY, Lupercio F, Ho G, Pollema T, Pretorius V, Birgersdotter-Green U. Safety and Efficacy of Cardiovascular Implantable Electronic Device Extraction in Elderly Patients: A Meta-Analysis and Systematic Review. Heart Rhythm O2. 2020;1(4):250-8.
	Mukhtar O, Jackson SH. The Hypertension in the Very Elderly Trial - latest data. British Journal of Clinical Pharmacology. 2013;75(4):951-4.
	Ploumen EH, Buiten RA, Doggen CJM, Stoel MG, van Houwelingen KG, Schotborgh CE, et al. New-generation drug-eluting coronary stents in octogenarians: Patient-level pooled analysis from the TWENTE I-IV trials. American Heart Journal. 2020;228:109-15.
	Sim J, Lim J, Lee H, Shin D, Park S. A 12-Week Nutrition and Physical Activity Program Improves Toe Strength Among Elderly Day Care Center Users in Korea: a Cluster Randomized Controlled Trial. Current developments in nutrition. 2023;7.
	Simon F, Ligtvoet R, Nösslinger T, Bohn J, von Tresckow J, Liersch R, et al. Safety of acalabrutinib treatment in very old (≥80 y) and/or frail patients with chronic lymphocytic leukemia - interim safety analysis of the ongoing phase II CLL-Frail trial. Hematological Oncology. 2023;41:468-9.
	Straatman J, Groen LCB, van der Wielen N, Jansma EP, Daams F, Cuesta MA, et al. Treatment of paraesophageal hiatal hernia in octogenarians: a systematic review and retrospective cohort study. Diseases of the Esophagus. 2018;31(7):01.
	Sun L, Zhou M, Ji Y, Wang X, Wang X. Off-pump versus on-pump coronary artery bypass grafting for octogenarians: A meta-analysis involving 146 372 patients. Clinical Cardiology. 2022;45(4):331-41.
	Sundstrom M, Petersson P, Ramgard M, Varland L, Blomqvist K. Health and social care planning in collaboration in older persons' homes: the perspectives of older persons, family members and professionals. Scandinavian journal of caring sciences. 2018;32(1):147-56.
	Texakalidis P, Chaitidis N, Giannopoulos S, Giannopoulos S, Machinis T, Jabbour P, et al. Carotid Revascularization in Older Adults: A Systematic Review and Meta-Analysis. World Neurosurgery. 2019;126:656-+.
	Thompson W, Pottegård A, Nielsen JB, Haastrup P, Jarbøl DE. How Common is Statin Use in the Oldest Old? Drugs & Aging. 2018;35(8):679-86.
	Van der Elst M, Schoenmakers B, Duppen D, Lambotte D, Fret B, Vaes B, et al. Interventions for frail community-dwelling older adults have no significant effect on adverse outcomes: a systematic review and meta-analysis. BMC Geriatrics. 2018;18(249).
	Van Der Vlegel W, Duckworth J, Partington H, de Jong A. Evaluating a transitional care program for the average and the oldest old: results from the qualitative phase of a mixed methods study...22nd International Conference on Integrated Care, May 23-25, 2022, Odense, Denmark. International Journal of Integrated Care (IJIC). 2022;22:1-2.
	Xandri JM, Muxella EH, Anzano AP, Ulloa E, Anglada M. How the VIVIFRAIL © Multi-Component Exercise Program can improve the functional capacity of older patients admitted to a functional recovery unit. European geriatric

	medicine. 2022;13:S312.
**Protocol** (n=2)	Brunner-La Rocca HP, Buser PT, Schindler R, Bernheim A, Rickenbacher P, Pfisterer M. Management of elderly patients with congestive heart failure--design of the Trial of Intensified versus standard Medical therapy in Elderly patients with Congestive Heart Failure (TIME-CHF). American heart journal. 2006;151(5):949-55.
	Rexach JA, Ruiz JR, Bustamante-Ara N, Villarán MH, Gil PG, Sanz Ibáñez MJ, et al. Health enhancing strength training in nonagenarians (STRONG): rationale, design and methods. BMC Public Health. 2009;9(1):152-.
**Excluded on intervention** (n=21)	Comprehensive care: older people living with frailty in hospitals. 2017.
	Aggar C, Ronaldson S, Cameron ID. Reactions to caregiving during an intervention targeting frailty in community living older people. BMC geriatrics. 2012;12:66.
	Arain MA, Graham L, Ahmad A, Cole M. Pilot implementation of elder-friendly care practices in acute care setting: a mixed methods study. BMC health services research. 2020;20(1):347.
	Barker RJ, Wilson P, Butler C. How can the priorities of older, frail patients and their carers be used to inform policy and practice at the end of life?: insights from qualitative research across multiple settings. BMJ Open. 2023;13(3).
	Blomaard LC, Olthof M, Meuleman Y, de Groot B, Gussekloo J, Mooijaart SP. Experiences with and attitudes towards geriatric screening among older emergency department patients: a qualitative study. BMC geriatrics. 2021;21(1):198.
	Bogaerts JMK, von Ballmoos LM, Achterberg WP, Gussekloo J, Streit S, van der Ploeg MA, et al. Do we AGREE on the targets of antihypertensive drug treatment in older adults: a systematic review of guidelines on primary prevention of cardiovascular diseases. Age and ageing. 2021.
	Cheng A, Leung Y, Brodaty H. A systematic review of the associations, mediators and moderators of life satisfaction, positive affect and happiness in near-centenarians and centenarians. Aging Ment Health. 2022;26(4):651-66.
	Condelius A, Andersson M. Exploring access to care among older people in the last phase of life using the behavioural model of health services use: a qualitative study from the perspective of the next of kin of older persons who had died in a nursing home. Bmc Geriatrics. 2015;15.
	Coughlan R, Ward L. Experiences of recently relocated residents of a long-term care facility in Ontario: assessing quality qualitatively. International Journal of Nursing Studies. 2007;44(1):47-57.
	Gwyther H, Shaw R, Dauden E-AJ, D'Avanzo B, Kurpas D, Bujnowska-Fedak M, et al. Understanding frailty: a qualitative study of European healthcare policy-makers' approaches to frailty screening and management. BMJ Open. 2018;8(1).
	Instenes I, Fridlund B, Amofah HA, Ranhoff AH, Eide LS, Norekval TM. 'I hope you get normal again': an explorative study on how delirious octogenarian patients experience their interactions with healthcare professionals and relatives after aortic valve therapy. European Journal of Cardiovascular Nursing. 2019;18(3):224-33.
	Instenes I, Gjengedal E, Eide LSP, Kuiper KKJ, Ranhoff AH, Norekvaal TM. Experiences of post-operative delirium in octogenarian patients after transcatheter aortic valve implantation or surgical aortic valve replacement - a qualitative interview study. European Journal of Cardiovascular Nursing. 2015;14:S43-S4.
	Instenes I, Gjengedal E, Eide LSp, Kuiper KKj, Ranhoff AH, Norekvål TM. "Eight Days of Nightmares … " - Octogenarian Patients' Experiences of Postoperative Delirium after Transcatheter or Surgical Aortic Valve Replacement. Heart, Lung & Circulation. 2018;27(2):260-6.

	Looman WM. Facing frailty: exploring the effectiveness of integrated care for frail older people. International Journal of Integrated Care. 2019;19(3):13.
	Pettersson E, Vernby A, Mölstad S, Lundborg CS. Can a multifaceted educational intervention targeting both nurses and physicians change the prescribing of antibiotics to nursing home residents? A cluster randomized controlled trial. Journal of antimicrobial chemotherapy. 2011;66(11):2659-66.
	Pohontsch NJ, Loffler A, Luck T, Heser K, Parker D, Haenisch B, et al. Informal caregivers' perspectives on health of and (potentially inappropriate) medication for (relatively) independent oldest-old people - a qualitative interview study. BMC Geriatrics. 2018;18(1):169.
	Scheibl F, Farquhar M, Buck J, Barclay S, Brayne C, Fleming J. When Frail Older People Relocate in Very Old Age, Who Makes the Decision? Innovation in Aging. 2019;3(4):igz030.
	Scheibl F, Fleming J, Buck J, Barclay S, Brayne C, Farquhar M. The experience of transitions in care in very old age: implications for general practice. Family Practice. 2019;36(6):778-84.
	Toye F, Jenkins C, Barker K. Understanding the experience of living well, beyond the age of 85 years: a qualitative analysis using themes from a meta-ethnography. Age and Ageing. 2021;50(6):2238-45.
	Underwood F, Burrows L, Gegg R, Latour JM, Kent B. The meaning of confidence for older people living with frailty: a qualitative systematic review. JBI Database Of Systematic Reviews And Implementation Reports. 2017;15(5):1316-49.
	Zhu X, Liu Z, Shen J, Liu J, Tang R. Comparison of open and laparoscopic inguinal-hernia repair in octogenarians. Asian J Surg. 2023;46(2):738-41.
**Insufficient methods** (n=76)	Abah U, Dunne M, Cook A, Hoole S, Brayne C, Vale L, et al. Does quality of life improve in octogenarians following cardiac surgery? A systematic review. BMJ Open. 2015;5(4):e006904.
	Abusnina W, Alam M, Dahal K. Meta-Analysis Comparing Outcomes of Invasive Versus Conservative Strategy in Octogenarians With Non-ST Segment Elevation Acute Myocardial Infarction. American Journal of Cardiology. 2021;160:130-2.
	Ahsan MJ, Fazeel HM, Malik SU, Din ATU, Hassan S, Khan BA, et al. Safety and Effectiveness of Trans-Catheter versus Surgical Aortic Valve Replacement in Nonagenarians With Severe Aortic Stenosis: A Systematic Review and Meta-Analysis. Circulation. 2019;140.
	Aitken SJ, Naganathan V, Blyth FM. Aortic aneurysm trials in octogenarians: Are we really measuring the outcomes that matter? Vascular. 2016;24(4):435-45.
	Altarabsheh SE, Deo SV, Rababa'h AM, Lim JY, Cho YH, Sharma V, et al. Off-pump coronary artery bypass reduces early stroke in octogenarians: a meta-analysis of 18,000 patients. Annals of Thoracic Surgery. 2015;99(5):1568-75.
	AlTurki A, Proietti R, Alturki H, Essebag V, Huynh T. Implantable cardioverter-defibrillator use in elderly patients receiving cardiac resynchronization: A meta-analysis. Hellenic Journal of Cardiology. 2019;60(5):276-81.
	Andalib A, Mamane S, Schiller I, Zakem A, Mylotte D, Martucci G, et al. A systematic review and meta-analysis of surgical outcomes following mitral valve surgery in octogenarians: implications for transcatheter mitral valve interventions. Eurointervention. 2014;9(10):1225-34.
	Antoniou GA, Georgiadis GS, Georgakarakos EI, Antoniou SA, Bessias N, Smyth JV, et al. Meta-analysis and Meta-Regression Analysis of Outcomes of Carotid Endarterectomy and Stenting in the Elderly. Jama Surgery. 2013;148(12):1140-52.
	Baert V, Gorus E, Mets T, Geerts C, Bautmans I. Motivators and barriers for physical activity in the oldest old: a systematic review. Ageing Research Reviews. 2011;10(4):464-74.

	Barlow J. A systematic review of the benefits of home telecare for frail elderly people and those with long-term conditions. Journal of Telemedicine and Telecare. 2007;13(4):172-9.
	Bauman A, Merom D, Bull FC, Buchner DM, Singh MAF. Updating the Evidence for Physical Activity: Summative Reviews of the Epidemiological Evidence, Prevalence, and Interventions to Promote "Active Aging". Gerontologist. 2016;56:S268-S80.
	Bejan-Angoulvant T, Saadatian-Elahi M, Wright JM, Schron EB, Lindholm LH, Fagard R, et al. Treatment of hypertension in patients 80 years and older: the lower the better? A meta-analysis of randomized controlled trials. Journal of Hypertension. 2010;28(7):1366-72.
	Benetos A, Rossignol P, Cherubini A, Joly L, Grodzicki T, Rajkumar C, et al. Polypharmacy in the Aging Patient: Management of Hypertension in Octogenarians. JAMA. 2015;314(2):170-80.
	Bhatnagar P, Sinha D, Parker RA, Guyler P, O'Brien A. Intravenous thrombolysis in acute ischaemic stroke: a systematic review and meta-analysis to aid decision making in patients over 80 years of age. Journal of Neurology, Neurosurgery & Psychiatry. 2011;82(7):712-7.
	Biancari F, D'Errigo P, Rosato S, Pol M, Tamburino C, Ranucci M, et al. Transcatheter aortic valve replacement in nonagenarians: early and intermediate outcome from the OBSERVANT study and meta-analysis of the literature. Heart & Vessels. 2017;32(2):157-65.
	Biancari F, Mazziotti MA, Paone R, Laukontaus S, Venermo M, Lepantalo M. Outcome after Open Repair of Ruptured Abdominal Aortic Aneurysm in Patients > 80 Years Old: A Systematic Review and Meta-analysis. World Journal of Surgery. 2011;35(7):1662-70.
	Biancari F, Schifano P, Pighi M, Vasques F, Juvonen T, Vinco G. Pooled estimates of immediate and late outcome of mitral valve surgery in octogenarians: a meta-analysis and meta-regression. Journal of Cardiothoracic & Vascular Anesthesia. 2013;27(2):213-9.
	Biancari F, Vasques F, Benenati V, Juvonen T. Contemporary results after surgical repair of type A aortic dissection in patients aged 80 years and older: a systematic review and meta-analysis. European Journal of Cardio-Thoracic Surgery. 2011;40(5):1058-63.
	Biondi Zoccai G, Abbate A, D'Ascenzo F, Presutti D, Peruzzi M, Cavarretta E, et al. Percutaneous coronary intervention in nonagenarians: pros and cons. Journal of Geriatric Cardiology. 2013;10(1):82-90.
	Bonanad C, Garcia-Blas S, Llergo JT, Fernandez-Olmo R, Diez-Villanueva P, Ariza-Sole A, et al. Direct Oral Anticoagulants versus Warfarin in Octogenarians with Nonvalvular Atrial Fibrillation: A Systematic Review and Meta-Analysis. Journal of Clinical Medicine. 2021;10(22).
	Cameron ID. Coordinated multidisciplinary rehabilitation after hip fracture. Disability and Rehabilitation. 2005;27(18).
	D'Alessandro S, Tuttolomondo D, Singh G, Hernandez-Vaquero D, Pattuzzi C, Gallingani A, et al. The early and long-term outcomes of coronary artery bypass grafting added to aortic valve replacement compared to isolated aortic valve replacement in elderly patients: a systematic review and meta-analysis. Heart and Vessels. 2022;37(10):1647-61.
	Day LW, Kwon A, Inadomi JM, Walter LC, Somsouk M. Adverse events in older patients undergoing colonoscopy: a systematic review and meta-analysis. Gastrointestinal Endoscopy. 2011;74(4):885-96.
	Devoto L, Celentano V, Cohen R, Khan J, Chand M. Colorectal cancer surgery in the very elderly patient: a systematic review of laparoscopic versus open colorectal resection. International Journal of Colorectal Disease. 2017;32(9):1237-42.
	Dong SY, Roberts SA, Chen S, Zhong XW, Yang SZ, Qu XH, et al. Survival after lobectomy versus sub-lobar resection in elderly with stage I NSCLC: a meta-analysis. Bmc Surgery. 2019;19.

	Eklund K, Wilhelmson K. Outcomes of coordinated and integrated interventions targeting frail elderly people: a systematic review of randomised controlled trials. Health and Social Care in the Community. 2009;17(5):447-58.
	Eranki A, Merakis M, Williams ML, Flynn CD, Villanueva C, Wilson-Smith A, et al. Outcomes of surgery for acute type A dissection in octogenarians versus non-octogenarians: a systematic review and meta analysis. Journal Of Cardiothoracic Surgery. 2022;17(1):222.
	Gacci M, Novara G, De Nunzio C, Tubaro A, Schiavina R, Brunocilla E, et al. Tolterodine extended release in the treatment of male OAB/storage LUTS: a systematic review. BMC Urology. 2014;14:84.
	Gao L, Hu X, Liu YQ, Xue Q, Wang Y. Comparison of coronary DES and BMS in octogenarians: A systematic review and meta-analysis. Journal of Geriatric Cardiology. 2013;10(4):336-43.
	Grgic J, Garofolini A, Orazem J, Sabol F, Schoenfeld BJ, Pedisic Z. Effects of Resistance Training on Muscle Size and Strength in Very Elderly Adults: A Systematic Review and Meta-Analysis of Randomized Controlled Trials. Sports Medicine. 2020;50(11):1983-99.
	Gueyffier F, Bulpitt C, Boissel J, Schron E, Ekbom T, Fagard R, et al. Antihypertensive drugs in very old people: a subgroup meta-analysis of randomised controlled trials. INDANA Group. Lancet. 1999;353(9155):793-6.
	He PY, Yang YJ, Hu FH. Transradial versus transfemoral percutaneous coronary intervention in elderly patients: a systematic overview and meta-analysis. Chinese Medical Journal. 2014;127(6):1110-7.
	Henebiens M, Vahl A, Koelemay MJ. Elective surgery of abdominal aortic aneurysms in octogenarians: a systematic review. Journal of Vascular Surgery. 2008;47(3):676-81.
	Hilditch CA, Nicholson P, Murad MH, Rabinstein A, Schaafsma J, Pikula A, et al. Endovascular Management of Acute Stroke in the Elderly: A Systematic Review and Meta-Analysis. Ajnr: American Journal of Neuroradiology. 2018;39(5):887-91.
	Hoshino N, Fukui Y, Hida K, Sakai Y. Short-term outcomes of laparoscopic surgery for colorectal cancer in the elderly versus non-elderly: a systematic review and meta-analysis. International Journal of Colorectal Disease. 2019;34(3):377-86.
	Hui-Shan L, Watts JN, Peel NM, Hubbard RE, Lin H-S. Frailty and post-operative outcomes in older surgical patients: a systematic review. BMC Geriatrics. 2016;17:1-12.
	Izquierdo M, Cadore EL. Muscle power training in the institutionalized frail: a new approach to counteracting functional declines and very late-life disability. Current Medical Research & Opinion. 2014;30(7):1385-90.
	Jeon JP, Kim SE, Kim CH. Endovascular treatment of acute ischemic stroke in octogenarians: A meta-analysis of observational studies. Clinical Neurology & Neurosurgery. 2017;161:70-7.
	Kechagias A, Rums P, Ylonen K, Kechagias G, Juvonen T, Biancari F. Institutional Results and Meta-Analysis of Outcome after Infrainguinal Surgical Revascularization in Patients Greater than 80 Years Old. American Surgeon. 2011;77(9):1222-9.
	Khan H, Uzzaman M, Benedetto U, Butt S, Raja SG. On- or off-pump coronary artery bypass grafting for octogenarians: A meta-analysis of comparative studies involving 27,623 patients. International Journal Of Surgery. 2017;47:42-51.
	Kheiri B, Osman M, Abdalla A, Haykal T, Chahine A, Gwinn M, et al. Drug-Eluting Versus Bare-Metal Stents in Older Patients: A Meta-Analysis of Randomized Controlled Trials. Cardiovascular Revascularization Medicine. 2019;20(9):744-51.
	Kim SY, Weinberg L, Christophi C, Nikfarjam M. The outcomes of pancreaticoduodenectomy in patients aged 80 or older: a systematic review and meta-analysis. HPB. 2017;19(6):475-82.

	Latif A, Ahsan MJ, Lateef N, Kapoor V, Mirza MM, Anwer F, et al. Outcomes of surgical versus transcatheter aortic valve replacement in nonagenarians- a systematic review and meta-analysis. Journal of Community Hospital Internal Medicine Perspectives. 2021;11(1):128-34.
	Li BSY, Chan CWH, Wong IKY, Yu YHU, Li M. 765 EFFECTIVENESS OF AROMATHERAPY IN MANAGING BEHAVIOURAL AND PSYCHOLOGICAL SYMPTOMS OF DEMENTIA: A MIXED METHODS: SYSTEMATIC REVIEW. Age and Ageing. 2022;51(Supplement):1.
	Li Y, Wang S, Gao S, Yang C, Yang W, Guo S. Laparoscopic colorectal resection versus open colorectal resection in octogenarians: a systematic review and meta-analysis of safety and efficacy. Techniques in Coloproctology. 2016;20(3):153-62.
	Liu Y, Du Y, Fu M, Ma Y, Wang D, Zhang J, et al. Clinical Outcomes of Transcatheter Aortic Valve Replacement in Nonagenarians: A Systematic Review and Meta-Analysis. Journal of Interventional Cardiology. 2019;2019:5819232.
	Luo WM, Wu MY, Chen YL. Laparoscopic versus open surgery for elderly patients with colorectal cancer: a systematic review and meta-analysis of matched studies. Anz Journal of Surgery. 2022;92(9):2003-17.
	Melkonian M, Jarzebowski W, Pautas E, Siguret V, Belmin J, Lafuente-Lafuente C. Bleeding risk of antiplatelet drugs compared with oral anticoagulants in older patients with atrial fibrillation: a systematic review and meta-analysis. Journal of Thrombosis & Haemostasis. 2017;15(7):1500-10.
	Meseguer E, Labreuche J, Olivot JM, Abboud H, Lavallee PC, Simon O, et al. Determinants of outcome and safety of intravenous rt-PA therapy in the very old: a clinical registry study and systematic review. Age & Ageing. 2008;37(1):107-11.
	Miller KJ, Suarez-Iglesias D, Varela S, Rodriguez D, Ayan C. Exercise for Nonagenarians: A Systematic Review. Journal of Geriatric Physical Therapy. 2020;43(4):208-18.
	Minghao X, Huabo Q, Qianxin L, Xiaosheng H, Ping L, Lei L, et al. Laparoscopic Colorectal Resection in Octogenarian Patients: Is it Safe? A Systematic Review and Meta-Analysis. Medicine. 2015;94(42):1-8.
	Moss S, Doyle M, Nagaraja V, Peeceeyen S. A systematic review and meta-analysis of the clinical outcomes of TAVI versus SAVR in the octogenarian population. Indian Journal of Thoracic & Cardiovascular Surgery. 2020;36(4):356-64.
	Pawlaczyk R, Swietlik D, Lango R, Rogowski J. Off-pump coronary surgery may reduce stroke, respiratory failure, and mortality in octogenarians. Annals of Thoracic Surgery. 2012;94(1):29-37.
	Petrelli F, Inno A, Ghidini A, Gori S, Bersanelli M. Efficacy of immune checkpoint inhibitors in elderly patients aged >= 75 years. Cancer Immunology, Immunotherapy. 2021;70(6):1777-80.
	Philp I, Mills KA, Thanvi B. Reducing hospital bed use by frail older people: results from a systematic review of the literature. International Journal of Integration. 2013;13.
	Presutti DG, D'Ascenzo F, Omede P, Biondi-Zoccai G, Moretti C, Bollati M, et al. Percutaneous coronary intervention in nonagenarian: a meta-analysis of observational studies. Journal of Cardiovascular Medicine. 2013;14(11):773-9.
	Quintrec JL, Verlhac B, Cadet C, Breville P, Vetel JM, Gauvain JB, et al. Physical exercise and weight loss for hip and knee osteoarthritis in very old patients: a systematic review of the literature. The open rheumatology journal. 2014;8:89-95.
	Rautalin I, Niemelä M, Korja M. Is surgery justified for 80-year-old or older intracranial meningioma patients? A systematic review. Neurosurgical review. 2020;44(2):1061-9.
	Roosendaal LC, Kramer GM, Wiersema AM, Wisselink W, Jongkind V. Outcome of Ruptured Abdominal Aortic Aneurysm Repair in Octogenarians: A Systematic Review and Meta-Analysis. European Journal of Vascular &

	Endovascular Surgery. 2020;59(1):16-22.
	Ryburn B. Enabling independence: restorative approaches to home care provision for frail older adults. Health and Social Care in the Community. 2009(3).
	Seishima R, Okabayashi K, Hasegawa H, Tsuruta M, Shigeta K, Matsui S, et al. Is laparoscopic Colorectal Surgery Beneficial for Elderly Patients? A Systematic Review and Meta-Analysis. Journal of Gastrointestinal Surgery. 2015;19(4):756-65.
	Shan L, Saxena A, Goh D, Robinson D. A systematic review on the quality of life and functional status after abdominal aortic aneurysm repair in elderly patients with an average age older than 75 years. Journal of Vascular Surgery. 2019;69(4):1268-81.
	Shan L, Saxena A, McMahon R. A Systematic Review on the Quality of Life Benefits after Percutaneous Coronary Intervention in the Elderly. Cardiology. 2014;129(1):46-54.
	Shan L, Saxena A, McMahon R, Wilson A, Newcomb A. A systematic review on the quality of life benefits after aortic valve replacement in the elderly. Journal of Thoracic and Cardiovascular Surgery. 2013;145(5):1173-89.
	Shang XK, Lu R, Liu M, Xiao SN, Dong NG. Mitral valve repair versus replacement in elderly patients: a systematic review and meta-analysis. Journal of Thoracic Disease. 2017;9(9):3045-+.
	Tsuda T, Hashimoto Y, Okamoto Y, Ando W, Ebina K. Meta-analysis for the efficacy of bisphosphonates on hip fracture prevention. Journal of Bone & Mineral Metabolism. 2020;38(5):678-86.
	Van Malderen L, Mets T, Gorus E. Interventions to enhance the Quality of Life of older people in residential long-term care: a systematic review. Ageing Research Reviews. 2013;12(1):141-50.
	Vasques F. Immediate and late outcome of patients aged 80 years and older undergoing isolated aortic valve replacement : a systematic review and meta-analysis of 48 studies. American Heart Journal. 2012;163.
	Vasques F, Lucenteforte E, Paone R, Mugelli A, Biancari F. Outcome of patients aged >= 80 years undergoing combined aortic valve replacement and coronary artery bypass grafting: A systematic review and meta-analysis of 40 studies. American Heart Journal. 2012;164(3):410-U317.
	Wang HY, Shi LY, Tian W, Zhao SJ. Invasive versus conservative strategy in the very elderly with non-ST-elevation acute coronary syndrome: A meta-analysis of randomized controlled trials. Geriatrics & Gerontology International. 2022;22(1):36-41.
	Watkins AR, Fialka N, El-Andari R, Kang JJH, Bozso SJ, Moon MC, et al. Mortality and morbidity of surgical and transcatheter mitral valve repair in octogenarians: A systematic review. Journal of Cardiac Surgery. 2022;37(9):2752-60.
	Wek C, Reichert I, Gee M, Foley R, Ahluwalia R. Have advances in surgical implants and techniques in hemiarthroplasty for intracapsular hip fractures improved patient outcomes compared to THA? A systematic review and meta-analysis of the evidence. Surgeon Journal of the Royal Colleges of Surgeons of Edinburgh & Ireland. 2022;25:25.
	Welsh TJ, Gladman JR, Gordon AL. The treatment of hypertension in people with dementia: a systematic review of observational studies. BMC Geriatrics. 2014;14:19.
	Zhang Q, Zhao XH, Gu HF, Xu ZR, Yang YM. Clinical Outcomes of Coronary Artery Bypass Grafting vs Percutaneous Coronary Intervention in Octogenarians With Coronary Artery Disease. Canadian Journal of Cardiology. 2016;32(9):1166.e21-8.
	Zhao W, Ma P, Zhang P, Yue X. Mechanical Thrombectomy for Acute Ischemic Stroke in Octogenarians: A Systematic Review and Meta-Analysis. Frontiers in neurology [electronic resource]. 2019;10:1355.

	Zhou Z, Fu G, Huang S, Chen S, Liang M, Wu Z. Bilateral Internal Thoracic Artery Coronary Grafting: Risks and Benefits in Elderly Patients. European Heart Journal Quality of Care & Clinical Outcomes. 2021;27:27.
**No Primary Data** (n=43)	Aliyeva T, Chait R. Meta-analysis of complications and success of catheter ablation for atrial fibrillation in octogenarians. Circulation. 2016.
	Amofah HA, Brostrom A, Fridlund B, Haaverstad R, Instenes I, Kuiper KKJ, et al. Octogenarian patients experiences with hypnotics in relation to sleep disturbances and delirium after aortic valve therapy. European Journal of Cardiovascular Nursing. 2018;17:104-5.
	Axelsson K, Lundh D, Lorentzon M. Alendronate treatment is associated with reduced fracture risk and maintained safety in the oldest old. Journal of bone and mineral research Conference: 2016 annual meeting of the american society for bone and mineral research, ASBMR 2016 United states. 2017;31(Supplement 1) (no pagination).
	Barssoum K, Kumar A, Rai D, Elkaryoni A, Thakkar SJ, Ibrahim M, et al. Clinical Outcomes Associated With Anti-coagulation versus No Anti-coagulation for Atrial Fibrillation Among Octogenarians and Nonagenarians; a Meta-analysis. Circulation. 2020;142.
	Bassi M, Sankaramangalam K, Mohapatra S, Broder A, Pitchumoni C. Efficacy and Safety of Endoscopic Retrograde Cholangiopancreatography in Octogenarians: A Systematic Review and Meta-Analysis. Am J Gastroenterol. 2020;115(SUPPL):S498-None.
	Buccafusca G, Cappuccio F, Cordio S, Mare M, Bruera G, Colombo A, et al. Treatment with FOLFIRI-aflibercept in an elderly population (over 75 and octogenarians) with metastatic colorectal cancer after failure of an oxaliplatin-based regimen: Experience in a real-life population. Annals of Oncology. 2020;31:S110-S1.
	Busby C, Sadat U, Aryal K. Age Alone Should not Preclude Surgery: Clinical Outcomes Following Major Emergency General Surgical Procedures in Nonagenarians- a 5-Year Experience. British Journal of Surgery. 2015;102:83-.
	Conradsson M, Littbrand H, Lindelöf N, Gustafson Y, Rosendahl E. Effects of a high-intensity functional exercise program on mental health among older people in residential care facilities: cluster-randomized controlled trial. Physiotherapy (united kingdom). 2011;97:eS232-eS3.
	Dahlin-Ivanoff S, Gosman-Hedstrom G, Edberg AK, Wilhelmson K, Eklund K, Duner A, et al. Elderly persons in the risk zone. Design of a multidimensional, health-promoting, randomised three-armed controlled trial for "prefrail" people of 80+ years living at home. BMC Geriatrics. 2010;10:27.
	Damluji A, Cohen ER, Myerburg R, Cohen M, Moscucci M, Rich M, et al. Insulin provision therapy and increased mortality in elderly patients with diabetes mellitus and stable ischemic heart disease: insights from BARI-2D trial. Journal of the American College of Cardiology. 2015;65(10 SUPPL. 1):A1592.
	Dels Angels Cebria IIM, Martínez DS, Camacho CI, Bueno LL, Chuliá Á, Tomás JM. Comparison between two inspiratory muscle-training programs in frail elderly: a randomized controlled trial. European respiratory journal. 2014;44.
	Ding Y, Si QJ. The multi effects of policosanol on cardiovascular diseases in the very old patients. Journal of the American Geriatrics Society. 2015;63:S382-S3.
	Dodge HH, Bowman M, Zhau J, Matteck N, Wild K, Kaye J. A 6-week randomized controlled trial to increase social interactions using home-based technologies improved language-based executive function. Alzheimer's & dementia. 2014;10:P442-.
	Downes T, Morgan N, Rash A, Channer K. A comparison of warfarin and aspirin for stroke prevention in octogenarians in atrial fibrillation study. Age and ageing. 2003;32 (Suppl 2):ii43.
	Evans WJ. Exercise training guidelines for the elderly. Medicine & Science in

	Sports & Exercise. 1999;31(1):12-7.
	Fiatarone MA, O'Neill EF, Doyle N, Clements KM, Roberts SB, Kehayias JJ, et al. The Boston FICSIT study: the effects of resistance training and nutritional supplementation on physical frailty in the oldest old. Journal of the American Geriatrics Society. 1993;41(3):333-7.
	Fulton RL, Witham MD, Greig C, Johnston D, Lang CC, Van Der Pol M, et al. A randomised controlled trial of exercise training for older heart failure patients. Age and ageing. 2011;40:ii9-.
	Hwang K, Hwang G, Kwon OK, Kim CH, Ban SP, Han MK, et al. Endovascular Treatment for Acute Ischemic Stroke Patients over 80 Years of Age. Journal of Cerebrovascular & Endovascular Neurosurgery. 2015;17(3):173-9.
	Ilic N, Ilic D, Juricic J, Krnic D, Frleta Ilic N, Simundza I, et al. Intubated versus non-intubated general anesthesia for minimally invasive videoassisted thoracic surgery (VATS) in octogenarians. Journal of thoracic oncology. 2018;13(4):S53-.
	Ilic N, Ilic D, Juricic J, Krnic D, Orsulic D, Simundza I, et al. Intubated versus non-intubated anesthesia for lung cancer VATS in octogenarians. Journal of thoracic oncology. 2017;12(11):S2051-.
	Ilic N, Ilic D, Juricic J, Krnic D, Orsulic D, Simundza I, et al. Intubated versus nonintubated general anesthesia for minimally invasive video-assisted thoracic surgery in octogenarians. Innovations: technology and techniques in cardiothoracic and vascular surgery. 2018;13:S98-.
	Instenes I, Fridlund B, Eide LSP, Ranhoff AH, Amofah HA, Norekval TM. A qualitative study on how octogenarians experience relatives and health professionals when being in a delrious state after aortic valve therapy. European Journal of Cardiovascular Nursing. 2017;16:S84-S5.
	Instenes I, Gjengedal E, Eide LSP, Kuiper KKJ, Ranhoff AH, Amofah HA, et al. "The nights were terrible; sleeping was a problem all the time": Octogenarians experience with sleep and delirium after aortic valve therapy. European Journal of Cardiovascular Nursing. 2016;15:S13-S.
	Iqbal U, Anwar H, Khan MA, Weissman S, Kothari ST, Kothari TH, et al. Safety and Efficacy of Endoscopic Retrograde Cholangiopancreatography in Nonagenarians: A Systematic Review and Meta-Analysis. Digestive diseases and sciences. 2021.
	Kwak KP. Exercise training in very old adults with mild cognitive impairment: improvements on BDNF peripheral levels and cognition. Alzheimer's & dementia. 2015;11(7 SUPPL. 1):P722.
	Kwak KP, Lee S, Kim T, Bae N. Cognitive training programs for very old lone adults in a Korean rural community. Alzheimer's & dementia. 2015;11(7 SUPPL. 1):P590.
	Legoux JL, Faroux R, Barriere N, Le Malicot K, Tougeron D, Lorgis V, et al. PRODIGE 25 (FFCD 11-01)-FOLFA: a randomized phase II trial evaluating aflibercept associated with LV5FU2 regimen as first-line treatment of non-resectable metastatic colorectal cancers. Annals of oncology. 2020;31:S430-S1.
	Li KL, Si QJ. A comparative study in the very old patients treated with different antiplatelet agents. Journal of the American Geriatrics Society. 2015;63:S378-S9.
	Marston HR, Freeman S, Bishop KA, Beech CL. A Scoping Review of Digital Gaming Research Involving Older Adults Aged 85 and Older. Games for Health Journal. 2016;5(3):157-74.
	Meziere A. Exercise inerventions with trained carers for preventing loss of autonomy and falls in elderly people at home (T4H): a cluster randomized controlled pilot trial. European geriatric medicine. 2019;10:S177-S8.
	Neafsey PJ, Anderson E, Coleman C, Lin CA, M'Lan CE, Walsh S. Reducing adverse self-medication behaviors in older adults with the Next Generation Personal Education Program (PEP-NG): Design and methodology. Patient Preference and Adherence. 2009;3:323-34.

	Ohoka T, Urabe Y, Shirakawa T. Therapeutic exercises for proximal femoral fracture of super-aged patients: effect of walking assistance using body weight-supported treadmill training (BWSTT). Physiotherapy (united kingdom). 2015;101:eS1124-eS5.
	Ortiz Alonso J, Javier M, Hidalgo M, López M, Valades M, González C, et al. Randomized controlled trial of an exercise intervention to improve functional capacity in hospitalized oldest old (AGECAR). Journal of the American Geriatrics Society. 2014;62:S8-.
	Pedrinolla A, Pogliaghi S, Colosio A, Muti E, Ce E, Longo S, et al. Passive mobilization-induced vascular function adaptations in bedridden oldest-old. FASEB journal. 2018;32(1).
	Piper AM, Garcia RC, Brewer RN. Understanding the challenges and opportunities of smart mobile devices among the oldest old. Special Issue on Reimagining Interfaces for Older Adults. 2016;8(2):83-98.
	Rodriguez-Pascual C, Paredes-Galan E, Ferrero-Martinez AL, Gonzalez-Guerrero JL, Hornillos M, Abizanda P, et al. A disease management program intervention in elderly patients with high comorbidity: results of a randomized-controlled trial (HF-GERIATRICS). European geriatric medicine. 2013;4:S15-.
	Shengde Z, Han L, Guoliang Z, Shidong L, Zijian L, Zhu S, et al. Comparison of outcome of transcatheter aortic valve implantation in patients with advanced age: A systematic review and meta-analysis. Medicine. 2020;99(31):1-3.
	Sobotka O, Fortunato J, Skorepa P, Blaha V, Havlova K, Lejskova L, et al. EFFECT OF EARLY GLUCOSE INFUSION ON PLASMA MINERAL LEVELS IN VERY OLD PATIENTS. Clinical nutrition (Edinburgh, Scotland). 2019;38:S183-S4.
	Talapatra RN, Pande A, Khan A, Chakraborty R. Short and intermediate term outcome of percutaneous coronary intervention in octogenarians. Indian heart journal. 2015;67:S64.
	Tortosa MA, Cebria IIMA. Preventive intervention in disabled institutionalized elderly: comparison between two inspiratory muscle training. European geriatric medicine. 2014;5:S174-S5.
	White SI, Wright D, Taylor CJ. A comparison of laparoscopic versus open rectal surgery in 114 consecutive octogenarians. Colorectal Disease. 2012;14(7):e395-9.
	Widell EC, Andreen ES, Axelsson AB. A better life-expectations of octogenarians on acute coronary syndrome treatment. European journal of cardiovascular nursing. 2015;14:66.
	Wubbeke LF, Naves CCLM, Daemen JWHC, Jacobs MJ, Mees BM. Mortality and Major Amputation after Revascularisation in Octogenarians Versus Non-Octogenarians with Chronic Limb Threatening Ischaemia: A Systematic Review and Meta-Analysis. J Vasc Surg. 2020;72(3):1146-7.
**Unable to translate** (n=3)	Cebrià IIMD, Arnall DA, Igual Camacho C, Tomás JM, Meléndez JC. Physiotherapy intervention for preventing the respiratory muscle deterioration in institutionalized older women with functional impairment. Archivos de bronconeumologia. 2013;49(1):1-9.
	Cebrià IIMD, Tortosa-Chuliá M, Igual-Camacho C, Sancho P, Galiana L, Tomás JM. Cost-consequence analysis of respiratory preventive intervention among institutionalized older people: randomized controlled trial. Revista espanola de geriatria y gerontologia. 2014;49(5):203-9.
	Choi W, Mizukami K. The effect of whole body vibration by sonic waves on mood, the autonomic nervous system, and brain function in elderly. Nihon Ronen Igakkai zasshi [Japanese journal of geriatrics]. 2020;57(4):441-9.

**Age of population**	

(n=731)	
1	Assessment of the Evercare programme in England 2003 2004: executive summary. 2005.
2	National evaluation of the Evercare approach to case management of frail elderly people. 2006.
3	Aas SN, Seynnes O, Benestad HB, Raastad T. Strength training and protein supplementation improve muscle mass, strength, and function in mobility-limited older adults: a randomized controlled trial. Aging-Clinical & Experimental Research. 2020;32(4):605-16.
4	Abe S, Ezaki O, Suzuki M. Medium-Chain Triglycerides in Combination with Leucine and Vitamin D Increase Muscle Strength and Function in Frail Elderly Adults in a Randomized Controlled Trial. Journal of nutrition. 2016;146(5):1017-26.
5	Abe S, Ezaki O, Suzuki M. Medium-Chain Triglycerides in Combination with Leucine and Vitamin D Benefit Cognition in Frail Elderly Adults: a Randomized Controlled Trial. Journal of nutritional science and vitaminology. 2017;63(2):133-40.
6	Abraha I, Rimland JM, Lozano-Montoya I, Dell'Aquila G, Velez-Diaz-Pallares M, Trotta FM, et al. Simulated presence therapy for dementia. Cochrane Database of Systematic Reviews. 2017;4:CD011882.
7	Abraha I, Rimland JM, Lozano-Montoya I, Dell'Aquila G, Velez-Diaz-Pallares M, Trotta FM, et al. Simulated presence therapy for dementia. The Cochrane database of systematic reviews. 2020;4:CD011882.
8	Alakare J, Kemp K, Strandberg T, Castren M, Jakovljevic D, Tolonen J, et al. Systematic geriatric assessment for older patients with frailty in the emergency department: a randomised controlled trial. BMC geriatrics. 2021;21(1):408.
9	Alemania E, Hind A, Samara J, Turner M, Ralph N, Paterson C. Nurse-led interventions among older adults affected by cancer: An integrative review. Asia-Pacific journal of oncology nursing. 2023;10(10):100289.
10	Alexander NB, Galecki AT, Grenier ML, Nyquist LV, Hofmeyer MR, Grunawalt JC, et al. Task-specific resistance training to improve the ability of activities of daily living-impaired older adults to rise from a bed and from a chair. Journal of the American Geriatrics Society. 2001;49(11):1418-27.
11	Alexopoulos G, Group O-ODS, Hakkarainen H, Krishnan KRR, Lavretsky H, Pollock BG, et al. Antidepressant pharmacotherapy in the treatment of depression in the very old: a randomized, placebo-controlled trial. American Journal of Psychiatry. 2004;161(11):2050-9.
12	Allard J, Hébert R, Rioux M, Asselin J, Voyer L. Efficacy of a clinical medication review on the number of potentially inappropriate prescriptions prescribed for community-dwelling elderly people. CMAJ : Canadian Medical Association journal. 2001;164(9):1291-6.
13	Altfeld SJ, Shier GE, Rooney M, Johnson TJ, Golden RL, Karavolos K, et al. Effects of an enhanced discharge planning intervention for hospitalized older adults: a randomized trial. Gerontologist. 2013;53(3):430-40.
14	Amarilla-Donoso FJ, Roncero-Martín R, Lavado-García J, Canal-Macías ML, Pedrera-Canal M, Chimpén-López C, et al. Impact of a Postoperative Intervention Educational Program on the Quality of Life of Patients with Hip Fracture: a Randomized, Open-Label Controlled Trial. International journal of environmental research and public health. 2020;17(24).
15	Amirov CM, Binns MA, Jacob LE, Candon HL. Impact of chlorhexidine bathing on methicillin-resistant Staphylococcus aureus incidence in an endemic chronic care setting: a randomized controlled trial. American journal of infection control. 2017;45(3):298-300.
16	Anderson-Hanley C, Meshberg SR, Marsh MA. The effects of a control-enhancing intervention for nursing home residents: cognition and locus of control as moderators. Palliative & supportive care. 2003;1(2):111-20.
17	Anne W. Improving life satisfaction for the elderly living independently in the community: care recipients' perspective of volunteers. Social Work in Health Care. 2012;51(2):125-39.

18	Anouk O, et al. Experiences with and outcomes of Advance Care Planning in bereaved relatives of frail older patients: a mixed methods study. Age and Ageing. 2019;48(2):299-306.
19	Apostolo J, Cooke R, Bobrowicz-Campos E, Santana S, Marcucci M, Cano A, et al. Effectiveness of the interventions in preventing the progression of pre-frailty and frailty in older adults: a systematic review protocol. JBI database of systematic reviews and implementation reports. 2016;14(1):4-19.
20	Apostolo J, Dixe MDA, Bobrowicz-Campos E, Areosa T, Santos-Rocha R, Brauna M, et al. Effectiveness of a Combined Intervention on Psychological and Physical Capacities of Frail Older Adults: A Cluster Randomized Controlled Trial. International journal of environmental research and public health. 2019;16(17).
21	Arendts G, Deans P, O'Brien K, Etherton-Beer C, Howard K, Lewin G, et al. A clinical trial of nurse practitioner care in residential aged care facilities. Archives of gerontology and geriatrics. 2018;77:129-32.
22	Arthur AJ, Jagger C, Lindesay J, Matthews RJ. Evaluating a mental health assessment for older people with depressive symptoms in general practice: a randomised controlled trial. The British journal of general practice : the journal of the Royal College of General Practitioners. 2002;52(476):202-7.
23	Avgerinou C, Gardner B, Kharicha K, Frost R, Liljas A, Elaswarapu R, et al. Health promotion for mild frailty based on behaviour change: Perceptions of older people and service providers. Health & social care in the community. 2019;27(5):1333-43.
24	Ayalon L. Fears come true: the experiences of older care recipients and their family members of live-in foreign home care workers. International psychogeriatrics. 2009;21(4):779-86.
25	Ayalon L, Kaniel M, Rosenberg L. Social workers' perspectives on care arrangements between vulnerable elders and foreign home care workers. Home Health Care Services Quarterly. 2008;27(2).
26	Ayton D, Morello R, Natora A. Perceptions of falls and falls prevention interventions among Personal Alert Victoria clients. Health and Social Care in the Community. 2018;26(6).
27	Baleztena J, Ruiz-Canela M, Sayon-Orea C, Pardo M, Anorbe T, Gost JI, et al. Association between cognitive function and supplementation with omega-3 PUFAs and other nutrients in >= 75 years old patients: A randomized multicenter study. PLoS ONE [Electronic Resource]. 2018;13(3):e0193568.
28	Baleztena J, Ruiz-Canela M, Sayon-Orea C, Pardo M, Añorbe T, Gost JI, et al. Association between cognitive function and supplementation with omega-3 PUFAs and other nutrients in ≥ 75 years old patients: a randomized multicenter study. PloS one. 2018;13(3):e0193568.
29	Ballard C, Corbett A, Orrell M, Williams G, Moniz-Cook E, Romeo R, et al. Impact of person-centred care training and person-centred activities on quality of life, agitation, and antipsychotic use in people with dementia living in nursing homes: a cluster-randomised controlled trial. PLoS medicine. 2018;15(2):e1002500.
30	Banerjee S, High J, Stirling S, Shepstone L, Swart AM, Telling T, et al. Study of mirtazapine for agitated behaviours in dementia (SYMBAD): a randomised, double-blind, placebo-controlled trial. Lancet (london, england). 2021;398(10310):1487-97.
31	Bang S, Chung J, Jeong J, Bak H, Kim D. Efficacy of ultrasound-guided fascia iliaca compartment block after hip hemiarthroplasty: a prospective, randomized trial. Medicine. 2016;95(39):e5018.
32	Bath PM, Skinner CJC, Bath CS, Woodhouse LJ, Korovesi AAK, Long H, et al. Dietary nitrate supplementation for preventing and reducing the severity of winter infections, including COVID-19, in care homes (BEET-Winter): a randomised placebo-controlled feasibility trial. European geriatric medicine. 2022;13(6):1343-55.
33	Baumgarten M, Lebel P, Laprise H. Adult day care for the frail elderly. Journal of Aging and Health. 2002;14(2).
34	Baumgartner A, Pachnis D, Parra L, Hersberger L, Bargetzi A, Bargetzi L, et al.

	The impact of nutritional support on malnourished inpatients with aging-related vulnerability. Nutrition (Burbank, Los Angeles County, Calif). 2021;89:111279.
35	Beauchet O, Fantino B, Allali G, Muir SW, Montero-Odasso M, Annweiler C. Timed Up and Go test and risk of falls in older adults: a systematic review. The journal of nutrition, health & aging. 2011;15(10):933-8.
36	Beaupre LA, Lier D, Magaziner JS, Jones CA, Johnston DWC, Wilson DM, et al. An Outreach Rehabilitation Program for Nursing Home Residents After Hip Fracture May Be Cost-Saving. Journals of gerontology Series A, Biological sciences and medical sciences. 2020;75(10):e159-e65.
37	Beaupre LA, Magaziner JS, Jones CA, Jhangri GS, Johnston DWC, Wilson DM, et al. Rehabilitation After Hip Fracture for Nursing Home Residents: a Controlled Feasibility Trial. Journals of gerontology Series A, Biological sciences and medical sciences. 2019;74(9):1518-25.
38	Benali F, Kappelhof M, Ospel J, Ganesh A, McDonough RV, Postma AA, et al. Benefit of successful reperfusion achieved by endovascular thrombectomy for patients with ischemic stroke and moderate pre-stroke disability (mRS 3): results from the MR CLEAN Registry. Journal of neurointerventional surgery. 2023;15(5):433-8.
39	Bentzen H, Forsén L, Becker C, Bergland A. Uptake and adherence with soft- and hard-shelled hip protectors in Norwegian nursing homes: a cluster randomised trial. Osteoporosis international. 2008;19(1):101-11.
40	Berggren M, Stenvall M, Olofsson B, Gustafson Y. Evaluation of a fall-prevention program in older people after femoral neck fracture: a one-year follow-up. Osteoporosis international : a journal established as result of cooperation between the European Foundation for Osteoporosis and the National Osteoporosis Foundation of the USA. 2008;19(6):801-9.
41	Berglund H, Hasson H, Kjellgren K, Wilhelmson K. Effects of a continuum of care intervention on frail older persons' life satisfaction: a randomized controlled study. Journal of clinical nursing. 2015;24(7-8):1079-90.
42	Berglund H, Wilhelmson K, Blomberg S, Duner A, Kjellgren K, Hasson H. Older people's views of quality of care: a randomised controlled study of continuum of care. Journal of clinical nursing. 2013;22(19-20):2934-44.
43	Bergstrom N, Horn SD, Rapp M, Stern A, Barrett R, Watkiss M, et al. Preventing Pressure Ulcers: a Multisite Randomized Controlled Trial in Nursing Homes. Ontario health technology assessment series. 2014;14(11):1-32.
44	Berner J, Anderberg P, Rennemark M, Berglund J. Case management for frail older adults through tablet computers and Skype. Informatics for health & social care. 2016;41(4):405-16.
45	Berning MJ, Oliveira J E Silva L, Suarez NE, Walker LE, Erwin P, Carpenter CR, et al. Interventions to improve older adults' Emergency Department patient experience: A systematic review. The American journal of emergency medicine. 2020;38(6):1257-69.
46	Bindels J, Cox K, De La Haye J, Mevissen G, Heijing S, van Schayck OCP, et al. Losing connections and receiving support to reconnect: experiences of frail older people within care programmes implemented in primary care settings. International journal of older people nursing. 2015;10(3):179-89.
47	Bindels J, Cox K, Widdershoven G, van Schayck OCP, Abma TA. Care for community-dwelling frail older people: a practice nurse perspective. Journal of clinical nursing. 2014;23(15-16):2313-22.
48	Binder EF, Schechtman KB, Ehsani AA, Steger-May K, Brown M, Sinacore DR, et al. Effects of exercise training on frailty in community-dwelling older adults: results of a randomized, controlled trial. Journal of the American Geriatrics Society. 2002;50(12):1921-8.
49	Binder EF, Yarasheski KE, Steger-May K, Sinacore DR, Brown M, Schechtman KB, et al. Effects of progressive resistance training on body composition in frail older adults: results of a randomized, controlled trial. The journals of gerontology Series A, Biological sciences and medical sciences. 2005;60(11):1425-31.

50	Biston P, Aldecoa C, Devriendt J, Madl C, Chochrad D, Vincent J-L, et al. Outcome of elderly patients with circulatory failure. Intensive Care Medicine. 2014;40(1):50-6.
51	Bjerk M, Brovold T, Skelton DA, Liu-Ambrose T, Bergland A. Effects of a falls prevention exercise programme on health-related quality of life in older home care recipients: a randomised controlled trial. Age and ageing. 2019;48(2):213-9.
52	Björkman MP, Sorva AJ, Risteli J, Tilvis RS. Low parathyroid hormone levels in bedridden geriatric patients with vitamin D deficiency. Journal of the American Geriatrics Society. 2009;57(6):1045-50.
53	Blanc-Bisson C, Dechamps A, Gouspillou G, Dehail P, Bourdel-Marchasson I. A randomized controlled trial on early physiotherapy intervention versus usual care in acute care unit for elderly: potential benefits in light of dietary intakes. Journal of nutrition, health & aging. 2008;12(6):395-9.
54	Bleijenberg N, Imhof L, Mahrer-Imhof R, Wallhagen MI, de Wit NJ, Schuurmans MJ. Patient Characteristics Associated With a Successful Response to Nurse-Led Care Programs Targeting the Oldest-Old: A Comparison of Two RCTs. Worldviews on Evidence-Based Nursing. 2017;14(3):210-22.
55	Bleijenberg N, Ten Dam VH, Steunenberg B, Drubbel I, Numans ME, De Wit NJ, et al. Exploring the expectations, needs and experiences of general practitioners and nurses towards a proactive and structured care programme for frail older patients: a mixed-methods study. Journal of advanced nursing. 2013;69(10):2262-73.
56	Blom JW, et al. Effectiveness and cost-effectiveness of proactive and multidisciplinary integrated care for older people with complex problems in general practice: an individual participant data meta-analysis. Age and Ageing. 2018;47(5):705-14.
57	Blomqvist K, Edberg A-K. Living with persistent pain: experiences of older people receiving home care. Journal of advanced nursing. 2002;40(3):297-306.
58	Boere TM, van Buul LW, Hopstaken RM, van Tulder MW, Twisk J, Verheij TJM, et al. Effect of C reactive protein point-of-care testing on antibiotic prescribing for lower respiratory tract infections in nursing home residents: cluster randomised controlled trial. BMJ (Clinical research ed). 2021;374:n2198.
59	Bollig C, Torbahn G, Bauer J, Brefka S, Dallmeier D, Denkinger M, et al. Evidence gap on antihyperglycemic pharmacotherapy in frail older adults : A systematic review. Evidenzmangel fur die antihyperglykamische Pharmakotherapie gebrechlicher alterer Patienten : Ein systematisches Review. 2021;54(3):278-84.
60	Bonjour JP, Benoit V, Payen F, Kraenzlin M. Consumption of yogurts fortified in vitamin D and calcium reduces serum parathyroid hormone and markers of bone resorption: a double-blind randomized controlled trial in institutionalized elderly women. Journal of clinical endocrinology and metabolism. 2013;98(7):2915-21.
61	Bonjour JP, Benoit V, Pourchaire O, Rousseau B, Souberbielle JC. Nutritional approach for inhibiting bone resorption in institutionalized elderly women with vitamin D insufficiency and high prevalence of fracture. Journal of nutrition, health & aging. 2011;15(5):404-9.
62	Bonnet-Zamponi D, d'Arailh L, Konrat C, Delpierre S, Lieberherr D, Lemaire A, et al. Drug-related readmissions to medical units of older adults discharged from acute geriatric units: results of the Optimization of Medication in AGEd multicenter randomized controlled trial. Journal of the American Geriatrics Society. 2013;61(1):113-21.
63	Boonen S, McClung MR, Eastell R, El-Hajj Fuleihan G, Barton IP, Delmas P. Safety and efficacy of risedronate in reducing fracture risk in osteoporotic women aged 80 and older: implications for the use of antiresorptive agents in the old and oldest old. Journal of the American Geriatrics Society. 2004;52(11):1832-9.
64	Borenstein JE, Aronow HU, Bolton LB, Dimalanta MI, Chan E, Palmer K, et al. Identification and team-based interprofessional management of hospitalized vulnerable older adults. Nursing outlook. 2016;64(2):137-45.

65	Bossers WJ, van der Woude LH, Boersma F, Hortobágyi T, Scherder EJ, van Heuvelen MJ. A 9-Week Aerobic and Strength Training Program Improves Cognitive and Motor Function in Patients with Dementia: a Randomized, Controlled Trial. American journal of geriatric psychiatry. 2015;23(11):1106-16.
66	Bossers WJ, van der Woude LH, Boersma F, Hortobágyi T, Scherder EJ, van Heuvelen MJ. Comparison of Effect of Two Exercise Programs on Activities of Daily Living in Individuals with Dementia: a 9-Week Randomized, Controlled Trial. Journal of the American Geriatrics Society. 2016;64(6):1258-66.
67	Botella-Carretero JI, Iglesias B, Balsa JA, Zamarrón I, Arrieta F, Vázquez C. Effects of oral nutritional supplements in normally nourished or mildly undernourished geriatric patients after surgery for hip fracture: a randomized clinical trial. JPEN Journal of parenteral and enteral nutrition. 2008;32(2):120-8.
68	Bouillanne O, Curis E, Hamon-Vilcot B, Nicolis I, Chrétien P, Schauer N, et al. Impact of protein pulse feeding on lean mass in malnourished and at-risk hospitalized elderly patients: a randomized controlled trial. Clinical nutrition (Edinburgh, Scotland). 2013;32(2):186-92.
69	Bouillanne O, Neveux N, Nicolis I, Curis E, Cynober L, Aussel C. Long-lasting improved amino acid bioavailability associated with protein pulse feeding in hospitalized elderly patients: a randomized controlled trial. Nutrition (Burbank, Los Angeles County, Calif). 2014;30(5):544-50.
70	Boult C, Reider L, Frey K, Leff B, Boyd CM, Wolff JL, et al. Early effects of "Guided Care" on the quality of health care for multimorbid older persons: a cluster-randomized controlled trial. The journals of gerontology Series A, Biological sciences and medical sciences. 2008;63(3):321-7.
71	Briggs R, McDonough A, Ellis G, Bennett K, O'Neill D, Robinson D. Comprehensive Geriatric Assessment for community-dwelling, high-risk, frail, older people. The Cochrane database of systematic reviews. 2022;5:CD012705.
72	Briggs S, Pearce R, Dilworth S, Higgins I, Hullick C, Attia J. Clinical pharmacist review: a randomised controlled trial. Emergency medicine Australasia. 2015;27(5):419-26.
73	Brovold T, Skelton DA, Bergland A. Older adults recently discharged from the hospital: effect of aerobic interval exercise on health-related quality of life, physical fitness, and physical activity. Journal of the American Geriatrics Society. 2013;61(9):1580-5.
74	Brown PM, Wilkinson-Meyers L, Parsons M, Weidenbohm K, McNeill R, Brandt T. Cost of prescribed and delivered health services resulting from a comprehensive geriatric assessment tool in New Zealand. Health & social care in the community. 2009;17(5):514-21.
75	Bryce C, Fleming J, Reeve J. Implementing change in primary care practice: lessons from a mixed-methods evaluation of a frailty initiative. BJGP Open. 2018;2(1).
76	Buckinx F, Beaudart C, Maquet D, Demonceau M, Crielaard JM, Reginster JY, et al. Evaluation of the impact of 6-month training by whole body vibration on the risk of falls among nursing home residents, observed over a 12-month period: a single blind, randomized controlled trial. Aging clinical and experimental research. 2014;26(4):369-76.
77	Bueno H, Betriu A, Heras M, Alonso JJ, Cequier A, García EJ, et al. Primary angioplasty vs. fibrinolysis in very old patients with acute myocardial infarction: TRIANA (TRatamiento del Infarto Agudo de miocardio eN Ancianos) randomized trial and pooled analysis with previous studies. European heart journal. 2011;32(1):51-60.
78	Bujnowska-Fedak MM, Gwyther H, Szwamel K, D'Avanzo B, Holland C, Shaw RL, et al. A qualitative study examining everyday frailty management strategies adopted by Polish stakeholders. The European journal of general practice. 2019;25(4):197-204.
79	Bula CJ, Berod AC, Stuck AE, Alessi CA, Aronow HU, Santos-Eggimann B, et al. Effectiveness of preventive in-home geriatric assessment in well functioning, community-dwelling older people: secondary analysis of a randomized trial. Journal of the American Geriatrics Society. 1999;47(4):389-95.

80	Bulpitt CJ, Beckett NS, Peters R, Banya W, Liu L, Wang JG, et al. Baseline characteristics of participants in the Hypertension in the Very Elderly Trial (HYVET). Blood Pressure. 2009;18(1-2):17-22.
81	Bungay H, Jacobs C. Dance for Health: The perceptions of healthcare professionals of the impact of music and movement sessions for older people in acute hospital settings. International journal of older people nursing. 2020;15(4):e12342.
82	Bunn D, Jimoh F, Wilsher SH, Hooper L. Increasing fluid intake and reducing dehydration risk in older people living in long-term care: a systematic review. Journal of the American Medical Directors Association. 2015;16(2):101-13.
83	Burdinski S, Smeaton S, Lutz SZ, Partheymuller A, Geyik U, Eschweiler GW, et al. [Telemedicine in mobile geriatric rehabilitation : Results of the VITAAL study]. Zeitschrift fur Gerontologie und Geriatrie. 2022;55(4):292-7.
84	Burns A, Banerjee S, Morris J, Woodward Y, Baldwin R, Proctor R, et al. Treatment and prevention of depression after surgery for hip fracture in older people: randomized, controlled trials. Journal of the American Geriatrics Society. 2007;55(1):75-80.
85	Burns A, Bernabei R, Bullock R, Cruz Jentoft AJ, Frölich L, Hock C, et al. Safety and efficacy of galantamine (Reminyl) in severe Alzheimer's disease (the SERAD study): a randomised, placebo-controlled, double-blind trial. The lancet Neurology. 2009;8(1):39-47.
86	Buurman BM, Parlevliet JL, Allore HG, Blok W, van Deelen BAJ, Moll van Charante EP, et al. Comprehensive Geriatric Assessment and Transitional Care in Acutely Hospitalized Patients: The Transitional Care Bridge Randomized Clinical Trial. JAMA internal medicine. 2016;176(3):302-9.
87	Byles JE, Francis L, McKernon M. The experiences of non-medical health professionals undertaking community-based health assessments for people aged 75 years and over. Health & social care in the community. 2002;10(2):67-73.
88	Byles JE, Tavener M, O'Connell RL, Nair BR, Higginbotham NH, Jackson CL, et al. Randomised controlled trial of health assessments for older Australian veterans and war widows. The Medical journal of Australia. 2004;181(4):186-90.
89	Cadore EL, Rodriguez-Manas L, Sinclair A, Izquierdo M. Effects of different exercise interventions on risk of falls, gait ability, and balance in physically frail older adults: a systematic review. Rejuvenation research. 2013;16(2):105-14.
90	Caldo-Silva A, Furtado GE, Chupel MU, Letieri RV, Valente PA, Farhang M, et al. Effect of a 40-weeks multicomponent exercise program and branched chain amino acids supplementation on functional fitness and mental health in frail older persons. Experimental gerontology. 2021;155:111592.
91	Cameron ID, Cumming RG, Kurrle SE, Quine S, Lockwood K, Salkeld G, et al. A randomised trial of hip protector use by frail older women living in their own homes. Injury prevention : journal of the International Society for Child and Adolescent Injury Prevention. 2003;9(2):138-41.
92	Cameron ID, Fairhall N, Langron C, Lockwood K, Monaghan N, Aggar C, et al. A multifactorial interdisciplinary intervention reduces frailty in older people: randomized trial. BMC medicine. 2013;11:65.
93	Cameron ID, Kurrle S, Quine S, Sambrook P, March L, Chan D, et al. Increasing adherence with the use of hip protectors for older people living in the community. Osteoporosis international. 2011;22(2):617-26.
94	Campbell E, Petermann-Rocha F, Welsh P, Celis-Morales C, Pell JP, Ho FK, et al. The effect of exercise on quality of life and activities of daily life in frail older adults: A systematic review of randomised control trials. Experimental gerontology. 2021;147:111287.
95	Cancela JM, Perez CA, Rodrigues LP, Bezerra P. The Long-Term Benefits of a Multicomponent Physical Activity Program to Body Composition, Muscle Strength, Cardiorespiratory Capacity, and Bone Mineral Density in a Group of Nonagenarians. Rejuvenation Research. 2020;23(3):217-23.
96	Canet-Velez O, Solis-Navarro L, Sitja-Rabert M, Perez LM, Roca J, Soto-Bagaria L, et al. Experience, facilitators, and barriers to the implementation of a multicomponent programme in older people living in the community, +AGIL

	Barcelona: A qualitative study. Frontiers in public health. 2023;11:1161883.
97	Caplan GA, Williams AJ, Daly B, Abraham K. A randomized, controlled trial of comprehensive geriatric assessment and multidisciplinary intervention after discharge of elderly from the emergency department--the DEED II study. Journal of the American Geriatrics Society. 2004;52(9):1417-23.
98	Carballeira E, Censi KC, Maseda A, López-López R, Lorenzo-López L, Millán-Calenti JC. Low-volume cycling training improves body composition and functionality in older people with multimorbidity: a randomized controlled trial. Scientific reports. 2021;11(1):13364.
99	Carpenter GI, Demopoulos GR. Screening the elderly in the community: controlled trial of dependency surveillance using a questionnaire administered by volunteers. BMJ (Clinical research ed). 1990;300(6734):1253-6.
100	Cassidy-Eagle E, Siebern A, Unti L, Glassman J, O'Hara R. Neuropsychological Functioning in Older Adults with Mild Cognitive Impairment and Insomnia Randomized to CBT-I or Control Group. Clinical gerontologist. 2018;41(2):136-44.
101	Castellanos VH, Marra MV, Johnson P. Enhancement of select foods at breakfast and lunch increases energy intakes of nursing home residents with low meal intakes. Journal of the American Dietetic Association. 2009;109(3):445-51.
102	Castello PA. The world of very elderly: is the effectiveness of interventions measurable? Assistenza Infermieristica E Ricerca. 2013;32(1):46-53.
103	Castillo-Perez M, Jerjes-Sanchez C, Rodriguez D, Paredes-Vazquez JG, Panneflek J, Vazquez-Guajardo M. Clinical outcomes of very elderly patients treated with ultrasound-assisted catheter-directed thrombolysis for pulmonary embolism: a systematic review. Journal of Thrombosis & Thrombolysis. 2021;52(1):260-71.
104	Cecilia D, Jódar E, Fernández C, Resines C, Hawkins F. Effect of alendronate in elderly patients after low trauma hip fracture repair. Osteoporosis international. 2009;20(6):903-10.
105	Cederbom S, Leveille SG, Bergland A. Effects of a behavioral medicine intervention on pain, health, and behavior among community-dwelling older adults: a randomized controlled trial. Clinical Interventions In Aging. 2019;14:1207-20.
106	Cederbom S, Rydwik E, Söderlund A, Denison E, Frändin K, von Heideken Wågert P. A behavioral medicine intervention for older women living alone with chronic pain - a feasibility study. Clinical interventions in aging. 2014;9:1383-97.
107	Celis H, Fagard RH, Staessen JA, Thijs L. Risk and benefit of treatment of isolated systolic hypertension in the elderly: evidence from the Systolic Hypertension in Europe Trial. Current Opinion in Cardiology. 2001;16(6):342-8.
108	Challis D, Clarkson P, Williamson J, Hughes J, Venables D, Burns A, et al. The value of specialist clinical assessment of older people prior to entry to care homes. Age and ageing. 2004;33(1):25-34.
109	Chan TC, Hung IF, Chan KH, Li CP, Li PT, Luk JK, et al. Immunogenicity and safety of intradermal trivalent influenza vaccination in nursing home older adults: a randomized controlled trial. Journal of the American Medical Directors Association. 2014;15(8):607.e5-12.
110	Chen Y-C, Lin K-C, Chen C-J, Wang C-H. Effects of leisure-time physical activity interventions on frailty-related characteristics of frail older adults in long-term care: a systematic review. Contemporary nurse. 2020;56(1):34-48.
111	Chen Z, Ding Z, Chen C, Sun Y, Jiang Y, Liu F, et al. Effectiveness of comprehensive geriatric assessment intervention on quality of life, caregiver burden and length of hospital stay: a systematic review and meta-analysis of randomised controlled trials. BMC geriatrics. 2021;21(1):377.
112	Cheng JO, Lo R, Chan F, Woo J. A pilot study on the effectiveness of anticipatory grief therapy for elderly facing the end of life. Journal of Palliative Care. 2010;26(4):261-9.
113	Cheng ST, Chow PK, Yu EC, Chan AC. Leisure activities alleviate depressive symptoms in nursing home residents with very mild or mild dementia. American journal of geriatric psychiatry. 2012;20(10):904-8.

114	Chenoweth L, Forbes I, Fleming R, King MT, Stein-Parbury J, Luscombe G, et al. PerCEN: a cluster randomized controlled trial of person-centered residential care and environment for people with dementia. International psychogeriatrics. 2014;26(7):1147-60.
115	Chenoweth L, King MT, Jeon YH, Brodaty H, Stein-Parbury J, Norman R, et al. Caring for Aged Dementia Care Resident Study (CADRES) of person-centred care, dementia-care mapping, and usual care in dementia: a cluster-randomised trial. The lancet Neurology. 2009;8(4):317-25.
116	Cheung DSK, Lai CKY, Wong FKY, Leung MCP. Is music-with-movement intervention better than music listening and social activities in alleviating agitation of people with moderate dementia? A randomized controlled trial. Dementia (London, England). 2020;19(5):1413-25.
117	Chevalley T, Hoffmeyer P, Bonjour JP, Rizzoli R. Early serum IGF-I response to oral protein supplements in elderly women with a recent hip fracture. Clinical nutrition (Edinburgh, Scotland). 2010;29(1):78-83.
118	Chi I, Chou KL, Kwan CW, Lam EKF, Lam TP. Use of the Minimum Data Set--Home Care: a cluster randomized controlled trial among the Chinese older adults. Aging & mental health. 2006;10(1):33-9.
119	Chiang FY, Chen JR, Lee WJ, Yang SC. Effects of Milk or Soy Milk Combined with Mild Resistance Exercise on the Muscle Mass and Muscle Strength in Very Old Nursing Home Residents with Sarcopenia. Foods. 2021;10(11):26.
120	Chong CP, van Gaal WJ, Ryan JE, Profitis K, Savige J, Lim WK. Does cardiology intervention improve mortality for post-operative troponin elevations after emergency orthopaedic-geriatric surgery? A randomised controlled study. Injury. 2012;43(7):1193-8.
121	Chou C-P, Chen K-M, Tung H-T, Belcastro F, Hsu H-F. Physical fitness and frailty status of frail older adults in long-term care facilities after acupunch exercises: A cluster-randomized controlled trial. Experimental gerontology. 2022;163:111799.
122	Clark PG, Blissmer BJ, Greene GW, Lees FD, Riebe DA, Stamm KE. Maintaining exercise and healthful eating in older adults: the SENIOR project II: study design and methodology. Contemporary Clinical Trials. 2011;32(1):129-39.
123	Clegg A. The Home-based Older People's Exercise (HOPE) trial : a pilot randomised controlled trial of a home-based exercise intervention for older people with frailty. Age and Ageing. 2014.
124	Clegg A, Barber S, Young J, Iliffe S, Forster A. The Home-based Older People's Exercise (HOPE) trial: a pilot randomised controlled trial of a home-based exercise intervention for older people with frailty. Age and ageing. 2014;43(5):687-95.
125	Cockayne S, Pighills A, Adamson J, Fairhurst C, Crossland S, Drummond A, et al. Home environmental assessments and modification delivered by occupational therapists to reduce falls in people aged 65 years and over: the OTIS RCT. Health Technology Assessment (Winchester, England). 2021;25(46):1-118.
126	Cohen SM, Volandes AE, Shaffer ML, Hanson LC, Habtemariam D, Mitchell SL. Concordance Between Proxy Level of Care Preference and Advance Directives Among Nursing Home Residents With Advanced Dementia: a Cluster Randomized Clinical Trial. Journal of pain and symptom management. 2019;57(1):37-46.e1.
127	Cohen-Mansfield J, Golander H, Iecovich E, Jensen B. Social Engagement Care for Frail Older Persons: Desire for It and Provision by Live-In Migrant Caregivers. The journals of gerontology Series B, Psychological sciences and social sciences. 2019;74(6):1062-71.
128	Cohen-Mansfield J, Marx MS, Dakheel-Ali M, Regier NG, Thein K, Freedman L. Can agitated behavior of nursing home residents with dementia be prevented with the use of standardized stimuli? Journal of the American Geriatrics Society. 2010;58(8):1459-64.
129	Combes S, Gillett K, Norton C, Nicholson CJ. The importance of living well now and relationships: A qualitative study of the barriers and enablers to engaging frail elders with advance care planning. Palliative medicine. 2021;35(6):1137-47.

130	Conneely M, Leahy A, O'Connor M, Gabr A, Okpaje B, Saleh A, et al. A physiotherapy-led transition to home intervention for older adults following Emergency Department discharge: a pilot feasibility randomised controlled trial. Rural and remote health. 2023;23(1):8154.
131	Conneely M, Leahy S, O'Connor M, Corey G, Gabr A, Saleh A, et al. A Physiotherapy-Led Transition to Home Intervention for Older Adults Following Emergency Department Discharge: A Pilot Feasibility Randomised Controlled Trial (ED PLUS). Clinical interventions in aging. 2023;18:1769-88.
132	Conroy SP, Stevens T, Parker SG, Gladman JRF. A systematic review of comprehensive geriatric assessment to improve outcomes for frail older people being rapidly discharged from acute hospital: 'interface geriatrics'. Age and ageing. 2011;40(4):436-43.
133	Cordes T, Zwingmann K, Rudisch J, Voelcker-Rehage C, Wollesen B. Multicomponent exercise to improve motor functions, cognition and well-being for nursing home residents who are unable to walk - A randomized controlled trial. Experimental Gerontology. 2021;153:111484.
134	Crotty M, Killington M, Liu E, Cameron ID, Kurrle S, Kaambwa B, et al. Should we provide outreach rehabilitation to very old people living in Nursing Care Facilities after a hip fracture? A randomised controlled trial. Age & Ageing. 2019;48(3):373-80.
135	Crotty M, Whitehead CH, Wundke R, Giles LC, Ben-Tovim D, Phillips PA. Transitional care facility for elderly people in hospital awaiting a long term care bed: randomised controlled trial. BMJ (Clinical research ed). 2005;331(7525):1110.
136	Cumming RG, Ivers R, Clemson L, Cullen J, Hayes MF, Tanzer M, et al. Improving vision to prevent falls in frail older people: a randomized trial. Journal of the American Geriatrics Society. 2007;55(2):175-81.
137	Dahlkvist E, Engstrom M, Nilsson A. Residents' use and perceptions of residential care facility gardens: A behaviour mapping and conversation study. International journal of older people nursing. 2020;15(1):e12283.
138	Dalleur O, Boland B, Losseau C, Henrard S, Wouters D, Speybroeck N, et al. Reduction of potentially inappropriate medications using the STOPP criteria in frail older inpatients: a randomised controlled study. Drugs & aging. 2014;31(4):291-8.
139	Damluji AA, Cohen ER, Moscucci M, Myerburg RJ, Cohen MG, Brooks MM, et al. Insulin provision therapy and mortality in older adults with diabetes mellitus and stable ischemic heart disease: Insights from BARI-2D trial. International Journal of Cardiology. 2017;241:35-40.
140	Dangas GD, Lefèvre T, Kupatt C, Tchetche D, Schäfer U, Dumonteil N, et al. Bivalirudin Versus Heparin Anticoagulation in Transcatheter Aortic Valve Replacement: the Randomized BRAVO-3 Trial. Journal of the American College of Cardiology. 2015;66(25):2860-8.
141	Daniels R, van Rossum E, de Witte L, Kempen GIJM, van den Heuvel W. Interventions to prevent disability in frail community-dwelling elderly: a systematic review. BMC health services research. 2008;8:278.
142	Darby J, Williamson T, Logan P, Gladman J. Comprehensive geriatric assessment on an acute medical unit: a qualitative study of older people's and informal carer's perspectives of the care and treatment received. Clinical rehabilitation. 2017;31(1):126-34.
143	Daryanti Saragih I, Yang YP, Saragih IS, Batubara SO, Lin CJ. Effects of resistance bands exercise for frail older adults: A systematic review and meta-analysis of randomised controlled studies. Journal of Clinical Nursing (John Wiley & Sons, Inc). 2022;31(1/2):43-61.
144	Davidson T, Lindelöf A, Wallén T. Point-of-care monitoring of warfarin treatment in community dwelling elderly: a randomised controlled study. Journal of Telemedicine and Telecare. 2015;21(5):298-301.
145	Day LW, Lin L, Somsouk M. Adverse events in older patients undergoing ERCP: a systematic review and meta-analysis. Endoscopy International Open. 2014;2(1):E28-36.

146	de Asteasu MLS, Martinez-Velilla N, Zambom-Ferraresi F, Casas-Herrero A, Cadore EL, Galbete A, et al. Assessing the impact of physical exercise on cognitive function in older medical patients during acute hospitalization: Secondary analysis of a randomized trial. Plos Medicine. 2019;16(7).
147	de Asteasu MLS, Martinez-Velilla N, Zambom-Ferraresi F, Casas-Herrero A, Lucia A, Galbete A, et al. Physical Exercise Improves Function in Acutely Hospitalized Older Patients: Secondary Analysis of a Randomized Clinical Trial. Journal of the American Medical Directors Association. 2019;20(7):866-73.
148	de Bruin ED, Murer K. Effect of additional functional exercises on balance in elderly people. Clinical rehabilitation. 2007;21(2):112-21.
149	De Coninck L, Bekkering GE, Bouckaert L, Declercq A, Graff MJL, Aertgeerts B. Home- and Community-Based Occupational Therapy Improves Functioning in Frail Older People: A Systematic Review. Journal of the American Geriatrics Society. 2017;65(8):1863-9.
150	de Labra C, Guimaraes-Pinheiro C, Maseda A, Lorenzo T, Millan-Calenti JC. Effects of physical exercise interventions in frail older adults: a systematic review of randomized controlled trials. BMC geriatrics. 2015;15:154.
151	de Souto Barreto P, Cesari M, Denormandie P, Armaingaud D, Vellas B, Rolland Y. Exercise or Social Intervention for Nursing Home Residents with Dementia: a Pilot Randomized, Controlled Trial. Journal of the American Geriatrics Society. 2017;65(9):E123-E9.
152	de Visme V, Picart F, Le Jouan R, Legrand A, Savry C, Morin V, et al. Combined lumbar and sacral plexus block compared with plain bupivacaine spinal anesthesia for hip fractures in the elderly. Regional Anesthesia & Pain Medicine. 2000;25(2):158-62.
153	De Visschere L, de Baat C, Schols JM, Deschepper E, Vanobbergen J. Evaluation of the implementation of an 'oral hygiene protocol' in nursing homes: a 5-year longitudinal study. Community dentistry and oral epidemiology. 2011;39(5):416-25.
154	De Vriendt P, Peersman W, Florus A, Verbeke M, Van de Velde D. Improving Health Related Quality of Life and Independence in Community Dwelling Frail Older Adults through a Client-Centred and Activity-Oriented Program. A Pragmatic Randomized Controlled Trial. The journal of nutrition, health & aging. 2016;20(1):35-40.
155	de Vries OJ, Peeters GMEEG, Elders PJM, Muller M, Knol DL, Danner SA, et al. Multifactorial intervention to reduce falls in older people at high risk of recurrent falls: a randomized controlled trial. Archives of internal medicine. 2010;170(13):1110-7.
156	Dechamps A, Alban R, Jen J, Decamps A, Traissac T, Dehail P. Individualized Cognition-Action intervention to prevent behavioral disturbances and functional decline in institutionalized older adults: a randomized pilot trial. International journal of geriatric psychiatry. 2010;25(8):850-60.
157	Dechamps A, Diolez P, Thiaudière E, Tulon A, Onifade C, Vuong T, et al. Effects of exercise programs to prevent decline in health-related quality of life in highly deconditioned institutionalized elderly persons: a randomized controlled trial. Archives of internal medicine. 2010;170(2):162-9.
158	den Uijl LC, Kremer S, Jager G, van der Stelt AJ, de Graaf C, Gibson P, et al. That's why I take my ONS. Means-end chain as a novel approach to elucidate the personally relevant factors driving ONS consumption in nutritionally frail elderly users. Appetite. 2015;89:33-40.
159	Deng Y, Fang Y, Li H, Chen J, An J, Qiao S, et al. A preoperative whey protein and glucose drink before hip fracture surgery in the aged improves symptomatic and metabolic recovery. Asia Pacific journal of clinical nutrition. 2020;29(2):234-8.
160	Deschodt M, Flamaing J, Haentjens P, Boonen S, Milisen K. Impact of geriatric consultation teams on clinical outcome in acute hospitals: a systematic review and meta-analysis. BMC medicine. 2013;11:48.

161	DeSure AR, Peterson K, Gianan FV, Pang L. An exercise program to prevent falls in institutionalized elderly with cognitive deficits: a crossover pilot study. Hawai'i journal of medicine & public health. 2013;72(11):391-5.
162	Di Pollina L, Guessous I, Petoud V, Combescure C, Buchs B, Schaller P, et al. Integrated care at home reduces unnecessary hospitalizations of community-dwelling frail older adults: a prospective controlled trial. BMC Geriatrics. 2017;17(53).
163	Díaz-Valenzuela A, García-Fernández FP, Carmona Fernández P, Valle Cañete MJ, Pancorbo-Hidalgo PL. Effectiveness and safety of olive oil preparation for topical use in pressure ulcer prevention: multicentre, controlled, randomised, and double-blinded clinical trial. International wound journal. 2019;16(6):1314-22.
164	Dickson VV, Blustein J, Weinstein B, Goldfeld K, Radcliffe K, Burlingame M, et al. Providing Hearing Assistance to Veterans in the Emergency Department: a Qualitative Study. Journal of emergency nursing. 2022;48(3):266-77.
165	Dorresteijn TAC, Zijlstra GAR, Ambergen AW, Delbaere K, Vlaeyen JWS, Kempen GIJM. Effectiveness of a home-based cognitive behavioral program to manage concerns about falls in community-dwelling, frail older people: results of a randomized controlled trial. BMC geriatrics. 2016;16:2.
166	Dozeman E, van Schaik DJ, van Marwijk HW, Stek ML, Beekman AT, van der Horst HE. Feasibility and effectiveness of activity-scheduling as a guided self-help intervention for the prevention of depression and anxiety in residents in homes for the elderly: a pragmatic randomized controlled trial. International Psychogeriatrics. 2011;23(6):969-78.
167	Dozeman E, van Schaik DJF, van Marwijk HWJ, Stek NL, Beekman ATF, van der Horst HE, et al. Feasibility and effectiveness of activity-scheduling as a guided self-help intervention for the prevention of depression and anxiety in residents in homes for the elderly: a pragmatic randomized controlled trial. International Psychogeriatrics. 2011;23(6):1p-p.
168	Drahota AK. Pilot cluster randomised controlled trial of flooring to reduce injuries from falls in wards for older people. Age and Ageing. 2013.
169	DuMontier C, Uno H, Hshieh T, Zhou G, Chen R, Magnavita ES, et al. Randomized controlled trial of geriatric consultation versus standard care in older adults with hematologic malignancies. Haematologica. 2022;107(5):1172-80.
170	Eckerblad J, Theander K, Ekdahl AW, Jaarsma T. Symptom trajectory and symptom burden in older people with multimorbidity, secondary outcome from the RCT AGe-FIT study. Journal of advanced nursing. 2016;72(11):2773-83.
171	Edmans J, Bradshaw L, Franklin M, Gladman J, Conroy S. Specialist geriatric medical assessment for patients discharged from hospital acute assessment units: randomised controlled trial. BMJ (Clinical research ed). 2013;347:f5874.
172	Edwards KS, Chow EKH, Dao C, Hossepian D, Johnson AG, Desai M, et al. Impact of cognitive behavioral therapy on depression symptoms after transcatheter aortic valve replacement: a randomized controlled trial. International journal of cardiology. 2020;321:61-8.
173	Ekdahl AW, Alwin J, Eckerblad J, Husberg M, Jaarsma T, Mazya AL, et al. Long-Term Evaluation of the Ambulatory Geriatric Assessment: A Frailty Intervention Trial (AGe-FIT): Clinical Outcomes and Total Costs After 36 Months. Journal of the American Medical Directors Association. 2016;17(3):263-8.
174	Ekdahl AW, Wirehn A-B, Alwin J, Jaarsma T, Unosson M, Husberg M, et al. Costs and Effects of an Ambulatory Geriatric Unit (the AGe-FIT Study): A Randomized Controlled Trial. Journal of the American Medical Directors Association. 2015;16(6):497-503.
175	Eklund K, Wilhelmson K, Gustafsson H, Landahl S, Dahlin-Ivanoff S. One-year outcome of frailty indicators and activities of daily living following the randomised controlled trial: "Continuum of care for frail older people". BMC geriatrics. 2013;13:76.

176	El Alili M, Smaling HJA, Joling KJ, Achterberg WP, Francke AL, Bosmans JE, et al. Cost-effectiveness of the Namaste care family program for nursing home residents with advanced dementia in comparison with usual care: a cluster-randomized controlled trial. BMC health services research. 2020;20(1):831.
177	Elia M, Parsons EL, Cawood AL, Smith TR, Stratton RJ. Cost-effectiveness of oral nutritional supplements in older malnourished care home residents. Clinical nutrition (Edinburgh, Scotland). 2018;37(2):651-8.
178	Ellis G, Gardner M, Tsiachristas A, Langhorne P, Burke O, Harwood RH, et al. Comprehensive geriatric assessment for older adults admitted to hospital. The Cochrane database of systematic reviews. 2017;9:CD006211.
179	Ellis G, Whitehead MA, O'Neill D, Langhorne P, Robinson D. Comprehensive geriatric assessment for older adults admitted to hospital. The Cochrane database of systematic reviews. 2011(7):CD006211.
180	Ellis G, Whitehead MA, Robinson D, O'Neill D, Langhorne P. Comprehensive geriatric assessment for older adults admitted to hospital: meta-analysis of randomised controlled trials. BMJ (Clinical research ed). 2011;343:d6553.
181	Erdal A, Flo E, Aarsland D, Ballard C, Slettebo DD, Husebo BS. Efficacy and Safety of Analgesic Treatment for Depression in People with Advanced Dementia: randomised, Multicentre, Double-Blind, Placebo-Controlled Trial (DEP.PAIN.DEM). Drugs & aging. 2018;35(6):545-58.
182	Ericsson I, Ekdahl AW, Hellstrom I. "To be seen" - older adults and their relatives' care experiences given by a geriatric mobile team (GerMoT). BMC geriatrics. 2021;21(1):636.
183	Ersek M, Turner JA, McCurry SM, Gibbons L, Kraybill BM. Efficacy of a self-management group intervention for elderly persons with chronic pain. Clinical Journal of Pain. 2003;19(3):156-67.
184	Etherton-Beer C, Page A, Naganathan V, Potter K, Comans T, Hilmer SN, et al. Deprescribing to optimise health outcomes for frail older people: a double-blind placebo-controlled randomised controlled trial-outcomes of the Opti-med study. Age and ageing. 2023;52(5).
185	Fairhall N, Sherrington C, Cameron ID, Kurrle SE, Lord SR, Lockwood K, et al. A multifactorial intervention for frail older people is more than twice as effective among those who are compliant: complier average causal effect analysis of a randomised trial. Journal of physiotherapy. 2017;63(1):40-4.
186	Fairhall N, Sherrington C, Kurrle SE, Lord SR, Lockwood K, Cameron ID. Effect of a multifactorial interdisciplinary intervention on mobility-related disability in frail older people: randomised controlled trial. BMC medicine. 2012;10:120.
187	Fairhall N, Sherrington C, Kurrle SE, Lord SR, Lockwood K, Howard K, et al. Economic evaluation of a multifactorial, interdisciplinary intervention versus usual care to reduce frailty in frail older people. Journal of the American Medical Directors Association. 2015;16(1):41-8.
188	Fairhall N, Sherrington C, Lord SR, Kurrle SE, Langron C, Lockwood K, et al. Effect of a multifactorial, interdisciplinary intervention on risk factors for falls and fall rate in frail older people: a randomised controlled trial. Age and ageing. 2014;43(5):616-22.
189	Fairhall NJ, Dyer SM, Mak JCS, Diong J, Kwok WS, Sherrington C. Interventions for improving mobility after hip fracture surgery in adults. Cochrane Database of Systematic Reviews. 2022(9).
190	Fiatarone MA, O'Neill EF, Ryan ND. Exercise training and nutritional supplementation for physical frailty in very elderly people. The New England Journal of Medicine. 1994;330(25).
191	Figueiro MG, Plitnick B, Roohan C, Sahin L, Kalsher M, Rea MS. Effects of a Tailored Lighting Intervention on Sleep Quality, Rest-Activity, Mood, and Behavior in Older Adults With Alzheimer Disease and Related Dementias: A Randomized Clinical Trial. Journal of clinical sleep medicine : JCSM : official publication of the American Academy of Sleep Medicine. 2019;15(12):1757-67.

192	Fiorini L, De Mul M, Fabbricotti I, Limosani R, Vitanza A, D'Onofrio G, et al. Assistive robots to improve the independent living of older persons: results from a needs study. Disability & Rehabilitation Assistive Technology. 2021;16(1):92-102.
193	Flamaing J, Knockaert D, Meijers B, Verhaegen J, Peetermans WE. Sequential therapy with cefuroxime and cefuroxime-axetil for community-acquired lower respiratory tract infection in the oldest old. Aging-Clinical & Experimental Research. 2008;20(1):81-6.
194	Fletcher AE, Price GM, Ng ESW, Stirling SL, Bulpitt CJ, Breeze E, et al. Population-based multidimensional assessment of older people in UK general practice: a cluster-randomised factorial trial. Lancet (London, England). 2004;364(9446):1667-77.
195	Flicker L, MacInnis RJ, Stein MS, Scherer SC, Mead KE, Nowson CA, et al. Should older people in residential care receive vitamin D to prevent falls? Results of a randomized trial. Journal of the American Geriatrics Society. 2005;53(11):1881-8.
196	Flood C, Mugford M, Stewart S, Harvey I, Poland F, Lloyd-Smith W. Occupational therapy compared with social work assessment for older people. An economic evaluation alongside the CAMELOT randomised controlled trial. Age and ageing. 2005;34(1):47-52.
197	Foley C, Bloomer M, Hutchinson AM. Factors that influence intensive care admission decisions for older people: A systematic review. Australian Critical Care. 2022;07:07.
198	Fox MT, Sidani S, Persaud M, Tregunno D, Maimets I, Brooks D, et al. Acute care for elders components of acute geriatric unit care: systematic descriptive review. Journal of the American Geriatrics Society. 2013;61(6):939-46.
199	Fox P, Richardson J, McInnes B, Tait D, Bedard M. Effectiveness of a bed positioning program for treating older adults with knee contractures who are institutionalized. Physical therapy. 2000;80(4):363-72.
200	Frihagen F, Nordsletten L, Madsen JE. Hemiarthroplasty or internal fixation for intracapsular displaced femoral neck fractures: randomised controlled trial. BMJ (Clinical research ed). 2007;335(7632):1251-4.
201	Frost R, Belk C, Jovicic A, Ricciardi F, Kharicha K, Gardner B, et al. Health promotion interventions for community-dwelling older people with mild or pre-frailty: a systematic review and meta-analysis. BMC Geriatrics. 2017;17(157).
202	Furtado GE, Carvalho HM, Loureiro M, Patricio M, Uba-Chupel M, Colado JC, et al. Chair-based exercise programs in institutionalized older women: Salivary steroid hormones, disabilities and frailty changes. Experimental gerontology. 2020;130:110790.
203	Furtado GE, Chupel MU, Minuzzi LG, Rama L, Colado JC, Hogervorst E, et al. The Mediating Effect of Different Exercise Programs on the Immune Profile of Frail Older Women with Cognitive Impairment. Current pharmaceutical design. 2020;26(9):906-15.
204	Gagnon AJ, Schein C, McVey L, Bergman H. Randomized controlled trial of nurse case management of frail older people. Journal of the American Geriatrics Society. 1999;47(9):1118-24.
205	Galik E, Resnick B, Lerner N, Hammersla M, Gruber-Baldini AL. Function Focused Care for Assisted Living Residents With Dementia. Gerontologist. 2015;55 Suppl 1(Suppl 1):S13-26.
206	García-Gollarte F, Baleriola-Júlvez J, Ferrero-López I, Cuenllas-Díaz Á, Cruz-Jentoft AJ. An educational intervention on drug use in nursing homes improves health outcomes resource utilization and reduces inappropriate drug prescription. Journal of the American Medical Directors Association. 2014;15(12):885-91.
207	Gardner B, Jovicic A, Belk C, Kharicha K, Iliffe S, Manthorpe J, et al. Specifying the content of home-based health behaviour change interventions for older people with frailty or at risk of frailty: an exploratory systematic review. BMJ open. 2017;7(2):e014127.
208	Gardner M, Shepperd S, Godfrey M, Makela P, Tsiachristas A, Singh-Mehta A, et al. Comprehensive Geriatric Assessment in hospital and hospital-at-home settings: a mixed-methods study. 2019.

209	Garrard JW, Cox NJ, Dodds RM, Roberts HC, Sayer AA. Comprehensive geriatric assessment in primary care: a systematic review. Aging clinical and experimental research. 2020;32(2):197-205.
210	Gebhard D, Mess F. Feasibility and Effectiveness of a Biography-Based Physical Activity Intervention in Institutionalized People With Dementia: quantitative and Qualitative Results From a Randomized Controlled Trial. Journal of aging and physical activity. 2022;30(2):237-51.
211	Gedde MH, Husebo BS, Mannseth J, Kjome RLS, Naik M, Berge LI. Less Is More: the Impact of Deprescribing Psychotropic Drugs on Behavioral and Psychological Symptoms and Daily Functioning in Nursing Home Patients. Results From the Cluster-Randomized Controlled COSMOS Trial. American journal of geriatric psychiatry. 2021;29(3):304-15.
212	George DR, Singer ME. Intergenerational volunteering and quality of life for persons with mild to moderate dementia: results from a 5-month intervention study in the United States. American journal of geriatric psychiatry. 2011;19(4):392-6.
213	Giamarellou H, Dontas AS, Zorbas P, Staszewska-Pistoni M, Xirouchaki E, Petrikkos G. Asymptomatic bacteriuria in freely voiding elderly subjects. Long-term continuous vs pulse treatment with ofloxacin. Clinical drug investigation. 1998;15(3):187-95.
214	Gibson G, Quirke M, Lovatt M. The role of environmental design in enabling intergenerational support for people with dementia: what lessons can we learn from Japan? Working with Older People. 2022;26(3):226-37.
215	Gill TM. A program to prevent functional decline in physically frail, elderly persons who live at home. New England Journal of Medicine. 2002;347(14).
216	Gill TM, Bhasin S, Reuben DB, Latham NK, Araujo K, Ganz DA, et al. Effect of a Multifactorial Fall Injury Prevention Intervention on Patient Well-Being: the STRIDE Study. Journal of the American Geriatrics Society. 2021;69(1):173-9.
217	Gillespie LD, Robertson MC, Gillespie WJ, Sherrington C, Gates S, Clemson LM, et al. Interventions for preventing falls in older people living in the community. Cochrane Database of Systematic Reviews. 2012(9):CD007146.
218	Gillis K, Tency I, Roelant E, Laureys S, Devriendt H, Lips D. Skin hydration in nursing home residents using disposable bed baths. Geriatric nursing (New York, NY). 2016;37(3):175-9.
219	Ginde AA, Blatchford P, Breese K, Zarrabi L, Linnebur SA, Wallace JI, et al. High-Dose Monthly Vitamin D for Prevention of Acute Respiratory Infection in Older Long-Term Care Residents: a Randomized Clinical Trial. Journal of the American Geriatrics Society. 2017;65(3):496-503.
220	Gitlin LN, Winter L, Dennis MP, Hauck WW. Variation in response to a home intervention to support daily function by age, race, sex, and education. Journals of Gerontology Series A-Biological Sciences & Medical Sciences. 2008;63(7):745-50.
221	Gladman J, Harwood R, Conroy S, Logan P, Elliott R, Jones R, et al. Medical Crises in Older People: cohort study of older people attending acute medical units, developmental work and randomised controlled trial of a specialist geriatric medical intervention for high-risk older people; cohort study of older people with mental health problems admitted to hospital, developmental work and randomised controlled trial of a specialist medical and mental health unit for general hospital patients with delirium and dementia; and cohort study of residents of care homes and interview study of health-care provision to residents of care homes. 2015.
222	Goldberg SE. Care in specialist medical and mental health unit compared with standard care for older people with cognitive impairment admitted to general hospital : randomised controlled trial (NIHR TEAM trial). British Medical Journal. 2013.
223	Goldberg SE, Bradshaw LE, Kearney FC. Care in specialist medical and mental health unit compared with standard care for older people with cognitive impairment admitted to general hospital. British Medical Journal. 2013.

224	Gomes GCV, Simoes MdS, Lin SM, Bacha JMR, Viveiro LAP, Varise EM, et al. Feasibility, safety, acceptability, and functional outcomes of playing Nintendo Wii Fit PlusTM for frail older adults: A randomized feasibility clinical trial. Maturitas. 2018;118:20-8.
225	Gosney MA, Hammond MF, Shenkin A, Allsup S. Effect of micronutrient supplementation on mood in nursing home residents. Gerontology. 2008;54(5):292-9.
226	Granbom M, Kristensson J, Sandberg M. Effects on leisure activities and social participation of a case management intervention for frail older people living at home: a randomised controlled trial. Health & social care in the community. 2017;25(4):1416-29.
227	Greenspan AI, Wolf SL, Kelley ME, O'Grady M. Tai chi and perceived health status in older adults who are transitionally frail: a randomized controlled trial. Physical therapy. 2007;87(5):525-35.
228	Greenspan SL, Perera S, Ferchak MA, Nace DA, Resnick NM. Efficacy and safety of single-dose zoledronic acid for osteoporosis in frail elderly women: a randomized clinical trial. JAMA internal medicine. 2015;175(6):913-21.
229	Gurwitz JH, Field TS, Rochon P, Judge J, Harrold LR, Bell CM, et al. Effect of computerized provider order entry with clinical decision support on adverse drug events in the long-term care setting. Journal of the American Geriatrics Society. 2008;56(12):2225-33.
230	Haider S, Grabovac I, Winzer E, Kapan A, Schindler KE, Lackinger C, et al. Change in inflammatory parameters in prefrail and frail persons obtaining physical training and nutritional support provided by lay volunteers: A randomized controlled trial. PloS one. 2017;12(10):e0185879.
231	Haines TP, Hill KD, Bennell KL, Osborne RH. Patient education to prevent falls in subacute care. Clinical rehabilitation. 2006;20(11):970-9.
232	Hajibandeh S, Hajibandeh S, Antoniou GA, Antoniou SA. Meta-analysis of mortality risk in octogenarians undergoing emergency general surgery operations. Surgery. 2021;169(6):1407-16.
233	Hall A, Boulton E, Stanmore E. Older adults' perceptions of wearable technology hip protectors: implications for further research and development strategies. Disability and rehabilitation Assistive technology. 2019;14(7):663-8.
234	Hall S, Kolliakou A, Petkova H, Froggatt K, Higginson IJ. Interventions for improving palliative care for older people living in nursing care homes. Cochrane Database of Systematic Reviews. 2011(3).
235	Hamdorf PA, Penhall RK. Walking with its training effects on the fitness and activity patterns of 79-91 year old females. Australian & New Zealand Journal of Medicine. 1999;29(1):22-8.
236	Han CY, Crotty M, Thomas S, Cameron ID, Whitehead C, Kurrle S, et al. Effect of Individual Nutrition Therapy and Exercise Regime on Gait Speed, Physical Function, Strength and Balance, Body Composition, Energy and Protein, in Injured, Vulnerable Elderly: a Multisite Randomized Controlled Trial (INTERACTIVE). Nutrients. 2021;13(9).
237	Han CY, Miller M, Yaxley A, Baldwin C, Woodman R, Sharma Y. Effectiveness of combined exercise and nutrition interventions in prefrail or frail older hospitalised patients: a systematic review and meta-analysis. BMJ open. 2020;10(12):e040146.
238	Handeland M, Grude N, Torp T, Slimestad R. Black chokeberry juice (Aronia melanocarpa) reduces incidences of urinary tract infection among nursing home residents in the long term--a pilot study. Nutrition research (New York, NY). 2014;34(6):518-25.
239	Hanson EJ, Clarke A. The role of telematics in assisting family carers and frail older people at home. Health and Social Care in the Community. 2000;8(2):129-37.
240	Hanson LC, Kistler CE, Lavin K, Gabriel SL, Ernecoff NC, Lin FC, et al. Triggered Palliative Care for Late-Stage Dementia: a Pilot Randomized Trial. Journal of pain and symptom management. 2019;57(1):10-9.

241	Hanson LC, Zimmerman S, Song MK, Lin FC, Rosemond C, Carey TS, et al. Effect of the Goals of Care Intervention for Advanced Dementia: a Randomized Clinical Trial. JAMA internal medicine. 2017;177(1):24-31.
242	Harvey P, Storer M, Berlowitz DJ, Jackson B, Hutchinson A, Lim WK. Feasibility and impact of a post-discharge geriatric evaluation and management service for patients from residential care: the Residential Care Intervention Program in the Elderly (RECIPE). BMC geriatrics. 2014;14:48.
243	Hashimoto M, Kato S, Tanabe Y, Katakura M, Mamun AA, Ohno M, et al. Beneficial effects of dietary docosahexaenoic acid intervention on cognitive function and mental health of the oldest elderly in Japanese care facilities and nursing homes. Geriatrics & gerontology international. 2017;17(2):330-7.
244	Hassan BH, Hewitt J, Keogh JW, Bermeo S, Duque G, Henwood TR. Impact of resistance training on sarcopenia in nursing care facilities: A pilot study. Geriatric Nursing. 2016;37(2):116-21.
245	Hassan BH, Hewitt J, Keogh JWL, Bermeo S, Duque G, Henwood TR. Impact of resistance training on sarcopenia in nursing care facilities: A pilot study. Geriatric Nursing. 2016;37(2):116-21.
246	Hauer K, Schwenk M, Zieschang T, Essig M, Becker C, Oster P. Physical training improves motor performance in people with dementia: a randomized controlled trial. Journal of the American Geriatrics Society. 2012;60(1):8-15.
247	Hebert R, Robichaud L, Roy PM, Bravo G, Voyer L. Efficacy of a nurse-led multidimensional preventive programme for older people at risk of functional decline. A randomized controlled trial. Age and ageing. 2001;30(2):147-53.
248	Heesterbeek M, van der Zee EA, van Heuvelen MJG. Feasibility of Three Novel Forms of Passive Exercise in a Multisensory Environment in Vulnerable Institutionalized Older Adults with Dementia. Journal of Alzheimer's disease. 2019;70(3):681-90.
249	Helbostad JL, Sletvold O, Moe-Nilssen R. Home training with and without additional group training in physically frail old people living at home: effect on health-related quality of life and ambulation. Clinical rehabilitation. 2004;18(5):498-508.
250	Hend A, et al. Optimisation of medications used in residential aged care facilities: a systematic review and meta-analysis of randomised controlled trials. BMC Geriatrics. 2020;20(236).
251	Henderson RM, Lovato L, Miller ME, Fielding RA, Church TS, Newman AB, et al. Effect of Statin Use on Mobility Disability and its Prevention in At-risk Older Adults: The LIFE Study. The journals of gerontology Series A, Biological sciences and medical sciences. 2016;71(11):1519-24.
252	Hengstenberg C, Chandrasekhar J, Sartori S, Lefevre T, Mikhail G, Meneveau N, et al. Impact of pre-existing or new-onset atrial fibrillation on 30-day clinical outcomes following transcatheter aortic valve replacement: results from the BRAVO 3 randomized trial. Catheterization and cardiovascular interventions. 2017;90(6):1027-37.
253	Herrmann N, O'Regan J, Ruthirakuhan M, Kiss A, Eryavec G, Williams E, et al. A Randomized Placebo-Controlled Discontinuation Study of Cholinesterase Inhibitors in Institutionalized Patients With Moderate to Severe Alzheimer Disease. Journal of the American Medical Directors Association. 2016;17(2):142-7.
254	Hess JA, Woollacott M, Shivitz N. Ankle force and rate of force production increase following high intensity strength training in frail older adults. Aging clinical and experimental research. 2006;18(2):107-15.
255	Hewitt J, Saing S, Goodall S, Henwood T, Clemson L, Refshauge K. An economic evaluation of the SUNBEAM programme: a falls-prevention randomized controlled trial in residential aged care. Clinical rehabilitation. 2019;33(3):524-34.
256	Hickman LD, Phillips JL, Newton PJ, Halcomb EJ, Al Abed N, Davidson PM. Multidisciplinary team interventions to optimise health outcomes for older people in acute care settings: A systematic review. Archives of gerontology and geriatrics. 2015;61(3):322-9.

257	Hill AM, McPhail SM, Waldron N, Etherton-Beer C, Ingram K, Flicker L, et al. Fall rates in hospital rehabilitation units after individualised patient and staff education programmes: a pragmatic, stepped-wedge, cluster-randomised controlled trial. Lancet (london, england). 2015;385(9987):2592-9.
258	Hjelm M, Holst G, Willman A. The work of case managers as experienced by older persons (75+) with multi-morbidity: a focused ethnography. BMC Geriatrics. 2015;15(168).
259	Hmwe NTT, Browne G, Mollart L, Allanson V, Chan SW-C. Older people's perspectives on use of complementary and alternative medicine and acupressure: A qualitative study. Complementary therapies in clinical practice. 2020;39:101163.
260	Ho L, Malden S, McGill K, Shimonovich M, Frost H, Aujla N, et al. Complex interventions for improving independent living and quality of life amongst community-dwelling older adults: a systematic review and meta-analysis. Age and ageing. 2023;52(7).
261	Ho RT, Fong TC, Sing CY, Lee PH, Leung AB, Chung KS, et al. Managing behavioral and psychological symptoms in Chinese elderly with dementia via group-based music intervention: a cluster randomized controlled trial. Dementia (London, England). 2019;18(7-8):2785-98.
262	Hoedemakers M, Looman WM, Leijten FRM, Czypionka T, Kraus M, Donkers H, et al. Integrated care for frail elderly: a qualitative study of a promising approach in the Netherlands. International Journal of Integrated Care. 2019;19(3):16.
263	Hofmann M, Schober-Halper B, Oesen S, Franzke B, Tschan H, Bachl N, et al. Effects of elastic band resistance training and nutritional supplementation on muscle quality and circulating muscle growth and degradation factors of institutionalized elderly women: the Vienna Active Ageing Study (VAAS). European Journal of Applied Physiology. 2016;116(5):885-97.
264	Holland R, Bond C, Alldred DP, Arthur A, Barton G, Birt L, et al. Evaluation of effectiveness and safety of pharmacist independent prescribers in care homes: cluster randomised controlled trial. BMJ (Clinical research ed). 2023;380:e071883.
265	Holland R, Lenaghan E, Harvey I, Smith R, et al. Does home based medication review keep older people out of hosptal? The HOMER randomised controlled trial. British Medical Journal. 2005;330(7486):293.
266	Holmerova I, Machacova K, Vankova H, Veleta P, Juraskova B, Hrnciarikova D, et al. Effect of the Exercise Dance for Seniors (EXDASE) program on lower-body functioning among institutionalized older adults. Journal of aging and health. 2010;22(1):106-19.
267	Holmes C, Wilkinson D, Dean C, Clare C, El-Okl M, Hensford C, et al. Risperidone and rivastigmine and agitated behaviour in severe Alzheimer's disease: a randomised double blind placebo controlled study. International journal of geriatric psychiatry. 2007;22(4):380-1.
268	Hoogendijk EO, van der Horst HE, van de Ven PM, Twisk JWR, Deeg DJH, Frijters DHM, et al. Effectiveness of a Geriatric Care Model for frail older adults in primary care: Results from a stepped wedge cluster randomized trial. European journal of internal medicine. 2016;28:43-51.
269	Hopkins SA, Bentley A, Phillips V, Barclay S. Advance care plans and hospitalized frail older adults: a systematic review. BMJ supportive & palliative care. 2020;10(2):164-74.
270	Hruda KV, Hicks AL, McCartney N. Training for muscle power in older adults: effects on functional abilities. Canadian journal of applied physiology = Revue canadienne de physiologie appliquee. 2003;28(2):178-89.
271	Hsieh T-J, Su S-C, Chen C-W, Kang Y-W, Hu M-H, Hsu L-L, et al. Individualized home-based exercise and nutrition interventions improve frailty in older adults: a randomized controlled trial. The international journal of behavioral nutrition and physical activity. 2019;16(1):119.
272	Huang HL, Kuo LM, Chen YS, Liang J, Huang HL, Chiu YC, et al. A home-based training program improves caregivers' skills and dementia patients' aggressive behaviors: a randomized controlled trial. American journal of geriatric psychiatry. 2013;21(11):1060-70.

273	Hubbard RE, Bak M, Watts J, Shum D, Lynch A, Peel NM. Enhancing Dignity for Older Inpatients: The Photograph-Next-to-the-Bed Study. Clinical Gerontologist. 2018;41(5):468-73.
274	Huddart R, Hafeez S, Lewis R, McNair H, Syndikus I, Henry A, et al. Clinical Outcomes of a Randomized Trial of Adaptive Plan-of-the-Day Treatment in Patients Receiving Ultra-hypofractionated Weekly Radiation Therapy for Bladder Cancer. International journal of radiation oncology, biology, physics. 2021;110(2):412-24.
275	Humbert P, Fanian F, Lihoreau T, Jeudy A, Pierard GE. Bateman purpura (dermatoporosis): a localized scurvy treated by topical vitamin C - double-blind randomized placebo-controlled clinical trial. Journal of the European Academy of Dermatology and Venereology : JEADV. 2018;32(2):323-8.
276	Husebo BS, Ballard C, Cohen-Mansfield J, Seifert R, Aarsland D. The response of agitated behavior to pain management in persons with dementia. American journal of geriatric psychiatry. 2014;22(7):708-17.
277	Huss A, Stuck AE, Rubenstein LZ, Egger M, Clough-Gorr KM. Multidimensional Preventive Home Visit Programs for Community-Dwelling Older Adults: A Systematic Review and Meta-Analysis of Randomized Controlled Trials. Journals of Gerontology: Series A: Biological Sciences and Medical Sciences. 2008;63(3):298-307.
278	Hutcheon SD, Gillespie ND, Crombie IK, Struthers AD, McMurdo ME. Perindopril improves six minute walking distance in older patients with left ventricular systolic dysfunction: a randomised double blind placebo controlled trial. Heart. 2002;88(4):373-7.
279	Hutcheon SD, Gillespie ND, Crombie IK, Struthers AD, McMurdo MET, Hutcheon SD, et al. Perindopril improves six minute walking distance in older patients with left ventricular systolic dysfunction: a randomised double blind placebo controlled trial. Heart. 2002;88(4):373-7.
280	Hvid LG, Strotmeyer ES, Skjødt M, Magnussen LV, Andersen M, Caserotti P. Voluntary muscle activation improves with power training and is associated with changes in gait speed in mobility-limited older adults - A randomized controlled trial. Experimental gerontology. 2016;80:51-6.
281	Ipsen JA, Pedersen LT, Draborg E, Bruun IH, Abrahamsen C, Viberg B. Cost-Effectiveness of Physical Rehabilitation and Care of Older Home-Dwelling Persons after Hip Fracture: A Systematic Review and Narrative Synthesis. Journal of rehabilitation medicine. 2022;54:jrm00351.
282	Iqbal U, Nawaz A, Ahmed Z, Kamal F, Lee-Smith W, Khan MA, et al. Safety of endoscopic mucosal resection of large colonic polyps in elderly patients: a systematic review and meta-analysis. Annals of Gastroenterology. 2022;35(4):420-6.
283	Ish-Shalom S, Segal E, Salganik T, Raz B, Bromberg IL, Vieth R. Comparison of daily, weekly, and monthly vitamin D3 in ethanol dosing protocols for two months in elderly hip fracture patients. Journal of clinical endocrinology and metabolism. 2008;93(9):3430-5.
284	Ito I, Kadowaki S, Tanabe N, Haruna A, Kase M, Yasutomo Y, et al. Tazobactam/piperacillin for moderate-to-severe pneumonia in patients with risk for aspiration: comparison with imipenem/cilastatin. Pulmonary pharmacology & therapeutics. 2010;23(5):403-10.
285	Ito T, Meguro K, Akanuma K, Ishii H, Mori E. A randomized controlled trial of the group reminiscence approach in patients with vascular dementia. Dementia and geriatric cognitive disorders. 2007;24(1):48-54.
286	Izumi M, Takeuchi K, Ganaha S, Akifusa S, Yamashita Y. Effects of oral care with tongue cleaning on coughing ability in geriatric care facilities: a randomised controlled trial. Journal of oral rehabilitation. 2016;43(12):953-9.
287	Jacobson BH, Thompson B, Wallace T, Brown L, Rial C. Independent static balance training contributes to increased stability and functional capacity in community-dwelling elderly people: a randomized controlled trial. Clinical rehabilitation. 2011;25(6):549-56.

288	Jadczak AD, Dollard J, Mahajan N, Visvanathan R. The perspectives of pre-frail and frail older people on being advised about exercise: a qualitative study. Family practice. 2018;35(3):330-5.
289	Janlov A-C, Hallberg IR, Petersson K. Older persons' experience of being assessed for and receiving public home help: do they have any influence over it? Health & social care in the community. 2006;14(1):26-36.
290	Jensen J, Lundin-Olsson L, Nyberg L, Gustafson Y. Fall and injury prevention in older people living in residential care facilities. A cluster randomized trial. Annals of internal medicine. 2002;136(10):733-41.
291	Jensen J, Nyberg L, Rosendahl E, Gustafson Y, Lundin-Olsson L. Effects of a fall prevention program including exercise on mobility and falls in frail older people living in residential care facilities. Aging clinical and experimental research. 2004;16(4):283-92.
292	Jobory A, Rolfson O, Åkesson KE, Arvidsson C, Nilsson I, Rogmark C. Hip precautions not meaningful after hemiarthroplasty due to hip fracture. Cluster-randomized study of 394 patients operated with direct anterolateral approach. Injury. 2019;50(7):1318-23.
293	Jobse I, Liao Y, Bartram M, Delantonio K, Uter W, Stehle P, et al. Compliance of nursing home residents with a nutrient- and energy-dense oral nutritional supplement determines effects on nutritional status. Journal of nutrition, health & aging. 2015;19(3):356-64.
294	Joling KJ, van Hout HP, van't Veer-Tazelaar PJ, van der Horst HE, Cuijpers P, van de Ven PM, et al. How effective is bibliotherapy for very old adults with subthreshold depression? A randomized controlled trial. American Journal of Geriatric Psychiatry. 2011;19(3):256-65.
295	Joling KJ, van Hout HP, Van't Veer-Tazelaar PJ, van der Horst HE, Cuijpers P, van de Ven PM, et al. How effective is bibliotherapy for very old adults with subthreshold depression? A randomized controlled trial. American Journal of Geriatric Psychiatry. 2011;19(3):256-65.
296	Jones C, Moyle W, Murfield J, Draper B, Shum D, Beattie E, et al. Does Cognitive Impairment and Agitation in Dementia Influence Intervention Effectiveness? Findings From a Cluster-Randomized-Controlled Trial With the Therapeutic Robot, PARO. Journal of the American Medical Directors Association. 2018;19(7):623-6.
297	Jonker AAGC, Comijs HC, Knipscheer KCPM. Benefits for elders with vulnerable health from the Chronic Disease Self-management Program (CDSMP) at short and longer term. BMC Geriatrics. 2015;15(101).
298	Juggan S, Ponnamreddy PK, Reilly CA, Dodge SE, Gilstrap LG, Zeitler EP. Comparative effectiveness of cardiac resynchronization therapy in older patients with heart failure: Systematic review and meta-analysis. Journal of Cardiac Failure. 2022;28(3):443-52.
299	Kallio EL, Öhman H, Hietanen M, Soini H, Strandberg TE, Kautiainen H, et al. Effects of Cognitive Training on Cognition and Quality of Life of Older Persons with Dementia. Journal of the American Geriatrics Society. 2018;66(4):664-70.
300	Kapan A, Luger E, Haider S, Titze S, Schindler K, Lackinger C, et al. Fear of falling reduced by a lay led home-based program in frail community-dwelling older adults: A randomised controlled trial. Archives of gerontology and geriatrics. 2017;68:25-32.
301	Kapan A, Winzer E, Haider S, Titze S, Schindler K, Lackinger C, et al. Impact of a lay-led home-based intervention programme on quality of life in community-dwelling pre-frail and frail older adults: a randomized controlled trial. BMC geriatrics. 2017;17(1):154.
302	Kerse N, McLean C, Moyes SA, Peri K, Ng T, Wilkinson-Meyers L, et al. The cluster-randomized BRIGHT trial: Proactive case finding for community-dwelling older adults. Annals of family medicine. 2014;12(6):514-24.
303	Kersten H, Molden E, Tolo IK, Skovlund E, Engedal K, Wyller TB. Cognitive effects of reducing anticholinergic drug burden in a frail elderly population: a randomized controlled trial. Journals of gerontology Series A, Biological sciences and medical sciences. 2013;68(3):271-8.

304	Kessler J, Radlinger L, Baur H, Rogan S. Effect of stochastic resonance whole body vibration on functional performance in the frail elderly: a pilot study. Archives of gerontology and geriatrics. 2014;59(2):305-11.
305	Kidd T, Mold F, Jones C, Ream E, Grosvenor W, Sund-Levander M, et al. What are the most effective interventions to improve physical performance in pre-frail and frail adults? A systematic review of randomised control trials. BMC geriatrics. 2019;19(1):184.
306	Kim C-O, Jeong Y, Park Y, Bae J-S, Kwon Y, Cho M, et al. Reinforcement Effects of Social Network Intervention during Nutritional Supplementation in Frail Older Adults. Gerontology. 2021;67(5):620-32.
307	Kim C-O, Lee K-R. Preventive effect of protein-energy supplementation on the functional decline of frail older adults with low socioeconomic status: a community-based randomized controlled study. The journals of gerontology Series A, Biological sciences and medical sciences. 2013;68(3):309-16.
308	Kim H. Interventions for frailty and sarcopenia in community-dwelling elderly women. Nihon Ronen Igakkai zasshi [Japanese journal of geriatrics]. 2012;49(6):726-30.
309	Kim H, Jung Y-I, Kim G-S, Choi H, Park Y-H. Effectiveness of a Technology-Enhanced Integrated Care Model for Frail Older People: A Stepped-Wedge Cluster Randomized Trial in Nursing Homes. The Gerontologist. 2021;61(3):460-9.
310	Kimura K, Momose T, Hasegawa T, Morita T, Misawa T, Motoki H, et al. Early administration of tolvaptan preserves renal function in elderly patients with acute decompensated heart failure. Journal of cardiology. 2016;67(5):399-405.
311	Koeneman MA, Chorus A, Hopman-Rock M, Chinapaw MJ. A novel method to promote physical activity among older adults in residential care: an exploratory field study on implicit social norms. BMC geriatrics. 2017;17(1):8.
312	Koike T, Orito Y, Toyoda H, Tada M, Sugama R, Hoshino M, et al. External hip protectors are effective for the elderly with higher-than-average risk factors for hip fractures. Osteoporosis international. 2009;20(9):1613-20.
313	Kolanowski A, Fick D, Litaker M, Mulhall P, Clare L, Hill N, et al. Effect of Cognitively Stimulating Activities on Symptom Management of Delirium Superimposed on Dementia: a Randomized Controlled Trial. Journal of the American Geriatrics Society. 2016;64(12):2424-32.
314	Kono A, Izumi K, Yoshiyuki N, Kanaya Y, Rubenstein LZ. Effects of an Updated Preventive Home Visit Program Based on a Systematic Structured Assessment of Care Needs for Ambulatory Frail Older Adults in Japan: A Randomized Controlled Trial. The journals of gerontology Series A, Biological sciences and medical sciences. 2016;71(12):1631-7.
315	Kono A, Kanaya Y, Fujita T, Tsumura C, Kondo T, Kushiyama K, et al. Effects of a preventive home visit program in ambulatory frail older people: a randomized controlled trial. The journals of gerontology Series A, Biological sciences and medical sciences. 2012;67(3):302-9.
316	Kosmat H, Vranic A. The Efficacy of a Dance Intervention as Cognitive Training for the Old-Old. Journal of aging and physical activity. 2017;25(1):32-40.
317	Kouzuki M, Taniguchi M, Suzuki T, Nagano M, Nakamura S, Katsumata Y, et al. Effect of monosodium L-glutamate (umami substance) on cognitive function in people with dementia. European journal of clinical nutrition. 2019;73(2):266-75.
318	Kovach CR, Logan BR, Noonan PE, Schlidt AM, Smerz J, Simpson M, et al. Effects of the Serial Trial Intervention on discomfort and behavior of nursing home residents with dementia. American journal of Alzheimer's disease and other dementias. 2006;21(3):147-55.
319	Koyi H, Hillerdal G, Kölbeck KG, Brodin D, Brandén E. P2.03a-013 Chemotherapy is Beneficial for Octogenarians with Non-Small Cell Lung Cancer (NSCLC): Topic: Clinical Trials. Journal of Thoracic Oncology. 2017;12:S895-S.
320	Kraft-Bodi E, Jorgensen MR, Keller MK, Kragelund C, Twetman S. Effect of Probiotic Bacteria on Oral Candida in Frail Elderly. Journal of dental research. 2015;94(9 Suppl):181S-6S.

321	Kristensson J, Ekwall AK, Jakobsson U, Midlov P, Hallberg IR. Case managers for frail older people: a randomised controlled pilot study. Scandinavian journal of caring sciences. 2010;24(4):755-63.
322	Kristensson J, Hallberg IR, Ekwall AK. Frail older adult's experiences of receiving health care and social services. Journal of gerontological nursing. 2010;36(10):20-1.
323	Kwak J, Ko E, Kramer BJ. Facilitating advance care planning with ethnically diverse groups of frail, low-income elders in the USA. Health and Social Care in the Community. 2014;22(2).
324	Kwok TCY, Bai X, Kao HSR, Li JCY, Ho FKY. Cognitive effects of calligraphy therapy for older people: a randomized controlled trial in Hong Kong. Clinical interventions in aging. 2011;6:269-73.
325	Kyodo R, Kudo T, Horiuchi A, Sakamoto T, Shimizu T. Pureed diets containing a gelling agent to reduce the risk of aspiration in elderly patients with moderate to severe dysphagia: a randomized, crossover trial. Medicine. 2020;99(31):e21165.
326	Lafond N, Maula A, Iliffe S, Vedhara K, Audsley S, Kendrick D, et al. 'We got more than we expected.' Older people's experiences of falls-prevention exercise interventions and implications for practice; a qualitative study. Primary health care research & development. 2019;20:e103.
327	Lammes E, Rydwik E, Akner G. Effects of nutritional intervention and physical training on energy intake, resting metabolic rate and body composition in frail elderly. a randomised, controlled pilot study. The journal of nutrition, health & aging. 2012;16(2):162-7.
328	Lampela P, Lavikainen P, Huupponen R, Leskinen E, Hartikainen S. Comprehensive geriatric assessment decreases prevalence of orthostatic hypotension in older persons. Scandinavian journal of public health. 2013;41(4):351-8.
329	Lampela P, Taipale H, Lavikainen P, Hartikainen S. The effect of comprehensive geriatric assessment on anticholinergic exposure assessed by four ranked anticholinergic lists. Archives of gerontology and geriatrics. 2017;68:195-201.
330	Landefeld CS, Palmer RM, Kresevic DM, Fortinsky RH, Kowal J. A randomized trial of care in a hospital medical unit especially designed to improve the functional outcomes of acutely ill older patients. The New England journal of medicine. 1995;332(20):1338-44.
331	Langer T, Santini A, Zadek F, Chiodi M, Pugni P, Cordolcini V, et al. Intraoperative hypotension is not associated with postoperative cognitive dysfunction in elderly patients undergoing general anesthesia for surgery: results of a randomized controlled pilot trial. Journal of clinical anesthesia. 2019;52:111-8.
332	Langkamp-Henken B, Herrlinger-Garcia KA, Stechmiller JK, Nickerson-Troy JA, Lewis B, Moffatt L. Arginine supplementation is well tolerated but does not enhance mitogen-induced lymphocyte proliferation in elderly nursing home residents with pressure ulcers. Jpen: Journal of Parenteral & Enteral Nutrition. 2000;24(5):280-7.
333	Langkamp-Henken B, Herrlinger-Garcia KA, Stechmiller JK, Nickerson-Troy JA, Lewis B, Moffatt L, et al. Arginine supplementation is well tolerated but does not enhance mitogen-induced lymphocyte proliferation in elderly nursing home residents with pressure ulcers. JPEN Journal of Parenteral & Enteral Nutrition. 2000;24(5):280-7.
334	Larsen RT, Korfitsen CB, Keller C, Christensen J, Andersen HB, Juhl C, et al. The MIPAM trial - motivational interviewing and physical activity monitoring to enhance the daily level of physical activity among older adults - a randomized controlled trial. European Reviews of Aging & Physical Activity. 2021;18(1):12.
335	Latham NK, Anderson CS, Lee A, Bennett DA, Moseley A, Cameron ID. A randomized, controlled trial of quadriceps resistance exercise and vitamin D in frail older people: the Frailty Interventions Trial in Elderly Subjects (FITNESS). Journal of the American Geriatrics Society. 2003;51(3):291-9.
336	Law M, Withers H, Morris J, Anderson F. Vitamin D supplementation and the prevention of fractures and falls: results of a randomised trial in elderly people in residential accommodation. Age and ageing. 2006;35(5):482-6.

337	Lawler K, Shields N, Taylor NF. Training family to assist with physiotherapy for older people transitioning from hospital to the community: a pilot randomized controlled trial. Clinical rehabilitation. 2019;33(10):1625-35.
338	Lee M-C, Wu T-Y, Huang S-J, Chen Y-M, Hsiao S-H, Tsai C-Y. Post-acute care for frail older people decreases 90-day emergency room visits, readmissions and mortality: An interventional study. PloS one. 2023;18(1):e0279654.
339	Leen DC, et al. Home and community-based occupational therapy improves functioning in frail older people: a systematic review. Journal of the American Geriatrics Society. 2017;65(8):1863-9.
340	Legrain S, Tubach F, Bonnet-Zamponi D, Lemaire A, Aquino J-P, Paillaud E, et al. A new multimodal geriatric discharge-planning intervention to prevent emergency visits and rehospitalizations of older adults: The optimization of medication in AGEd Multicenter Randomized Controlled Trial. Journal of the American Geriatrics Society. 2011;59(11):2017-28.
341	Leung AC, Yau DC, Liu CP, Yeoh CS, Chui TY, Chi I, et al. Reducing utilisation of hospital services by case management: a randomised controlled trial. Australian health review. 2004;28(1):79-86.
342	Leung AC-t, Liu C-p, Chow NW-s. Cost-benefit analysis of a case management project for the community-dwelling frail elderly in Hong Kong. Journal of Applied Gerontology. 2004;23(1).
343	Leveille SG, Wagner EH, Davis C, Grothaus L, Wallace J, LoGerfo M, et al. Preventing disability and managing chronic illness in frail older adults: a randomized trial of a community-based partnership with primary care. Journal of the American Geriatrics Society. 1998;46(10):1191-8.
344	Levesque L, Ducharme F, Caron C, Hanson E, Magnusson L, Nolan J, et al. A partnership approach to service needs assessment with family caregivers of an aging relative living at home: a qualitative analysis of the experiences of caregivers and practitioners. International journal of nursing studies. 2010;47(7):876-87.
345	Lewin G, Allan J, Patterson C, Knuiman M, Boldy D, Hendrie D. A comparison of the home-care and healthcare service use and costs of older Australians randomised to receive a restorative or a conventional home-care service. Health & social care in the community. 2014;22(3):328-36.
346	Lewin G, De San Miguel K, Knuiman M, Alan J, Boldy D, Hendrie D, et al. A randomised controlled trial of the Home Independence Program, an Australian restorative home-care programme for older adults. Health & Social Care in the Community. 2013;21(1):69-78.
347	Lewin G, Miguel KDS, Knulman M. A randomised controlled trial of the Home Independence Program (HIP), an Australian restorative home-care programme for older adults. Health and Social Care in the Community. 2013;21(1).
348	Li B, Powell AM, Hooper PL, Sheidow TG. Prospective evaluation of teleophthalmology in screening and recurrence monitoring of neovascular age-related macular degeneration: a randomized clinical trial. JAMA ophthalmology. 2015;133(3):276-82.
349	Li C-M, Chen C-Y, Li C-Y, Wang W-D, Wu S-C. The effectiveness of a comprehensive geriatric assessment intervention program for frailty in community-dwelling older people: a randomized, controlled trial. Archives of gerontology and geriatrics. 2010;50 Suppl 1:S39-42.
350	Li MK, Lee TF, Suen KP. Complementary effects of auricular acupressure in relieving constipation symptoms and promoting disease-specific health-related quality of life: a randomized placebo-controlled trial. Complementary therapies in medicine. 2014;22(2):266-77.
351	Li Y, Gao Y, Hu S, Chen H, Zhang M, Yang Y, et al. Effects of multicomponent exercise on the muscle strength, muscle endurance and balance of frail older adults: A meta-analysis of randomised controlled trials. Journal of clinical nursing. 2023;32(9-10):1795-805.
352	Liao C-D, Lee P-H, Hsiao D-J, Huang S-W, Tsauo J-Y, Chen H-C, et al. Effects of Protein Supplementation Combined with Exercise Intervention on Frailty Indices, Body Composition, and Physical Function in Frail Older Adults. Nutrients.

	2018;10(12).
353	Liao Y-Y, Chen IH, Hsu W-C, Tseng H-Y, Wang R-Y. Effect of exergaming versus combined exercise on cognitive function and brain activation in frail older adults: A randomised controlled trial. Annals of physical and rehabilitation medicine. 2021;64(5):101492.
354	Lightbody E, Watkins C, Leathley M, Sharma A, Lye M. Evaluation of a nurse-led falls prevention programme versus usual care: a randomized controlled trial. Age and ageing. 2002;31(3):203-10.
355	Lihavainen K, Sipila S, Rantanen T, Kauppinen M, Sulkava R, Hartikainen S. Effects of comprehensive geriatric assessment and targeted intervention on mobility in persons aged 75 years and over: a randomized controlled trial. Clinical rehabilitation. 2012;26(4):314-26.
356	Lihavainen K, Sipila S, Rantanen T, Seppanen J, Lavikainen P, Sulkava R, et al. Effects of comprehensive geriatric intervention on physical performance among people aged 75 years and over. Aging clinical and experimental research. 2012;24(4):331-8.
357	Liimatta H, Lampela P, Laitinen-Parkkonen P, Pitkala KH. Effects of preventive home visits on health-related quality-of-life and mortality in home-dwelling older adults. Scandinavian journal of primary health care. 2019;37(1):90-7.
358	Liimatta HA, Lampela P, Kautiainen H, Laitinen-Parkkonen P, Pitkala KH. The Effects of Preventive Home Visits on Older People's Use of Health Care and Social Services and Related Costs. Journals of Gerontology: Series A: Biological Sciences and Medical Sciences. 2020;75(8):1586-93.
359	Lim YK, Jackson C, Dauway EL, Richter KK. Risk Factors for Adverse Outcome for Elderly Patients undergoing Curative Oncological Resection for Gastrointestinal Malignancies. Visceral Medicine. 2017;33(4):254-61.
360	Lin FV, Cottone K, McDermott K, Jacobs A, Nelson D, Porsteinsson A, et al. Attitudes Toward Computers Moderate the Effect of Computerized Cognitive Trainings in Oldest-Old Senior Living Center Residents. American Journal of Geriatric Psychiatry. 2021;29(3):285-94.
361	Lin M-R, Wolf SL, Hwang H-F, Gong S-Y, Chen C-Y. A randomized, controlled trial of fall prevention programs and quality of life in older fallers. Journal of the American Geriatrics Society. 2007;55(4):499-506.
362	Liu C-J, Chang W-P, Chang MC. Occupational Therapy Interventions to Improve Activities of Daily Living for Community-Dwelling Older Adults: A Systematic Review. The American journal of occupational therapy : official publication of the American Occupational Therapy Association. 2018;72(4):7204190060p1-p11.
363	Liu JYW, Kwan RYC, Yin Y-H, Lee PH, Siu JY-M, Bai X. Enhancing the Physical Activity Levels of Frail Older Adults with a Wearable Activity Tracker-Based Exercise Intervention: A Pilot Cluster Randomized Controlled Trial. International journal of environmental research and public health. 2021;18(19).
364	Liu JYW, Lai CKY. Implementation of Observational Pain Management Protocol for Residents With Dementia: a Cluster-RCT. Journal of the American Geriatrics Society. 2017;65(3):e56-e63.
365	Liu X, Song L, Xiao S, Wang Y. Comprehensive Geriatric Assessment, Multidisciplinary Treatment, and Nurse-Guided Transitional Care in Hospitalized Older Adults: A Randomized Controlled Trial. Research in gerontological nursing. 2023;16(5):224-30.
366	Liu Z, Hao H, Yin C, Chu Y, Li J, Xu D. Therapeutic effects of atorvastatin and ezetimibe compared with double-dose atorvastatin in very elderly patients with acute coronary syndrome. Oncotarget. 2017;8(25):41582-9.
367	Livingston G, Barber J, Marston L, Stringer A, Panca M, Hunter R, et al. Clinical and cost-effectiveness of the Managing Agitation and Raising Quality of Life (MARQUE) intervention for agitation in people with dementia in care homes: a single-blind, cluster-randomised controlled trial. The lancet Psychiatry. 2019;6(4):293-304.

368	Looman WM, Huijsman R, Fabbricotti IN. The (cost-)effectiveness of preventive, integrated care for community-dwelling frail older people: a systematic review. Health and Social Care in the Community. 2019;27(1):1-30.
369	Looman WM, Huijsman R, Fabbricotti IN. The (cost-)effectiveness of preventive, integrated care for community-dwelling frail older people. Health and Social Care in the Community. 2019;27(1).
370	Lopez P, Izquierdo M, Radaelli R, Sbruzzi G, Grazioli R, Pinto RS, et al. Effectiveness of Multimodal Training on Functional Capacity in Frail Older People: A Meta-Analysis of Randomized Controlled Trials. Journal of aging and physical activity. 2018;26(3):407-18.
371	Lopez-Hartmann M, Wens J, Verhoeven V. The effect of caregiver support interventions for informal caregivers of community-dwelling frail elderly: a systematic review. International Journal of Integrated Care. 2012;12.
372	Louzada LL, Machado FV, Quintas JL, Ribeiro GA, Silva MV, Mendonca-Silva DL, et al. The efficacy and safety of zolpidem and zopiclone to treat insomnia in Alzheimer's disease: A randomized, triple-blind, placebo-controlled trial. Neuropsychopharmacology. 2022;47(2):570-9.
373	Low LF, Goodenough B, Fletcher J, Xu K, Casey AN, Chenoweth L, et al. The Effects of Humor Therapy on Nursing Home Residents Measured Using Observational Methods: the SMILE Cluster Randomized Trial. Journal of the American Medical Directors Association. 2014;15(8):564-9.
374	Luger E, Dorner TE, Haider S, Kapan A, Lackinger C, Schindler K. Effects of a Home-Based and Volunteer-Administered Physical Training, Nutritional, and Social Support Program on Malnutrition and Frailty in Older Persons: A Randomized Controlled Trial. Journal of the American Medical Directors Association. 2016;17(7):671.e9-.e16.
375	Lundqvist M, Alwin J, Henriksson M, Husberg M, Carlsson P, Ekdahl AW. Cost-effectiveness of comprehensive geriatric assessment at an ambulatory geriatric unit based on the AGe-FIT trial. BMC geriatrics. 2018;18(1):32.
376	Luttenberger K, Donath C, Uter W, Graessel E. Effects of multimodal nondrug therapy on dementia symptoms and need for care in nursing home residents with degenerative dementia: a randomized-controlled study with 6-month follow-up. Journal of the American Geriatrics Society. 2012;60(5):830-40.
377	Lyndon H, Latour JM, Marsden J, Kent B. A nurse-led comprehensive geriatric assessment intervention in primary care: A feasibility cluster randomized controlled trial. Journal of advanced nursing. 2023;79(9):3473-86.
378	MacDonald AA, Joyson A, Lee R, Seymour DG, Soiza RL. The effect of hearing augmentation on cognitive assessment scales at admission to hospital. American journal of geriatric psychiatry. 2012;20(4):355-61.
379	Maki N, Sakamoto H, Takata Y, Kobayashi N, Kikuchi S, Goto Y, et al. Effect of respiratory rehabilitation for frail older patients with musculoskeletal disorders: a randomized controlled trial. Journal of rehabilitation medicine. 2018;50(10):908-13.
380	Manietta C, Labonté V, Thiesemann R, Sirsch EG, Möhler R. Algorithm-based pain management for people with dementia in nursing homes. Cochrane Database of Systematic Reviews. 2022(4).
381	Maniewicz S, Duvernay E, Srinivasan M, Perneger T, Schimmel M, Muller F. Effect of implant-supported mandibular overdentures versus reline on masticatory performance and salivary flow rates in very old adults-A randomized clinical trial. Clinical Oral Implants Research. 2019;30(1):59-67.
382	Manor B, Lough M, Gagnon MM, Cupples A, Wayne PM, Lipsitz LA. Functional benefits of tai chi training in senior housing facilities. Journal of the American Geriatrics Society. 2014;62(8):1484-9.
383	Marek KD, Stetzer F, Ryan PA, Bub LD, Adams SJ, Schlidt A, et al. Nurse care coordination and technology effects on health status of frail older adults via enhanced self-management of medication: randomized clinical trial to test efficacy. Nursing research. 2013;62(4):269-78.

384	Martin JL, Marler MR, Harker JO, Josephson KR, Alessi CA. A multicomponent nonpharmacological intervention improves activity rhythms among nursing home residents with disrupted sleep/wake patterns. Journals of gerontology Series A, Biological sciences and medical sciences. 2007;62(1):67-72.
385	Martin RS, Hayes BJ, Hutchinson A, Tacey M, Yates P, Lim WK. Introducing Goals of Patient Care in Residential Aged Care Facilities to Decrease Hospitalization: a Cluster Randomized Controlled Trial. Journal of the American Medical Directors Association. 2019;20(10):1318-24.e2.
386	Martinez-Velilla N, Abizanda P, Gomez-Pavon J, Zambom-Ferraresi F, Saez de Asteasu ML, Fiatarone Singh M, et al. Effect of an Exercise Intervention on Functional Decline in Very Old Patients During Acute Hospitalizations: Results of a Multicenter, Randomized Clinical Trial. JAMA Internal Medicine. 2022;182(3):345-7.
387	Martinez-Velilla N, Valenzuela PL, de Asteasu MLS, Zambom-Ferraresi F, Ramirez-Velez R, Garcia-Hermoso A, et al. Effects of a Tailored Exercise Intervention in Acutely Hospitalized Oldest Old Diabetic Adults: an Ancillary Analysis. Journal of clinical endocrinology and metabolism. 2021;106(2):E899-E906.
388	Maruyama M, Abe R, Shimono T, Iwabuchi N, Abe F, Xiao J-Z. The effects of non-viable Lactobacillus on immune function in the elderly: a randomised, double-blind, placebo-controlled study. International Journal of Food Sciences & Nutrition. 2016;67(1):67-73.
389	Mathews M. The Experience of Counseling Among a Singaporean Elderly Population: A Qualitative Account of What Clients Report as Beneficial. Journal of Cross-Cultural Gerontology. 2016;31(3):277-91.
390	Mazya AL, Garvin P, Ekdahl AW. Outpatient comprehensive geriatric assessment: effects on frailty and mortality in old people with multimorbidity and high health care utilization. Aging clinical and experimental research. 2019;31(4):519-25.
391	McCreedy EM, Sisti A, Gutman R, Dionne L, Rudolph JL, Baier R, et al. Pragmatic Trial of Personalized Music for Agitation and Antipsychotic Use in Nursing Home Residents With Dementia. Journal of the American Medical Directors Association. 2022;23(7):1171-7.
392	McMurdo ME, Price RJ, Shields M, Potter J, Stott DJ. Should oral nutritional supplementation be given to undernourished older people upon hospital discharge? A controlled trial. Journal of the American Geriatrics Society. 2009;57(12):2239-45.
393	McMurdo ME, Rennie L. A controlled trial of exercise by residents of old people's homes. Age & Ageing. 1993;22(1):11-5.
394	Melis RJF, Adang E, Teerenstra S, van Eijken MIJ, Wimo A, van Achterberg T, et al. Cost-effectiveness of a multidisciplinary intervention model for community-dwelling frail older people. The journals of gerontology Series A, Biological sciences and medical sciences. 2008;63(3):275-82.
395	Melis RJF, van Eijken MIJ, Teerenstra S, van Achterberg T, Parker SG, Borm GF, et al. A randomized study of a multidisciplinary program to intervene on geriatric syndromes in vulnerable older people who live at home (Dutch EASYcare Study). The journals of gerontology Series A, Biological sciences and medical sciences. 2008;63(3):283-90.
396	Melis RJF, van Eijken MIJ, van Achterberg T, Teerenstra S, Vernooij-Dassen MJFJ, van de Lisdonk EH, et al. The effect on caregiver burden of a problem-based home visiting programme for frail older people. Age and ageing. 2009;38(5):542-7.
397	Meng R, Ding Y, Asmaro K, Brogan D, Meng L, Sui M, et al. Ischemic Conditioning Is Safe and Effective for Octo- and Nonagenarians in Stroke Prevention and Treatment. Neurotherapeutics. 2015;12(3):667-77.
398	Merz MA, Terheyden H, Huber CG, Seixas AA, Schoetzau A, Schneeberger AR. Facilitators and barriers influencing the readiness to receive dental implants in a geriatric institutionalised population-A randomized non-invasive interventional study. Gerodontology. 2017;34(3):306-12.

399	Metzelthin S, van Rossum E, de Whitte L. Effectiveness of interdisciplinary primary care approach to reduce disability in community dwelling frail older people: cluster randomised controlled trial. BMJ. 2013;347(7962):12.
400	Mikolaizak AS, Lord SR, Tiedemann A, Simpson P, Caplan GA, Bendall J, et al. A multidisciplinary intervention to prevent subsequent falls and health service use following fall-related paramedic care: a randomised controlled trial. Age and ageing. 2017;46(2):200-7.
401	Miller LJ, Chacko R. The role of cholesterol and statins in Alzheimer's disease. Annals of Pharmacotherapy. 2004;38(1):91-8.
402	Miller MD, Crotty M, Whitehead C, Bannerman E, Daniels LA. Nutritional supplementation and resistance training in nutritionally at risk older adults following lower limb fracture: a randomized controlled trial. Clinical rehabilitation. 2006;20(4):311-23.
403	Milte CM, Luszcz MA, Ratcliffe J, Masters S, Crotty M. Influence of health locus of control on recovery of function in recently hospitalized frail older adults. Geriatrics & gerontology international. 2015;15(3):341-9.
404	Milte R, Miller MD, Crotty M, Mackintosh S, Thomas S, Cameron ID, et al. Cost-effectiveness of individualized nutrition and exercise therapy for rehabilitation following hip fracture. Journal of rehabilitation medicine. 2016;48(4):378-85.
405	Mitchell SL, D'Agata EMC, Hanson LC, Loizeau AJ, Habtemariam DA, Tsai T, et al. The Trial to Reduce Antimicrobial Use in Nursing Home Residents With Alzheimer Disease and Other Dementias (TRAIN-AD): a Cluster Randomized Clinical Trial. JAMA internal medicine. 2021;181(9):1174-82.
406	Moller UO, Kristensson J, Midlov P, Ekdahl C, Jakobsson U. Effects of a one-year home-based case management intervention on falls in older people: a randomized controlled trial. Journal of aging and physical activity. 2014;22(4):457-64.
407	Mollinedo Cardalda I, Lopez A, Cancela Carral JM. The effects of different types of physical exercise on physical and cognitive function in frail institutionalized older adults with mild to moderate cognitive impairment. A randomized controlled trial. Archives of gerontology and geriatrics. 2019;83:223-30.
408	Monette J, Miller MA, Monette M, Laurier C, Boivin JF, Sourial N, et al. Effect of an educational intervention on optimizing antibiotic prescribing in long-term care facilities. Journal of the American Geriatrics Society. 2007;55(8):1231-5.
409	Monteserin R. Effectiveness of a geriatric intervention in primary care: a randomized clinical trial. Family Practice. 2010;27(3).
410	Moppett IK, Rowlands M, Mannings AM, Marufu TC, Sahota O, Yeung J. The effect of intravenous iron on erythropoiesis in older people with hip fracture. Age and ageing. 2019;48(5):751-5.
411	Moreira NB, Goncalves G, da Silva T, Zanardini FEH, Bento PCB. Multisensory exercise programme improves cognition and functionality in institutionalized older adults: A randomized control trial. Physiotherapy research international : the journal for researchers and clinicians in physical therapy. 2018;23(2):e1708.
412	Morioka S, Fujita H, Hiyamizu M, Maeoka H, Matsuo A. Effects of plantar perception training on standing posture balance in the old old and the very old living in nursing facilities: a randomized controlled trial. Clinical Rehabilitation. 2011;25(11):1011-20.
413	Mortsiefer A, Loscher S, Pashutina Y, Santos S, Altiner A, Drewelow E, et al. Family Conferences to Facilitate Deprescribing in Older Outpatients With Frailty and With Polypharmacy: The COFRAIL Cluster Randomized Trial. JAMA network open. 2023;6(3):e234723.
414	Movrin I. Cemented versus uncemented hemiarthroplasty for displaced femoral neck fractures: a randomized controlled trial with two years follow-up. Acta orthopaedica et traumatologica turcica. 2020;54(1):83-8.
415	Mueller Y, Schwarz J, Monod S, Locatelli I, Senn N. Use of standardized brief geriatric evaluation compared with routine care in general practice for preventing functional decline: a pragmatic cluster-randomized trial. CMAJ : Canadian Medical Association journal = journal de l'Association medicale canadienne. 2021;193(33):E1289-E99.

416	Mukka S, Sjoholm P, Chammout G, Kelly-Pettersson P, Sayed-Noor AS, Skoldenberg O. External Validity of the HOPE-Trial: Hemiarthroplasty Compared with Total Hip Arthroplasty for Displaced Femoral Neck Fractures in Octogenarians. JB & JS Open Access. 2019;4(2):e0061.
417	Muller F, Duvernay E, Loup A, Vazquez L, Herrmann FR, Schimmel M. Implant-supported mandibular overdentures in very old adults: a randomized controlled trial. Journal of Dental Research. 2013;92(12 Suppl):154S-60S.
418	Muneretto C, Bisleri G, Negri A, Manfredi J, Metra M, Nodari S, et al. Total arterial myocardial revascularization with composite grafts improves results of coronary surgery in elderly: A prospective randomized comparison with conventional coronary artery bypass surgery. Circulation. 2003;108(10):29-33.
419	Muneretto C, Negri A, Bisleri G, Manfredi J, Terrini A, Metra M, et al. Is total arterial myocardial revascularization with composite grafts a safe and useful procedure in the elderly? European Journal of Cardio-Thoracic Surgery. 2003;23(5):657-64.
420	Mustacchi G, Ceccherini R, Milani S, Pluchinotta A, De Matteis A, Maiorino L, et al. Tamoxifen alone versus adjuvant tamoxifen or operable breast cancer of the elderly: long-term results of the phase III randomized controlled multicenter GRETA trial. Annals of Oncology. 2003;14(3):414-20.
421	Nace DA, Lin CJ, Ross TM, Saracco S, Churilla RM, Zimmerman RK. Randomized, controlled trial of high-dose influenza vaccine among frail residents of long-term care facilities. Journal of infectious diseases. 2015;211(12):1915-24.
422	Nagata CdA, Garcia PA, Hamu TCDdS, Caetano MBD, Costa RR, Leal JC, et al. Are dose-response relationships of resistance training reliable to improve functional performance in frail and pre-frail older adults? A systematic review with meta-analysis and meta-regression of randomized controlled trials. Ageing research reviews. 2023;91:102079.
423	Nagayama H, Tomori K, Ohno K, Takahashi K, Ogahara K, Sawada T, et al. Effectiveness and Cost-Effectiveness of Occupation-Based Occupational Therapy Using the Aid for Decision Making in Occupation Choice (ADOC) for Older Residents: pilot Cluster Randomized Controlled Trial. PloS one. 2016;11(3):e0150374.
424	Nakagami G, Sanada H, Konya C, Kitagawa A, Tadaka E, Matsuyama Y. Evaluation of a new pressure ulcer preventive dressing containing ceramide 2 with low frictional outer layer. Journal of advanced nursing. 2007;59(5):520-9.
425	Namba K, Hatano M, Yaeshima T, Takase M, Suzuki K. Effects of Bifidobacterium longum BB536 administration on influenza infection, influenza vaccine antibody titer, and cell-mediated immunity in the elderly. Bioscience, biotechnology, and biochemistry. 2010;74(5):939-45.
426	Narme P, Clément S, Ehrlé N, Schiaratura L, Vachez S, Courtaigne B, et al. Efficacy of musical interventions in dementia: evidence from a randomized controlled trial. Journal of Alzheimer's disease. 2014;38(2):359-69.
427	Naughton BJ, Moran MB, Feinglass J, Falconer J, Williams ME. Reducing hospital costs for the geriatric patient admitted from the emergency department: a randomized trial. Journal of the American Geriatrics Society. 1994;42(10):1045-9.
428	Nava S, Grassi M, Fanfulla F, Domenighetti G, Carlucci A, Perren A, et al. Non-invasive ventilation in elderly patients with acute hypercapnic respiratory failure: a randomised controlled trial. Age & Ageing. 2011;40(4):444-50.
429	Nieves JW, Cosman F, McMahon D, Redko M, Hentschel I, Bartolotta R, et al. Teriparatide and pelvic fracture healing: a phase 2 randomized controlled trial. Osteoporosis international. 2022;33(1):239-50.
430	Niki K, Yahara M, Inagaki M, Takahashi N, Watanabe A, Okuda T, et al. Immersive Virtual Reality Reminiscence Reduces Anxiety in the Oldest-Old Without Causing Serious Side Effects: A Single-Center, Pilot, and Randomized Crossover Study. Frontiers in Human Neuroscience. 2020;14:598161.
431	Nikolaus T, Bach M. Preventing falls in community-dwelling frail older people using a home intervention team (HIT): results from the randomized Falls-HIT trial. Journal of the American Geriatrics Society. 2003;51(3):300-5.

432	Nikolaus T, Specht-Leible N, Bach M. A randomized trial of comprehensive geriatric assessment and home intervention in the care of hospitalized patients. Age and Ageing. 1999;28(6).
433	Nilsen P, Wallerstedt B, Behm L, Ahlström G. Towards evidence-based palliative care in nursing homes in Sweden: a qualitative study informed by the organizational readiness to change theory. Implementation Science. 2018;13(1):1-N.PAG.
434	Nord M, Lyth J, Alwin J, Marcusson J. Costs and effects of comprehensive geriatric assessment in primary care for older adults with high risk for hospitalisation. BMC geriatrics. 2021;21(1):263.
435	Nord M, Lyth J, Marcusson J, Alwin J. Cost-Effectiveness of Comprehensive Geriatric Assessment Adapted to Primary Care. Journal of the American Medical Directors Association. 2022;23(12):2003-9.
436	Nourhashemi F, Andrieu S, Gillette-Guyonnet S, Giraudeau B, Cantet C, Coley N, et al. Effectiveness of a specific care plan in patients with Alzheimer's disease: cluster randomised trial (PLASA study). BMJ (Clinical research ed). 2010;340:c2466.
437	Nygårdh A, Ahlström G, Wann-Hansson C. Handling a challenging context: experiences of facilitating evidence-based elderly care. Journal of Nursing Management. 2016;24(3):201-10.
438	Nykanen I, Torronen R, Schwab U. Dairy-Based and Energy-Enriched Berry-Based Snacks Improve or Maintain Nutritional and Functional Status in Older People in Home Care. Journal of nutrition, health & aging. 2018;22(10):1205-10.
439	O'Halloran PD, Shields N, Blackstock F, Wintle E, Taylor NF. Motivational interviewing increases physical activity and self-efficacy in people living in the community after hip fracture: a randomized controlled trial. Clinical rehabilitation. 2016;30(11):1108-19.
440	O'Hare L, Savage E, McCullagh R, Bantry White E, Fitzgerald E, Timmons S. Frail older adults' perceptions of an in-hospital structured exercise intervention. Physiotherapy. 2017;103(4):478-84.
441	O'Reilly J, Lowson K, Green J, Young JB, Forster A. Post-acute care for older people in community hospitals--a cost-effectiveness analysis within a multi-centre randomised controlled trial. Age and ageing. 2008;37(5):513-20.
442	Ogasawara T, Umezawa H, Naito Y, Takeuchi T, Kato S, Yano T, et al. Procalcitonin-guided antibiotic therapy in aspiration pneumonia and an assessment of the continuation of oral intake. Respiratory investigation. 2014;52(2):107-13.
443	Oh KM, Warnes AM, Bath P. Effectiveness of a rapid response service for frail older people. Nursing older people. 2009;21(5):25-32.
444	Ohura T, Nakajo T, Okada S, Omura K, Adachi K, Oishi S. Effects of nutrition intervention for pressure ulcer patients--healing rate and speed of wound size and nutrition. Nihon Ronen Igakkai zasshi [Japanese journal of geriatrics]. 2013;50(3):377-83.
445	Okoye C, Calsolaro V, Niccolai F, Calabrese AM, Franchi R, Rogani S, et al. A Randomized, Open-Label Study to Assess Efficacy of Weekly Assumption of Cholecalciferol versus Calcifediol in Older Patients with Hypovitaminosis D. Geriatrics (Basel, Switzerland). 2022;7(1).
446	Olazarán J, González B, López-Álvarez J, Castagna A, Osa-Ruiz E, Herrero-Cano V, et al. Motor effects of REAC in advanced Alzheimer's disease: results from a pilot trial. Journal of Alzheimer's disease. 2013;36(2):297-302.
447	Olazarán J, González B, Osa-Ruiz E, Felipe-Ruiz S, Boyano I, Fontani V, et al. Motor effects of radio electric asymmetric conveyer in Alzheimer's disease: results from a cross-over trial. Journal of Alzheimer's disease. 2014;42(1):325-32.
448	Oliveira J, Mesquita-Bastos J, de Melo CA, Ribeiro F. Postaerobic Exercise Blood Pressure Reduction in Very Old Persons With Hypertension. Journal of Geriatric Physical Therapy. 2016;39(1):8-13.
449	Ollonqvist K. Alleviating loneliness among frail older people: Findings from a randomised controlled trial. International Journal of Mental Health Promotion. 2008;10(2).

450	Ollonqvist K. Network-based rehabilitation increases formal support of frail elderly home-dwelling persons in Finland: randomised controlled trial. Health and Social Care in the Community. 2008;16(2):115-25.
451	Oostra DL, Fierkens C, Alewijnse MEJ, Olde Rikkert MGM, Nieuwboer MS, Perry M. Implementation of interprofessional digital communication tools in primary care for frail older adults: An interview study. Journal of interprofessional care. 2023;37(3):362-70.
452	Opinder S, et al. The Community In-reach Rehabilitation and Care Transition (CIRACT) clinical and cost-effectiveness randomisation controlled trial in older people admitted to hospital as an acute medical emergency. Age and Ageing. 2017;46(1):26-32.
453	Optale G, Urgesi C, Busato V, Marin S, Piron L, Priftis K, et al. Controlling memory impairment in elderly adults using virtual reality memory training: a randomized controlled pilot study. Neurorehabilitation and neural repair. 2010;24(4):348-57.
454	Orrell M, Aguirre E, Spector A, Hoare Z, Woods RT, Streater A, et al. Maintenance cognitive stimulation therapy for dementia: single-blind, multicentre, pragmatic randomised controlled trial. British journal of psychiatry. 2014;204(6):454-61.
455	Ortiz-Alonso J, Bustamante-Ara N, Valenzuela PL, Vidán-Astiz M, Rodríguez-Romo G, Mayordomo-Cava J, et al. Effect of a Simple Exercise Program on Hospitalization-Associated Disability in Older Patients: a Randomized Controlled Trial. Journal of the American Medical Directors Association. 2020;21(4):531-7.e1.
456	Ortiz-Alonso J, Bustamante-Ara N, Valenzuela PL, Vidán-Astiz M, Rodríguez-Romo G, Mayordomo-Cava J, et al. Effect of a Simple Exercise Program on Hospitalization-Associated Disability in Older Patients: A Randomized Controlled Trial. Journal of the American Medical Directors Association. 2020;21(4):531-.
457	Overbeek A, Korfage IJ, Jabbarian LJ, Billekens P, Hammes BJ, Polinder S, et al. Advance Care Planning in Frail Older Adults: A Cluster Randomized Controlled Trial. Journal of the American Geriatrics Society. 2018;66(6):1089-95.
458	Overgaard C, Bøggild H, Hede B, Bagger M, Hartmann LG, Aagaard K. Improving oral health in nursing home residents: a cluster randomized trial of a shared oral care intervention. Community dentistry and oral epidemiology. 2022;50(2):115-23.
459	Overshott R, Vernon M, Morris J, Burns A. Rivastigmine in the treatment of delirium in older people: a pilot study. International psychogeriatrics. 2010;22(5):812-8.
460	Park J, Tolea MI, Sherman D, Rosenfeld A, Arcay V, Lopes Y, et al. Feasibility of Conducting Nonpharmacological Interventions to Manage Dementia Symptoms in Community-Dwelling Older Adults: a Cluster Randomized Controlled Trial. American journal of Alzheimer's disease and other dementias. 2020;35:1533317519872635.
461	Park W, Lee J, Hong K, Park H-Y, Park S, Kim N, et al. Protein-Added Healthy Lunch-Boxes Combined with Exercise for Improving Physical Fitness and Vascular Function in Pre-Frail Older Women: A Community-Based Randomized Controlled Trial. Clinical interventions in aging. 2023;18:13-27.
462	Parraga-Montilla JA, Aibar-Almazan A, Cabrera-Linares JC, Lozano-Aguilera E, Serrano Huete V, Escarabajal Arrieta MD, et al. A Randomized Controlled Trial Protocol to Test the Efficacy of a Dual-Task Multicomponent Exercise Program vs. a Simple Program on Cognitive and Fitness Performance in Elderly People. International journal of environmental research and public health. 2021;18(12).
463	Parsons J, Mathieson S, Jull A, Parsons M. Does vibration training reduce the fall risk profile of frail older people admitted to a rehabilitation facility? A randomised controlled trial. Disability and rehabilitation. 2016;38(11):1082-8.
464	Parsons M. The Assessment of Services Promoting Independence and Recovery in Elders Trial (ASPIRE) : a pre-planned meta-analysis of three independent randomised controlled trial evaluations of ageing in place initiatives in New Zealand. Age and Ageing. 2012.
465	Parsons M, Senior H, Kerse N, Chen M-H, Jacobs S, Anderson C. Randomised trial of restorative home care for frail older people in New Zealand. Nursing older people. 2017;29(7):27-33.

466	Parsons M, Senior H, Kerse N, Chen M-H, Jacobs S, Vanderhoorn S, et al. Should care managers for older adults be located in primary care? A randomized controlled trial. Journal of the American Geriatrics Society. 2012;60(1):86-92.
467	Parsons M, Senior H, Mei-Hu Chen X, Jacobs S, Parsons J, Sheridan N, et al. Assessment without action; a randomised evaluation of the interRAI home care compared to a national assessment tool on identification of needs and service provision for older people in New Zealand. Health & social care in the community. 2013;21(5):536-44.
468	Partridge JSL, Harari D, Martin FC, Dhesi JK. The impact of pre-operative comprehensive geriatric assessment on postoperative outcomes in older patients undergoing scheduled surgery: a systematic review. Anaesthesia. 2014;69 Suppl 1:8-16.
469	Patterson SM, Hughes CM, Cardwell C, Lapane KL, Murray AM, Crealey GE. A cluster randomized controlled trial of an adapted U.S. model of pharmaceutical care for nursing home residents in Northern Ireland (Fleetwood Northern Ireland study): a cost-effectiveness analysis. Journal of the American Geriatrics Society. 2011;59(4):586-93.
470	Patterson SM, Hughes CM, Crealey G, Cardwell C, Lapane KL. An evaluation of an adapted U.S. model of pharmaceutical care to improve psychoactive prescribing for nursing home residents in northern ireland (fleetwood northern ireland study). Journal of the American Geriatrics Society. 2010;58(1):44-53.
471	Paulden M, Bergstrom N, Horn SD, Rapp M, Stern A, Barrett R, et al. Turning for Ulcer Reduction (TURN) Study: an Economic Analysis. Ontario health technology assessment series. 2014;14(12):1-24.
472	Pauser J, Nordmeyer M, Biber R, Jantsch J, Kopschina C, Bail HJ, et al. Incisional negative pressure wound therapy after hemiarthroplasty for femoral neck fractures - reduction of wound complications. International wound journal. 2016;13(5):663-7.
473	Pedersen JL, Pedersen PU, Damsgaard EM. Early Nutritional Follow-Up after Discharge Prevents Deterioration of ADL Functions in Malnourished, Independent, Geriatric Patients Who Live Alone - A Randomized Clinical Trial. Journal of nutrition, health & aging. 2016;20(8):845-53.
474	Pedone C, Rossi FF, Cecere A, Costanzo L, Antonelli Incalzi R. Efficacy of a Physician-Led Multiparametric Telemonitoring System in Very Old Adults with Heart Failure. Journal of the American Geriatrics Society. 2015;63(6):1175-80.
475	Pedziwiatr M, Malczak P, Mizera M, Witowski J, Torbicz G, Major P, et al. Pancreatoduodenectomy for pancreatic head tumors in the elderly - Systematic review and meta-analysis. Surgical Oncology-Oxford. 2018;27(3):346-64.
476	Pellfolk TJ, Gustafson Y, Bucht G, Karlsson S. Effects of a restraint minimization program on staff knowledge, attitudes, and practice: a cluster randomized trial. Journal of the American Geriatrics Society. 2010;58(1):62-9.
477	Perez-Gomez J, Redondo PC, Navarrete-Villanueva D, Lozano-Berges G, Ara I, Gonzalez-Gross M, et al. New Evidence on Regucalcin, Body Composition, and Walking Ability Adaptations to Multicomponent Exercise Training in Functionally Limited and Frail Older Adults. International journal of environmental research and public health. 2021;19(1).
478	Peri K. Does functionality based activity make a difference to health status and mobility?: a randomised controlled trial in residential care facilities (The promoting independent living study; PILS). Age and Ageing. 2008;37(1).
479	Peri K, Kerse N, Robinson E, Parsons M, Parsons J, Latham N. Does functionally based activity make a difference to health status and mobility? A randomised controlled trial in residential care facilities (The Promoting Independent Living Study; PILS). Age and ageing. 2008;37(1):57-63.
480	Perry M, Melis RJF, Teerenstra S, Draskovic I, van Achterberg T, van Eijken MIJ, et al. An in-home geriatric programme for vulnerable community-dwelling older people improves the detection of dementia in primary care. International journal of geriatric psychiatry. 2008;23(12):1312-9.

481	Pershad A, Fraij G, Massaro JM, David SW, Kleiman NS, Denktas AE, et al. Comparison of the use of hemodynamic support in patients >80 years versus patients <80 years during high-risk percutaneous coronary interventions (from the multicenter protect II randomized study). American journal of cardiology. 2014;114(5):657-64.
482	Persson J, Johansson I, Torge CJ, Bergstrom E-K, Hagglin C, Wardh I. Oral Care Cards as a Support in Daily Oral Care of Frail Older Adults: Experiences and Perceptions of Professionals in Nursing and Dental Care-A Qualitative Study. International journal of environmental research and public health. 2022;19(15).
483	Persson M, Hytter-Landahl A, Brismar K, Cederholm T. Nutritional supplementation and dietary advice in geriatric patients at risk of malnutrition. Clinical nutrition (Edinburgh, Scotland). 2007;26(2):216-24.
484	Peskind ER, Tsuang DW, Bonner LT, Pascualy M, Riekse RG, Snowden MB, et al. Propranolol for disruptive behaviors in nursing home residents with probable or possible Alzheimer disease: a placebo-controlled study. Alzheimer Disease & Associated Disorders. 2005;19(1):23-8.
485	Peters R, Pinto E, Beckett N, Swift C, Potter J, McCormack T, et al. Association of depression with subsequent mortality, cardiovascular morbidity and incident dementia in people aged 80 and over and suffering from hypertension. Data from the Hypertension in the Very Elderly Trial (HYVET). Age and ageing. 2010;39(4):439-45.
486	Petersen S, Houston S, Qin H, Tague C, Studley J. The Utilization of Robotic Pets in Dementia Care. Journal of Alzheimer's disease. 2017;55(2):569-74.
487	Pfeiffer K, Kampe K, Klenk J, Rapp K, Kohler M, Albrecht D, et al. Effects of an intervention to reduce fear of falling and increase physical activity during hip and pelvic fracture rehabilitation. Age and ageing. 2020;49(5):771-8.
488	Pieper MJ, Francke AL, van der Steen JT, Scherder EJ, Twisk JW, Kovach CR, et al. Effects of a Stepwise Multidisciplinary Intervention for Challenging Behavior in Advanced Dementia: a Cluster Randomized Controlled Trial. Journal of the American Geriatrics Society. 2016;64(2):261-9.
489	Pitkälä KH, Juola AL, Kautiainen H, Soini H, Finne-Soveri UH, Bell JS, et al. Education to reduce potentially harmful medication use among residents of assisted living facilities: a randomized controlled trial. Journal of the American Medical Directors Association. 2014;15(12):892-8.
490	Pitkalia KH, Laurila JV, Strandberg TE, Tilvis RS. Multicomponent geriatric intervention for elderly inpatients with delirium: a randomized, controlled trial. Journals of Gerontology: Series A: Biological Sciences and Medical Sciences. 2006;61A(2):176-81.
491	Pol MC, Ter Riet G, van Hartingsveldt M, Kröse B, Buurman BM. Effectiveness of sensor monitoring in a rehabilitation programme for older patients after hip fracture: a three-arm stepped wedge randomised trial. Age and ageing. 2019;48(5):650-7.
492	Pollock BG, Mulsant BH, Rosen J, Mazumdar S, Blakesley RE, Houck PR, et al. A double-blind comparison of citalopram and risperidone for the treatment of behavioral and psychotic symptoms associated with dementia. American journal of geriatric psychiatry. 2007;15(11):942-52.
493	Pollock RD, Martin FC, Newham DJ. Whole-body vibration in addition to strength and balance exercise for falls-related functional mobility of frail older adults: a single-blind randomized controlled trial. Clinical rehabilitation. 2012;26(10):915-23.
494	Polymeris AA, Karwacki GM, Siepen BM, Schaedelin S, Tsakiris DA, Stippich C, et al. Tranexamic Acid for Intracerebral Hemorrhage in Patients on Non-Vitamin K Antagonist Oral Anticoagulants (TICH-NOAC): a Multicenter, Randomized, Placebo-Controlled, Phase 2 Trial. Stroke. 2023;54(9):2223-34.
495	Poon P, Hui E, Dai D, Kwok T, Woo J. Cognitive intervention for community-dwelling older persons with memory problems: telemedicine versus face-to-face treatment. International Journal of Geriatric Psychiatry. 2005;20(3):285-6.

496	Porter EJ, Ganong LH. Older widows' speculations and expectancies concerning professional home-care providers. Nursing Ethics. 2005;12(5):507-21.
497	Potter K, Flicker L, Page A, Etherton-Beer C. Deprescribing in Frail Older People: A Randomised Controlled Trial. PloS one. 2016;11(3):e0149984.
498	Prasad N, Rajamani V, Hullin D, Murray JM. Post-operative anaemia in femoral neck fracture patients: does it need treatment? A single blinded prospective randomised controlled trial. Injury. 2009;40(10):1073-6.
499	Price K, Grimmer K, Foot J. Is the Australian 75+ Health Assessment person-centred? A qualitative descriptive study of older people's perceptions. Australian health review : a publication of the Australian Hospital Association. 2017;41(6):606-12.
500	Price R, Daly F, Pennington CR, McMurdo ME. Nutritional supplementation of very old people at hospital discharge increases muscle strength: a randomised controlled trial. Gerontology. 2005;51(3):179-85.
501	Provencher V, Demers L, Gagnon L, Gelinas I. Impact of familiar and unfamiliar settings on cooking task assessments in frail older adults with poor and preserved executive functions. International psychogeriatrics. 2012;24(5):775-83.
502	Puts MTE, Sattar S, Kulik M, MacDonald ME, McWatters K, Lee K, et al. A randomized phase II trial of geriatric assessment and management for older cancer patients. Supportive care in cancer : official journal of the Multinational Association of Supportive Care in Cancer. 2018;26(1):109-17.
503	Qu B, Chen L, Zhang Y, Jiang M, Wu C, Ma W, et al. Landmark-guided versus modified ultrasound-assisted Paramedian techniques in combined spinal-epidural anesthesia for elderly patients with hip fractures: a randomized controlled trial. BMC anesthesiology. 2020;20(1):248.
504	Qu Y, Zhuang L, Zhang H, Liu C, Wang X. The Effects of Light Therapy for Depression in Dementia: A Systematic Review and Meta-Analysis. Journal of Alzheimer's disease : JAD. 2023;93(4):1223-35.
505	Quagliarello V, Juthani-Mehta M, Ginter S, Towle V, Allore H, Tinetti M. Pilot testing of intervention protocols to prevent pneumonia in nursing home residents. Journal of the American Geriatrics Society. 2009;57(7):1226-31.
506	Radhakrishnan KPRNM, Saxena SMSW, Jillapalli RRNMSN, Jang YP, Kim MPRNF. Barriers to and Facilitators of South Asian Indian-Americans' Engagement in Advanced Care Planning Behaviors. Journal of Nursing Scholarship. 2017;49(3):294-302.
507	Rao AV, Hsieh F, Feussner JR. Geriatric evaluation and management units in the care of the frail elderly cancer patient. Journals of Gerontology: Series A, Biological Sciences and Medical Sciences. 2005;60A(6).
508	Rapp MA, Mell T, Majic T, Treusch Y, Nordheim J, Niemann-Mirmehdi M, et al. Agitation in nursing home residents with dementia (VIDEANT trial): effects of a cluster-randomized, controlled, guideline implementation trial. Journal of the American Medical Directors Association. 2013;14(9):690-5.
509	Rappaport SA, Marcus RN, Manos G, McQuade RD, Oren DA. A Randomized, Double-Blind, Placebo-Controlled Tolerability Study of Intramuscular Aripiprazole in Acutely Agitated Patients With Alzheimer's, Vascular, or Mixed Dementia. Journal of the American Medical Directors Association. 2009;10(1):21-7.
510	Reid-Agboola C, Klukowska A, Malcolm FL, Harrison C, Parks RM, Cheung K-L. Comprehensive Geriatric Assessment for Older Women with Early-Stage (Non-Metastatic) Breast Cancer-An Updated Systematic Review of the Literature. Current oncology (Toronto, Ont). 2023;30(9):8294-309.
511	Resnick B, Boltz M, Galik E, Fix S, Holmes S, Zhu S, et al. Testing the Implementation of Function-focused Care in Assisted Living Settings. Journal of the American Medical Directors Association. 2021;22(8):1706-13.e1.
512	Resnick B, Boltz M, Galik E, Fix S, Holmes S, Zhu S, et al. Testing the Impact of FFC-AL-EIT on Psychosocial and Behavioral Outcomes in Assisted Living. Journal of the American Geriatrics Society. 2021;69(2):459-66.
513	Resnick B, Boltz M, Galik E, Zhu S. The Impact of a Randomized Controlled Trial Testing the Implementation of Function-Focused Care in Assisted Living on Resident Falls, Hospitalizations, and Nursing Home Transfers. Journal of aging

	and physical activity. 2021;29(6):922-30.
514	Resnick B, Galik E, Gruber-Baldini A, Zimmerman S. Testing the effect of function-focused care in assisted living. Journal of the American Geriatrics Society. 2011;59(12):2233-40.
515	Resnick B, Van Haitsma K, Kolanowski A, Galik E, Boltz M, Zhu S, et al. Implementation of the Evidence Integration Triangle for behavioral and psychological symptoms of dementia (EIT-4-BPSD) in care communities. Nursing outlook. 2021;69(6):1058-71.
516	Reuben DB, Frank JC, Hirsch SH, McGuigan KA, Maly RC. A randomized clinical trial of outpatient comprehensive geriatric assessment coupled with an intervention to increase adherence to recommendations. Journal of the American Geriatrics Society. 1999;47(3):269-76.
517	Reynolds MR, Magnuson EA, Wang K, Thourani VH, Williams M, Zajarias A, et al. Health-related quality of life after transcatheter or surgical aortic valve replacement in high-risk patients with severe aortic stenosis: results from the PARTNER (Placement of AoRTic TraNscathetER Valve) Trial (Cohort A). Journal of the American College of Cardiology. 2012;60(6):548-58.
518	Rezaei-Shahsavarloo Z, Atashzadeh-Shoorideh F, Gobbens RJJ, Ebadi A, Ghaedamini Harouni G. The impact of interventions on management of frailty in hospitalized frail older adults: a systematic review and meta-analysis. BMC geriatrics. 2020;20(1):526.
519	Rezola-Pardo C, Arrieta H, Gil SM, Zarrazquin I, Yanguas JJ, López MA, et al. Comparison between multicomponent and simultaneous dual-task exercise interventions in long-term nursing home residents: the Ageing-ONDUAL-TASK randomized controlled study. Age and ageing. 2019;48(6):817-23.
520	Richter C, Berg A, Langner H, Meyer G, Köpke S, Balzer K, et al. Effect of person-centred care on antipsychotic drug use in nursing homes (EPCentCare): a cluster-randomised controlled trial. Age and ageing. 2019;48(3):419-25.
521	Rieckert A, Reeves D, Altiner A, Drewelow E, Esmail A, Flamm M, et al. Use of an electronic decision support tool to reduce polypharmacy in elderly people with chronic diseases: cluster randomised controlled trial. BMJ (Clinical research ed). 2020;369:m1822.
522	Riemersma-van der Lek RF, Swaab DF, Twisk J, Hol EM, Hoogendijk WJ, Van Someren EJ. Effect of bright light and melatonin on cognitive and noncognitive function in elderly residents of group care facilities: a randomized controlled trial. JAMA. 2008;299(22):2642-55.
523	Riphaus A, Stergiou N, Wehrmann T. Sedation with propofol for routine ERCP in high-risk octogenarians: a randomized, controlled study. American Journal of Gastroenterology. 2005;100(9):1957-63.
524	Rizik DG, Rajagopal V, Makkar RR, Bajwa T, Kleiman NS, Linke A, et al. Long-term Outcomes of Transcatheter Aortic Valve Replacement With the Lotus Valve vs CoreValve/EvolutR: a Secondary Analysis of the REPRISE III Randomized Clinical Trial. JAMA network open. 2022;5(10):e2238792.
525	Roberts B, Holloway-Kew K, Pretorius T, Hosking S, Kennedy A, Armstrong K. Does 20-min rounding reduce falls in an aged-care setting? A pilot intervention study. Geriatric nursing (New York, NY). 2020;41(5):579-84.
526	Robinson H, Broadbent E, MacDonald B. Group sessions with Paro in a nursing home: structure, observations and interviews. Australasian journal on ageing. 2016;35(2):106-12.
527	Rockwood K, Stadnyk K, Carver D, MacPherson KM, Beanlands HE, Powell C, et al. A clinimetric evaluation of specialized geriatric care for rural dwelling, frail older people. Journal of the American Geriatrics Society. 2000;48(9):1080-5.
528	Rodondi A, Ammann P, Ghilardi-Beuret S, Rizzoli R. Zinc increases the effects of essential amino acids-whey protein supplements in frail elderly. Journal of nutrition, health & aging. 2009;13(6):491-7.
529	Rodriguez-Diaz MT, Perez-Marfil MN, Cruz-Quintana F. Coexisting with dependence and well-being: The results of a pilot study intervention on 75-99-year-old individuals. International Psychogeriatrics. 2016;28(12):2067-78.

530	Roets-Merken LM, Zuidema SU, Vernooij-Dassen M, Teerenstra S, Hermsen P, Kempen G, et al. Effectiveness of a nurse-supported self-management programme for dual sensory impaired older adults in long-term care: a cluster randomised controlled trial. BMJ open. 2018;8(1):e016674.
531	Rokstad AM, Røsvik J, Kirkevold Ø, Selbaek G, Saltyte Benth J, Engedal K. The effect of person-centred dementia care to prevent agitation and other neuropsychiatric symptoms and enhance quality of life in nursing home patients: a 10-month randomized controlled trial. Dementia and geriatric cognitive disorders. 2013;36(5-6):340-53.
532	Romeo R, Knapp M, Banerjee S, Morris J, Baldwin R, Tarrier N, et al. Treatment and prevention of depression after surgery for hip fracture in older people: cost-effectiveness analysis. Journal of affective disorders. 2011;128(3):211-9.
533	Romeo R, Zala D, Knapp M, Orrell M, Fossey J, Ballard C. Improving the quality of life of care home residents with dementia: cost-effectiveness of an optimized intervention for residents with clinically significant agitation in dementia. Alzheimer's & dementia. 2019;15(2):282-91.
534	Romskaug R, Skovlund E, Straand J, Molden E, Kersten H, Pitkala KH, et al. Effect of Clinical Geriatric Assessments and Collaborative Medication Reviews by Geriatrician and Family Physician for Improving Health-Related Quality of Life in Home-Dwelling Older Patients Receiving Polypharmacy: A Cluster Randomized Clinical Trial. JAMA internal medicine. 2020;180(2):181-9.
535	Rondanelli M, Opizzi A, Faliva M, Mozzoni M, Antoniello N, Cazzola R, et al. Effects of a diet integration with an oily emulsion of DHA-phospholipids containing melatonin and tryptophan in elderly patients suffering from mild cognitive impairment. Nutritional neuroscience. 2012;15(2):46-54.
536	Roos C, Silen M, Skytt B, Engstrom M. An intervention targeting fundamental values among caregivers at residential facilities: effects of a cluster-randomized controlled trial on residents' self-reported empowerment, person-centered climate and life satisfaction. BMC geriatrics. 2016;16:130.
537	Rosati G, Cordio S, Bordonaro R, Caputo G, Novello G, Reggiardo G, et al. Capecitabine in combination with oxaliplatin or irinotecan in elderly patients with advanced colorectal cancer: results of a randomized phase II study. Annals of oncology : official journal of the european society for medical oncology. 2010;21(4):781-6.
538	Rosenberg DE. Outcomes of a multilevel walking intervention for older adults living in retirement communities. Dissertation Abstracts International: Section B: The Sciences and Engineering. 2011;71(8-B):5143.
539	Rosendahl E, Lindelof N, Littbrand H, Yifter-Lindgren E, Lundin-Olsson L, Haglin L, et al. High-intensity functional exercise program and protein-enriched energy supplement for older persons dependent in activities of daily living: a randomised controlled trial. The Australian journal of physiotherapy. 2006;52(2):105-13.
540	Rosie J, Taylor D. Sit-to-stand as home exercise for mobility-limited adults over 80 years of age--GrandStand System[sup]TM may keep you standing? Age and Ageing. 2007;36(5):555.
541	Roukema GR, De Jong L, Van Rijckevorsel V, Van Onkelen RS, Bekken JA, Van der Vlies CH, et al. Radiation exposure during direct versus indirect image acquisition during fluoroscopy-controlled internal fixation of a hip fracture: results of a randomized controlled trial. Injury. 2019;50(12):2263-7.
542	Rubenstein LZ, Aronow HU, Schloe M, Steiner A, Alessi CA. Home-based geriatric assessment, follow-up and health promotion program: design, methods, and baseline findings from a 3-year randomized clinical trial. Aging. 1994;6(2):105-20.
543	Ruggiero C, Baroni M, Bini V, Brozzetti A, Parretti L, Zengarini E, et al. Effects of Weekly Supplementation of Cholecalciferol and Calcifediol Among the Oldest-Old People: Findings From a Randomized Pragmatic Clinical Trial. Nutrients. 2019;11(11):15.

544	Ruikes FGH, Zuidema SU, Akkermans RP, Assendelft WJJ, Schers HJ, Koopmans RTCM. Multicomponent Program to Reduce Functional Decline in Frail Elderly People: A Cluster Controlled Trial. Journal of the American Board of Family Medicine : JABFM. 2016;29(2):209-17.
545	Sackley CM, van den Berg ME, Lett K, Patel S, Hollands K, Wright CC, et al. Effects of a physiotherapy and occupational therapy intervention on mobility and activity in care home residents: a cluster randomised controlled trial. BMJ (Clinical research ed). 2009;339:b3123.
546	Sackley CM, Walker MF, Burton CR, Watkins CL, Mant J, Roalfe AK, et al. An occupational therapy intervention for residents with stroke related disabilities in UK care homes (OTCH): cluster randomised controlled trial. BMJ (Clinical research ed). 2015;350:h468.
547	Sackley CM, Walker MF, Burton CR, Watkins CL, Mant J, Roalfe AK, et al. An Occupational Therapy intervention for residents with stroke-related disabilities in UK Care Homes (OTCH): cluster randomised controlled trial with economic evaluation. Health technology assessment (Winchester, England). 2016;20(15):1-138.
548	Sáez de Asteasu ML, Martínez-Velilla N, Zambom-Ferraresi F, Casas-Herrero Á, Cadore EL, Galbete A, et al. Assessing the impact of physical exercise on cognitive function in older medical patients during acute hospitalization: Secondary analysis of a randomized trial. PLoS Medicine. 2019;16(7):1-14.
549	Sáez de Asteasu ML, Martínez-Velilla N, Zambom-Ferraresi F, Casas-Herrero Á, Lucía A, Galbete A, et al. Physical Exercise Improves Function in Acutely Hospitalized Older Patients: Secondary Analysis of a Randomized Clinical Trial. Journal of the American Medical Directors Association. 2019;20(7):866-73.
550	Sáez de Asteasu ML, Martínez-Velilla N, Zambom-Ferraresi F, Ramírez-Vélez R, García-Hermoso A, Cadore EL, et al. Changes in muscle power after usual care or early structured exercise intervention in acutely hospitalized older adults. Journal of cachexia, sarcopenia and muscle. 2020;11(4):997-1006.
551	Safari R, Jackson J, Boole L. Comprehensive geriatric assessment delivered by advanced nursing practitioners within primary care setting: a mixed-methods pilot feasibility randomised controlled trial. BMC geriatrics. 2023;23(1):513.
552	Sakamoto K, Endo N, Harada A, Sakada T, Tsushita K, Kita K, et al. Why not use your own body weight to prevent falls? A randomized, controlled trial of balance therapy to prevent falls and fractures for elderly people who can stand on one leg for ≤15 s. Journal of orthopaedic science. 2013;18(1):110-20.
553	Samefors M, Tengblad A, Östgren CJ. Sunlight Exposure and Vitamin D Levels in Older People- An Intervention Study in Swedish Nursing Homes. Journal of nutrition, health & aging. 2020;24(10):1047-52.
554	Sandberg M, Jakobsson U, Midlov P, Kristensson J. Case management for frail older people - a qualitative study of receivers' and providers' experiences of a complex intervention. BMC health services research. 2014;14:14.
555	Sandberg M, Kristensson J, Midlov P, Jakobsson U. Effects on healthcare utilization of case management for frail older people: a randomized controlled trial (RCT). Archives of gerontology and geriatrics. 2015;60(1):71-81.
556	Satoh M, Ogawa JI, Tokita T, Nakaguchi N, Nakao K, Kida H, et al. Physical Exercise with Music Maintains Activities of Daily Living in Patients with Dementia: mihama-Kiho Project Part 21. Journal of Alzheimer's disease. 2017;57(1):85-96.
557	Sattin RW, Easley KA, Wolf SL, Chen Y, Kutner MH. Reduction in fear of falling through intense tai chi exercise training in older, transitionally frail adults. Journal of the American Geriatrics Society. 2005;53(7):1168-78.
558	Savolainen L. An Internet-based videoconferencing system for supporting frail elderly people and their carers. Journal of Telemedicine and Telecare. 2008;14(2):79-82.
559	Schapira M, Outumuro MB, Giber F, Pino C, Mattiussi M, Montero-Odasso M, et al. Geriatric co-management and interdisciplinary transitional care reduced hospital readmissions in frail older patients in Argentina: results from a randomized controlled trial. Aging clinical and experimental research. 2022;34(1):85-93.

560	Scherder E. J A, al. e. Physical activity and executive functions in the elderly with mild cognitive impairment. Aging and Mental Health. 2005;9(3):272-80.
561	Scherder EJ, Van Paasschen J, Deijen J, Van Der Knokke S, Orlebeke JF, Burgers I, et al. Physical activity and executive functions in the elderly with mild cognitive impairment. Aging & Mental Health. 2005;9(3):272-80.
562	Schlender L, Martinez YV, Adeniji C, Reeves D, Faller B, Sommerauer C, et al. Efficacy and safety of metformin in the management of type 2 diabetes mellitus in older adults: a systematic review for the development of recommendations to reduce potentially inappropriate prescribing. BMC Geriatrics. 2017;17(Suppl 1):227.
563	Schmidt-Mende K, Andersen M, Wettermark B, Hasselstrom J. Educational intervention on medication reviews aiming to reduce acute healthcare consumption in elderly patients with potentially inappropriate medicines-A pragmatic open-label cluster-randomized controlled trial in primary care. Pharmacoepidemiology & Drug Safety. 2017;26(11):1347-56.
564	Schwenk M, Grewal GS, Honarvar B, Schwenk S, Mohler J, Khalsa DS, et al. Interactive balance training integrating sensor-based visual feedback of movement performance: a pilot study in older adults. Journal of neuroengineering and rehabilitation. 2014;11:164.
565	Schwindling FS, Krisam J, Hassel AJ, Rammelsberg P, Zenthöfer A. Long-term success of oral health intervention among care-dependent institutionalized seniors: findings from a controlled clinical trial. Community dentistry and oral epidemiology. 2018;46(2):109-17.
566	Selman LE, Bristowe K, Higginson IJ, Murtagh FEM. The views and experiences of older people with conservatively managed renal failure: a qualitative study of communication, information and decision-making. BMC Nephrology. 2019;20(1):38.
567	Senior HEJ, Parsons M, Kerse N, Chen M-H, Jacobs S, Hoorn SV, et al. Promoting independence in frail older people: a randomised controlled trial of a restorative care service in New Zealand. Age and ageing. 2014;43(3):418-24.
568	Serra-Rexach JA, Bustamante-Ara N, Hierro Villarán M, González Gil P, Sanz Ibáñez MJ, Blanco Sanz N, et al. Short-term, light- to moderate-intensity exercise training improves leg muscle strength in the oldest old: a randomized controlled trial. Journal of the American Geriatrics Society. 2011;59(4):594-602.
569	Serrani Azcurra DJ. A reminiscence program intervention to improve the quality of life of long-term care residents with Alzheimer's disease: a randomized controlled trial. Revista brasileira de psiquiatria (Sao Paulo, Brazil : 1999). 2012;34(4):422-33.
570	Shah MN, Morris D, Jones CMC, Gillespie SM, Nelson DL, McConnochie KM, et al. A qualitative evaluation of a telemedicine-enhanced emergency care program for older adults. Journal of the American Geriatrics Society. 2013;61(4):571-6.
571	Shaw C. Systematic review of respite care in the frail elderly. Health Technology Assessment. 2009;13(20).
572	Shepperd S, Butler C, Cradduck-Bamford A, Ellis G, Gray A, Hemsley A, et al. Is Comprehensive Geriatric Assessment Admission Avoidance Hospital at Home an Alternative to Hospital Admission for Older Persons? : A Randomized Trial. Annals of internal medicine. 2021;174(7):889-98.
573	Shepperd S, Cradduck-Bamford A, Butler C, Ellis G, Godfrey M, Gray A, et al. Hospital at Home admission avoidance with comprehensive geriatric assessment to maintain living at home for people aged 65 years and over: a RCT. 2022.
574	Sherrington C, Lord SR, Vogler CM, Close JC, Howard K, Dean CM, et al. A post-hospital home exercise program improved mobility but increased falls in older people: a randomised controlled trial. PloS one. 2014;9(9):e104412.
575	Shuman CJ, Xie XJ, Herr KA, Titler MG. Sustainability of Evidence-Based Acute Pain Management Practices for Hospitalized Older Adults. Western journal of nursing research. 2018;40(12):1749-64.

576	Siegrist M, Freiberger E, Geilhof B, Salb J, Hentschke C, Landendoerfer P, et al. Fall Prevention in a Primary Care Setting. Deutsches Arzteblatt international. 2016;113(21):365-72.
577	Sihvonen S, Sipila S, Taskinen S, Era P. Fall incidence in frail older women after individualized visual feedback-based balance training. Gerontology. 2004;50(6):411-6.
578	Sihvonen SE, Sipila S, Era PA. Changes in postural balance in frail elderly women during a 4-week visual feedback training: a randomized controlled trial. Gerontology. 2004;50(2):87-95.
579	Silver HJ, Dietrich MS, Castellanos VH. Increased energy density of the home-delivered lunch meal improves 24-hour nutrient intakes in older adults. Journal of the American Dietetic Association. 2008;108(12):2084-9.
580	Simmons SF, Hollingsworth EK, Long EA, Liu X, Shotwell MS, Keeler E, et al. Training Nonnursing Staff to Assist with Nutritional Care Delivery in Nursing Homes: a Cost-Effectiveness Analysis. Journal of the American Geriatrics Society. 2017;65(2):313-22.
581	Simmons SF, Keeler E, An R, Liu X, Shotwell MS, Kuertz B, et al. Cost-Effectiveness of Nutrition Intervention in Long-Term Care. Journal of the American Geriatrics Society. 2015;63(11):2308-16.
582	Simmons SF, Zhuo X, Keeler E. Cost-effectiveness of nutrition interventions in nursing home residents: a pilot intervention. Journal of nutrition, health & aging. 2010;14(5):367-72.
583	Simons D, Brailsford SR, Kidd EAM, Beighton D. The effect of medicated chewing gums on oral health in frail older people: a 1-year clinical trial. Journal of the American Geriatrics Society. 2002;50(8):1348-53.
584	Simons R, Andel R. The effects of resistance training and walking on functional fitness in advanced old age. Journal of Aging & Health. 2006;18(1):91-105.
585	Singh S, Gray A, Shepperd S, Stott DJ, Ellis G, Hemsley A, et al. Is comprehensive geriatric assessment hospital at home a cost-effective alternative to hospital admission for older people? Age and ageing. 2022;51(1).
586	Sjosten NM, Vahlberg TJ, Kivela S-L. The effects of multifactorial fall prevention on depressive symptoms among the aged at increased risk of falling. International journal of geriatric psychiatry. 2008;23(5):504-10.
587	Skelton DA, Young A, Greig CA, Malbut KE. Effects of resistance training on strength, power, and selected functional abilities of women aged 75 and older. Journal of the American Geriatrics Society. 1995;43(10):1081-7.
588	Sloane PD, Cohen LW, Williams CS, Munn J, Preisser JS, Sobsey MD, et al. Effect of specialized bathing systems on resident cleanliness and water quality in nursing homes: a randomized controlled trial. Journal of water and health. 2007;5(2):283-94.
589	Smith-Carrier T, Sinha sK, Nowaczynski M. It 'makes you feel more like a person than a patient'. Health and Social Care in the Community. 2017;25(2).
590	Snooks HA, Anthony R, Chatters R, Dale J, Fothergill RT, Gaze S, et al. Paramedic Assessment of Older Adults After Falls, Including Community Care Referral Pathway: Cluster Randomized Trial. Annals of emergency medicine. 2017;70(4):495-505.e28.
591	Sondell A, Littbrand H, Holmberg H, Lindelöf N, Rosendahl E. Is the Effect of a High-Intensity Functional Exercise Program on Functional Balance Influenced by Applicability and Motivation among Older People with Dementia in Nursing Homes? Journal of nutrition, health & aging. 2019;23(10):1011-20.
592	Soukkio PK, Suikkanen SA, Kukkonen-Harjula KT, Kautiainen H, Hupli MT, Aartolahti EM, et al. Effects of a 12-month home-based exercise program on functioning after hip fracture - Secondary analyses of an RCT. Journal of the American Geriatrics Society. 2022;70(9):2561-70.
593	Spencer S, Johnson P, Smith IC. De-escalation techniques for managing non-psychosis induced aggression in adults. Cochrane Database of Systematic Reviews. 2018(7).

594	Spice CL, Morotti W, George S, Dent THS, Rose J, Harris S, et al. The Winchester falls project: a randomised controlled trial of secondary prevention of falls in older people. Age and ageing. 2009;38(1):33-40.
595	Spinewine A, Swine C, Dhillon S, Lambert P, Nachega JB, Wilmotte L, et al. Effect of a collaborative approach on the quality of prescribing for geriatric inpatients: a randomized, controlled trial. Journal of the American Geriatrics Society. 2007;55(5):658-65.
596	Stanziano DC, Roos BA, Perry AC, Lai S, Signorile JF. The effects of an active-assisted stretching program on functional performance in elderly persons: a pilot study. Clinical interventions in aging. 2009;4:115-20.
597	Stappaerts KH, Deldycke J, Broos PL, Staes FF, Rommens PM, Claes P. Treatment of unstable peritrochanteric fractures in elderly patients with a compression hip screw or with the Vandeputte (VDP) endoprosthesis: a prospective randomized study. Journal of Orthopaedic Trauma. 1995;9(4):292-7.
598	Stein MS, Scherer SC, Ladd KS, Harrison LC. A randomized controlled trial of high-dose vitamin D2 followed by intranasal insulin in Alzheimer's disease. Journal of Alzheimer's disease. 2011;26(3):477-84.
599	Steinmetz JP, Federspiel C. The effects of cognitive training on gait speed and stride variability in old adults: findings from a pilot study. Aging clinical and experimental research. 2014;26(6):635-43.
600	Stenvall M, Olofsson B, Nyberg L, Lundstrom M, Gustafson Y. Improved performance in activities of daily living and mobility after a multidisciplinary postoperative rehabilitation in older people with femoral neck fracture: a randomized controlled trial with 1-year follow-up. Journal of rehabilitation medicine. 2007;39(3):232-8.
601	Stenzelius K, Molander U, Odeberg J, Hammarstrom M, Franzen K, Midlov P, et al. The effect of conservative treatment of urinary incontinence among older and frail older people: a systematic review. Age and ageing. 2015;44(5):736-44.
602	Stevens M, Holman CD, Bennett N. Preventing falls in older people: impact of an intervention to reduce environmental hazards in the home. Journal of the American Geriatrics Society. 2001;49(11):1442-7.
603	Stevens M, Holman CD, Bennett N, de Klerk N. Preventing falls in older people: outcome evaluation of a randomized controlled trial. Journal of the American Geriatrics Society. 2001;49(11):1448-55.
604	Strasser EM, Hofmann M, Franzke B, Schober-Halper B, Oesen S, Jandrasits W, et al. Strength training increases skeletal muscle quality but not muscle mass in old institutionalized adults: a randomized, multi-arm parallel and controlled intervention study. European journal of physical and rehabilitation medicine. 2018;54(6):921-33.
605	Stuck AE, Egger M, Hammer A, Minder CE, Beck JC. Home visits to prevent nursing home admission and functional decline in elderly people: systematic review and meta-regression analysis. JAMA. 2002;287(8):1022-8.
606	Suijker JJ, MacNeil-Vroomen JL, van Rijn M, Buurman BM, de Rooij SE, Moll van Charante EP, et al. Cost-effectiveness of nurse-led multifactorial care to prevent or postpone new disabilities in community-living older people: Results of a cluster randomized trial. PloS one. 2017;12(4):e0175272.
607	Suijker JJ, van Rijn M, Buurman BM, Ter Riet G, Moll van Charante EP, de Rooij SE. Effects of Nurse-Led Multifactorial Care to Prevent Disability in Community-Living Older People: Cluster Randomized Trial. PloS one. 2016;11(7):e0158714.
608	Sumi Y, Ozawa N, Miura H, Michiwaki Y, Umemura O. Oral care help to maintain nutritional status in frail older people. Archives of gerontology and geriatrics. 2010;51(2):125-8.
609	Sun S, Sun D, Yang L, Han J, Liu R, Wang L. Dose-dependent effects of intravenous methoxamine infusion during hip-joint replacement surgery on postoperative cognitive dysfunction and blood TNF-α level in elderly patients: a randomized controlled trial. BMC anesthesiology. 2017;17(1):75.

610	Suttanon P, Hill KD, Said CM, Williams SB, Byrne KN, LoGiudice D, et al. Feasibility, safety and preliminary evidence of the effectiveness of a home-based exercise programme for older people with Alzheimer's disease: a pilot randomized controlled trial. Clinical rehabilitation. 2013;27(5):427-38.
611	Suzuki M, Hattori H, Abe K, Nakamura Y, Saruhara T. Utility of a Life-trouble Scale-based care planning tool for elderly patients living in a long-term care health facility: effects of an intervention based on person-centered care. Nihon Ronen Igakkai zasshi [Japanese journal of geriatrics]. 2019;56(3):312-22.
612	Swamy B, Cumming RG, Ivers R, Clemson L, Cullen J, Hayes MF, et al. Vision screening for frail older people: a randomised trial. The British journal of ophthalmology. 2009;93(6):736-41.
613	Szturm T, Betker AL, Moussavi Z, Desai A, Goodman V. Effects of an interactive computer game exercise regimen on balance impairment in frail community-dwelling older adults: a randomized controlled trial. Physical therapy. 2011;91(10):1449-62.
614	Takeuchi S, Motohashi J, Kimori H, Nakagawa Y, Tsurumoto A. Effects of oral moisturising gel containing egg yolk antibodies against Candida albicans in older people. Gerodontology. 2016;33(1):128-34.
615	Talley KMC, Wyman JF, Bronas U, Olson-Kellogg BJ, McCarthy TC. Defeating Urinary Incontinence with Exercise Training: Results of a Pilot Study in Frail Older Women. Journal of the American Geriatrics Society. 2017;65(6):1321-7.
616	Tanajewski L, Franklin M, Gkountouras G, Berdunov V, Edmans J, Conroy S, et al. Cost-Effectiveness of a Specialist Geriatric Medical Intervention for Frail Older People Discharged from Acute Medical Units: Economic Evaluation in a Two-Centre Randomised Controlled Trial (AMIGOS). PloS one. 2015;10(5):e0121340.
617	Taraldsen K, Sletvold O, Thingstad P, Saltvedt I, Granat MH, Lydersen S, et al. Physical behavior and function early after hip fracture surgery in patients receiving comprehensive geriatric care or orthopedic care--a randomized controlled trial. The journals of gerontology Series A, Biological sciences and medical sciences. 2014;69(3):338-45.
618	Taube E, Kristensson J, Midlov P, Jakobsson U. The use of case management for community-dwelling older people: the effects on loneliness, symptoms of depression and life satisfaction in a randomised controlled trial. Scandinavian journal of caring sciences. 2018;32(2):889-901.
619	Taylor ME, Wesson J, Sherrington C, Hill KD, Kurrle S, Lord SR, et al. Tailored Exercise and Home Hazard Reduction Program for Fall Prevention in Older People With Cognitive Impairment: the i-FOCIS Randomized Controlled Trial. Journals of gerontology Series A, Biological sciences and medical sciences. 2021;76(4):655-65.
620	Teh R, Barnett D, Edlin R, Kerse N, Waters DL, Hale L, et al. Effectiveness of a complex intervention of group-based nutrition and physical activity to prevent frailty in pre-frail older adults (SUPER): a randomised controlled trial. The lancet Healthy longevity. 2022;3(8):e519-e30.
621	Terp R, Jacobsen KO, Kannegaard P, Larsen AM, Madsen OR, Noiesen E. A nutritional intervention program improves the nutritional status of geriatric patients at nutritional risk-a randomized controlled trial. Clinical rehabilitation. 2018;32(7):930-41.
622	Testad I, Ballard C, Brønnick K, Aarsland D. The effect of staff training on agitation and use of restraint in nursing home residents with dementia: a single-blind, randomized controlled trial. Journal of clinical psychiatry. 2010;71(1):80-6.
623	Thivierge S, Jean L, Simard M. A randomized cross-over controlled study on cognitive rehabilitation of instrumental activities of daily living in Alzheimer disease. American journal of geriatric psychiatry. 2014;22(11):1188-99.
624	Thomson K, Rice S, Arisa O, Johnson E, Tanner L, Marshall C, et al. Oral nutritional interventions in frail older people who are malnourished or at risk of malnutrition: a systematic review. Health technology assessment (Winchester, England). 2022;26(51):1-112.

625	Thomson KH, Rice S, Arisa O, Johnson E, Tanner L, Marshall C, et al. Effectiveness and cost-effectiveness of oral nutritional supplements in frail older people who are malnourished or at risk of malnutrition: a systematic review and meta-analysis. The lancet Healthy longevity. 2022;3(10):e654-e66.
626	Tibaldi V, Isaia G, Bergerone S, Moiraghi C, Gariglio F, Marchetto C, et al. A randomized clinical trial on the efficacy of an early discharge to a hospital at home service of elderly patients with acute decompensation of severe chronic heart failure. Giornale di gerontologia. 2013;61(2):78-85.
627	Timmer AJ, Unsworth CA, Browne M. Occupational therapy and activity pacing with hospital-associated deconditioned older adults: a randomised controlled trial. Disability and rehabilitation. 2020;42(12):1727-35.
628	Timonen L, Rantanen T, Makinen E, Timonen TE, Tormakangas T, Sulkava R. Effects of a group-based exercise program on functional abilities in frail older women after hospital discharge. Aging-Clinical & Experimental Research. 2006;18(1):50-6.
629	Timonen L, Rantanen T, Makinen E, Timonen TE, Tormakangas T, Sulkava R. Effects of group-based exercise program on functional abilities in frail older women after hospital discharge. Aging clinical and experimental research. 2006;18(1):50-6.
630	Timonen L, Rantanen T, Ryynanen O-P, Taimela S, Timonen TE, Sulkava R. A randomized controlled trial of rehabilitation after hospitalization in frail older women: effects on strength, balance and mobility. Scandinavian journal of medicine & science in sports. 2002;12(3):186-92.
631	Timonen L, Rantanen T, Timonen TE. Effects of a group based exercise program on the mood state of frail older women after discharge from hospital. International Journal of Geriatric Psychiatry. 2002;17(12).
632	Timonen L, Rantanen T, Timonen TE, Sulkava R. Effects of a group-based exercise program on the mood state of frail older women after discharge from hospital. International Journal of Geriatric Psychiatry. 2002;17(12):1106-11.
633	Tjia J, Velten SJ, Parsons C, Valluri S, Briesacher BA. Studies to reduce unnecessary medication use in frail older adults: a systematic review. Drugs & aging. 2013;30(5):285-307.
634	Toots A, Wiklund R, Littbrand H, Nordin E, Nordström P, Lundin-Olsson L, et al. The Effects of Exercise on Falls in Older People With Dementia Living in Nursing Homes: a Randomized Controlled Trial. Journal of the American Medical Directors Association. 2019;20(7):835-42.e1.
635	Torra iBJ, Segovia Gomez T, Verdu Soriano J, Nolasco Bonmati A, Rueda Lopez J, Arboix iPM. The effectiveness of a hyperoxygenated fatty acid compound in preventing pressure ulcers. EWMA journal. 2005;5(2):27-31.
636	Tosi FC, Lin SM, Gomes GC, Aprahamian I, Nakagawa NK, Viveiro L, et al. A multidimensional program including standing exercises, health education, and telephone support to reduce sedentary behavior in frail older adults: Randomized clinical trial. Experimental gerontology. 2021;153:111472.
637	Tou NX, Wee S-L, Seah WT, Ng DHM, Pang BWJ, Lau LK, et al. Effectiveness of Community-Delivered Functional Power Training Program for Frail and Pre-frail Community-Dwelling Older Adults: a Randomized Controlled Study. Prevention science : the official journal of the Society for Prevention Research. 2021;22(8):1048-59.
638	Toulotte C, Fabre C, Dangremont B. Effects of physical training on the physical capacity of frail, demented patients with a history of falling. Age and Ageing. 2003;32(1).
639	Tousignant M. Efficacy of supervised Tai Chi exercises versus conventional physical therapy exercises in fall prevention for frail older adults : a randomized controlled trial. Disability and Rehabilitation. 2013.
640	Tousignant M, Corriveau H, Roy P-M, Desrosiers J, Dubuc N, Hebert R. Efficacy of supervised Tai Chi exercises versus conventional physical therapy exercises in fall prevention for frail older adults: a randomized controlled trial. Disability and rehabilitation. 2013;35(17):1429-35.

641	Tracy S-C, et al. It 'makes you feel more like a person than a patient': patients' experiences receiving home-based primary care (HBPC) in Ontario, Canada. Health and Social Care in the Community. 2017;25(2):723-33.
642	Trivalle C, Cartier T, Verny C, Mathieu AM, Davrinche P, Agostini H, et al. Identifying and preventing adverse drug events in elderly hospitalised patients: a randomised trial of a program to reduce adverse drug effects. Journal of nutrition, health & aging. 2010;14(1):57-61.
643	Trombetti A, Hars M, Hsu F-C, Reid KF, Church TS, Gill TM, et al. Effect of Physical Activity on Frailty: Secondary Analysis of a Randomized Controlled Trial. Annals of internal medicine. 2018;168(5):309-16.
644	Tse MM, Tang SK, Wan VT, Vong SK. The effectiveness of physical exercise training in pain, mobility, and psychological well-being of older persons living in nursing homes. Pain management nursing. 2014;15(4):778-88.
645	Tse MMY, Ng SSM, Lee PH, Lai C, Kwong E, Liu JYW, et al. Play Activities Program to Relieve Chronic Pain and Enhance Functional Mobility and Psychological Well-Being for Frail Older Adults: A Pilot Cluster Randomized Controlled Trial. Journal of the American Geriatrics Society. 2016;64(10):e86-e8.
646	Tseng M-Y, Yang C-T, Liang J, Huang H-L, Kuo L-M, Wu C-C, et al. A family care model for older persons with hip-fracture and cognitive impairment: A randomized controlled trial. International journal of nursing studies. 2021;120:103995.
647	Valenzuela PL, Ortiz-Alonso J, Bustamante-Ara N, Vidan MT, Rodriguez-Romo G, Mayordomo-Cava J, et al. Individual responsiveness to physical exercise intervention in acutely hospitalized older adults. Journal of clinical medicine. 2020;9(3).
648	Van Craen K, Braes T, Wellens N, Denhaerynck K, Flamaing J, Moons P, et al. The effectiveness of inpatient geriatric evaluation and management units: a systematic review and meta-analysis. Journal of the American Geriatrics Society. 2010;58(1):83-92.
649	van der Putten GJ, Mulder J, de Baat C, De Visschere LM, Vanobbergen JN, Schols JM. Effectiveness of supervised implementation of an oral health care guideline in care homes; a single-blinded cluster randomized controlled trial. Clinical oral investigations. 2013;17(4):1143-53.
650	van der Weele GM, de Jong R, de Waal MWM, Spinhoven P, Rooze HAH, Reis R, et al. Response to an unsolicited intervention offer to persons aged ≥ 75 years after screening positive for depressive symptoms: a qualitative study. International Psychogeriatrics. 2012;24(2):270-7.
651	van der Weele GM, de Waal MWM, van den Hout WB, de Craen AJM, Spinhoven P, Stijnen T, et al. Effects of a stepped-care intervention programme among older subjects who screened positive for depressive symptoms in general practice: the PROMODE randomised controlled trial. Age and Ageing. 2012;41(4):482-8.
652	van Halsema MS, Boers RAR, Leferink VJM. An overview on the treatment and outcome factors of ankle fractures in elderly men and women aged 80 and over: a systematic review. Archives of orthopaedic and trauma surgery. 2022;142(11):3311-25.
653	van Hout HP, Jansen AP, van Marwijk HW, Pronk M, Frijters DF, Nijpels G. Prevention of adverse health trajectories in a vulnerable elderly population through nurse home visits: a randomized controlled trial. Journals of gerontology Series A, Biological sciences and medical sciences. 2010;65(7):734-42.
654	van Hout HPJ, Jansen APD, van Marwijk HWJ, Pronk M, Frijters DF, Nijpels G. Prevention of adverse health trajectories in a vulnerable elderly population through nurse home visits: a randomized controlled trial [ISRCTN05358495]. The journals of gerontology Series A, Biological sciences and medical sciences. 2010;65(7):734-42.
655	van Kempen JAL, Robben SHM, Zuidema SU. Home visits for frail older people: a qualitative study on the needs and preferences of frail older people and their informal caregivers. British Journal of General Practice. 2012;62(601):417-8.

656	van Leen M, Hovius S, Neyens J, Halfens R, Schols J. Pressure relief, cold foam or static air? A single center, prospective, controlled randomized clinical trial in a Dutch nursing home. Journal of tissue viability. 2011;20(1):30-4.
657	van Leeuwen KM, Bosmans JE, Jansen APD, Hoogendijk EO, Muntinga ME, van Hout HPJ, et al. Cost-Effectiveness of a Chronic Care Model for Frail Older Adults in Primary Care: Economic Evaluation Alongside a Stepped-Wedge Cluster-Randomized Trial. Journal of the American Geriatrics Society. 2015;63(12):2494-504.
658	Van Roie E, Martien S, Hurkmans E, Pelssers J, Seghers J, Boen F, et al. Ergometer-cycling with strict versus minimal contact supervision among the oldest adults: A cluster-randomised controlled trial. Archives of Gerontology & Geriatrics. 2017;70:112-22.
659	van Weert J, van Munster B, Sanders R. Decision aids to help older people make health decisions: a systematic review and meta-analysis. BMC Medical Informatics and Decision Making. 2016;16(45).
660	van Weert JCM, van Munster BC, Sanders R, Spijker R, Hooft L, Jansen J. Decision aids to help older people make health decisions: a systematic review and meta-analysis. BMC Medical Informatics & Decision Making. 2016;16:1-20.
661	van Zon L, Kirby JR, Anderson N. The efficacy of a volunteer-administered cognitive stimulation program in long-term care homes. International psychogeriatrics. 2016;28(6):995-1004.
662	van't Veer-Tazelaar PJ, van Marwijk HW, van Oppen P, van der Horst HE, Smit F, Cuijpers P, et al. Prevention of late-life anxiety and depression has sustained effects over 24 months: a pragmatic randomized trial. American journal of geriatric psychiatry. 2011;19(3):230-9.
663	Vanderwee K, Grypdonck MH, De Bacquer D, Defloor T. Effectiveness of turning with unequal time intervals on the incidence of pressure ulcer lesions. Journal of advanced nursing. 2007;57(1):59-68.
664	Vass M, Avlund K, Lauridsen J, Hendriksen C. Feasible model for prevention of functional decline in older people: municipality-randomized, controlled trial. Journal of the American Geriatrics Society. 2005;53(4):563-8.
665	Vass M, Avlund K, Siersma V, Hendriksen C. A feasible model for prevention of functional decline in older home-dwelling people--the GP role. A municipality-randomized intervention trial. Family practice. 2009;26(1):56-64.
666	Veleva BI, Caljouw MAA, Muurman A, van der Steen JT, Chel VGM, Numans ME, et al. The effect of ultraviolet irradiation compared to oral vitamin D supplementation on blood pressure of nursing home residents with dementia. BMC geriatrics. 2021;21(1):577.
667	Vemmos KN, Tsivgoulis G, Spengos K, Manios E, Xinos K, Vassilopoulou S, et al. Primary prevention of arterial thromboembolism in the oldest old with atrial fibrillation--a randomized pilot trial comparing adjusted-dose and fixed low-dose coumadin with aspirin. European Journal of Internal Medicine. 2006;17(1):48-52.
668	Vemmos KN, Tsivgoulis G, Spengos K, Manios E, Xinos K, Vassilopoulou S, et al. Primary prevention of arterial thromboembolism in the oldest old with atrial fibrillation - A randomized pilot trial comparing adjusted-dose and fixed low-dose coumadin with aspirin. European journal of internal medicine. 2006;17(1):48-52.
669	Venables D, Clarkson P, Hughes J, Burns A, Challis D. Specialist clinical assessment of vulnerable older people: outcomes for carers from a randomised controlled trial. Ageing and Society. 2006;26(6):867-82.
670	Venturelli M, Lanza M, Muti E, Schena F. Positive effects of physical training in activity of daily living-dependent older adults. Experimental aging research. 2010;36(2):190-205.
671	Venturelli M, Scarsini R, Schena F. Six-month walking program changes cognitive and ADL performance in patients with Alzheimer. American journal of Alzheimer's disease and other dementias. 2011;26(5):381-8.
672	Verrusio W, Renzi A, Cecchetti F, Gaj F, Coi M, Ripani M, et al. The Effect of a Physical Training with the Use of an Exoskeleton on Depression Levels in Institutionalized Elderly Patients: a Pilot Study. Journal of nutrition, health & aging. 2018;22(8):934-7.

673	Vestergaard S, Kronborg C, Puggaard L. Home-based video exercise intervention for community-dwelling frail older women: a randomized controlled trial. Aging clinical and experimental research. 2008;20(5):479-86.
674	Vestjens L, Cramm JM, Nieboer AP. An integrated primary care approach for frail community-dwelling older persons: a step forward in improving the quality of care. BMC Health Services Research. 2018;18(28).
675	Vicente V, Svensson L, Wireklint Sundstrom B, Sjostrand F, Castren M. Randomized controlled trial of a prehospital decision system by emergency medical services to ensure optimal treatment for older adults in Sweden. Journal of the American Geriatrics Society. 2014;62(7):1281-7.
676	Vidan M, Serra JA, Moreno C, Riquelme G, Ortiz J. Efficacy of a comprehensive geriatric intervention in older patients hospitalized for hip fracture: a randomized, controlled trial. Journal of the American Geriatrics Society. 2005;53(9):1476-82.
677	Villani R, Monami M, Di Cosimo F, Fioravanti G, Mannucci E, Vendemiale G, et al. Direct-acting antivirals for HCV treatment in older patients: A systematic review and meta-analysis. Journal of viral hepatitis. 2019;26(11):1249-56.
678	Villareal DT, Steger-May K, Schechtman KB, Yarasheski KE, Brown M, Sinacore DR, et al. Effects of exercise training on bone mineral density in frail older women and men: a randomised controlled trial. Age and ageing. 2004;33(3):309-12.
679	Vincenzo JL, Patton SK. Older Adults' Experience With Fall Prevention Recommendations Derived From the STEADI. Health promotion practice. 2021;22(2):236-47.
680	Vinks TH, Egberts TC, de Lange TM, de Koning FH. Pharmacist-based medication review reduces potential drug-related problems in the elderly: the SMOG controlled trial. Drugs & aging. 2009;26(2):123-33.
681	Wadsworth D, Turnbull J, Lark S. Psychological Effects of Whole-Body Vibration Training in Frail Older Adults: An Open, Randomized Control Trial. Journal of aging and physical activity. 2022;30(1):54-64.
682	Wagner KH, Haber P, Elmadfa I. Thanks to body exercise, getting mobile and being less dependent. Annals of nutrition & metabolism. 2008;52 Suppl 1:38-42.
683	Waite, Linda, Carey KM. The lived experiences of the independent oldest old in community -based programs: A Heideggerian hermeneutical analysis 2004.
684	Wales K, Salkeld G, Clemson L, Lannin NA, Gitlin L, Rubenstein L, et al. A trial based economic evaluation of occupational therapy discharge planning for older adults: the HOME randomized trial. Clinical rehabilitation. 2018;32(7):919-29.
685	Walker R, Johns J, Halliday D. How older people cope with frailty within the context of transition care in Australia: implications for improving service delivery. Health & social care in the community. 2015;23(2):216-24.
686	Walton K, do Rosario VA, Pettingill H, Cassimatis E, Charlton K. The impact of home-delivered meal services on the nutritional intake of community living older adults: a systematic literature review. Journal of human nutrition and dietetics : the official journal of the British Dietetic Association. 2020;33(1):38-47.
687	Wang S, Yin H, Meng X, Shang B, Meng Q, Zheng L, et al. Effects of Chinese square dancing on older adults with mild cognitive impairment. Geriatric nursing (New York, NY). 2020;41(3):290-6.
688	Wang YT, Li Z, Yang Y, Zhong Y, Lee SY, Chen S, et al. Effects of wheelchair Tai Chi on physical and mental health among elderly with disability. Research in sports medicine (Print). 2016;24(3):157-70.
689	Watanabe Y, Hayashida K, Yamamoto M, Yamanaka F, Yamasaki K, Naganuma T, et al. Edoxaban vs. Vitamin K Antagonist for Atrial Fibrillation After Transcatheter Aortic Valve Replacement in Japanese Patients - A Subanalysis of the ENVISAGE-TAVI AF Trial. Circulation journal. 2022;86(11):1756-63.
690	Waterworth S, Raphael D, Parsons J, Arroll B, Gott M. Older people's experiences of nurse-patient telephone communication in the primary healthcare setting. Journal of advanced nursing. 2018;74(2):373-82.

691	Weber M, Klein U, Weigert A, Schiller W, Bayley-Ezziddin V, Wirtz DC, et al. Use of Pre- and Intensified Postprocedural Physiotherapy in Patients with Symptomatic Aortic Stenosis Undergoing Transcatheter Aortic Valve Replacement Study (the 4P-TAVR Study). Journal of interventional cardiology. 2021;2021:8894223.
692	Weingessel B, Mihaltz K, Vécsei-Marlovits PV. Predictors of 1-year visual outcome in OCT analysis comparing ranibizumab monotherapy versus combination therapy with PDT in exsudative age-related macular degeneration. Wiener klinische Wochenschrift. 2016;128(15-16):560-5.
693	Weng W-H, Cheng Y-H, Yang T-H, Lee S-J, Yang Y-R, Wang R-Y. Effects of strength exercises combined with other training on physical performance in frail older adults: A systematic review and meta-analysis. Archives of gerontology and geriatrics. 2022;102:104757.
694	Werner C, Moustris GP, Tzafestas CS, Hauer K. User-Oriented Evaluation of a Robotic Rollator That Provides Navigation Assistance in Frail Older Adults with and without Cognitive Impairment. Gerontology. 2018;64(3):278-90.
695	Westgard T, Andersson Hammar I, Dahlin-Ivanoff S, Wilhelmson K. Can Comprehensive Geriatric Assessment Meet Frail Older People's Needs? Results from the Randomized Controlled Study CGA-Swed. Geriatrics (Basel, Switzerland). 2020;5(4).
696	Westgard T, Hammar IA, Wilhelmson K, Waern M. Comprehensive geriatric assessment is associated with increased antidepressant treatment in frail older people with unplanned hospital admissions-results from the randomised controlled study CGA-Swed. BMC geriatrics. 2022;22(1):645.
697	Westgard T, Ottenvall Hammar I, Holmgren E, Ehrenberg A, Wisten A, Ekdahl AW, et al. Comprehensive geriatric assessment pilot of a randomized control study in a Swedish acute hospital: a feasibility study. Pilot and feasibility studies. 2018;4:41.
698	Westgard T, Wilhelmson K, Dahlin-Ivanoff S, Ottenvall Hammar I. Feeling Respected as a Person: a Qualitative Analysis of Frail Older People's Experiences on an Acute Geriatric Ward Practicing a Comprehensive Geriatric Assessment. Geriatrics (Basel, Switzerland). 2019;4(1).
699	Whitman A, DeGregory K, Morris A, Mohile S, Ramsdale E. Pharmacist-led medication assessment and deprescribing intervention for older adults with cancer and polypharmacy: a pilot study. Supportive care in cancer : official journal of the Multinational Association of Supportive Care in Cancer. 2018;26(12):4105-13.
700	Whitney J, Jackson SHD, Martin FC. Feasibility and efficacy of a multi-factorial intervention to prevent falls in older adults with cognitive impairment living in residential care (ProF-Cog). A feasibility and pilot cluster randomised controlled trial. BMC geriatrics. 2017;17(1):115.
701	Wichmann AB, Adang EMM, Vissers KCP, Szczerbińska K, Kylänen M, Payne S, et al. Decreased costs and retained QoL due to the 'PACE Steps to Success' intervention in LTCFs: cost-effectiveness analysis of a randomized controlled trial. BMC medicine. 2020;18(1):258.
702	Widell C, Andreen S, Albertsson P, Axelsson AB. Octogenarian preferences and expectations for acute coronary syndrome treatment. European Journal of Cardiovascular Nursing. 2020;19(6):521-8.
703	Widimsky J. Treatment of very elderly hypertensives significantly reduces total mortality and the risk of death from stroke. Results of the HYVET trial. Cor et vasa. 2008;50(9):354-7.
704	Wieland J, Almodallal Y, Martin NA, Mannion S, Nguyen P, Jatoi A. Understanding "Patient refuses" among 90+ year old patients with cancer or presumed cancer. Journal of Geriatric Oncology. 2022;13(5):715-9.
705	Wigley J, Shantikumar S, Hameed W, Griffin K, Handa A, Scott DJ. Endovascular aneurysm repair in nonagenarians: a systematic review. Annals of Vascular Surgery. 2015;29(2):385-91.
706	Wilhelmson K, Andersson Hammar I, Westgard T, Holmquist Henrikson L, Dahlin-Ivanoff S. Positive effects on activities of daily living one year after receiving comprehensive geriatric assessment - results from the randomised controlled study CGA-Swed. BMC geriatrics. 2022;22(1):180.

707	Wilhelmson K, Hammar IA, Ehrenberg A, Niklasson J, Eckerblad J, Ekerstad N, et al. Comprehensive Geriatric Assessment for Frail Older People in Swedish Acute Care Settings (CGA-Swed): A Randomised Controlled Study. Geriatrics (Basel, Switzerland). 2020;5(1).
708	Willumsen T, Solemdal K, Wenaasen M, Ogaard B. Stannous fluoride in dentifrice: an effective anti-plaque agent in the elderly? Gerodontology. 2007;24(4):239-43.
709	Wising J, Mattsson G, Rambaree K, Willmer M, Wallhagen M, Magnusson P. 'Life with a device': the octogenarians' experiences with an implantable cardioverter-defibrillator—a qualitative study. European Journal of Cardiovascular Nursing. 2022;21(2):161-8.
710	Witham MD, Argo IS, Johnston DW, Struthers AD, McMurdo ME. Long-term follow-up of very old heart failure patients enrolled in a trial of exercise training. American Journal of Geriatric Cardiology. 2007;16(4):243-8.
711	Wolf SL, O'Grady M, Easley KA, Guo Y, Kressig RW, Kutner M. The influence of intense Tai Chi training on physical performance and hemodynamic outcomes in transitionally frail, older adults. The journals of gerontology Series A, Biological sciences and medical sciences. 2006;61(2):184-9.
712	Wolf SL, Sattin RW, Kutner M. Intense Tai Chi exercise training and fall occurrences in older, transitionally frail adults. Journal of the American Geriatrics Society. 2003;51(12).
713	Won H, Her AY, Kim BK, Kim YH, Shin DH, Kim JS, et al. Percutaneous Coronary Intervention Is More Beneficial Than Optimal Medical Therapy in Elderly Patients with Angina Pectoris. Yonsei Medical Journal. 2016;57(2):382-7.
714	Wubbeke LF, Naves CCLM, Daemen J-WHC, Jacobs MJ, Mees BME. Editor's Choice - Mortality and Major Amputation after Revascularisation in Octogenarians Versus Non-Octogenarians with Chronic Limb Threatening Ischaemia: A Systematic Review and Meta-Analysis. European journal of vascular and endovascular surgery : the official journal of the European Society for Vascular Surgery. 2020;60(2):231-41.
715	Xu Y, Wang Y, Xi C, Ye N, Xu X. Is it safe to perform gastrectomy in gastric cancer patients aged 80 or older?: A meta-analysis and systematic review. Medicine. 2019;98(24):e16092.
716	Yamada Y, Uchida T, Sasaki S, Taguri M, Shiose T, Ikenoue T, et al. Nudge-Based Interventions on Health Promotion Activity Among Very Old People: A Pragmatic, 2-Arm, Participant-Blinded Randomized Controlled Trial. Journal of the American Medical Directors Association. 2023;24(3):390-4.e5.
717	Yeo AL, Levy D, Martin FC, Sonksen P, Sturgess I, Wheeler MM, et al. Frailty and the biochemical effects of recombinant human growth hormone in women after surgery for hip fracture. Growth hormone & IGF research : official journal of the Growth Hormone Research Society and the International IGF Research Society. 2003;13(6):361-70.
718	Yeun Y, Lee J. Effect of a double-coated probiotic formulation on functional constipation in the elderly: a randomized, double blind, controlled study. Archives of pharmacal research. 2015;38(7):1345-50.
719	Yung-Chi C, Yen-Chi L, Ling-Yu H. Effects of the Otago Exercise Program on Lower Extremity Strength in Residents of a Long-Term Care Institution. Journal of Nursing. 2020;67(3):48-55.
720	Zanchetti A, Elmfeldt D. Findings and implications of the Study on COgnition and Prognosis in the Elderly (SCOPE) - a review. Blood Pressure. 2006;15(2):71-9.
721	Zarralanga-Lasobras T, Romero-Estarlich V, Carrasco-Paniagua C, Serra-Rexach JA, Mayordomo-Cava J. "Inspiratory muscle weakness in acutely hospitalized patients 75 years and over": a secondary analysis of a randomized controlled trial on the effectiveness of multicomponent exercise and inspiratory muscle training. European geriatric medicine. 2023.

722	Zarzeczny R, Nawrat-Szoltysik A, Polak A. Effects of 12 weeks of neuromuscular electrical stimulation of the quadriceps muscles on the function and physio-biochemical traits in functionally fit female nursing-home residents aged 75 + years: a pilot study. European journal of applied physiology. 2023.
723	Zbroński K, Grodecki K, Gozdowska R, Ostrowska E, Wysińska J, Rymuza B, et al. Protamine sulfate during transcatheter aortic valve implantation (PS TAVI) - a single-center, single-blind, randomized placebo-controlled trial. Kardiologia polska. 2021;79(9):995-1002.
724	Zech A, Drey M, Freiberger E, Hentschke C, Bauer JM, Sieber CC, et al. Residual effects of muscle strength and muscle power training and detraining on physical function in community-dwelling prefrail older adults: a randomized controlled trial. BMC geriatrics. 2012;12:68.
725	Zhang C, Gu Z-C, Shen L, Pan M-M, Yan Y-D, Pu J, et al. Non-vitamin K antagonist oral anticoagulants and cognitive impairment in atrial fibrillation: Insights from the meta-analysis of over 90,000 patients of randomized controlled trials and real-world studies. Frontiers in Aging Neuroscience. 2018;10.
726	Zhang L, Weng C, Liu M, Wang Q, Liu L, He Y. Effect of whole-body vibration exercise on mobility, balance ability and general health status in frail elderly patients: a pilot randomized controlled trial. Clinical rehabilitation. 2014;28(1):59-68.
727	Zhang Y, Zhang Y, Du S, Wang Q, Xia H, Sun R. Exercise interventions for improving physical function, daily living activities and quality of life in community-dwelling frail older adults: A systematic review and meta-analysis of randomized controlled trials. Geriatric nursing (New York, NY). 2020;41(3):261-73.
728	Zheng A, Sakari R, Cheng SM, Hietikko A, Moilanen P, Timonen J, et al. Effects of a low-frequency sound wave therapy programme on functional capacity, blood circulation and bone metabolism in frail old men and women. Clinical rehabilitation. 2009;23(10):897-908.
729	Zheng L, Li G, Wang X, Yin H, Jia Y, Leng M, et al. Effect of exergames on physical outcomes in frail elderly: a systematic review. Aging clinical and experimental research. 2020;32(11):2187-200.
730	Zou L, Han J, Li C, Yeung AS, Hui SS, Tsang WWN, et al. Effects of Tai Chi on Lower Limb Proprioception in Adults Aged Over 55: A Systematic Review and Meta-Analysis. Archives of Physical Medicine & Rehabilitation. 2019;100(6):1102-13.
731	Zwijsen SA, Depla MFIA, Niemeijer AR, Francke AL, Hertogh CMPM. The concept of restraint in nursing home practice: a mixed-method study in nursing homes for people with dementia. International Psychogeriatrics. 2011;23(5):826-34.

APPENDIX (6) Quality Appraisal AMSTAR-2

AMSTAR 2

*indicates key/critical domains (decided *a priori*), used to determine the overall study rating – see questions provided below

2. Did the report of the review contain an explicit statement that the review methods were established prior to the conduct of the review and did the report justify any significant deviations from the protocol?
4. Did the review authors use a comprehensive literature search strategy?
9. Did the review authors use a satisfactory technique for assessing the risk of bias (RoB) in individual studies that were included in the review?
11. If meta-analysis was performed did the review authors use appropriate methods for statistical combination of results?
13. Did the review authors account for RoB in individual studies when interpreting/ discussing the results of the review?

KEY	+	Yes	?	Partial Yes	-	No	**NA**	Not applicable

**Author**	1	2*	3	4*	5	6	7	8	9a*	9b*	10	11a*	11b*-	**12**	**13***	**14**	**15***	**16**	**Overall rating**
Bai 2021	+	-	+	?	+	+	-	?	NA	+	-	NA	+	+	-	+	+	+	Low
Barssoum 2021	+	-	-	?	+	+	-	+	+	+	-	NA	-	+	-	+	-	+	Low
Bhatnager 2011	+	-	+	-	+	-	-	+	NA	-	-	NA	+	-	+	+	+	-	Critically Low

Biancari 2017	+	-	+	-	+	-	-	+	NA	-	-	NA	+	-	+	-	-	+	Critically Low
Briand 2022	+	-	+	-	-	-	-	+	NA	-	-	-	-	NA	-	+	NA	-	Critically Low
Chen 2023	+	-	-	-	-	+	-	?	NA	+	-	NA	+	NA	-	+	+	+	Critically Low
Courage 2021	+	+	-	+	+	+	-	+	NA	+	+	NA	+	-	+	+	-	+	Moderate
DeBernardis (2023)	-	-		+	+	-	-	-	NA	?	-	NA	NA	NA	+	+	NA	+	Critically Low
Duffis 2013	-	-	-	-	-	-	-	?	NA	-	-	NA	-	-	+	+	+	-	Critically Low
Elahwal (2023)	-	-	+	?	+	+	-	+	NA	+	-	NA	NA	NA	-	+	NA	-	Low
Engelter (2006)	+	-	+	-	-	+	-	+	NA	+	-	NA	-	-	+	+	-	+	Critically Low
Gallingani (2022)	+	-	+	?	+	+	-	+	NA	+	-	NA	-	+	+	+	+	+	Low

Haas (2017)	+	-	-	?	+	+	-	?	NA	+	-	NA	NA	NA	-	-	NA	+	Critically Low
Hajibandeh (2021)	+	-	+	+	+	+	-	?	NA	+	-	-	-	-	+	+	+	+	Low
Iqbal (2022)	+	-	+	?	+	+	+	+	NA	+	-	NA	-	-	+	-	+	+	Critically Low
Johnman (2013)	+	-	+	-	+	-	-	?	NA	-	-	NA	NA	NA	+	-	NA	+	Critically Low
Kim (2017)	+	-	+	-	-	-	-	+	NA	-	-	NA	-	-	-	-	-	+	Critically Low
Lee 2022	+	+	+	+	+	+	-	+	+	+	-	+	+	+	+	+	+	+	Moderate
Liang (2020)	+	-	+	?	+	+	-	+	NA	NA	-	NA	-	+	+	+	+	+	Low
Lin 2022	+	-	+	-	-	-	-	+	NA	NA	-	NA	-	-	-	-	-	+	Critically Low
Marcellaud (2023)	+	-	+	-	-	-	-	+	+	+	-	NA	NA	NA	-	-	NA	+	Critically Low

Mende 2022	+	+	+	+	+	+	-	+	+	NA	+	NA	NA	-	+	-	-	+	Moderate
Merga 2023	+	+	+	?	+	+	-	+	NA	+	-	NA	+	+	+	+	+	-	Moderate
Nana 2023	+	+	+	?	+	+	-	+	NA	+	-	NA	+	-	+	+	-	+	Moderate
**Nicolson (2021)**	+	-	+	+	+	+	-	+			-	-	NA	-	+	-	-	+	Low
**Noguchi (2022)**	+	-	+	-	+	-	-	+	NA	-	-	NA	-	-	+	+	-	-	Critically Low
**Owen (2022)**	-	-	-	?	+	-	-	?	+	+	-	NA	NA	NA	-	-	NA	-	Low
**Rautalin (2021)**	+	-	+	?	-	-	-	+	NA	?	-	NA	NA	NA	-	-	NA	+	Low
**Shantsila (2023)**	+	-	+	?	+	-	-	-	NA	-	-	NA	NA	NA	-	-	-	-	Critically Low
**Sun (2022)**	+	-	+	-	+	+	-	+	NA	+	-	NA	+	+	+	+	+	+	Low

**van Halsema (2021)**	-	-	+	?	+	+	-	+	NA	+	+	NA	NA	NA	+	+	NA	+	Low
**Wang (2023)**	+	-	+	-	+	+	-	+	NA	+	-	NA	-	-	+	+	-	+	Low
**Watkins (2022)**	+	-	-	-	+	+	-	?	NA	-	-	NA	NA	NA	+	-	-	-	Critically Low
**Wubbeke (2020)**	+	-	-	?	+	-	-	?	NA	+	+	NA	-	-	+	+	-	+	Low
**Xu (2019)**	+	-	-	-	-	+	-	+	NA	+	-	NA	-	-	+	+	+	+	Critically Low
**Yan (2019)**	+	-	+	-	+	+	-	-	NA	+	-	NA	+	-	-	-	-	+	Low

APPENDIX (7) Risk of Bias summaries for RCTs

	Random sequence generation	Allocation concealment	Blinding of participants and personnel	Blinding of outcome assessment	Incomplete outcome data	Selective reporting	Other bias
Akao (2024)	+	+	+	+	+	+	?
Akashi (2022)	+	+	+	+	+	+	?
Alassaad (2014)	+	+	-	+	+	+	+
Álvarez-Barbosa (2014)	+	+	-	?	+	+	+
Ansai (2015)	+	+	-	-	?	+	-
Ansai (2016)	+	+	-	?	?	+	-
Barbosa (2016)	+	?	-	+	+	+	+
Barker (2006)	?	+	+	+	+	+	+
Bays-Moneo (2023)	+	+	-	?	+	+	+
Bechshoft (2017)	+	+	-	+	+	+	+
Beckett (2008)	+	+	+	+	+	+	+
Beckett (2011)	+	+	+	+	+	+	+
Behm (2014)	+	?	-	+	+	+	+
Behm (2016)	+	+	-	+	+	+	+
Berg (2023)	+	+	-	-	+	+	+

Boonen (2004)	+	+	+	+	+	+	?
Brettschneider (2015)	+	+	-	-	+	+	+
Bruunsgaard (2004)	?	?	-	?	?	+	+
Bulpitt (2003)	+	+	-	-	+	+	+
Bulpitt (2012)	+	+	+	+	+	+	+
Bulpitt (2013)	+	+	+	+	+	+	+
Buondonno (2020)	+	?	-	+	?	+	+
Burrows (2002)	?	?	+	?	+	+	?
Burtin (1995)	?	?	-	-	?	+	?
Cadore (2014)	+	+	-	+	?	+	?
Calder (1996)	+	+	+	-	+	+	+
Campbell (1997)	+	+	-	?	?	?	?
Campbell (1999)	?	?	-	-	?	+	+
Campo-Prieto (2022)	?	?	?	?	+	+	+
Carral (2019)	?	?	?	?	+	+	+
Chammout (2019)	+	+	+	?	+	+	+
Clough (2019)	+	+	+	+	-	+	+
Cofre-Bolados (2023)	?	?	-	-	+	+	-
de Belder (2014)	+	+	+	+	+	+	+

de Belder (2021)	+	+	-	?	?	+	?
Dodge (2008)	+	?	+	+	+	?	+
Ehsani (2003)	+	+	-	?	+	+	+
Eriksen (2018)	+	+	-	+	+	+	+
Ferrer (2014)	+	+	-	+	?	+	+
Freixa (2012)	?	?	+	-	?	+	?
Fu (2020)	+	?	-	?	?	?	+
Gavazzi (2022)	+	?	?	-	-	+	-
Gene (2018)	+	?	-	-	?	+	?
Gillespie (2009)	+	+	-	?	+	+	?
Gillespie (2013)	+	+	-	?	+	+	?
Gine-Garriga (2010)	?	?	-	?	+	+	+
Giné-Garriga (2013)	?	?	-	?	+	+	+
Godwin (2016)	?	+	-	-	?	+	+
Greenspan (2020)	+	+	+	+	+	+	?
Greuter (2020)	+	+	-	+	+	?	+
Gustafsson (2012)	+	+	-	+	?	+	+
Gustafsson (2013)	+	+	-	+	?	+	+
Haanes (2015)	?	?	-	-	?	+	+

Hagiwara (2019)	?	?	?	-	?	+	+
Han (2017)	+	+	+	+	+	+	+
Hedbeck (2011)	+	+	+	-	+	+	+
Hilt (2023)	?	?	-	?	+	+	+
Hirlekar (2020)	+	+	?	?	+	+	-
Holland (2005)	+	+	-	+	+	+	+
Imhof (2012)	+	+	-	?	+	+	+
Kalapotharakos (2010)	?	?	-	?	+	+	+
Karlsen (2019)	+	+	-	+	+	+	+
Khatri (2022)	+	+	+	?	+	+	+
Kryger (2007)	?	?	-	?	+	+	+
Lenaghan (2007)	?	?	-	+	+	+	+
Liang (2020)	+	+	-	+	+	+	?
Lin (2009)	?	+	-	+	+	+	+
Lopiz (2019)	+	+	-	+	+	+	+
Lorenzano (2021)	+	+	?	-	+	+	?
Louvard (2004)	?	+	?	?	+	+	+
Lu (2017)	+	?	?	+	+	+	+
Luck (2013)	+	?	-	-	-	+	+

Luukinen (2007)	+	+	-	+	+	+	?
Malaguarnera (2007)	+	+	+	+	+	+	?
McClung (2018)	+	+	+	+	+	+	?
McCord (2020)	+	+	-	?	+	+	+
Meng (2015)	+	+	?	+	?	+	+
Mikami (2022)	+	+	+	+	+	+	?
Minai (2002)	+	+	+	+	+	+	?
Namkoong (2015)	+	+	?	-	+	+	+
Oberic (2021)	?	?	?	-	+	+	+
Okazaki (2022)	+	+	+	+	+	+	?
Okumura (2020)	+	+	+	+	+	+	?
Oliveira (2016)	?	?	-	?	+	+	?
Ouwehand (2008)	?	?	?	?	?	+	+
Pajewski (2019)	+	+	-	+	?	+	?
Pershad (2014)	+	+	+	?	?	?	+
Peters (2008)	+	+	+	+	+	+	?
Peters (2010)	+	+	+	+	+	+	?
Peters (2013)	+	+	+	+	+	+	?
Puggaard (1999)	?	?	-	-	?	-	+

Puggaard (2000)	?	?	-	-	?	+	?
Rabin (2016)	+	+	+	+	?	+	?
Rash (2007)	+	+	-	?	+	+	?
Rimon (2005)	+	?	?	+	+	+	?
Rodrigues (2022)	?	?	-	?	+	+	+
Rosado (2008)	?	?	-	?	?	+	+
Rosie (2007)	+	+	+	+	+	+	+
Ruiz (2015)	+	?	-	+	+	+	+
Seeman (2006)	+	+	+	+	+	+	?
Seeman (2010)	+	+	+	+	+	+	?
Seinela (2003)	?	?	-	?	+	+	+
Serra-Rexach (2011)	+	+	-	+	+	+	+
Sheppard (2020)	+	+	-	-	?	+	?
Tegn (2016)	+	+	-	-	+	+	+
Tegn (2018)	+	+	-	-	+	+	+
Tegn (2020)	+	+	-	-	+	+	+
Thommessen (2021)	+	+	+	?	+	+	?
Vorilhon (2016)	+	+	-	-	?	+	+
Wang (2010)	+	+	-	-	+	+	?

Warwick (2015)	+	+	+	+	+	+	+
West (2020)	+	+	+	+	?	+	?
Wilhelmson (2013)	+	+	-	-	-	+	+
Xu (2022)	+	+	+	+	+	+	?
Yamashita (2009)	+	?	+	+	+	+	?
Yamashita (2024)	+	+	+	+	+	+	?
Yoshida (2022)	+	+	+	+	+	+	?
Zak (2006)	+	+	?	-	-	+	?
Zhou (2020)	+	?	?	?	?	+	+
Zidén (2014)	?	?	?	+	+	+	+

ROB DISTRIBUTION

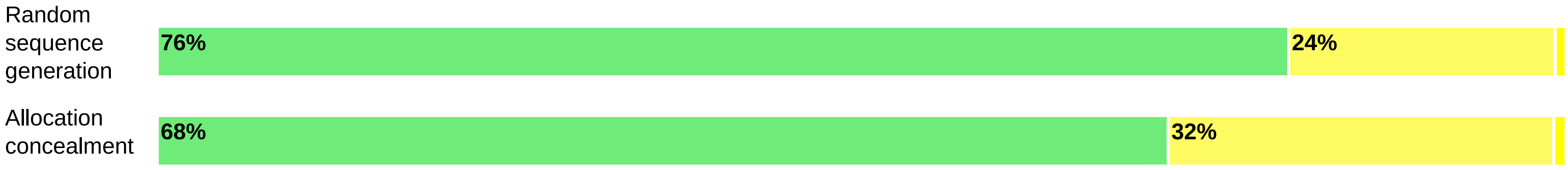

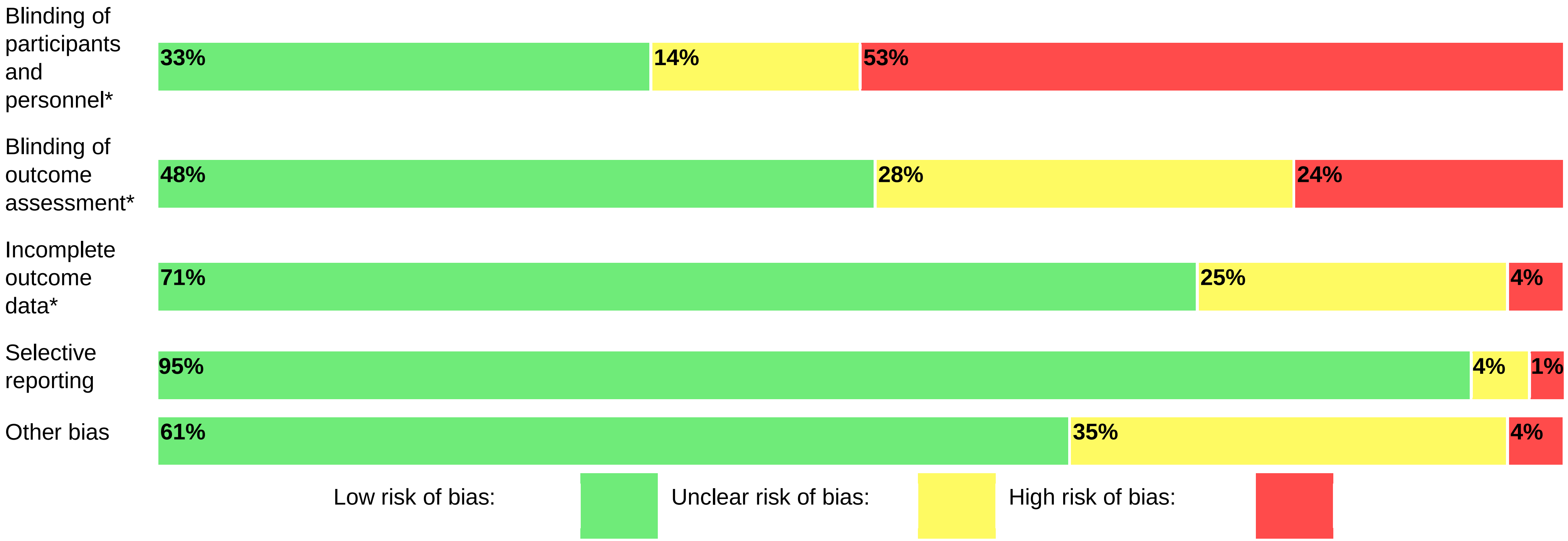
Blinding of participants and personnel*
33%
14%
53%
Blinding of outcome assessment*
48%
28%
24%
Incomplete outcome data*
71%
25%
4%
Selective reporting
95%
4%
1%
Other bias
61%
35%
4%
Low risk of bias:
Unclear risk of bias:
High risk of bias:

APPENDIX (8) – Quality appraisal summaries of qualitative studies (Wallace Criteria)

Author(s)	Is the research question clear?	Theory/ ideology explicit?	Theory / ideology influenced the study design?	Is the study design appropriate?	Is the context adequately described?	Sample adequate and drawn from appropriate population?	Data collection adequately described?	Data collection rigorously conducted to ensure confidence	Data analysis rigorously conducted?	Findings substantiated by the data	Limitations of methods considered?	Claims to generalizability follow from data?	Ethical issues addressed?	Author(s) relflexive?
Amofah	Y	N	N	Y	Y	Y	Y	Y	Y	Y	Y	Y	Y	N
Behm	Y	Y	Y	Y	Y	Y	Y	Y	Y	Y	Y	Y	Y	Y
Berkhout	Y	N	N	Y	Y	Y	N	Y	Y	Y	Y	Y	Y	N
Burden	Y	N	N	Y	Y	N	N	CT	N	Y	N	N	N	N
Bynum	Y	N	N	Y	Y	Y	Y	CT	N	Y	Y	Y	Y	N
Chen	Y	N	N	Y	Y	Y	N	CT	CT	CT	N	Y	Y	N
Eriksen	Y	N	N	Y	Y	Y	Y	CT	Y	Y	Y	Y	Y	N
Ganske	Y	Y	Y	Y	Y	Y	Y	Y	Y	Y	Y	Y	Y	Y
Instenes	Y	Y	Y	Y	Y	Y	Y	Y	N	Y	Y	Y	Y	N
Kylberg	Y	N	N	Y	Y	N	Y	Y	N	Y	Y	Y	Y	N
Lofqvist	Y	Y	Y	Y	Y	Y	Y	Y	Y	Y	Y	Y	Y	Y
McCombie	Y	N	N	CT	Y	Y	N	N	N	CT	Y	N	Y	N
Salter	Y	N	N	Y	Y	Y	Y	N	Y	Y	Y	N	Y	N
Schonberg	Y	N	N	Y	Y	Y	N	CT	CT	CT	Y	Y	Y	N
Tomsone	Y	N	N	Y	Y	N	Y	Y	Y	Y	Y	Y	Y	Y
Wising	Y	Y	Y	Y	Y	Y	Y	Y	Y	Y	N	Y	N	N
